# Supplementary material for: Concurrent mutations in RNA-dependent RNA polymerase and spike protein emerged as the epidemiologically most successful SARS-CoV-2 variant
Source: Sci Rep. 2021 Jul 1;11:13705. doi: 10.1038/s41598-021-91662-w (PMC8249556; doi:10.1038/s41598-021-91662-w)
Supplement: Supplementary file 1 — Supplementary Legends. [file 41598_2021_91662_MOESM1_ESM.docx]

**Concurrent mutations in RNA-dependent RNA polymerase and spike protein emerged as the epidemiologically most successful SARS-CoV-2 variant**

Sten Ilmjärv^1^, Fabien Abdul^2^, Silvia Acosta-Gutiérrez^3,4,5^, Carolina Estarellas^3^, Ioannis Galdadas^3^, Marina Casimir^6^, Marco Alessandrini^1^, Francesco Luigi Gervasio^3,5,7,8^†, Karl-Heinz Krause^1, 9^†*

**Supplementary information**

**Supplementary Figure 1. Heat maps representing the pairwise root-mean-square-deviation (RMSD) matrices for RDB and the NTD domains of each the protomers of the wild-type S-protein in the open (magma palette) and closed conformation (virdis palette)**. Pairwise RMSD matrices were calculated using the python package MDAnalysis ^32-35^.

**Supplementary Figure 2. Heat maps representing the pairwise root-mean-square-deviation (RMSD) matrices for RDB and the NTD domains of each the protomers of the D614G S-protein in the open conformation (magma palette).**

Matrix calculated from a 600ns molecular dynamics simulation initiated in the ‘up’ state (PDBid 6VXX) replacing D614 for glycine in the three protomers of the oligomer. For consistency the simulations used the Amber ff99SB-ILDN force field ^28^ for proteins, the TIP3P model ^29^ for water, and the Amber Glycam force field ^30,31^ for the glycosylated parts of the system. Pairwise RMSD matrices were calculated using the python package MDAnalysis ^32-35^.

**Supplementary Figure 3. Worldwide counts of specific amino acid variants in S-protein and RdRp.**

**Supplementary Figure 4. Weekly number of mutation variants of both double and single mutations in different countries.** Only countries with at least 20 sequences are shown.

**Supplementary Table 1.** **GISAID ^23^ acknowledgment table.**

**References**

23 Elbe, S. & Buckland-Merrett, G. Data, disease and diplomacy: GISAID's innovative contribution to global health. *Glob Chall* **1**, 33-46, doi:10.1002/gch2.1018 (2017).

28 Lindorff-Larsen, K. *et al.* Improved side-chain torsion potentials for the Amber ff99SB protein force field. *Proteins* **78**, 1950-1958, doi:10.1002/prot.22711 (2010).

29 Jorgensen, W., Chandrasekhar, J., Madura, J., Impey, R. & Klein, M. Comparison of Simple Potential Functions for Simulating Liquid Water. *J. Chem. Phys.* **79**, 926-935, doi:10.1063/1.445869 (1983).

30 Kirschner, K. N. *et al.* GLYCAM06: a generalizable biomolecular force field. Carbohydrates. *J Comput Chem* **29**, 622-655, doi:10.1002/jcc.20820 (2008).

31 Website builders, e.g., Carbohydrate Builder, Glycoprotein Builder, etc. *Woods Group. GLYCAM Web. Complex Carbohydrate Research Center, University of Georgia, Athens, GA. (*[*http://glycam.org*](http://glycam.org)*)* (2005-2020).

32 Beckstein, O., Denning, E. J., Perilla, J. R. & Woolf, T. B. Zipping and unzipping of adenylate kinase: atomistic insights into the ensemble of open<-->closed transitions. *J Mol Biol* **394**, 160-176, doi:10.1016/j.jmb.2009.09.009 (2009).

33 Gowers, R. *et al.* *MDAnalysis: A Python Package for the Rapid Analysis of Molecular Dynamics Simulations*. (2016).

34 Michaud-Agrawal, N., Denning, E. J., Woolf, T. B. & Beckstein, O. MDAnalysis: a toolkit for the analysis of molecular dynamics simulations. *J Comput Chem* **32**, 2319-2327, doi:10.1002/jcc.21787 (2011).

35 Theobald, D. Rapid calculation of RMSDs using a quaternion-based characteristic polynomial. *Acta crystallographica. Section A, Foundations of crystallography* **61**, 478-480, doi:10.1107/S0108767305015266 (2005).
