# Supplementary material for: Concurrent mutations in RNA-dependent RNA polymerase and spike protein emerged as the epidemiologically most successful SARS-CoV-2 variant
Source: Sci Rep. 2021 Jul 1;11:13705. doi: 10.1038/s41598-021-91662-w (PMC8249556; doi:10.1038/s41598-021-91662-w)
Supplement: Supplementary file 2 — Supplementary Figure S1. [file 41598_2021_91662_MOESM2_ESM.pdf]

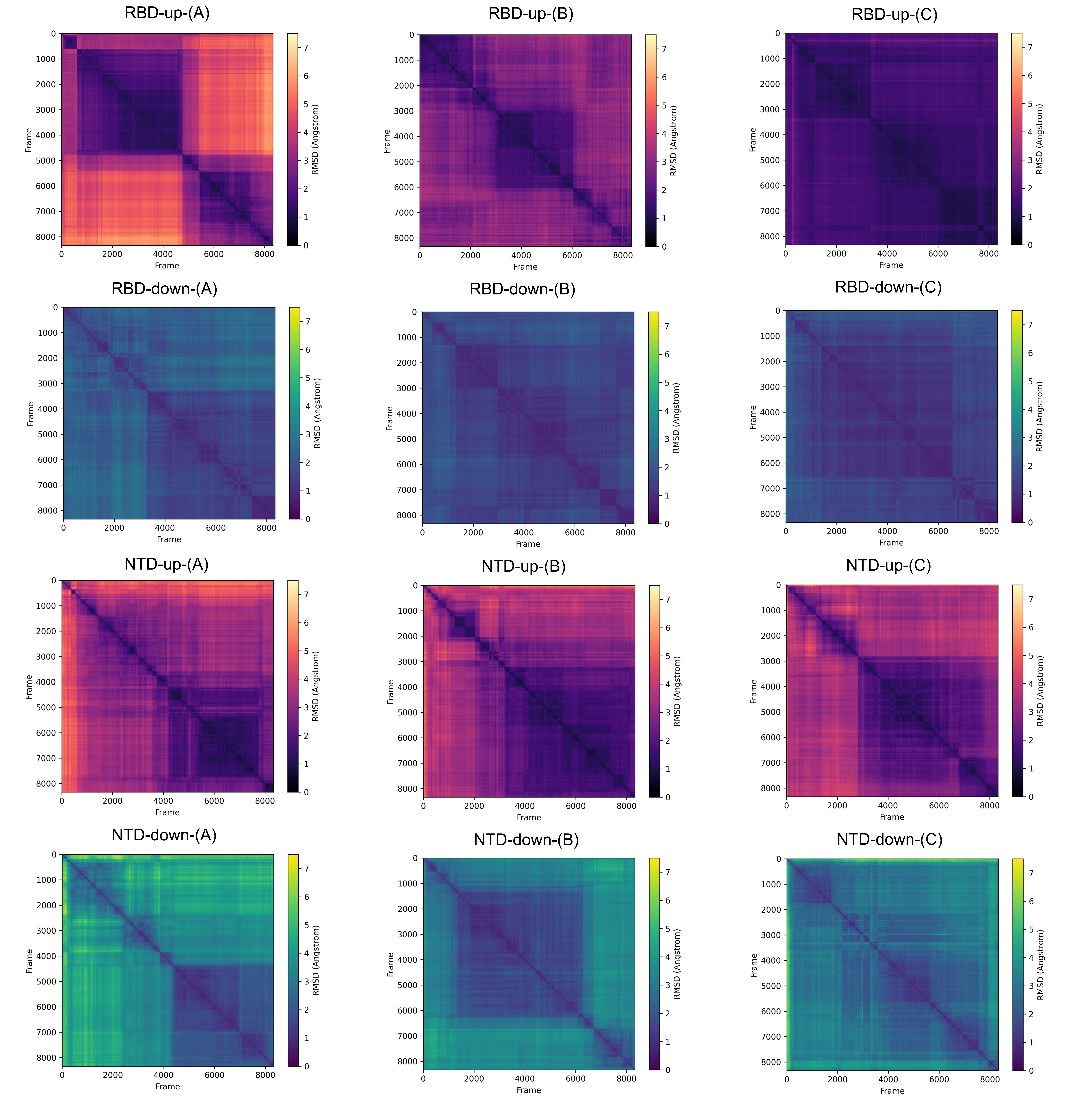
RBD-up-(A)
RBD-up-(B)
RBD-up-(C)
RBD-down-(A)
RBD-down-(B)
RBD-down-(C)
NTD-up-(A)
NTD-up-(B)
NTD-up-(C)
NTD-down-(A)
NTD-down-(B)
NTD-down-(C)
Frame
RMSD (Angstrom)
