# Supplementary figures and images for: Concurrent mutations in RNA-dependent RNA polymerase and spike protein emerged as the epidemiologically most successful SARS-CoV-2 variant
Source: Sci Rep. 2021 Jul 1;11:13705. doi: 10.1038/s41598-021-91662-w (PMC8249556; doi:10.1038/s41598-021-91662-w)

RBD-up-(A)

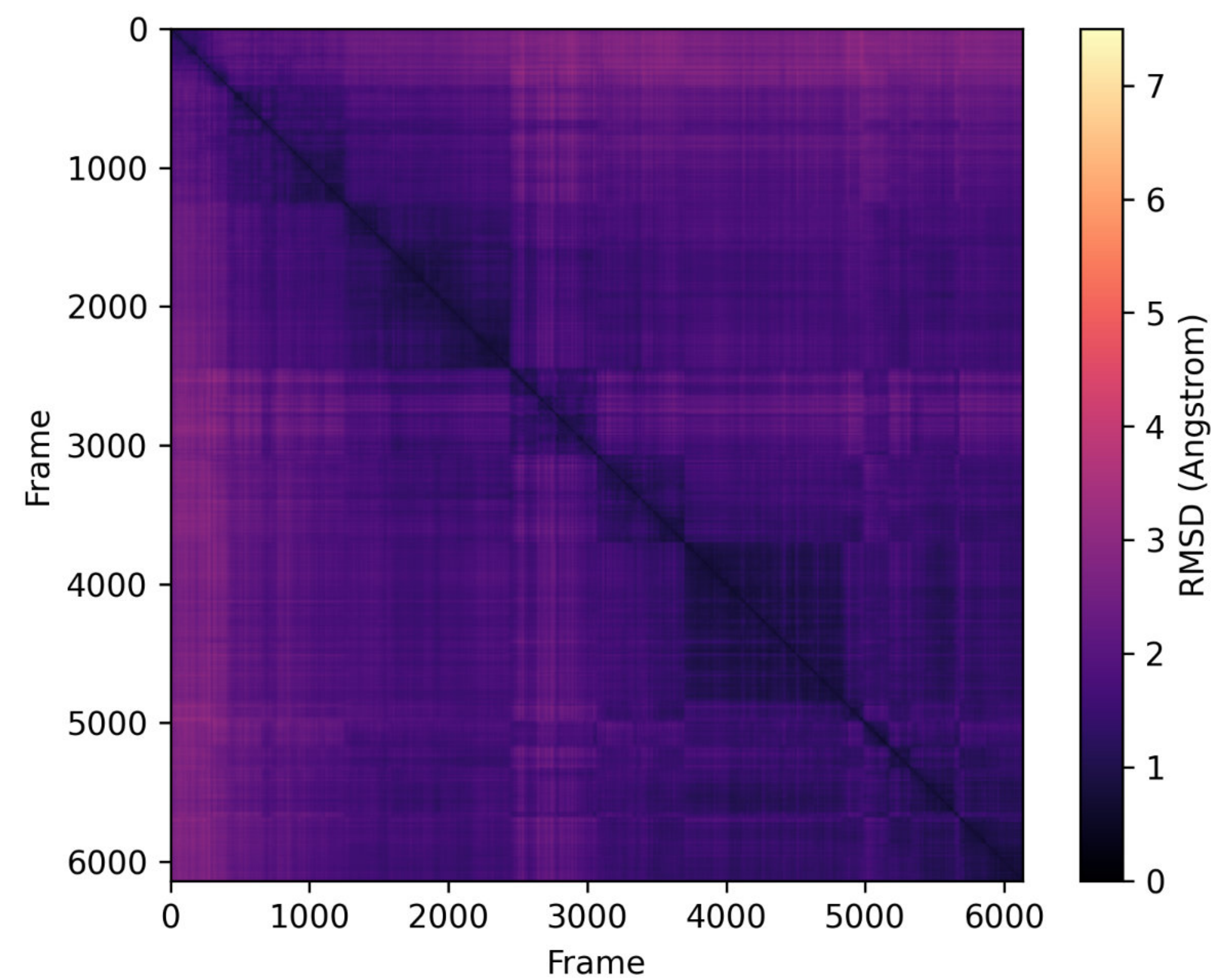

RBD-up-(B)

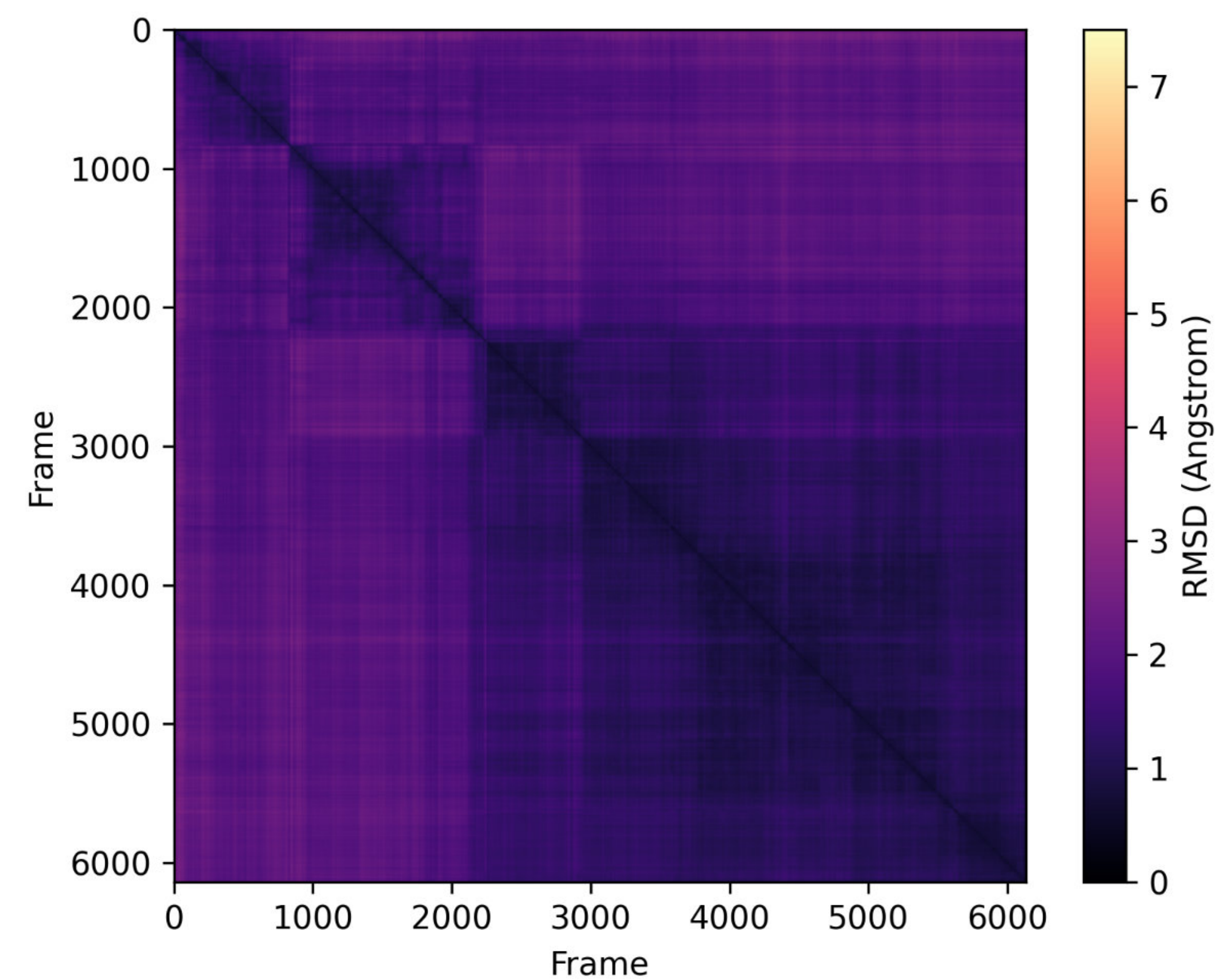

RBD-up-(C)

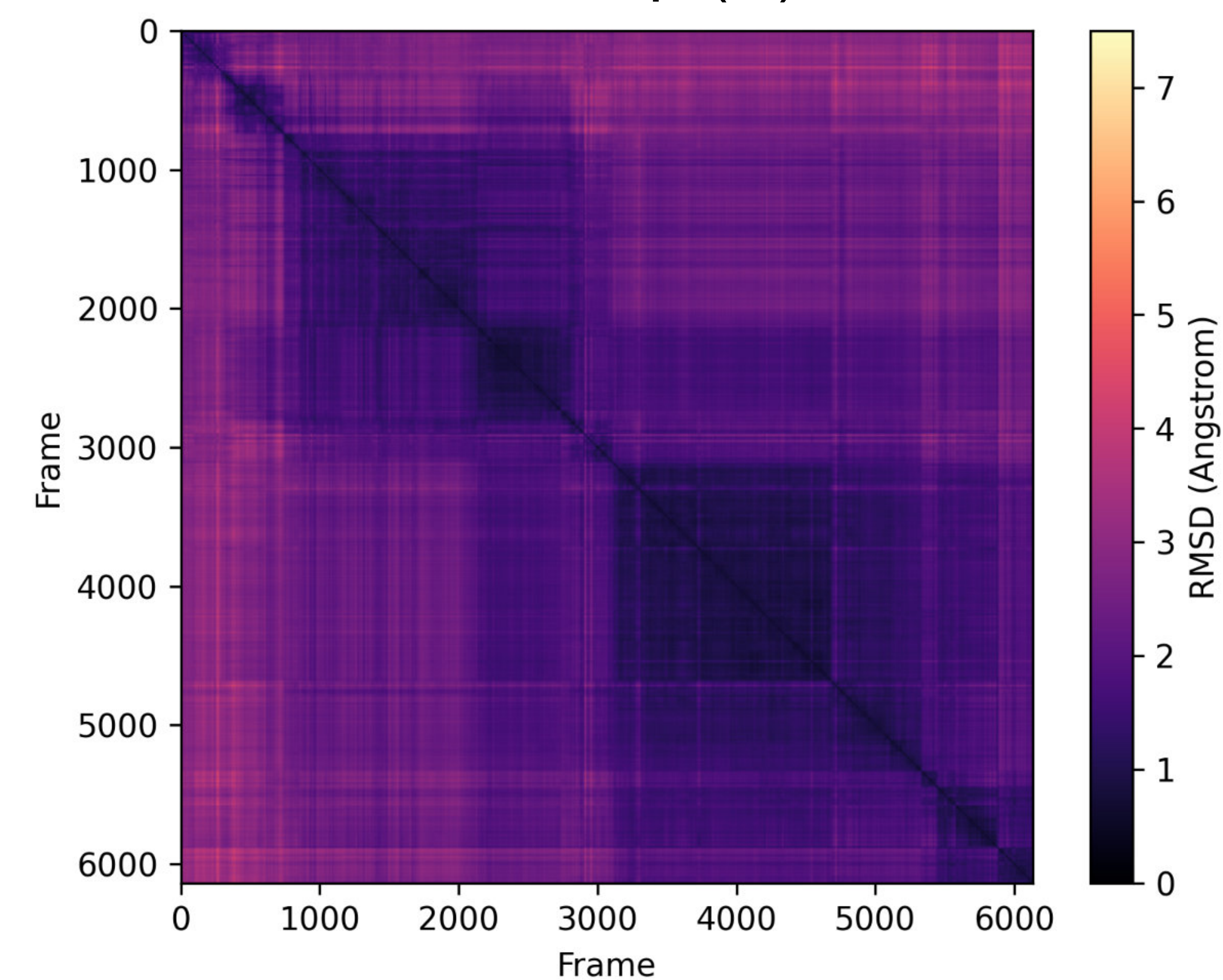

NTD-up-(A)

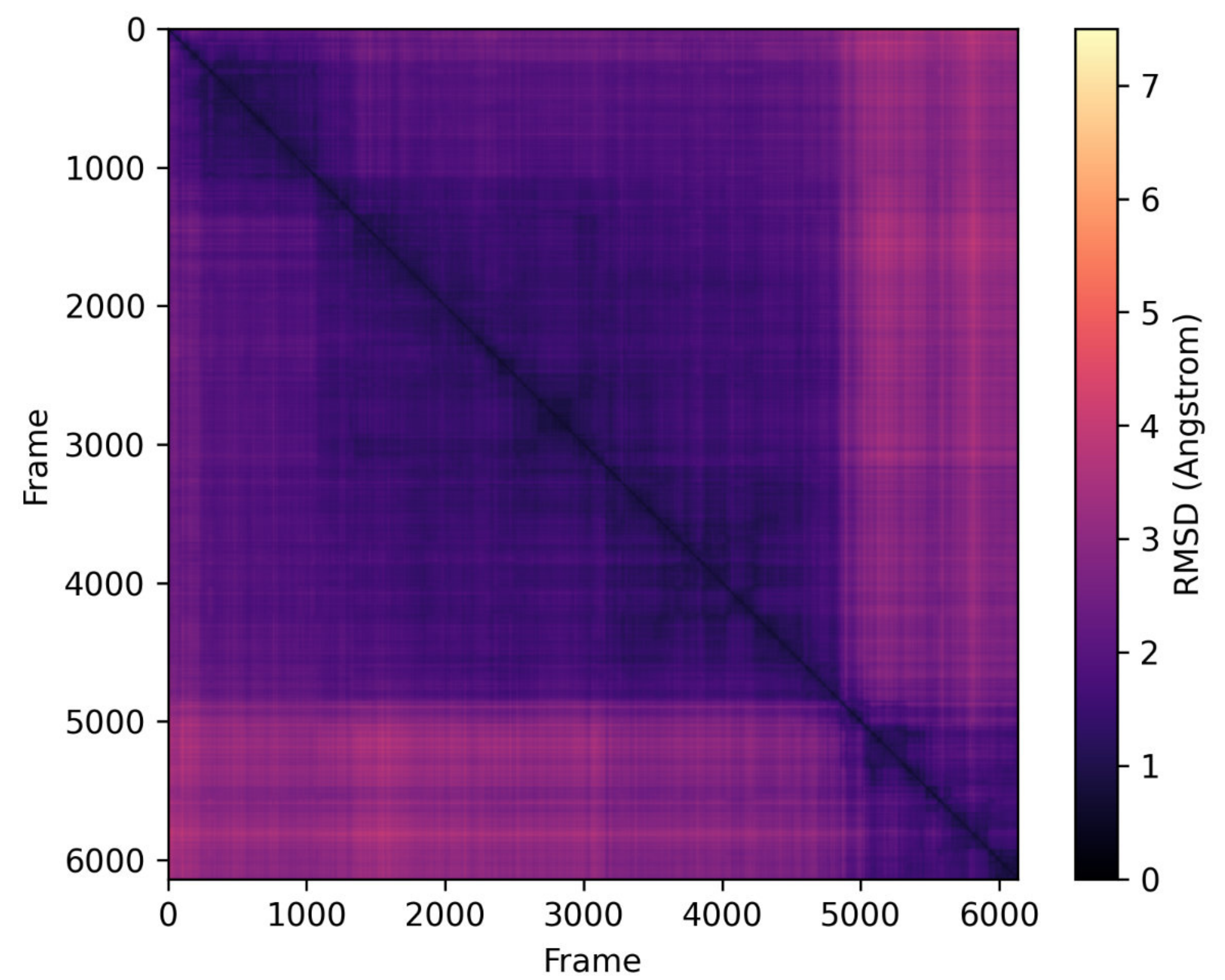

NTD-down-(B)

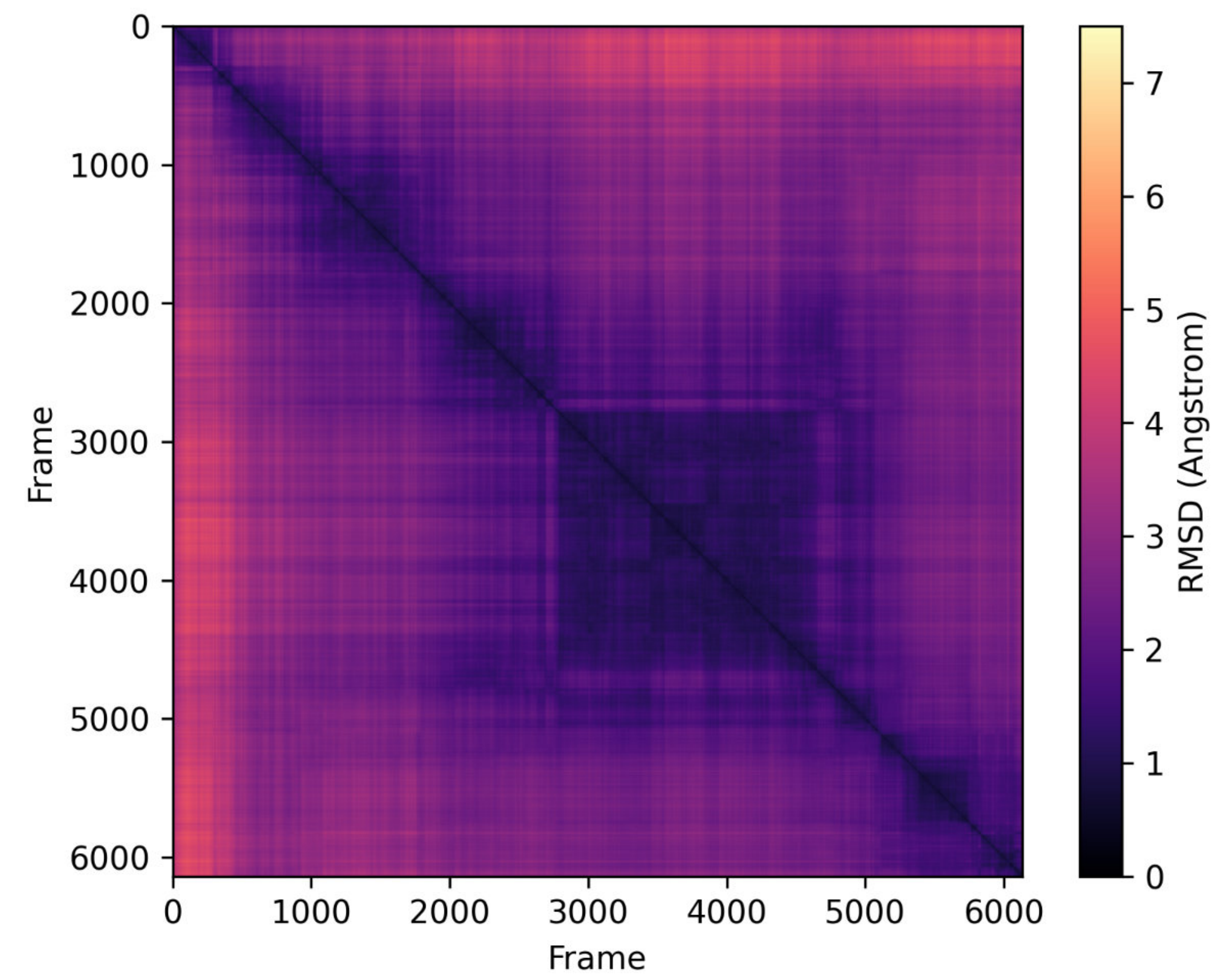

NTD-down-(C)

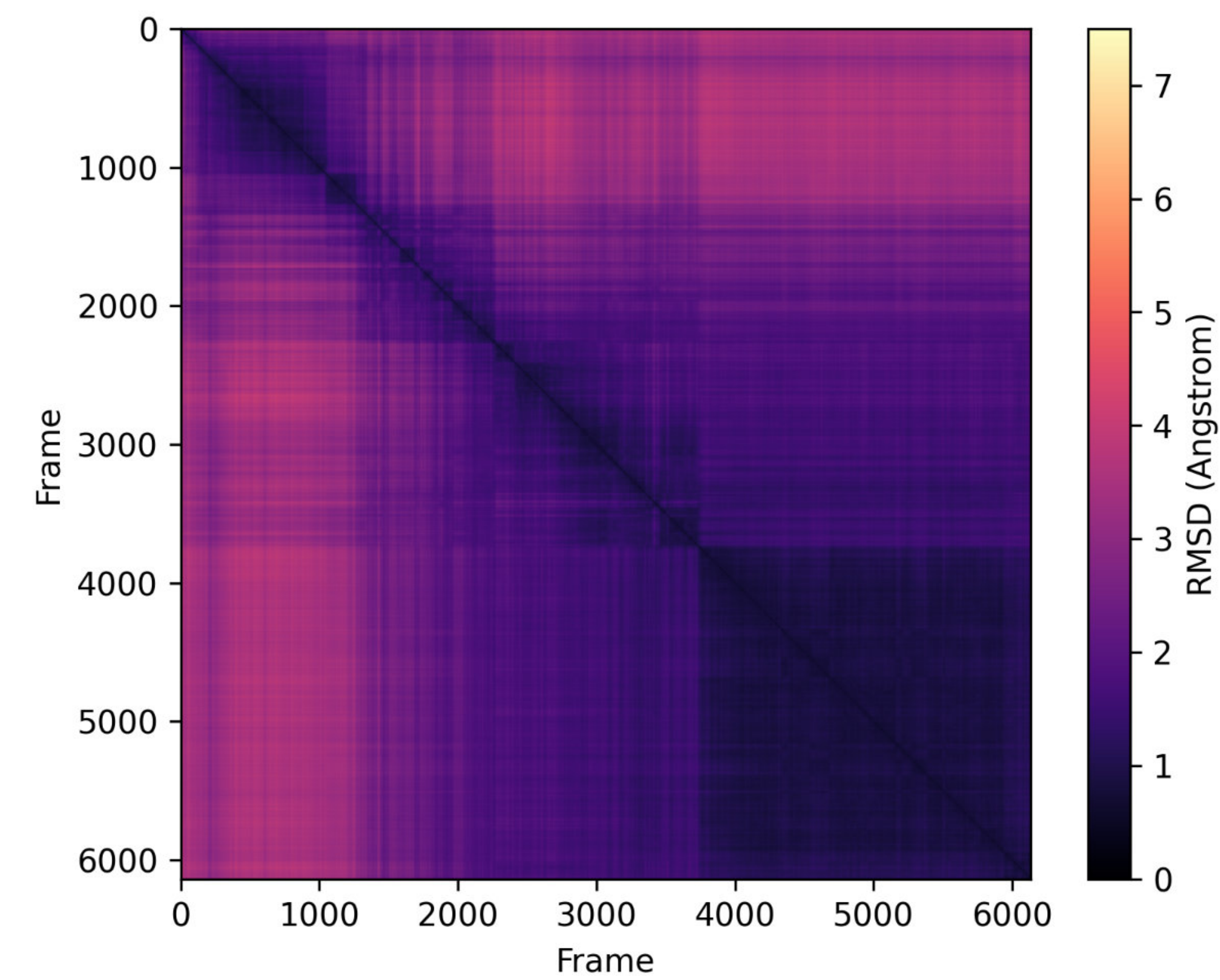

Supplement: Supplementary file 3 — Supplementary Figure S2. [file 41598_2021_91662_MOESM3_ESM.pdf]

**a**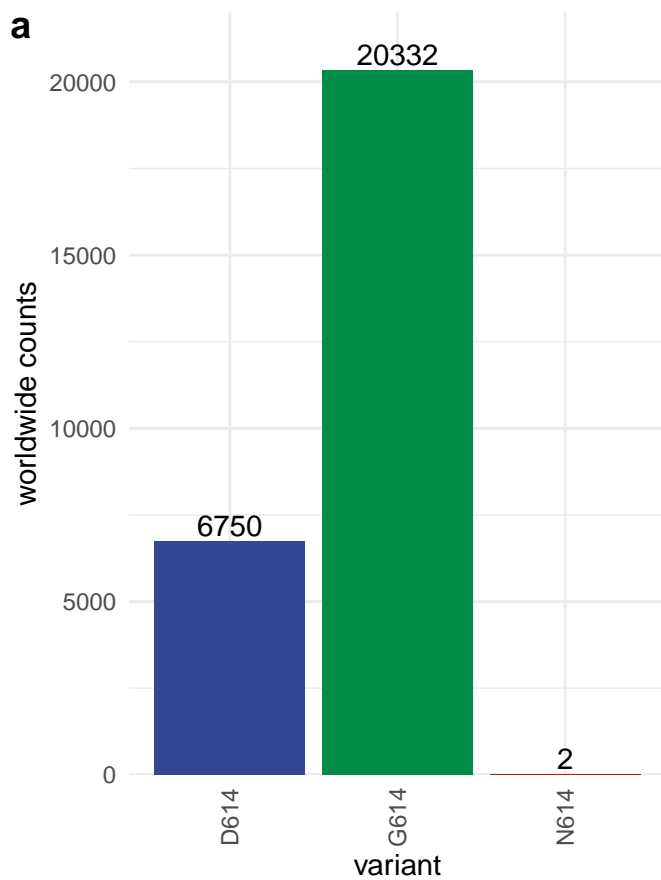**b**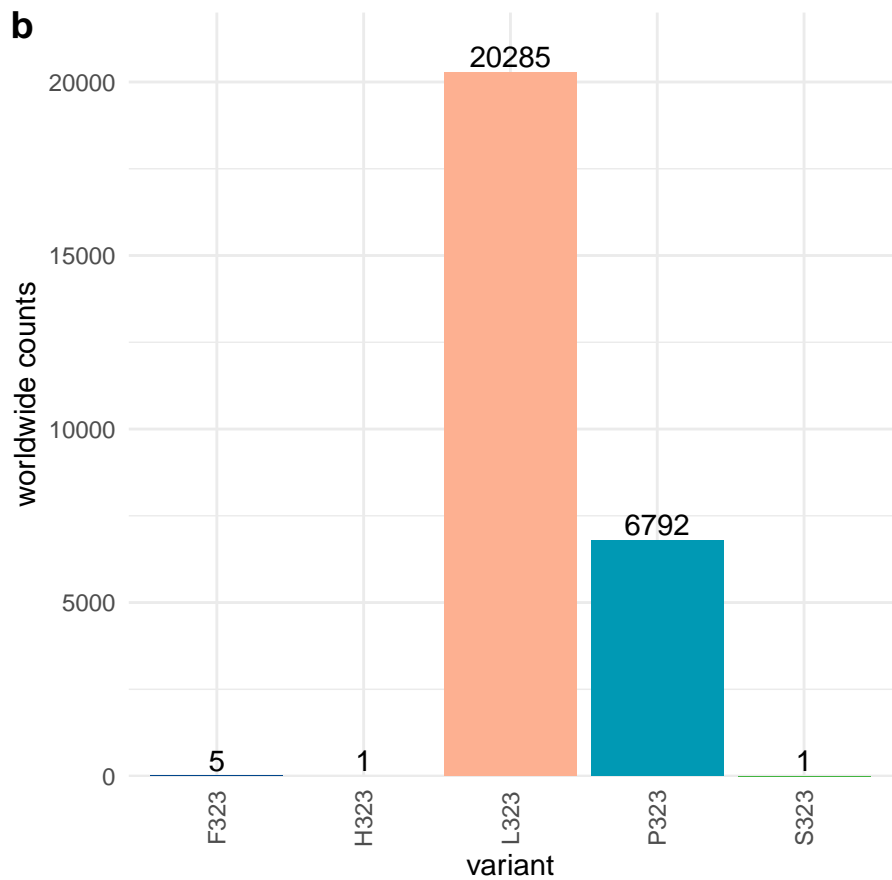

Supplement: Supplementary file 4 — Supplementary Figure S3. [file 41598_2021_91662_MOESM4_ESM.pdf]

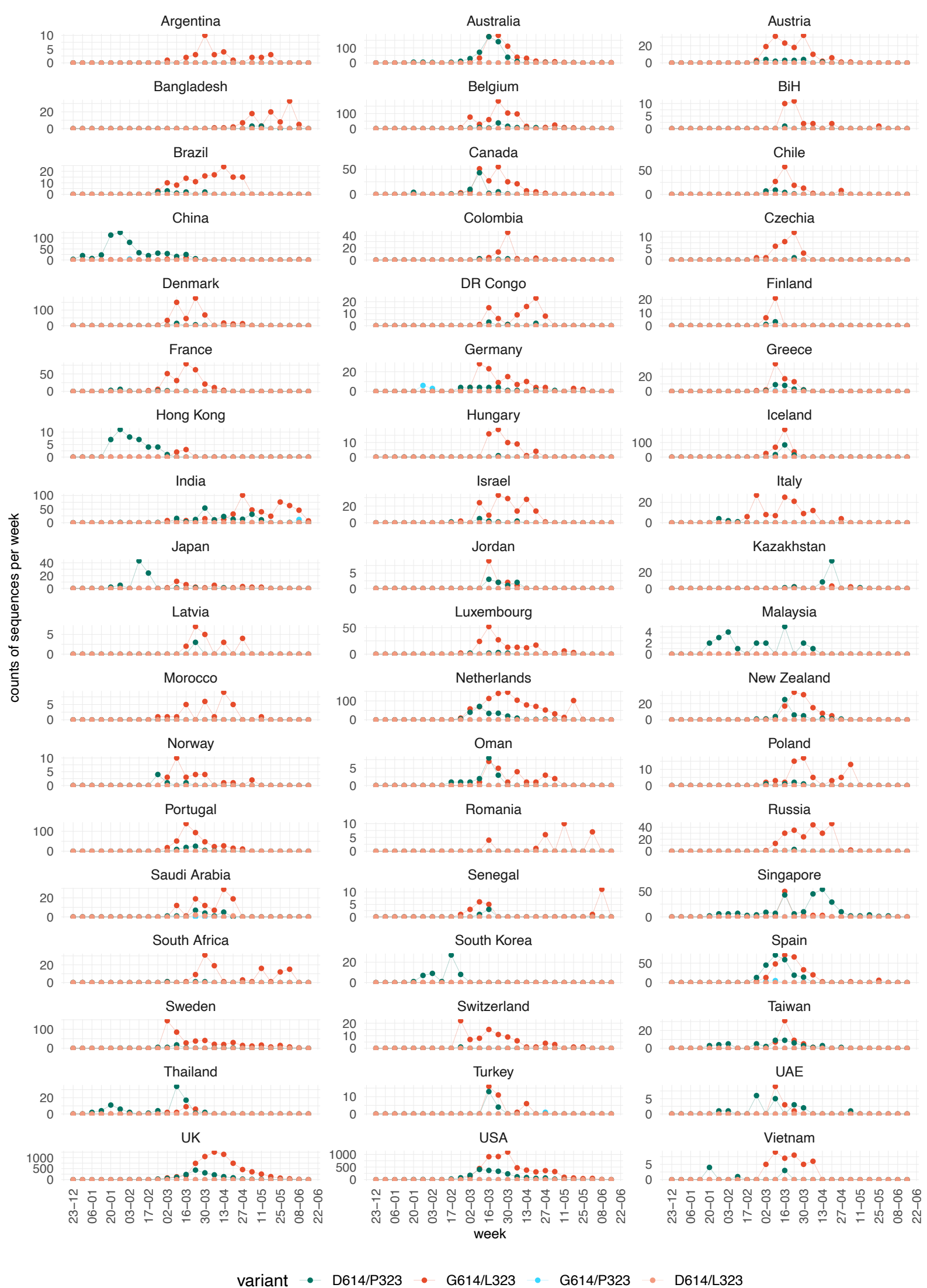

Supplement: Supplementary file 5 — Supplementary Figure S4. [file 41598_2021_91662_MOESM5_ESM.pdf]
