# Supplementary material for: Concurrent mutations in RNA-dependent RNA polymerase and spike protein emerged as the epidemiologically most successful SARS-CoV-2 variant
Source: Sci Rep. 2021 Jul 1;11:13705. doi: 10.1038/s41598-021-91662-w (PMC8249556; doi:10.1038/s41598-021-91662-w)
Supplement: Supplementary file 6 — Supplementary Table S1. [file 41598_2021_91662_MOESM6_ESM.docx]

strain gisaid_epi_isl date originating_lab submitting_lab authors

Wuhan/IPBCAMS-WH-01/2019 EPI_ISL_402123 2019-12-24 Institute of Pathogen Biology, Chinese Academy of Medical Sciences & Peking Union Medical College Institute of Pathogen Biology, Chinese Academy of Medical Sciences & Peking Union Medical College Lili Ren et al

Wuhan-Hu-1/2019 EPI_ISL_402125 2019-12-26 unknown National Institute for Communicable Disease Control and Prevention (ICDC) Chinese Center for Disease Control and Prevention (China CDC) Zhang et al

Wuhan/WH01/2019 EPI_ISL_406798 2019-12-26 General Hospital of Central Theater Command of People's Liberation Army of China BGI & Institute of Microbiology, Chinese Academy of Sciences & Shandong First Medical University & Shandong Academy of Medical Sciences & General Hospital of Central Theater Command of People's Liberation Army of China Weijun Chen et al

Wuhan/HBCDC-HB-01/2019 EPI_ISL_402132 2019-12-30 Wuhan Jinyintan Hospital Hubei Provincial Center for Disease Control and Prevention Bin Fang et al

Wuhan/HBCDC-HB-02/2019 EPI_ISL_412898 2019-12-30 Wuhan Jinyintan Hospital Hubei Provincial Center for Disease Control and Prevention Bin Fang et al

Wuhan/HBCDC-HB-03/2019 EPI_ISL_412899 2019-12-30 Wuhan Jinyintan Hospital Hubei Provincial Center for Disease Control and Prevention Bin Fang et al

Wuhan/IPBCAMS-WH-02/2019 EPI_ISL_403931 2019-12-30 Institute of Pathogen Biology, Chinese Academy of Medical Sciences & Peking Union Medical College Institute of Pathogen Biology, Chinese Academy of Medical Sciences & Peking Union Medical College Lili Ren et al

Wuhan/IPBCAMS-WH-03/2019 EPI_ISL_403930 2019-12-30 Institute of Pathogen Biology, Chinese Academy of Medical Sciences & Peking Union Medical College Institute of Pathogen Biology, Chinese Academy of Medical Sciences & Peking Union Medical College Lili Ren et al

Wuhan/IPBCAMS-WH-04/2019 EPI_ISL_403929 2019-12-30 Institute of Pathogen Biology, Chinese Academy of Medical Sciences & Peking Union Medical College Institute of Pathogen Biology, Chinese Academy of Medical Sciences & Peking Union Medical College Lili Ren et al

Wuhan/IVDC-HB-01/2019 EPI_ISL_402119 2019-12-30 National Institute for Viral Disease Control and Prevention, China CDC National Institute for Viral Disease Control and Prevention, China CDC Wenjie Tan et al

Wuhan/IVDC-HB-05/2019 EPI_ISL_402121 2019-12-30 National Institute for Viral Disease Control and Prevention, China CDC National Institute for Viral Disease Control and Prevention, China CDC Wenjie Tan et al

Wuhan/IVDC-HB-GX02/2019 EPI_ISL_434534 2019-12-30 National Institute for Viral Disease Control and Prevention, China CDC National Institute for Viral Disease Control and Prevention, China CDC, Yunnan Provincial CDC Wenjie Tan et al

Wuhan/WIV02/2019 EPI_ISL_402127 2019-12-30 Wuhan Jinyintan Hospital Wuhan Institute of Virology, Chinese Academy of Sciences Peng Zhou et al

Wuhan/WIV04/2019 EPI_ISL_402124 2019-12-30 Wuhan Jinyintan Hospital Wuhan Institute of Virology, Chinese Academy of Sciences Peng Zhou et al

Wuhan/WIV05/2019 EPI_ISL_402128 2019-12-30 Wuhan Jinyintan Hospital Wuhan Institute of Virology, Chinese Academy of Sciences Peng Zhou et al

Wuhan/WIV06/2019 EPI_ISL_402129 2019-12-30 Wuhan Jinyintan Hospital Wuhan Institute of Virology, Chinese Academy of Sciences Peng Zhou et al

Wuhan/WIV07/2019 EPI_ISL_402130 2019-12-30 Wuhan Jinyintan Hospital Wuhan Institute of Virology, Chinese Academy of Sciences Peng Zhou et al

Wuhan/IPBCAMS-WH-05/2020 EPI_ISL_403928 2020-01-01 Institute of Pathogen Biology, Chinese Academy of Medical Sciences & Peking Union Medical College Institute of Pathogen Biology, Chinese Academy of Medical Sciences & Peking Union Medical College Lili Ren et al

Wuhan/IVDC-HB-04/2020 EPI_ISL_402120 2020-01-01 National Institute for Viral Disease Control and Prevention, China CDC National Institute for Viral Disease Control and Prevention, China CDC Wenjie Tan et al

Wuhan/WH03/2020 EPI_ISL_406800 2020-01-01 General Hospital of Central Theater Command of People's Liberation Army of China BGI & Institute of Microbiology, Chinese Academy of Sciences & Shandong First Medical University & Shandong Academy of Medical Sciences & General Hospital of Central Theater Command of People's Liberation Army of China Weijun Chen et al

Wuhan/WHU01/2020 EPI_ISL_406716 2020-01-02 unknown State Key Laboratory of Virology, Wuhan University Chen et al

Wuhan/WHU02/2020 EPI_ISL_406717 2020-01-02 unknown State Key Laboratory of Virology, Wuhan University Chen et al

Wuhan/WH04/2020 EPI_ISL_406801 2020-01-05 General Hospital of Central Theater Command of People's Liberation Army of China BGI & Institute of Microbiology, Chinese Academy of Sciences & Shandong First Medical University & Shandong Academy of Medical Sciences & General Hospital of Central Theater Command of People's Liberation Army of China Weijun Chen et al

China/WH-09/2020 EPI_ISL_411957 2020-01-08 unknown Key Laboratory of Human Diseases, Comparative Medicine, Institute of Laboratory Animal Science Linlin et al

Jingzhou/HBCDC-HB-01/2020 EPI_ISL_412459 2020-01-08 Jingzhou Center for Disease Control and Prevention Hubei Provincial Center for Disease Control and Prevention Bin Fang et al

Thailand/61/2020 EPI_ISL_403962 2020-01-08 Bamrasnaradura Hospital 1. Department of Medical Sciences, Ministry of Public Health, Thailand 2. Thai Red Cross Emerging Infectious Diseases - Health Science Centre 3. Department of Disease Control, Ministry of Public Health, Thailand Pilailuk et al

Thailand/SI200040-NT/2020 EPI_ISL_437623 2020-01-08 unknown Faculty of Medicine Rodpan et al

Shenzhen/HKU-SZ-002/2020 EPI_ISL_406030 2020-01-10 The University of Hong Kong - Shenzhen Hospital Li Ka Shing Faculty of Medicine, The University of Hong Kong Chan et al

Jiangxi/IVDC-JX-002/2020 EPI_ISL_408486 2020-01-11 National Institute for Viral Disease Control and Prevention, China CDC National Institute for Viral Disease Control & Prevention, CCDC Wenjie Tan et al

Pingxiang/JX5/2020 EPI_ISL_421252 2020-01-11 Jiangxi Province Center for Disease Control and Prevention Jiangxi Province Center for Disease Control and Prevention JianXiong Li et al

Shenzhen/HKU-SZ-005/2020 EPI_ISL_405839 2020-01-11 The University of Hong Kong - Shenzhen Hospital Li Ka Shing Faculty of Medicine, The University of Hong Kong Chan et al

Nepal/61/2020 EPI_ISL_410301 2020-01-13 National Influenza Centre, National Public Health Laboratory, Kathmandu, Nepal The University of Hong Kong Ranjit Sah et al

Shenzhen/SZTH-001/2020 EPI_ISL_406592 2020-01-13 Shenzhen Third People's Hospital Shenzhen Key Laboratory of Pathogen and Immunity, National Clinical Research Center for Infectious Disease,Shenzhen Third People's Hospital Yang Yang et al

Shenzhen/SZTH-002/2020 EPI_ISL_406593 2020-01-13 Shenzhen Key Laboratory of Pathogen and Immunity, National Clinical Research Center for Infectious Disease, Shenzhen Third People's Hospital Shenzhen Key Laboratory of Pathogen and Immunity, National Clinical Research Center for Infectious Disease, Shenzhen Third People's Hospital Yang Yang et al

Thailand/74/2020 EPI_ISL_403963 2020-01-13 Bamrasnaradura Hospital 1. Department of Medical Sciences, Ministry of Public Health, Thailand 2. Thai Red Cross Emerging Infectious Diseases - Health Science Centre 3. Department of Disease Control, Ministry of Public Health, Thailand Pilailuk et al

Thailand/Nonthaburi_59/2020 EPI_ISL_447909 2020-01-13 n/a National Institute of Health. Department of medical Sciences, Ministry of Public Health, Thailand Pilailuk et al

Guangdong/20SF012/2020 EPI_ISL_403932 2020-01-14 Guangdong Provincial Center for Diseases Control and Prevention; Guangdong Provincial Public Health Department of Microbiology, Guangdong Provincial Center for Diseases Control and Prevention Min Kang et al

Guangdong/20SF013/2020 EPI_ISL_403933 2020-01-15 Guangdong Provincial Center for Diseases Control and Prevention; Guangdong Provincial Public Health Department of Microbiology, Guangdong Provincial Center for Diseases Control and Prevention Min Kang et al

Guangdong/20SF014/2020 EPI_ISL_403934 2020-01-15 Guangdong Provincial Center for Diseases Control and Prevention; Guangdong Provincial Public Health Department of Microbiology, Guangdong Provincial Center for Diseases Control and Prevention Min Kang et al

Guangdong/20SF025/2020 EPI_ISL_403935 2020-01-15 Guangdong Provincial Center for Diseases Control and Prevention; Guangdong Provincial Public Health Department of Microbiology, Guangdong Provincial Center for Diseases Control and Prevention Min Kang et al

Sichuan/IVDC-SC-001/2020 EPI_ISL_408484 2020-01-15 National Institute for Viral Disease Control and Prevention, China CDC National Institute for Viral Disease Control & Prevention, CCDC Wenjie Tan et al

Thailand/Nonthaburi_68/2020 EPI_ISL_447910 2020-01-15 n/a National Institute of Health. Department of medical Sciences, Ministry of Public Health, Thailand Pilailuk et al

Shenzhen/SZTH-003/2020 EPI_ISL_406594 2020-01-16 Shenzhen Key Laboratory of Pathogen and Immunity, National Clinical Research Center for Infectious Disease, Shenzhen Third People's Hospital Shenzhen Key Laboratory of Pathogen and Immunity, National Clinical Research Center for Infectious Disease, Shenzhen Third People's Hospital Yang Yang et al

Shenzhen/SZTH-004/2020 EPI_ISL_406595 2020-01-16 Shenzhen Key Laboratory of Pathogen and Immunity, National Clinical Research Center for Infectious Disease, Shenzhen Third People's Hospital Shenzhen Key Laboratory of Pathogen and Immunity, National Clinical Research Center for Infectious Disease, Shenzhen Third People's Hospital Yang Yang et al

Thailand/NIH-59/2020 EPI_ISL_434693 2020-01-16 Bamrasnaradura hospital National Institute of Health. Department of medical Sciences, Ministry of Public Health, Thailand Pilailuk et al

Zhejiang/WZ-01/2020 EPI_ISL_404227 2020-01-16 Zhejiang Provincial Center for Disease Control and Prevention Department of Microbiology, Zhejiang Provincial Center for Disease Control and Prevention Yin Chen et al

Guangdong/20SF028/2020 EPI_ISL_403936 2020-01-17 Guangdong Provincial Center for Diseases Control and Prevention; Guangdong Provincial Public Health Department of Microbiology, Guangdong Provincial Center for Diseases Control and Prevention Min Kang et al

Wuhan/HBCDC-HB-02/2020 EPI_ISL_412978 2020-01-17 The Central Hospital Of Wuhan Hubei Provincial Center for Disease Control and Prevention Bin Fang et al

Yunnan/IVDC-YN-003/2020 EPI_ISL_408480 2020-01-17 National Institute for Viral Disease Control and Prevention, China CDC National Institute for Viral Disease Control & Prevention, CCDC Wenjie Tan et al

Zhejiang/WZ-02/2020 EPI_ISL_404228 2020-01-17 Zhejiang Provincial Center for Disease Control and Prevention Department of Microbiology, Zhejiang Provincial Center for Disease Control and Prevention Yanjun Zhang et al

Beijing/IVDC-BJ-005/2020 EPI_ISL_408485 2020-01-18 National Institute for Viral Disease Control and Prevention, China CDC National Institute for Viral Disease Control & Prevention, CCDC Wenjie Tan et al

Chongqing/IVDC-CQ-001/2020 EPI_ISL_408481 2020-01-18 National Institute for Viral Disease Control and Prevention, China CDC National Institute for Viral Disease Control & Prevention, CCDC Wenjie Tan et al

Guangdong/20SF040/2020 EPI_ISL_403937 2020-01-18 Guangdong Provincial Center for Diseases Control and Prevention; Guangdong Provincial Public Health Department of Microbiology, Guangdong Provincial Center for Diseases Control and Prevention Min Kang et al

Wuhan/HBCDC-HB-03/2020 EPI_ISL_412979 2020-01-18 Union Hospital of Tongji Medical College, Huazhong University of Science and Technology Hubei Provincial Center for Disease Control and Prevention Bin Fang et al

Wuhan/HBCDC-HB-04/2020 EPI_ISL_412980 2020-01-18 Union Hospital of Tongji Medical College, Huazhong University of Science and Technology Hubei Provincial Center for Disease Control and Prevention Bin Fang et al

Wuhan/HBCDC-HB-05/2020 EPI_ISL_412981 2020-01-18 CR&WISCO GENERAL HOSPITAL Hubei Provincial Center for Disease Control and Prevention Bin Fang et al

Hangzhou/HZCDC0001/2020 EPI_ISL_407313 2020-01-19 Hangzhou Center for Disease Control and Prevention Hangzhou Center for Disease Control and Prevention Jun Li et al

Jiangsu/IVDC-JS-001/2020 EPI_ISL_408488 2020-01-19 National Institute for Viral Disease Control and Prevention, China CDC National Institute for Viral Disease Control & Prevention, CCDC Wenjie Tan et al

Shandong/IVDC-SD-001/2020 EPI_ISL_408482 2020-01-19 National Institute for Viral Disease Control and Prevention, China CDC National Institute for Viral Disease Control & Prevention, CCDC Wenjie Tan et al

USA/WA1/2020 EPI_ISL_404895 2020-01-19 Providence Regional Medical Center Division of Viral Diseases, Centers for Disease Control and Prevention Queen et al

Guangdong/20SF123/2020 EPI_ISL_428449 2020-01-20 Guangdong Provincial Center for Diseases Control and Prevention;Guangdong Provincial Institute of Public Health School of Public Health, The University of Hong Kong Bosheng Li et al

Hangzhou/HZ-1/2020 EPI_ISL_406970 2020-01-20 Hangzhou Center for Disease and Control Microbiology Lab Hangzhou Center for Disease and Control Microbiology Lab Yu Hua et al

Hangzhou/HZCDC0012/2020 EPI_ISL_421236 2020-01-20 Hangzhou Center for Diseases Control and Prevention Hangzhou Center for Diseases Control and Prevention Jun Li et al

Hangzhou/HZCDC0013/2020 EPI_ISL_421235 2020-01-20 Hangzhou Center for Diseases Control and Prevention Hangzhou Center for Diseases Control and Prevention Jun Li et al

Chongqing/YC01/2020 EPI_ISL_408478 2020-01-21 Yongchuan District Center for Disease Control and Prevention Chongqing Municipal Center for Disease Control and Prevention Ye Sheng et al

Fujian/8/2020 EPI_ISL_411060 2020-01-21 Fujian Center for Disease Control and Prevention Fujian Center for Disease Control and Prevention Chen Wei et al

Guangdong/20SF115/2020 EPI_ISL_428443 2020-01-21 Guangdong Provincial Center for Diseases Control and Prevention;Guangdong Provincial Institute of Public Health School of Public Health, The University of Hong Kong Bosheng Li et al

Guangdong/20SF117/2020 EPI_ISL_428447 2020-01-21 Guangdong Provincial Center for Diseases Control and Prevention;Guangdong Provincial Institute of Public Health School of Public Health, The University of Hong Kong Bosheng Li et al

Guangdong/20SF118/2020 EPI_ISL_428448 2020-01-21 Guangdong Provincial Center for Diseases Control and Prevention;Guangdong Provincial Institute of Public Health School of Public Health, The University of Hong Kong Bosheng Li et al

Hangzhou/HZ48/2020 EPI_ISL_418441 2020-01-21 Hangzhou Center for Disease Control and Prevention Inspection Center of Hangzhou Center for Disease Control and Prevention Yu hua et al

Hangzhou/HZ49/2020 EPI_ISL_418442 2020-01-21 Hangzhou Center for Disease Control and Prevention Inspection Center of Hangzhou Center for Disease Control and Prevention Yu hua et al

Hangzhou/HZ79/2020 EPI_ISL_418504 2020-01-21 Hangzhou Center for Disease Control and Prevention Inspection Center of Hangzhou Center for Disease Control and Prevention Yu hua et al

Hangzhou/HZ90/2020 EPI_ISL_418506 2020-01-21 Hangzhou Center for Disease Control and Prevention Inspection Center of Hanghzou Center for Disease Control and Prevention Yu hua et al

Hangzhou/HZ91/2020 EPI_ISL_418507 2020-01-21 Hangzhou Center for Disease Control and Prevention Inspection Center of Hangzhou Center for Disease Control and Prevention Yu hua et al

Hangzhou/HZCDC0025/2020 EPI_ISL_421234 2020-01-21 Hangzhou Center for Diseases Control and Prevention Hangzhou Center for Diseases Control and Prevention Jun Li et al

Hangzhou/HZCDC0048/2020 EPI_ISL_421233 2020-01-21 Hangzhou Center for Diseases Control and Prevention Hangzhou Center for Diseases Control and Prevention Jun Li et al

Hangzhou/HZCDC0048L/2020 EPI_ISL_421232 2020-01-21 Hangzhou Center for Diseases Control and Prevention Hangzhou Center for Diseases Control and Prevention Jun Li et al

Hangzhou/HZCDC0049L/2020 EPI_ISL_421231 2020-01-21 Hangzhou Center for Diseases Control and Prevention Hangzhou Center for Diseases Control and Prevention Jun Li et al

Hangzhou/HZCDC0090L/2020 EPI_ISL_421229 2020-01-21 Hangzhou Center for Diseases Control and Prevention Hangzhou Center for Diseases Control and Prevention Jun Li et al

Hangzhou/HZCDC0091/2020 EPI_ISL_421228 2020-01-21 Hangzhou Center for Diseases Control and Prevention Hangzhou Center for Diseases Control and Prevention Jun Li et al

Hangzhou/HZCDC0091L/2020 EPI_ISL_421227 2020-01-21 Hangzhou Center for Diseases Control and Prevention Hangzhou Center for Diseases Control and Prevention Jun Li et al

HongKong/VB20017970/2020 EPI_ISL_417064 2020-01-21 Prince of Wales Hospital Hong Kong Department of Health Alan K.L. Tsang et al

Nanchang/JX14/2020 EPI_ISL_421243 2020-01-21 Jiangxi Province Center for Disease Control and Prevention Jiangxi Province Center for Disease Control and Prevention JianXiong Li et al

Australia/NSW02/2020 EPI_ISL_408976 2020-01-22 Centre for Infectious Diseases and Microbiology Laboratory Services NSW Health Pathology - Institute of Clinical Pathology and Medical Research; Westmead Hospital; University of Sydney Rockett R et al

Foshan/20SF207/2020 EPI_ISL_406534 2020-01-22 Guangdong Provincial Center for Diseases Control and Prevention; Guangdong Provincial Public Health Guangdong Provincial Center for Diseases Control and Prevention Min Kang et al

Foshan/20SF210/2020 EPI_ISL_406535 2020-01-22 Guangdong Provincial Center for Diseases Control and Prevention; Guangdong Provincial Public Health Guangdong Provincial Center for Diseases Control and Prevention Min Kang et al

Foshan/20SF211/2020 EPI_ISL_406536 2020-01-22 Guangdong Provincial Center for Diseases Control and Prevention; Guangdong Provincial Public Health Guangdong Provincial Center for Diseases Control and Prevention Min Kang et al

Fujian/13/2020 EPI_ISL_411066 2020-01-22 Fujian Center for Disease Control and Prevention Fujian Center for Disease Control and Prevention Chen Wei et al

Guangdong/20SF174/2020 EPI_ISL_406531 2020-01-22 Guangdong Provincial Center for Diseases Control and Prevention; Guangdong Provinical Public Health Guangdong Provincial Center for Disease Control and Prevention Min Kang et al

Guangdong/20SF198/2020 EPI_ISL_428454 2020-01-22 Guangdong Provincial Center for Diseases Control and Prevention;Guangdong Provincial Institute of Public Health School of Public Health, The University of Hong Kong Bosheng Li et al

Guangzhou/20SF206/2020 EPI_ISL_406533 2020-01-22 Guangdong Provincial Center for Diseases Control and Prevention; Guangdong Provinical Public Health Guangdong Provincial Center for Diseases Control and Prevention Min Kang et al

Hangzhou/HZ60/2020 EPI_ISL_418502 2020-01-22 Hangzhou Center for Disease Control and Prevention Inspection Center of Hangzhou Center for Disease Control and Prevention Yu hua et al

Hangzhou/HZ62/2020 EPI_ISL_418503 2020-01-22 Hangzhou Center for Disease Control and Prevention Inspection Center of Hangzhou Center for Disease Control and Prevention Yu hua et al

Hangzhou/HZCDC0119/2020 EPI_ISL_421226 2020-01-22 Hangzhou Center for Diseases Control and Prevention Hangzhou Center for Diseases Control and Prevention Jun Li et al

Hangzhou/HZCDC0135/2020 EPI_ISL_421225 2020-01-22 Hangzhou Center for Diseases Control and Prevention Hangzhou Center for Diseases Control and Prevention Jun Li et al

Hangzhou/ZJU-05/2020 EPI_ISL_415711 2020-01-22 State Key Laboratory for Diagnosis and Treatment of Infectious Diseases, National Clinical Research Center for Infectious Diseases, First Affiliated Hospital, Zhejiang University School of Medicine, Hangzhou, China. 310003 State Key Laboratory for Diagnosis and Treatment of Infectious Diseases, National Clinical Research Center for Infectious Diseases, First Affiliated Hospital, Zhejiang University School of Medicine, Hangzhou, China. 310003 Hangping Yao et al

HongKong/HKU-001a/2020 EPI_ISL_434571 2020-01-22 unknown Microbiology Chan et al

Jiujiang/JX22/2020 EPI_ISL_421237 2020-01-22 Jiangxi Province Center for Disease Control and Prevention Jiangxi Province Center for Disease Control and Prevention JianXiong Li et al

Shangrao/JX29/2020 EPI_ISL_421244 2020-01-22 Jiangxi Province Center for Disease Control and Prevention Jiangxi Province Center for Disease Control and Prevention JianXiong Li et al

Thailand/NIH-162/2020 EPI_ISL_434694 2020-01-22 Bamrasnaradura hospital National Institute of Health. Department of medical Sciences, Ministry of Public Health, Thailand Pilailuk et al

USA/AZ1/2020 EPI_ISL_406223 2020-01-22 Arizona Department of Health Services Pathogen Discovery, Respiratory Viruses Branch, Division of Viral Diseases, Centers for Disease Control and Prevention Ying Tao et al

USA/CA2/2020 EPI_ISL_406036 2020-01-22 California Department of Public Health Pathogen Discovery, Respiratory Viruses Branch, Division of Viral Diseases, Centers for Disease Control and Prevention Anna Uehara et al

Vietnam/19-01S/2020 EPI_ISL_418269 2020-01-22 unknown Microbiology and Immunology department Cao et al

Vietnam/19-02S/2020 EPI_ISL_418267 2020-01-22 unknown Microbiology and Immunology department Nguyen et al

Wuhan/WHUH001/2020 EPI_ISL_449476 2020-01-22 unknown Department of Respiratory and Critical Care Wang et al

Wuhan/WHUH002/2020 EPI_ISL_449477 2020-01-22 unknown Department of Respiratory and Critical Care Wang et al

Wuhan/WHUH003/2020 EPI_ISL_449478 2020-01-22 unknown Department of Respiratory and Critical Care Wang et al

Wuhan/WHUH004/2020 EPI_ISL_449479 2020-01-22 unknown Department of Respiratory and Critical Care Wang et al

Wuhan/WHUH005/2020 EPI_ISL_449480 2020-01-22 unknown Department of Respiratory and Critical Care Wang et al

Wuhan/WHUH006/2020 EPI_ISL_449481 2020-01-22 unknown Department of Respiratory and Critical Care Wang et al

Wuhan/WHUH007/2020 EPI_ISL_449482 2020-01-22 unknown Department of Respiratory and Critical Care Wang et al

Wuhan/WHUH008/2020 EPI_ISL_449483 2020-01-22 unknown Department of Respiratory and Critical Care Wang et al

Wuhan/WHUH011/2020 EPI_ISL_449484 2020-01-22 unknown Department of Respiratory and Critical Care Wang et al

Wuhan/WHUH012/2020 EPI_ISL_449485 2020-01-22 unknown Department of Respiratory and Critical Care Wang et al

Wuhan/WHUH020/2020 EPI_ISL_449486 2020-01-22 unknown Department of Respiratory and Critical Care Wang et al

Wuhan/WHUH021/2020 EPI_ISL_449487 2020-01-22 unknown Department of Respiratory and Critical Care Wang et al

Canada/ON-VIDO-01/2020 EPI_ISL_413015 2020-01-23 Public Health Ontario Laboratory National Microbiology Laboratory Shari Tyson et al

Chongqing/ZX01/2020 EPI_ISL_408479 2020-01-23 Zhongxian Center for Disease Control and Prevention Chongqing Municipal Center for Disease Control and Prevention Ye Sheng et al

France/IDF0372-isl/2020 EPI_ISL_410720 2020-01-23 Department of Infectious and Tropical Diseases, Bichat Claude Bernard Hospital, Paris National Reference Center for Viruses of Respiratory Infections, Institut Pasteur, Paris M√©lanie Albert et al

France/IDF0372/2020 EPI_ISL_406596 2020-01-23 Department of Infectious and Tropical Diseases, Bichat Claude Bernard Hospital, Paris National Reference Center for Viruses of Respiratory Infections, Institut Pasteur, Paris M√©lanie Albert et al

France/IDF0373/2020 EPI_ISL_406597 2020-01-23 Department of Infectious and Tropical Diseases, Bichat Claude Bernard Hospital, Paris National Reference Center for Viruses of Respiratory Infections, Institut Pasteur, Paris M√©lanie Albert et al

Fujian/3520022Y/2020 EPI_ISL_431785 2020-01-23 Fujian Center for Disease Control and Prevention Fujian Center for Disease Control and Prevention Lin Qi et al

Guangdong/20SF190/2020 EPI_ISL_428452 2020-01-23 Guangdong Provincial Center for Diseases Control and Prevention;Guangdong Provincial Institute of Public Health School of Public Health, The University of Hong Kong Bosheng Li et al

Guangdong/20SF201/2020 EPI_ISL_406538 2020-01-23 Guangdong Provincial Center for Diseases Control and Prevention;Guangdong Provincial Institute of Public Health Guangdong Provincial Center for Diseases Control and Prevention Min Kang et al

Guangdong/20SF273/2020 EPI_ISL_428460 2020-01-23 Guangdong Provincial Center for Diseases Control and Prevention;Guangdong Provincial Institute of Public Health School of Public Health, The University of Hong Kong Bosheng Li et al

Hangzhou/HZ162/2020 EPI_ISL_418508 2020-01-23 Hangzhou Center for Disease Control and Prevention Inspection Center of Hangzhou Center for Disease Control and Prevention Yu hua et al

Hangzhou/HZ178/2020 EPI_ISL_418509 2020-01-23 Hangzhou Center for Disease Control and Prevention Inspection Center of Hangzhou Center for Disease Control and Prevention Yu hua et al

Hangzhou/HZ185/2020 EPI_ISL_418510 2020-01-23 Hangzhou Center for Disease Control and Prevention Insepction Center of Hangzhou Center for Disease Control and Prevention Yu hua et al

Hangzhou/HZCDC0162/2020 EPI_ISL_421224 2020-01-23 Hangzhou Center for Diseases Control and Prevention Hangzhou Center for Diseases Control and Prevention Jun Li et al

HongKong/HKU-902a/2020 EPI_ISL_434563 2020-01-23 unknown Microbiology To et al

Japan/OS-20-07-1/2020 EPI_ISL_410532 2020-01-23 Dept. of Pathology, National Institute of Infectious Diseases Pathogen Genomics Center, National Institute of Infectious Diseases Tsuyoshi Sekizuka et al

Jiangsu/JS01/2020 EPI_ISL_411950 2020-01-23 NHC Key laboratory of Enteric Pathogenic Microbiology, Institute of Pathogenic Microbiology Jiangsu Provincial Center for Disease Control & Prevention Lunbiao Cui et al

Nanchang/JX39/2020 EPI_ISL_421248 2020-01-23 Jiangxi Province Center for Disease Control and Prevention Jiangxi Province Center for Disease Control and Prevention JianXiong Li et al

Shandong/LY003/2020 EPI_ISL_414936 2020-01-23 Shandong Provincial Center for Disease Control and Prevention Beijing Institute of Microbiology and Epidemiology Xiao-Lin Jiang et al

Singapore/1/2020 EPI_ISL_406973 2020-01-23 Singapore General Hospital National Public Health Laboratory Mak et al

Taiwan/2/2020 EPI_ISL_406031 2020-01-23 Centers for Disease Control, R.O.C. (Taiwan) Centers for Disease Control, R.O.C. (Taiwan) Ji-Rong Yang et al

Thailand/SI200383-NT/2020 EPI_ISL_437622 2020-01-23 unknown Faculty of Medicine Rodpan et al

Thailand/SI203285-NT/2020 EPI_ISL_437612 2020-01-23 unknown Faculty of Medicine Rodpan et al

USA/CA1/2020 EPI_ISL_406034 2020-01-23 California Department of Public Health Pathogen Discovery, Respiratory Viruses Branch, Division of Viral Diseases, Centers for Disease Control and Prevention Anna Uehara et al

Australia/NSW01/2020 EPI_ISL_407893 2020-01-24 Centre for Infectious Diseases and Microbiology Laboratory Services NSW Health Pathology - Institute of Clinical Pathology and Medical Research; Westmead Hospital; University of Sydney Eden J-S et al

Australia/NSW04/2020 EPI_ISL_417030 2020-01-24 Centre for Infectious Diseases and Microbiology Laboratory Services NSW Health Pathology - Institute of Clinical Pathology and Medical Research; Westmead Hospital; University of Sydney Eden J-S et al

Beijing/BJ53/2020 EPI_ISL_430734 2020-01-24 Chinese PLA Institute for Disease Control and Prevention Chinese PLA Institute for Disease Control and Prevention Peng Li et al

Beijing/BJ54/2020 EPI_ISL_430735 2020-01-24 Chinese PLA Institute for Disease Control and Prevention Chinese PLA Institute for Disease Control and Prevention Peng Li et al

Beijing/Wuhan_IME-BJ01/2020 EPI_ISL_424355 2020-01-24 unknown Beijing Institute of Microbiology and Epidemiology Fan et al

Guangdong/20SF616/2020 EPI_ISL_428465 2020-01-24 Guangdong Provincial Center for Diseases Control and Prevention;Guangdong Provincial Institute of Public Health School of Public Health, The University of Hong Kong Bosheng Li et al

Hangzhou/HZ477/2020 EPI_ISL_418511 2020-01-24 Hangzhou Center for Disease Control and Prevention Inspection Center of Hangzhou Center for Disease Control and Prevention Yu hua et al

Hangzhou/ZJU-04/2020 EPI_ISL_416046 2020-01-24 State Key Laboratory for Diagnosis and Treatment of Infectious Diseases, National Clinical Research Center for Infectious Diseases, First Affiliated Hospital, Zhejiang University School of Medicine, Hangzhou, China 310003 State Key Laboratory for Diagnosis and Treatment of Infectious Diseases, National Clinical Research Center for Infectious Diseases, First Affiliated Hospital, Zhejiang University School of Medicine, Hangzhou, China 310003 Hangping Yao et al

HongKong/HKU-902b/2020 EPI_ISL_434564 2020-01-24 unknown unknown To et al

HongKong/HKU-903a/2020 EPI_ISL_434565 2020-01-24 unknown Microbiology To et al

Jiangsu/JS02/2020 EPI_ISL_411952 2020-01-24 NHC Key laboratory of Enteric Pathogenic Microbiology, Institute of Pathogenic Microbiology Jiangsu Provincial Center for Disease Control & Prevention Kangchen Zhao et al

Jiangsu/JS03/2020 EPI_ISL_411953 2020-01-24 NHC Key laboratory of Enteric Pathogenic Microbiology, Institute of Pathogenic Microbiology Jiangsu Provincial Center for Disease Control & Prevention Kangchen Zhao et al

Malaysia/MKAK-CL-2020-5045/2020 EPI_ISL_416829 2020-01-24 National Public Health Laboratory Malaysia Genome Institute Mohd Noor Mat Isa et al

Malaysia/MKAK-CL-2020-5047/2020 EPI_ISL_416866 2020-01-24 National Public Health Laboratory Malaysia Genome Institute Mohd Noor Mat Isa et al

Shandong/LY005/2020 EPI_ISL_414938 2020-01-24 Shandong Provincial Center for Disease Control and Prevention Beijing Institute of Microbiology and Epidemiology Xiao-Lin Jiang et al

Taiwan/3/2020 EPI_ISL_411926 2020-01-24 Taiwan Centers for Disease Control Taiwan Centers for Disease Control Ji-Rong Yang et al

Thailand/Nonthaburi_193/2020 EPI_ISL_447911 2020-01-24 n/a National Institute of Health. Department of medical Sciences, Ministry of Public Health, Thailand Pilailuk et al

Vietnam/38142/2020 EPI_ISL_416427 2020-01-24 National Influenza Center, National Institute of Hygiene and Epidemiology (NIHE) National Influenza Center, National Institute of Hygiene and Epidemiology (NIHE) Le Quynh Mai et al

Vietnam/VR03-38142/2020 EPI_ISL_408668 2020-01-24 National Influenza Center - National Institute of Hygiene and Epidemiology (NIHE) National Influenza Center - National Institute of Hygiene and Epidemiology (NIHE) Ung Thi Hong Trang et al

Zhejiang/HZ103/2020 EPI_ISL_422425 2020-01-24 Zhejiang Provincial Center for Disease Control and Prevention Zhejiang Provincial Center for Disease Control and Prevention Yanjun Zhang et al

Australia/NSW03/2020 EPI_ISL_408977 2020-01-25 Serology, Virology and OTDS Laboratories (SAViD), NSW Health Pathology Randwick NSW Health Pathology - Institute of Clinical Pathology and Medical Research; Centre for Infectious Diseases and Microbiology Laboratory Services; Westmead Hospital; University of Sydney Eden J-S et al

Australia/VIC03/2020 EPI_ISL_416411 2020-01-25 Victorian Infectious Diseases Reference Laboratory (VIDRL) Victorian Infectious Diseases Reference Laboratory and Microbiological Diagnostic Unit Public Health Laboratory, Doherty Institute Caly L. et al

Beijing/Wuhan_IME-BJ02/2020 EPI_ISL_424356 2020-01-25 unknown Beijing Institute of Microbiology and Epidemiology Fan et al

Canada/ON-PHL2445/2020 EPI_ISL_413014 2020-01-25 Public Health Ontario Laboratory Ontario Agency for Health Protection and Promotion (OAHPP) Alireza Eshaghi et al

Canada/ON_PHL4181/2020 EPI_ISL_418327 2020-01-25 Public Health Ontario Laboratories Public Health Ontario Laboratories Alireza Eshaghi et al

Ganzhou/JX81/2020 EPI_ISL_421242 2020-01-25 Jiangxi Province Center for Disease Control and Prevention Jiangxi Province Center for Disease Control and Prevention JianXiong Li et al

Guangdong/20SF602/2020 EPI_ISL_428464 2020-01-25 Guangdong Provincial Center for Diseases Control and Prevention;Guangdong Provincial Institute of Public Health School of Public Health, The University of Hong Kong Bosheng Li et al

Hangzhou/HZ481/2020 EPI_ISL_418512 2020-01-25 Hangzhou Center for Disease Control and Prevention Inspection Center of Hangzhou Center for Disease Control and Prevention Yu hua et al

Hangzhou/HZ551/2020 EPI_ISL_418513 2020-01-25 Hangzhou Center for Disease Control and Prevention Inspection Center of Hangzhou Center for Disease Control and Prevention Yu hua et al

Hangzhou/HZ576/2020 EPI_ISL_418514 2020-01-25 Hangzhou Center for Disease Control and Prevention Inspection Center of Hangzhou Center for Disease Control and Prevention Yu hua et al

Hangzhou/HZ638/2020 EPI_ISL_418515 2020-01-25 Hangzhou Center for Disease Control and Prevention Inspection Center of Hangzhou Center for Disease Control and Prevention Yu hua et al

Hangzhou/ZJU-01/2020 EPI_ISL_415709 2020-01-25 State Key Laboratory for Diagnosis and Treatment of Infectious Diseases, National Clinical Research Center for Infectious Diseases, First Affiliated Hospital, Zhejiang University School of Medicine, Hangzhou, China. 310003 State Key Laboratory for Diagnosis and Treatment of Infectious Diseases, National Clinical Research Center for Infectious Diseases, First Affiliated Hospital, Zhejiang University School of Medicine, Hangzhou, China. 310003 Hangping Yao et al

Hangzhou/ZJU-03/2020 EPI_ISL_416044 2020-01-25 State Key Laboratory for Diagnosis and Treatment of Infectious Diseases, National Clinical Research Center for Infectious Diseases, First Affiliated Hospital, Zhejiang University School of Medicine, Hangzhou, China 310003 State Key Laboratory for Diagnosis and Treatment of Infectious Diseases, National Clinical Research Center for Infectious Diseases, First Affiliated Hospital, Zhejiang University School of Medicine, Hangzhou, China 310003 Hangping Yao et al

HongKong/HKPU6_2101/2020 EPI_ISL_417178 2020-01-25 Department of Pathology, Princess Margaret Hospital Department of Health Technology and Informatics, Faculty of Health and Social Science, The Hong Kong Polytechnic University Kenneth Siu-Sing LEUNG et al

Japan/NA-20-05-1/2020 EPI_ISL_410531 2020-01-25 Dept. of Pathology, National Institute of Infectious Diseases Pathogen Genomics Center, National Institute of Infectious Diseases Tsuyoshi Sekizuka et al

Lishui/LS111/2020 EPI_ISL_429852 2020-01-25 Centers for Disease Control and Prevention of Lishui Department of InspectionÔºåCenters for Disease Control and Prevention of Lishui Wang Xiaoguang et al

Nanchang/JX90/2020 EPI_ISL_421245 2020-01-25 Jiangxi Province Center for Disease Control and Prevention Jiangxi Province Center for Disease Control and Prevention JianXiong Li et al

Shandong/LY007/2020 EPI_ISL_414940 2020-01-25 Shandong Provincial Center for Disease Control and Prevention Beijing Institute of Microbiology and Epidemiology Xiao-Lin Jiang et al

Shanghai/SH0002/2020 EPI_ISL_416316 2020-01-25 Shanghai Public Health Clinical Center, Shanghai Medical College, Fudan University National Research Center for Translational Medicine (Shanghai), Ruijin Hospital affiliated to Shanghai Jiao Tong University School of Medicine & Shanghai Public Health Clinical Center Shengyue Wang et al

Shanghai/SH0003/2020 EPI_ISL_416317 2020-01-25 Shanghai Public Health Clinical Center, Shanghai Medical College, Fudan University National Research Center for Translational Medicine (Shanghai), Ruijin Hospital affiliated to Shanghai Jiao Tong University School of Medicine & Shanghai Public Health Clinical Center Shengyue Wang et al

Singapore/2/2020 EPI_ISL_407987 2020-01-25 Singapore General Hospital Programme in Emerging Infectious Diseases, Duke-NUS Medical School Danielle E Anderson et al

SouthKorea/KCDC03/2020 EPI_ISL_407193 2020-01-25 Korea Centers for Disease Control & Prevention (KCDC) Center for Laboratory Control of Infectious Diseases Division of Viral Diseases Korea Centers for Disease Control & Prevention (KCDC) Center for Laboratory Control of Infectious Diseases Division of Viral Diseases Jeong-Min Kim et al

Taiwan/CGMH-CGU-01/2020 EPI_ISL_411915 2020-01-25 Laboratory Medicine Department of Laboratory Medicine, Lin-Kou Chang Gung Memorial Hospital, Taoyuan, Taiwan. Kuo-Chien Tsao et al

Thailand/Bangkok_237/2020 EPI_ISL_447912 2020-01-25 n/a National Institute of Health. Department of medical Sciences, Ministry of Public Health, Thailand Pilailuk et al

Thailand/Phuket_247/2020 EPI_ISL_447914 2020-01-25 n/a National Institute of Health. Department of medical Sciences, Ministry of Public Health, Thailand Pilailuk et al

Thailand/SI200433-NT/2020 EPI_ISL_437621 2020-01-25 unknown Faculty of Medicine Rodpan et al

Thailand/SI200468-NT/2020 EPI_ISL_437620 2020-01-25 unknown Faculty of Medicine Rodpan et al

USA/WA1-A12/2020 EPI_ISL_407214 2020-01-25 WA State Department of Health Pathogen Discovery, Respiratory Viruses Branch, Division of Viral Diseases, Centers for Disease Control and Prevention Krista Queen et al

USA/WA1-F6/2020 EPI_ISL_407215 2020-01-25 Washington State Department of Health Pathogen Discovery, Respiratory Viruses Branch, Division of Viral Diseases, Centers for Disease Control and Prevention Krista Queen et al

Xinyu/JX122/2020 EPI_ISL_421249 2020-01-25 Jiangxi Province Center for Disease Control and Prevention Jiangxi Province Center for Disease Control and Prevention JianXiong Li et al

Beijing/105/2020 EPI_ISL_413518 2020-01-26 unknown Infectious Disease Control Center Li et al

Guangdong/20SF629/2020 EPI_ISL_428466 2020-01-26 Guangdong Provincial Center for Diseases Control and Prevention;Guangdong Provincial Institute of Public Health School of Public Health, The University of Hong Kong Bosheng Li et al

Guangdong/20SF632/2020 EPI_ISL_428468 2020-01-26 Guangdong Provincial Center for Diseases Control and Prevention;Guangdong Provincial Institute of Public Health School of Public Health, The University of Hong Kong Bosheng Li et al

Hangzhou/ZJU-02/2020 EPI_ISL_416042 2020-01-26 State Key Laboratory for Diagnosis and Treatment of Infectious Diseases, National Clinical Research Center for Infectious Diseases, First Affiliated Hospital, Zhejiang University School of Medicine, Hangzhou, China. 310003 State Key Laboratory for Diagnosis and Treatment of Infectious Diseases, National Clinical Research Center for Infectious Diseases, First Affiliated Hospital, Zhejiang University School of Medicine, Hangzhou, China. 310003 Hangping Yao et al

Hangzhou/ZJU-08/2020 EPI_ISL_416473 2020-01-26 State Key Laboratory for Diagnosis and Treatment of Infectious Diseases, National Clinical Research Center for Infectious Diseases, First Affiliated Hospital, Zhejiang University School of Medicine, Hangzhou, China 310003 State Key Laboratory for Diagnosis and Treatment of Infectious Diseases, National Clinical Research Center for Infectious Diseases, First Affiliated Hospital, Zhejiang University School of Medicine, Hangzhou, China 310003 Hangping Yao et al

Jian/JX129/2020 EPI_ISL_421253 2020-01-26 Jiangxi Province Center for Disease Control and Prevention Jiangxi Province Center for Disease Control and Prevention JianXiong Li et al

Shandong/LY004/2020 EPI_ISL_414937 2020-01-26 Shandong Provincial Center for Disease Control and Prevention Beijing Institute of Microbiology and Epidemiology Xiao-Lin Jiang et al

Shangrao/JX105/2020 EPI_ISL_421239 2020-01-26 Jiangxi Province Center for Disease Control and Prevention Jiangxi Province Center for Disease Control and Prevention JianXiong Li et al

Thailand/Bangkok_269/2020 EPI_ISL_447915 2020-01-26 n/a National Institute of Health. Department of medical Sciences, Ministry of Public Health, Thailand Pilailuk et al

Thailand/SI200478-NST/2020 EPI_ISL_437619 2020-01-26 unknown Faculty of Medicine Rodpan et al

Xinyu/JX124/2020 EPI_ISL_421260 2020-01-26 Jiangxi Province Center for Disease Control and Prevention Jiangxi Province Center for Disease Control and Prevention JianXiong Li et al

Beijing/Wuhan_IME-BJ05/2020 EPI_ISL_424359 2020-01-27 unknown Beijing Institute of Microbiology and Epidemiology Fan et al

Cambodia/0012/2020 EPI_ISL_411902 2020-01-27 Virology Unit, Institut Pasteur du Cambodge. Virology Unit, Institut Pasteur du Cambodge (Sequencing done by: Jessica E Manning/Jennifer A Bohl at Malaria and Vector Research Research Laboratory, National Institute of Allergy and Infectious Diseases and Vida Ahyong from Chan-Zuckerberg Biohub) Erik A Karlsson et al

Guangdong/20SF665/2020 EPI_ISL_428469 2020-01-27 Guangdong Provincial Center for Diseases Control and Prevention;Guangdong Provincial Institute of Public Health School of Public Health, The University of Hong Kong Bosheng Li et al

Guangdong/20SF684/2020 EPI_ISL_428470 2020-01-27 Guangdong Provincial Center for Diseases Control and Prevention;Guangdong Provincial Institute of Public Health School of Public Health, The University of Hong Kong Bosheng Li et al

Guangdong/20SF685/2020 EPI_ISL_428471 2020-01-27 Guangdong Provincial Center for Diseases Control and Prevention;Guangdong Provincial Institute of Public Health School of Public Health, The University of Hong Kong Bosheng Li et al

Guangdong/20SF758/2020 EPI_ISL_428473 2020-01-27 Guangdong Provincial Center for Diseases Control and Prevention;Guangdong Provincial Institute of Public Health School of Public Health, The University of Hong Kong Bosheng Li et al

Guangzhou/GZMU0058/2020 EPI_ISL_429101 2020-01-27 The First Affiliated Hospital of Guangzhou Medical University BGI-shenzhen & The First Affiliated Hospital of Guangzhou Medical University et al

Guangzhou/GZMU0059/2020 EPI_ISL_429102 2020-01-27 The First Affiliated Hospital of Guangzhou Medical University BGI-shenzhen & The First Affiliated Hospital of Guangzhou Medical University et al

HongKong/HKU-903b/2020 EPI_ISL_434566 2020-01-27 unknown Microbiology To et al

HongKong/HKU-907a/2020 EPI_ISL_434567 2020-01-27 unknown Microbiology To et al

HongKong/HKU-908a/2020 EPI_ISL_434569 2020-01-27 unknown Microbiology To et al

Jian/JX169/2020 EPI_ISL_421256 2020-01-27 Jiangxi Province Center for Disease Control and Prevention Jiangxi Province Center for Disease Control and Prevention JianXiong Li et al

Nanchang/JX149/2020 EPI_ISL_421247 2020-01-27 Jiangxi Province Center for Disease Control and Prevention Jiangxi Province Center for Disease Control and Prevention JianXiong Li et al

Pingxiang/JX151/2020 EPI_ISL_421259 2020-01-27 Jiangxi Province Center for Disease Control and Prevention Jiangxi Province Center for Disease Control and Prevention JianXiong Li et al

Singapore/15/2020 EPI_ISL_418996 2020-01-27 National Public Health Laboratory, National Centre for Infectious Diseases National Public Health Laboratory, National Centre for Infectious Diseases Mak TM et al

Singapore/7/2020 EPI_ISL_410713 2020-01-27 National Public Health Laboratory, National Centre for Infectious Diseases National Public Health Laboratory, National Centre for Infectious Diseases Octavia S et al

USA/CA6/2020 EPI_ISL_410044 2020-01-27 California Department of Public Health Pathogen Discovery, Respiratory Viruses Branch, Division of Viral Diseases, Centers for Disease Control and Prevention Jing Zhang et al

Australia/QLD01/2020 EPI_ISL_407894 2020-01-28 Pathology Queensland Public Health Virology Laboratory Ben Huang et al

Beijing/231/2020 EPI_ISL_413519 2020-01-28 unknown Infectious Disease Control Center Li et al

Beijing/233/2020 EPI_ISL_413520 2020-01-28 unknown Infectious Disease Control Center Li et al

Beijing/235/2020 EPI_ISL_413521 2020-01-28 unknown Infectious Disease Control Center Li et al

Beijing/Wuhan_IME-BJ03/2020 EPI_ISL_424357 2020-01-28 unknown Beijing Institute of Microbiology and Epidemiology Fan et al

Beijing/Wuhan_IME-BJ04/2020 EPI_ISL_424358 2020-01-28 unknown Beijing Institute of Microbiology and Epidemiology Fan et al

France/IDF0386-islP1/2020 EPI_ISL_411219 2020-01-28 Department of Infectious and Tropical Diseases, Bichat Claude Bernard Hospital, Paris Laboratoire Virpath, CIRI U111, UCBL1, INSERM, CNRS, ENS Lyon Olivier Terrier et al

France/IDF0386-islP3/2020 EPI_ISL_411220 2020-01-28 Department of Infectious and Tropical Diseases, Bichat Claude Bernard Hospital, Paris Laboratoire Virpath, CIRI U111, UCBL1, INSERM, CNRS, ENS Lyon Olivier Terrier et al

Germany/BavPat1/2020 EPI_ISL_406862 2020-01-28 Charit√© Universit√§tsmedizin Berlin, Institute of Virology; Institut f√ºr Mikrobiologie der Bundeswehr, Munich Charit√© Universit√§tsmedizin Berlin, Institute of Virology Victor M Corman et al

Guangdong/20SF753/2020 EPI_ISL_428472 2020-01-28 Guangdong Provincial Center for Diseases Control and Prevention;Guangdong Provincial Institute of Public Health School of Public Health, The University of Hong Kong Bosheng Li et al

Guangdong/20SF808/2020 EPI_ISL_428474 2020-01-28 Guangdong Provincial Center for Diseases Control and Prevention;Guangdong Provincial Institute of Public Health School of Public Health, The University of Hong Kong Bosheng Li et al

Guangdong/20SF812/2020 EPI_ISL_428475 2020-01-28 Guangdong Provincial Center for Diseases Control and Prevention;Guangdong Provincial Institute of Public Health School of Public Health, The University of Hong Kong Bosheng Li et al

Guangdong/20SF813/2020 EPI_ISL_428476 2020-01-28 Guangdong Provincial Center for Diseases Control and Prevention;Guangdong Provincial Institute of Public Health School of Public Health, The University of Hong Kong Bosheng Li et al

Hangzhou/ZJU-09/2020 EPI_ISL_416474 2020-01-28 State Key Laboratory for Diagnosis and Treatment of Infectious Diseases, National Clinical Research Center for Infectious Diseases, First Affiliated Hospital, Zhejiang University School of Medicine, Hangzhou, China 310003 State Key Laboratory for Diagnosis and Treatment of Infectious Diseases, National Clinical Research Center for Infectious Diseases, First Affiliated Hospital, Zhejiang University School of Medicine, Hangzhou, China 310003 Hangping Yao et al

Lishui/LS556/2020 EPI_ISL_429853 2020-01-28 Centers for Disease Control and Prevention of Lishui Department of InspectionÔºåCenters for Disease Control and Prevention of Lishui Wang Xiaoguang et al

Malaysia/IMR_WC085/2020 EPI_ISL_430443 2020-01-28 Institute for Medical Research, Infectious Disease Research Centre, National Institutes of Health, Ministry of Health Malaysia Institute for Medical Research, Infectious Disease Research Centre, National Institutes of Health, Ministry of Health Malaysia Suppiah.J et al

Shanghai/SH0004/2020 EPI_ISL_416318 2020-01-28 Shanghai Public Health Clinical Center, Shanghai Medical College, Fudan University National Research Center for Translational Medicine (Shanghai), Ruijin Hospital affiliated to Shanghai Jiao Tong University School of Medicine & Shanghai Public Health Clinical Center Shengyue Wang et al

Shanghai/SH0007/2020 EPI_ISL_416320 2020-01-28 Shanghai Public Health Clinical Center, Shanghai Medical College, Fudan University National Research Center for Translational Medicine (Shanghai), Ruijin Hospital affiliated to Shanghai Jiao Tong University School of Medicine & Shanghai Public Health Clinical Center Shengyue Wang et al

Shanghai/SH0008/2020 EPI_ISL_416321 2020-01-28 Shanghai Public Health Clinical Center, Shanghai Medical College, Fudan University National Research Center for Translational Medicine (Shanghai), Ruijin Hospital affiliated to Shanghai Jiao Tong University School of Medicine & Shanghai Public Health Clinical Center Shengyue Wang et al

Taiwan/4/2020 EPI_ISL_411927 2020-01-28 Taiwan Centers for Disease Control Taiwan Centers for Disease Control Ji-Rong Yang et al

USA/IL2/2020 EPI_ISL_410045 2020-01-28 IL Department of Public Health Chicago Laboratory Pathogen Discovery, Respiratory Viruses Branch, Division of Viral Diseases, Centers for Disease Control and Prevention Yan Li et al

Beijing/BJ251/2020 EPI_ISL_430742 2020-01-29 Chinese PLA Institute for Disease Control and Prevention Chinese PLA Institute for Disease Control and Prevention Peng Li et al

Beijing/BJ252/2020 EPI_ISL_430736 2020-01-29 Chinese PLA Institute for Disease Control and Prevention Chinese PLA Institute for Disease Control and Prevention Peng Li et al

Beijing/BJ287/2020 EPI_ISL_430722 2020-01-29 Chinese PLA Institute for Disease Control and Prevention Chinese PLA Institute for Disease Control and Prevention Peng Li et al

Beijing/BJ288/2020 EPI_ISL_430725 2020-01-29 Chinese PLA Institute for Disease Control and Prevention Chinese PLA Institute for Disease Control and Prevention Peng Li et al

Beijing/Wuhan_IME-BJ07/2020 EPI_ISL_424360 2020-01-29 unknown Beijing Institute of Microbiology and Epidemiology Fan et al

China/IQTC02/2020 EPI_ISL_412967 2020-01-29 unknown Technology Centre, Guangzhou Customs Shi et al

England/02/2020 EPI_ISL_407073 2020-01-29 Respiratory Virus Unit, Microbiology Services Colindale, Public Health England Respiratory Virus Unit, Microbiology Services Colindale, Public Health England Monica Galiano et al

France/IDF0515-isl/2020 EPI_ISL_410984 2020-01-29 Department of Infectious and Tropical Diseases, Bichat Claude Bernard Hospital, Paris National Reference Center for Viruses of Respiratory Infections, Institut Pasteur, Paris M√©lanie Albert et al

France/IDF0515/2020 EPI_ISL_408430 2020-01-29 Department of Infectious and Tropical Diseases, Bichat Claude Bernard Hospital, Paris National Reference Center for Viruses of Respiratory Infections, Institut Pasteur, Paris M√©lanie Albert et al

France/IDF0626/2020 EPI_ISL_408431 2020-01-29 Sorbonne Universit√©, Inserm et Assistance Publique-H√¥pitaux de Paris (Piti√© Salp√©tri√®re) National Reference Center for Viruses of Respiratory Infections, Institut Pasteur, Paris M√©lanie Albert et al

Guangdong/20SF1107/2020 EPI_ISL_428441 2020-01-29 Guangdong Provincial Center for Diseases Control and Prevention;Guangdong Provincial Institute of Public Health School of Public Health, The University of Hong Kong Bosheng Li et al

Guangdong/20SF840/2020 EPI_ISL_428478 2020-01-29 Guangdong Provincial Center for Diseases Control and Prevention;Guangdong Provincial Institute of Public Health School of Public Health, The University of Hong Kong Bosheng Li et al

Guangzhou/GZMU0051/2020 EPI_ISL_429096 2020-01-29 The First Affiliated Hospital of Guangzhou Medical University BGI-shenzhen & The First Affiliated Hospital of Guangzhou Medical University et al

Guangzhou/GZMU0052/2020 EPI_ISL_429097 2020-01-29 The First Affiliated Hospital of Guangzhou Medical University BGI-shenzhen & The First Affiliated Hospital of Guangzhou Medical University et al

Guangzhou/GZMU0054/2020 EPI_ISL_429098 2020-01-29 The First Affiliated Hospital of Guangzhou Medical University BGI-shenzhen & The First Affiliated Hospital of Guangzhou Medical University et al

Guangzhou/GZMU0055/2020 EPI_ISL_429105 2020-01-29 The First Affiliated Hospital of Guangzhou Medical University BGI-shenzhen & The First Affiliated Hospital of Guangzhou Medical University et al

HongKong/HKU-907b/2020 EPI_ISL_434568 2020-01-29 unknown Microbiology To et al

HongKong/HKU-908b/2020 EPI_ISL_434570 2020-01-29 unknown Microbiology To et al

Italy/INMI1-cs/2020 EPI_ISL_410546 2020-01-29 INMI Lazzaro Spallanzani IRCCS Laboratory of Virology, INMI Lazzaro Spallanzani IRCCS Maria R. Capobianchi et al

Italy/INMI1-isl/2020 EPI_ISL_410545 2020-01-29 INMI Lazzaro Spallanzani IRCCS Laboratory of Virology, INMI Lazzaro Spallanzani IRCCS Maria R. Capobianchi et al

Italy/SPL1/2020 EPI_ISL_412974 2020-01-29 Department of Infectious Diseases, Istituto Superiore di Sanit√†, Rome, Italy Virology Laboratory, Scientific Department, Army Medical Center Paola Stefanelli et al

Japan/KY-V-029/2020 EPI_ISL_408669 2020-01-29 Dept. of Virology III, National Institute of Infectious Diseases Pathogen Genomics Center, National Institute of Infectious Diseases Tsuyoshi Sekizuka et al

Japan/TY-WK-012/2020 EPI_ISL_408665 2020-01-29 Dept. of Virology III, National Institute of Infectious Diseases Pathogen Genomics Center, National Institute of Infectious Diseases Tsuyoshi Sekizuka et al

Nanchang/JX155/2020 EPI_ISL_421262 2020-01-29 Jiangxi Province Center for Disease Control and Prevention Jiangxi Province Center for Disease Control and Prevention JianXiong Li et al

Nanchang/JX174/2020 EPI_ISL_421238 2020-01-29 Jiangxi Province Center for Disease Control and Prevention Jiangxi Province Center for Disease Control and Prevention JianXiong Li et al

Nanchang/JX176/2020 EPI_ISL_421261 2020-01-29 Jiangxi Province Center for Disease Control and Prevention Jiangxi Province Center for Disease Control and Prevention JianXiong Li et al

Shanghai/SH0009/2020 EPI_ISL_416322 2020-01-29 Shanghai Public Health Clinical Center, Shanghai Medical College, Fudan University National Research Center for Translational Medicine (Shanghai), Ruijin Hospital affiliated to Shanghai Jiao Tong University School of Medicine & Shanghai Public Health Clinical Center Shengyue Wang et al

Shanghai/SH0010/2020 EPI_ISL_416323 2020-01-29 Shanghai Public Health Clinical Center, Shanghai Medical College, Fudan University National Research Center for Translational Medicine (Shanghai), Ruijin Hospital affiliated to Shanghai Jiao Tong University School of Medicine & Shanghai Public Health Clinical Center Shengyue Wang et al

Shanghai/SH0011/2020 EPI_ISL_416324 2020-01-29 Shanghai Public Health Clinical Center, Shanghai Medical College, Fudan University National Research Center for Translational Medicine (Shanghai), Ruijin Hospital affiliated to Shanghai Jiao Tong University School of Medicine & Shanghai Public Health Clinical Center Shengyue Wang et al

Singapore/20/2020 EPI_ISL_418993 2020-01-29 National Public Health Laboratory, National Centre for Infectious Diseases National Public Health Laboratory, National Centre for Infectious Diseases Mak TM et al

Thailand/Bangkok_323/2020 EPI_ISL_447916 2020-01-29 n/a National Institute of Health. Department of medical Sciences, Ministry of Public Health, Thailand Pilailuk et al

Thailand/Nonthaburi_363/2020 EPI_ISL_447917 2020-01-29 n/a National Institute of Health. Department of medical Sciences, Ministry of Public Health, Thailand Pilailuk et al

Thailand/SI200615-NT/2020 EPI_ISL_437618 2020-01-29 unknown Faculty of Medicine Rodpan et al

USA/CA3/2020 EPI_ISL_408008 2020-01-29 California Department of Health Pathogen Discovery, Respiratory Viruses Branch, Division of Viral Diseases, Centers for Disease Control and Prevention Krista Queen et al

USA/CA4/2020 EPI_ISL_408009 2020-01-29 California Department of Health Pathogen Discovery, Respiratory Viruses Branch, Division of Viral Diseases, Centers for Disease Control and Prevention Krista Queen et al

USA/CA5/2020 EPI_ISL_408010 2020-01-29 California Department of Health Pathogen Discovery, Respiratory Viruses Branch, Division of Viral Diseases, Centers for Disease Control and Prevention Ying Tao et al

USA/MA1/2020 EPI_ISL_409067 2020-01-29 Massachusetts Department of Public Health Pathogen Discovery, Respiratory Viruses Branch, Division of Viral Diseases, Centers for Disease Control and Prevention Clinton R. Paden et al

UnitedArabEmirates/L5630/2020 EPI_ISL_435137 2020-01-29 Mohammed Bin Rashid University of Medicine and Health Sciences Al Jalila Genomics Center Ahmad Abou Tayoun et al

Australia/QLD02/2020 EPI_ISL_407896 2020-01-30 Pathology Queensland Public Health Virology Laboratory Ben Huang et al

Guangdong/2020XN4243-P0035/2020 EPI_ISL_413853 2020-01-30 Guangdong Provincial Institution of Public Health, Guangdong Provinical Center for Disease Control and Prevention Guangdong Provincial Institution of Public Health Jing Lu et al

Guangdong/2020XN4273-P0036/2020 EPI_ISL_413860 2020-01-30 Guangdong Provincial Institution of Public Health, Guangdong Provinical Center for Disease Control and Prevention Guangdong Provincial Institution of Public Health Jing Lu et al

Guangdong/2020XN4459-P0041/2020 EPI_ISL_413858 2020-01-30 Guangdong Provincial Institution of Public Health, Guangdong Provinical Center for Disease Control and Prevention Guangdong Provincial Institution of Public Health Jing Lu et al

Guangdong/2020XN4475-P0042/2020 EPI_ISL_413854 2020-01-30 Guangdong Provincial Institution of Public Health, Guangdong Provinical Center for Disease Control and Prevention Guangdong Provincial Institution of Public Health Jing Lu et al

Guangdong/20SF1152/2020 EPI_ISL_428444 2020-01-30 Guangdong Provincial Center for Diseases Control and Prevention;Guangdong Provincial Institute of Public Health School of Public Health, The University of Hong Kong Bosheng Li et al

HongKong/VB20024950/2020 EPI_ISL_412029 2020-01-30 Hong Kong Department of Health The University of Hong Kong Dominic N.C. Tsang et al

Korea/KCDC2002/2020 EPI_ISL_425118 2020-01-30 Division of Viral Diseases, Center for Laboratory Control of Infectious Diseases, Korea Centers for Diseases Control and Prevention Division of Viral Diseases, Center for Laboratory Control of Infectious Diseases, Korea Centers for Diseases Control and Prevention Jeong-Min Kim et al

Malaysia/MKAK-CL-2020-5096/2020 EPI_ISL_416885 2020-01-30 National Public Health Laboratory Malaysia Genome Institute Mohd Noor Mat Isa et al

Nanchang/JX177/2020 EPI_ISL_421241 2020-01-30 Jiangxi Province Center for Disease Control and Prevention Jiangxi Province Center for Disease Control and Prevention JianXiong Li et al

Shandong/LY008/2020 EPI_ISL_414941 2020-01-30 Shandong Provincial Center for Disease Control and Prevention Beijing Institute of Microbiology and Epidemiology Xiao-Lin Jiang et al

Shanghai/SH0013/2020 EPI_ISL_416326 2020-01-30 Shanghai Public Health Clinical Center, Shanghai Medical College, Fudan University National Research Center for Translational Medicine (Shanghai), Ruijin Hospital affiliated to Shanghai Jiao Tong University School of Medicine & Shanghai Public Health Clinical Center Shengyue Wang et al

Shanghai/SH0022/2020 EPI_ISL_416331 2020-01-30 Shanghai Public Health Clinical Center, Shanghai Medical College, Fudan University National Research Center for Translational Medicine (Shanghai), Ruijin Hospital affiliated to Shanghai Jiao Tong University School of Medicine & Shanghai Public Health Clinical Center Shengyue Wang et al

Shanghai/SH0023/2020 EPI_ISL_416332 2020-01-30 Shanghai Public Health Clinical Center, Shanghai Medical College, Fudan University National Research Center for Translational Medicine (Shanghai), Ruijin Hospital affiliated to Shanghai Jiao Tong University School of Medicine & Shanghai Public Health Clinical Center Shengyue Wang et al

Shanghai/SH0024/2020 EPI_ISL_416333 2020-01-30 Shanghai Public Health Clinical Center, Shanghai Medical College, Fudan University National Research Center for Translational Medicine (Shanghai), Ruijin Hospital affiliated to Shanghai Jiao Tong University School of Medicine & Shanghai Public Health Clinical Center Shengyue Wang et al

Shanghai/SH0040/2020 EPI_ISL_416349 2020-01-30 Shanghai Public Health Clinical Center, Shanghai Medical College, Fudan University National Research Center for Translational Medicine (Shanghai), Ruijin Hospital affiliated to Shanghai Jiao Tong University School of Medicine & Shanghai Public Health Clinical Center Shengyue Wang et al

Shanghai/SH0043/2020 EPI_ISL_416352 2020-01-30 Shanghai Public Health Clinical Center, Shanghai Medical College, Fudan University National Research Center for Translational Medicine (Shanghai), Ruijin Hospital affiliated to Shanghai Jiao Tong University School of Medicine & Shanghai Public Health Clinical Center Shengyue Wang et al

Shanghai/SH0058/2020 EPI_ISL_416365 2020-01-30 Shanghai Public Health Clinical Center, Shanghai Medical College, Fudan University National Research Center for Translational Medicine (Shanghai), Ruijin Hospital affiliated to Shanghai Jiao Tong University School of Medicine & Shanghai Public Health Clinical Center Shengyue Wang et al

Shanghai/SH0059/2020 EPI_ISL_416366 2020-01-30 Shanghai Public Health Clinical Center, Shanghai Medical College, Fudan University National Research Center for Translational Medicine (Shanghai), Ruijin Hospital affiliated to Shanghai Jiao Tong University School of Medicine & Shanghai Public Health Clinical Center Shengyue Wang et al

SouthKorea/KCDC05/2020 EPI_ISL_412869 2020-01-30 Division of Viral Diseases, Center for Laboratory Control of Infectious Diseases, Korea Centers for Diseases Control and Prevention Division of Viral Diseases, Center for Laboratory Control of Infectious Diseases, Korea Centers for Diseases Control and Prevention Jeong-Min Kim et al

SouthKorea/KCDC06/2020 EPI_ISL_412870 2020-01-30 Division of Viral Diseases, Center for Laboratory Control of Infectious Diseases, Korea Centers for Diseases Control and Prevention Division of Viral Diseases, Center for Laboratory Control of Infectious Diseases, Korea Centers for Diseases Control and Prevention Jeong-Min Kim et al

Beijing/BJ390/2020 EPI_ISL_430730 2020-01-31 Chinese PLA Institute for Disease Control and Prevention Chinese PLA Institute for Disease Control and Prevention Peng Li et al

Guangdong/2020XN4448-P0002/2020 EPI_ISL_413857 2020-01-31 Guangdong Provincial Institution of Public Health, Guangdong Provinical Center for Disease Control and Prevention Guangdong Provincial Institution of Public Health Jing Lu et al

India/1-31/2020 EPI_ISL_413523 2020-01-31 Indian Council of Medical Research-National Institute of Virology National Influenza Center, Indian Council of Medical Research-National Institute of Virology Potdar V et al

Japan/TY-WK-501/2020 EPI_ISL_408666 2020-01-31 Dept. of Virology III, National Institute of Infectious Diseases Pathogen Genomics Center, National Institute of Infectious Diseases Tsuyoshi Sekizuka et al

Japan/TY-WK-521/2020 EPI_ISL_408667 2020-01-31 Dept. of Virology III, National Institute of Infectious Diseases Pathogen Genomics Center, National Institute of Infectious Diseases Tsuyoshi Sekizuka et al

Shanghai/SH0094/2020 EPI_ISL_416390 2020-01-31 Shanghai Public Health Clinical Center, Shanghai Medical College, Fudan University National Research Center for Translational Medicine (Shanghai), Ruijin Hospital affiliated to Shanghai Jiao Tong University School of Medicine & Shanghai Public Health Clinical Center Shengyue Wang et al

SouthKorea/KCDC07/2020 EPI_ISL_412871 2020-01-31 Division of Viral Diseases, Center for Laboratory Control of Infectious Diseases, Korea Centers for Diseases Control and Prevention Division of Viral Diseases, Center for Laboratory Control of Infectious Diseases, Korea Centers for Diseases Control and Prevention Jeong-Min Kim et al

Taiwan/5/2020 EPI_ISL_428489 2020-01-31 Centers for Disease Control, R.O.C. (Taiwan) Centers for Disease Control, R.O.C. (Taiwan) Ji-Rong Yang et al

Taiwan/8/2020 EPI_ISL_428488 2020-01-31 Centers for Disease Control, R.O.C. (Taiwan) Centers for Disease Control, R.O.C. (Taiwan) Ji-Rong Yang et al

Taiwan/NTU01/2020 EPI_ISL_408489 2020-01-31 Department of Laboratory Medicine, National Taiwan University Hospital Microbial Genomics Core Lab, National Taiwan University Centers of Genomic and Precision Medicine Shiou-Hwei Yeh et al

Thailand/Bangkok_238/2020 EPI_ISL_447913 2020-01-31 n/a National Institute of Health. Department of medical Sciences, Ministry of Public Health, Thailand Pilailuk et al

USA/WI1/2020 EPI_ISL_408670 2020-01-31 Wisconsin Department of Health Services Pathogen Discovery, Respiratory Viruses Branch, Division of Viral Diseases, Centers for Disease Control and Prevention Jing Zhang et al

Guangdong/GD2020080-P0010/2020 EPI_ISL_413861 2020-02-01 Guangdong Provincial Institution of Public Health, Guangdong Provinical Center for Disease Control and Prevention Guangdong Provincial Institution of Public Health Jing Lu et al

Guangdong/GD2020087-P0008/2020 EPI_ISL_413863 2020-02-01 Guangdong Provincial Institution of Public Health, Guangdong Provinical Center for Disease Control and Prevention Guangdong Provincial Institution of Public Health Jing Lu et al

Guangzhou/GZMU0033/2020 EPI_ISL_429085 2020-02-01 The First Affiliated Hospital of Guangzhou Medical University BGI-shenzhen & The First Affiliated Hospital of Guangzhou Medical University et al

Guangzhou/GZMU0034/2020 EPI_ISL_429086 2020-02-01 The First Affiliated Hospital of Guangzhou Medical University BGI-shenzhen & The First Affiliated Hospital of Guangzhou Medical University et al

Guangzhou/GZMU0035/2020 EPI_ISL_429088 2020-02-01 The First Affiliated Hospital of Guangzhou Medical University BGI-shenzhen & The First Affiliated Hospital of Guangzhou Medical University et al

Guangzhou/GZMU0036/2020 EPI_ISL_429104 2020-02-01 The First Affiliated Hospital of Guangzhou Medical University BGI-shenzhen & The First Affiliated Hospital of Guangzhou Medical University et al

HongKong/VB20026565/2020 EPI_ISL_412030 2020-02-01 Hong Kong Department of Health School of Public Health, The University of Hon g Kong Dominic N.C. Tsang et al

Japan/UT-NCGM02/2020 EPI_ISL_418809 2020-02-01 University of Wisconsin - Madison: Influenza Research Institute University of Wisconsin Madison, AIDS Vaccine Research Laboratories Katarina Braun et al

Lishui/LS557/2020 EPI_ISL_429854 2020-02-01 Centers for Disease Control and Prevention of Lishui Department of InspectionÔºåCenters for Disease Control and Prevention of Lishui Wang Xiaoguang et al

Shanghai/SH0027/2020 EPI_ISL_416336 2020-02-01 Shanghai Public Health Clinical Center, Shanghai Medical College, Fudan University National Research Center for Translational Medicine (Shanghai), Ruijin Hospital affiliated to Shanghai Jiao Tong University School of Medicine & Shanghai Public Health Clinical Center Shengyue Wang et al

Shanghai/SH0029/2020 EPI_ISL_416338 2020-02-01 Shanghai Public Health Clinical Center, Shanghai Medical College, Fudan University National Research Center for Translational Medicine (Shanghai), Ruijin Hospital affiliated to Shanghai Jiao Tong University School of Medicine & Shanghai Public Health Clinical Center Shengyue Wang et al

Shanghai/SH0032/2020 EPI_ISL_416341 2020-02-01 Shanghai Public Health Clinical Center, Shanghai Medical College, Fudan University National Research Center for Translational Medicine (Shanghai), Ruijin Hospital affiliated to Shanghai Jiao Tong University School of Medicine & Shanghai Public Health Clinical Center Shengyue Wang et al

Shanghai/SH0053/2020 EPI_ISL_416361 2020-02-01 Shanghai Public Health Clinical Center, Shanghai Medical College, Fudan University National Research Center for Translational Medicine (Shanghai), Ruijin Hospital affiliated to Shanghai Jiao Tong University School of Medicine & Shanghai Public Health Clinical Center Shengyue Wang et al

Shanghai/SH0060/2020 EPI_ISL_416367 2020-02-01 Shanghai Public Health Clinical Center, Shanghai Medical College, Fudan University National Research Center for Translational Medicine (Shanghai), Ruijin Hospital affiliated to Shanghai Jiao Tong University School of Medicine & Shanghai Public Health Clinical Center Shengyue Wang et al

Shanghai/SH0067/2020 EPI_ISL_416370 2020-02-01 Shanghai Public Health Clinical Center, Shanghai Medical College, Fudan University National Research Center for Translational Medicine (Shanghai), Ruijin Hospital affiliated to Shanghai Jiao Tong University School of Medicine & Shanghai Public Health Clinical Center Shengyue Wang et al

Shanghai/SH0076/2020 EPI_ISL_416379 2020-02-01 Shanghai Public Health Clinical Center, Shanghai Medical College, Fudan University National Research Center for Translational Medicine (Shanghai), Ruijin Hospital affiliated to Shanghai Jiao Tong University School of Medicine & Shanghai Public Health Clinical Center Shengyue Wang et al

Shanghai/SH0079/2020 EPI_ISL_416381 2020-02-01 Shanghai Public Health Clinical Center, Shanghai Medical College, Fudan University National Research Center for Translational Medicine (Shanghai), Ruijin Hospital affiliated to Shanghai Jiao Tong University School of Medicine & Shanghai Public Health Clinical Center Shengyue Wang et al

Shanghai/SH0088/2020 EPI_ISL_416387 2020-02-01 Shanghai Public Health Clinical Center, Shanghai Medical College, Fudan University National Research Center for Translational Medicine (Shanghai), Ruijin Hospital affiliated to Shanghai Jiao Tong University School of Medicine & Shanghai Public Health Clinical Center Shengyue Wang et al

Singapore/3/2020 EPI_ISL_407988 2020-02-01 National Centre for Infectious Diseases Programme in Emerging Infectious Diseases, Duke-NUS Medical School Danielle E Anderson et al

SouthKorea/KCDC12/2020 EPI_ISL_412872 2020-02-01 Division of Viral Diseases, Center for Laboratory Control of Infectious Diseases, Korea Centers for Diseases Control and Prevention Division of Viral Diseases, Center for Laboratory Control of Infectious Diseases, Korea Centers for Diseases Control and Prevention Jeong-Min Kim et al

Thailand/Bangkok_580/2020 EPI_ISL_447918 2020-02-01 n/a National Institute of Health. Department of medical Sciences, Ministry of Public Health, Thailand Pilailuk et al

Thailand/SI200893-NT/2020 EPI_ISL_437624 2020-02-01 unknown Faculty of Medicine Rodpan et al

Beijing/BJ492/2020 EPI_ISL_430728 2020-02-02 Chinese PLA Institute for Disease Control and Prevention Chinese PLA Institute for Disease Control and Prevention Peng Li et al

France/IDF0571/2020 EPI_ISL_411218 2020-02-02 Department of Infectious and Tropical Diseases, Bichat Claude Bernard Hospital, Paris Laboratoire Virpath, CIRI U111, UCBL1, INSERM, CNRS, ENS Lyon Olivier Terrier et al

Hangzhou/ZJU-06/2020 EPI_ISL_416047 2020-02-02 State Key Laboratory for Diagnosis and Treatment of Infectious Diseases, National Clinical Research Center for Infectious Diseases, First Affiliated Hospital, Zhejiang University School of Medicine, Hangzhou, China 310003 State Key Laboratory for Diagnosis and Treatment of Infectious Diseases, National Clinical Research Center for Infectious Diseases, First Affiliated Hospital, Zhejiang University School of Medicine, Hangzhou, China 310003 Hangping Yao et al

Korea/KCDC2004/2020 EPI_ISL_426164 2020-02-02 Division of Viral Diseases, Center for Laboratory Control of Infectious Diseases, Korea Centers for Diseases Control and Prevention Division of Viral Diseases, Center for Laboratory Control of Infectious Diseases, Korea Centers for Diseases Control and Prevention Jeong-Min Kim et al

Korea/KCDC2006/2020 EPI_ISL_426168 2020-02-02 Division of Viral Diseases, Center for Laboratory Control of Infectious Diseases, Korea Centers for Diseases Control and Prevention Division of Viral Diseases, Center for Laboratory Control of Infectious Diseases, Korea Centers for Diseases Control and Prevention Jeong-Min Kim et al

Shanghai/SH0012/2020 EPI_ISL_416325 2020-02-02 Shanghai Public Health Clinical Center, Shanghai Medical College, Fudan University National Research Center for Translational Medicine (Shanghai), Ruijin Hospital affiliated to Shanghai Jiao Tong University School of Medicine & Shanghai Public Health Clinical Center Shengyue Wang et al

Shanghai/SH0021/2020 EPI_ISL_416330 2020-02-02 Shanghai Public Health Clinical Center, Shanghai Medical College, Fudan University National Research Center for Translational Medicine (Shanghai), Ruijin Hospital affiliated to Shanghai Jiao Tong University School of Medicine & Shanghai Public Health Clinical Center Shengyue Wang et al

Shanghai/SH0026/2020 EPI_ISL_416335 2020-02-02 Shanghai Public Health Clinical Center, Shanghai Medical College, Fudan University National Research Center for Translational Medicine (Shanghai), Ruijin Hospital affiliated to Shanghai Jiao Tong University School of Medicine & Shanghai Public Health Clinical Center Shengyue Wang et al

Shanghai/SH0031/2020 EPI_ISL_416340 2020-02-02 Shanghai Public Health Clinical Center, Shanghai Medical College, Fudan University National Research Center for Translational Medicine (Shanghai), Ruijin Hospital affiliated to Shanghai Jiao Tong University School of Medicine & Shanghai Public Health Clinical Center Shengyue Wang et al

Shanghai/SH0054/2020 EPI_ISL_416362 2020-02-02 Shanghai Public Health Clinical Center, Shanghai Medical College, Fudan University National Research Center for Translational Medicine (Shanghai), Ruijin Hospital affiliated to Shanghai Jiao Tong University School of Medicine & Shanghai Public Health Clinical Center Shengyue Wang et al

Shanghai/SH01/2020 EPI_ISL_414510 2020-02-02 unknown Key Laboratory of Medical Molecular Virology (MOE/NHC/CAMS) Zhang et al

Shanghai/SH0107/2020 EPI_ISL_416397 2020-02-02 Shanghai Public Health Clinical Center, Shanghai Medical College, Fudan University National Research Center for Translational Medicine (Shanghai), Ruijin Hospital affiliated to Shanghai Jiao Tong University School of Medicine & Shanghai Public Health Clinical Center Shengyue Wang et al

Shanghai/SH0109/2020 EPI_ISL_416398 2020-02-02 Shanghai Public Health Clinical Center, Shanghai Medical College, Fudan University National Research Center for Translational Medicine (Shanghai), Ruijin Hospital affiliated to Shanghai Jiao Tong University School of Medicine & Shanghai Public Health Clinical Center Shengyue Wang et al

Shanghai/SH0112/2020 EPI_ISL_416400 2020-02-02 Shanghai Public Health Clinical Center, Shanghai Medical College, Fudan University National Research Center for Translational Medicine (Shanghai), Ruijin Hospital affiliated to Shanghai Jiao Tong University School of Medicine & Shanghai Public Health Clinical Center Shengyue Wang et al

Shanghai/SH0114/2020 EPI_ISL_416401 2020-02-02 Shanghai Public Health Clinical Center, Shanghai Medical College, Fudan University National Research Center for Translational Medicine (Shanghai), Ruijin Hospital affiliated to Shanghai Jiao Tong University School of Medicine & Shanghai Public Health Clinical Center Shengyue Wang et al

Shanghai/SH0117/2020 EPI_ISL_416403 2020-02-02 Shanghai Public Health Clinical Center, Shanghai Medical College, Fudan University National Research Center for Translational Medicine (Shanghai), Ruijin Hospital affiliated to Shanghai Jiao Tong University School of Medicine & Shanghai Public Health Clinical Center Shengyue Wang et al

Shanghai/SH0121/2020 EPI_ISL_416405 2020-02-02 Shanghai Public Health Clinical Center, Shanghai Medical College, Fudan University National Research Center for Translational Medicine (Shanghai), Ruijin Hospital affiliated to Shanghai Jiao Tong University School of Medicine & Shanghai Public Health Clinical Center Shengyue Wang et al

Shanghai/SH0128/2020 EPI_ISL_416409 2020-02-02 Shanghai Public Health Clinical Center, Shanghai Medical College, Fudan University National Research Center for Translational Medicine (Shanghai), Ruijin Hospital affiliated to Shanghai Jiao Tong University School of Medicine & Shanghai Public Health Clinical Center Shengyue Wang et al

Shangrao/JX1215/2020 EPI_ISL_421257 2020-02-02 Jiangxi Province Center for Disease Control and Prevention Jiangxi Province Center for Disease Control and Prevention JianXiong Li et al

Singapore/11/2020 EPI_ISL_410719 2020-02-02 National Public Health Laboratory National Public Health Laboratory Octavia S et al

Belgium/GHB-03021/2020 EPI_ISL_407976 2020-02-03 KU Leuven, Clinical and Epidemiological Virology KU Leuven, Clinical and Epidemiological Virology Bert Vanmechelen et al

Hangzhou/ZJU-010/2020 EPI_ISL_418990 2020-02-03 State Key Laboratory for Diagnosis and Treatment of Infectious Diseases, National Clinical Research Center for Infectious Diseases, First Affiliated Hospital, Zhejiang University School of Medicine, Hangzhou, China 310003 State Key Laboratory for Diagnosis and Treatment of Infectious Diseases, National Clinical Research Center for Infectious Diseases, First Affiliated Hospital, Zhejiang University School of Medicine, Hangzhou, China 310003 Hangping Yao et al

Hangzhou/ZJU-07/2020 EPI_ISL_416425 2020-02-03 State Key Laboratory for Diagnosis and Treatment of Infectious Diseases, National Clinical Research Center for Infectious Diseases, First Affiliated Hospital, Zhejiang University School of Medicine, Hangzhou, China 310003 State Key Laboratory for Diagnosis and Treatment of Infectious Diseases, National Clinical Research Center for Infectious Diseases, First Affiliated Hospital, Zhejiang University School of Medicine, Hangzhou, China 310003 Hangping Yao et al

Shanghai/SH0030/2020 EPI_ISL_416339 2020-02-03 Shanghai Public Health Clinical Center, Shanghai Medical College, Fudan University National Research Center for Translational Medicine (Shanghai), Ruijin Hospital affiliated to Shanghai Jiao Tong University School of Medicine & Shanghai Public Health Clinical Center Shengyue Wang et al

Shangrao/JX1176/2020 EPI_ISL_421251 2020-02-03 Jiangxi Province Center for Disease Control and Prevention Jiangxi Province Center for Disease Control and Prevention JianXiong Li et al

Shangrao/JX1177/2020 EPI_ISL_421250 2020-02-03 Jiangxi Province Center for Disease Control and Prevention Jiangxi Province Center for Disease Control and Prevention JianXiong Li et al

Shangrao/JX1178/2020 EPI_ISL_421254 2020-02-03 Jiangxi Province Center for Disease Control and Prevention Jiangxi Province Center for Disease Control and Prevention JianXiong Li et al

Singapore/4/2020 EPI_ISL_410535 2020-02-03 National Centre for Infectious Diseases Programme in Emerging Infectious Diseases, Duke-NUS Medical School Danielle E Anderson et al

Singapore/8/2020 EPI_ISL_410714 2020-02-03 National Public Health Laboratory, National Centre for Infectious Diseases National Public Health Laboratory, National Centre for Infectious Diseases Octavia S et al

Thailand/SI201712-NT/2020 EPI_ISL_437617 2020-02-03 unknown Faculty of Medicine Rodpan et al

Beijing/BJ556/2020 EPI_ISL_430733 2020-02-04 Chinese PLA Institute for Disease Control and Prevention Chinese PLA Institute for Disease Control and Prevention Peng Li et al

Beijing/BJ561/2020 EPI_ISL_430731 2020-02-04 Chinese PLA Institute for Disease Control and Prevention Chinese PLA Institute for Disease Control and Prevention Peng Li et al

Hangzhou/ZJU-011/2020 EPI_ISL_418991 2020-02-04 State Key Laboratory for Diagnosis and Treatment of Infectious Diseases, National Clinical Research Center for Infectious Diseases, First Affiliated Hospital, Zhejiang University School of Medicine, Hangzhou, China 310003 State Key Laboratory for Diagnosis and Treatment of Infectious Diseases, National Clinical Research Center for Infectious Diseases, First Affiliated Hospital, Zhejiang University School of Medicine, Hangzhou, China 310003 Hangping Yao et al

Korea/KCDC2005/2020 EPI_ISL_426166 2020-02-04 Division of Viral Diseases, Center for Laboratory Control of Infectious Diseases, Korea Centers for Diseases Control and Prevention Division of Viral Diseases, Center for Laboratory Control of Infectious Diseases, Korea Centers for Diseases Control and Prevention Jeong-Min Kim et al

Malaysia/MKAK-CL-2020-6430/2020 EPI_ISL_416886 2020-02-04 National Public Health Laboratory Malaysia Genome Institute Mohd Noor Mat Isa et al

Shanghai/SH0028/2020 EPI_ISL_416337 2020-02-04 Shanghai Public Health Clinical Center, Shanghai Medical College, Fudan University National Research Center for Translational Medicine (Shanghai), Ruijin Hospital affiliated to Shanghai Jiao Tong University School of Medicine & Shanghai Public Health Clinical Center Shengyue Wang et al

Shanghai/SH0033/2020 EPI_ISL_416342 2020-02-04 Shanghai Public Health Clinical Center, Shanghai Medical College, Fudan University National Research Center for Translational Medicine (Shanghai), Ruijin Hospital affiliated to Shanghai Jiao Tong University School of Medicine & Shanghai Public Health Clinical Center Shengyue Wang et al

Shanghai/SH0044/2020 EPI_ISL_416353 2020-02-04 Shanghai Public Health Clinical Center, Shanghai Medical College, Fudan University National Research Center for Translational Medicine (Shanghai), Ruijin Hospital affiliated to Shanghai Jiao Tong University School of Medicine & Shanghai Public Health Clinical Center Shengyue Wang et al

Shanghai/SH0047/2020 EPI_ISL_416355 2020-02-04 Shanghai Public Health Clinical Center, Shanghai Medical College, Fudan University National Research Center for Translational Medicine (Shanghai), Ruijin Hospital affiliated to Shanghai Jiao Tong University School of Medicine & Shanghai Public Health Clinical Center Shengyue Wang et al

Shanghai/SH0083/2020 EPI_ISL_416384 2020-02-04 Shanghai Public Health Clinical Center, Shanghai Medical College, Fudan University National Research Center for Translational Medicine (Shanghai), Ruijin Hospital affiliated to Shanghai Jiao Tong University School of Medicine & Shanghai Public Health Clinical Center Shengyue Wang et al

Singapore/10/2020 EPI_ISL_410716 2020-02-04 National Public Health Laboratory, National Centre for Infectious Diseases National Centre for Infectious Diseases, National Centre for Infectious Diseases Octavia S et al

Singapore/9/2020 EPI_ISL_410715 2020-02-04 National Public Health Laboratory, National Centre for Infectious Diseases National Public Health Laboratory, National Centre for Infectious Diseases Octavia S et al

Thailand/Samutprakarn_840/2020 EPI_ISL_447919 2020-02-04 n/a National Institute of Health. Department of medical Sciences, Ministry of Public Health, Thailand Pilailuk et al

Australia/QLD03/2020 EPI_ISL_410717 2020-02-05 Pathology Queensland Public Health Virology Laboratory Ben Huang et al

Australia/QLD04/2020 EPI_ISL_410718 2020-02-05 Pathology Queensland Public Health Virology Laboratory Ben Huang et al

Beijing/BJ589/2020 EPI_ISL_430738 2020-02-05 Chinese PLA Institute for Disease Control and Prevention Chinese PLA Institute for Disease Control and Prevention Peng Li et al

China/IQTC01/2020 EPI_ISL_412966 2020-02-05 unknown Technology Centre, Guangzhou Customs Shi et al

Guangzhou/GZMU0017/2020 EPI_ISL_429079 2020-02-05 The First Affiliated Hospital of Guangzhou Medical University BGI-shenzhen & The First Affiliated Hospital of Guangzhou Medical University et al

Guangzhou/GZMU0019/2020 EPI_ISL_429080 2020-02-05 The First Affiliated Hospital of Guangzhou Medical University BGI-shenzhen & The First Affiliated Hospital of Guangzhou Medical University et al

Guangzhou/GZMU0023/2020 EPI_ISL_429082 2020-02-05 The First Affiliated Hospital of Guangzhou Medical University BGI-shenzhen & The First Affiliated Hospital of Guangzhou Medical University et al

Guangzhou/GZMU0024/2020 EPI_ISL_429083 2020-02-05 The First Affiliated Hospital of Guangzhou Medical University BGI-shenzhen & The First Affiliated Hospital of Guangzhou Medical University et al

Guangzhou/GZMU0025/2020 EPI_ISL_429084 2020-02-05 The First Affiliated Hospital of Guangzhou Medical University BGI-shenzhen & The First Affiliated Hospital of Guangzhou Medical University et al

Guangzhou/IQTC05/2020 EPI_ISL_444273 2020-02-05 unknown State Key Laboratory of Respiratory Disease Sun et al

Korea/KCDC2007/2020 EPI_ISL_426169 2020-02-05 Division of Viral Diseases, Center for Laboratory Control of Infectious Diseases, Korea Centers for Diseases Control and Prevention Division of Viral Diseases, Center for Laboratory Control of Infectious Diseases, Korea Centers for Diseases Control and Prevention Jeong-Min Kim et al

Korea/KCDC2008/2020 EPI_ISL_426171 2020-02-05 Division of Viral Diseases, Center for Laboratory Control of Infectious Diseases, Korea Centers for Diseases Control and Prevention Division of Viral Diseases, Center for Laboratory Control of Infectious Diseases, Korea Centers for Diseases Control and Prevention Jeong-Min Kim et al

Korea/KCDC2010/2020 EPI_ISL_426180 2020-02-05 Division of Viral Diseases, Center for Laboratory Control of Infectious Diseases, Korea Centers for Diseases Control and Prevention Division of Viral Diseases, Center for Laboratory Control of Infectious Diseases, Korea Centers for Diseases Control and Prevention Jeong-Min Kim et al

Shanghai/SH0050/2020 EPI_ISL_416358 2020-02-05 Shanghai Public Health Clinical Center, Shanghai Medical College, Fudan University National Research Center for Translational Medicine (Shanghai), Ruijin Hospital affiliated to Shanghai Jiao Tong University School of Medicine & Shanghai Public Health Clinical Center Shengyue Wang et al

Shanghai/SH0066/2020 EPI_ISL_416369 2020-02-05 Shanghai Public Health Clinical Center, Shanghai Medical College, Fudan University National Research Center for Translational Medicine (Shanghai), Ruijin Hospital affiliated to Shanghai Jiao Tong University School of Medicine & Shanghai Public Health Clinical Center Shengyue Wang et al

Shanghai/SH0068/2020 EPI_ISL_416371 2020-02-05 Shanghai Public Health Clinical Center, Shanghai Medical College, Fudan University National Research Center for Translational Medicine (Shanghai), Ruijin Hospital affiliated to Shanghai Jiao Tong University School of Medicine & Shanghai Public Health Clinical Center Shengyue Wang et al

Shanghai/SH0073/2020 EPI_ISL_416376 2020-02-05 Shanghai Public Health Clinical Center, Shanghai Medical College, Fudan University National Research Center for Translational Medicine (Shanghai), Ruijin Hospital affiliated to Shanghai Jiao Tong University School of Medicine & Shanghai Public Health Clinical Center Shengyue Wang et al

Shangrao/JX1948/2020 EPI_ISL_421240 2020-02-05 Jiangxi Province Center for Disease Control and Prevention Jiangxi Province Center for Disease Control and Prevention JianXiong Li et al

Taiwan/NTU02/2020 EPI_ISL_410218 2020-02-05 Department of Laboratory Medicine, National Taiwan University Hospital Microbial Genomics Core Lab, National Taiwan University Centers of Genomic and Precision Medicine Shiou-Hwei Yeh et al

Guangdong/20SF2546/2020 EPI_ISL_428458 2020-02-06 Guangdong Provincial Center for Diseases Control and Prevention;Guangdong Provincial Institute of Public Health School of Public Health, The University of Hong Kong Bosheng Li et al

Malaysia/MKAK-CL-2020-7554/2020 EPI_ISL_416907 2020-02-06 National Public Health Laboratory Malaysia Genome Institute Mohd Noor Mat Isa et al

Shanghai/SH0025/2020 EPI_ISL_416334 2020-02-06 Shanghai Public Health Clinical Center, Shanghai Medical College, Fudan University National Research Center for Translational Medicine (Shanghai), Ruijin Hospital affiliated to Shanghai Jiao Tong University School of Medicine & Shanghai Public Health Clinical Center Shengyue Wang et al

Shanghai/SH0039/2020 EPI_ISL_416348 2020-02-06 Shanghai Public Health Clinical Center, Shanghai Medical College, Fudan University National Research Center for Translational Medicine (Shanghai), Ruijin Hospital affiliated to Shanghai Jiao Tong University School of Medicine & Shanghai Public Health Clinical Center Shengyue Wang et al

Shanghai/SH0051/2020 EPI_ISL_416359 2020-02-06 Shanghai Public Health Clinical Center, Shanghai Medical College, Fudan University National Research Center for Translational Medicine (Shanghai), Ruijin Hospital affiliated to Shanghai Jiao Tong University School of Medicine & Shanghai Public Health Clinical Center Shengyue Wang et al

Singapore/5/2020 EPI_ISL_410536 2020-02-06 Singapore General Hospital, Molecular Laboratory, Division of Pathology Programme in Emerging Infectious Diseases, Duke-NUS Medical School Danielle E Anderson et al

SouthKorea/KCDC24/2020 EPI_ISL_412873 2020-02-06 Division of Viral Diseases, Center for Laboratory Control of Infectious Diseases, Korea Centers for Diseases Control and Prevention Division of Viral Diseases, Center for Laboratory Control of Infectious Diseases, Korea Centers for Diseases Control and Prevention Jeong-Min Kim et al

SouthKorea/KUMC01/2020 EPI_ISL_413017 2020-02-06 Department of Microbiology, Institute for Viral Diseases, College of Medicine, Korea University Department of Microbiology, Institute for Viral Diseases, College of Medicine, Korea University Changmin Kang et al

SouthKorea/KUMC02/2020 EPI_ISL_413018 2020-02-06 Department of Microbiology, Institute for Viral Diseases, College of Medicine, Korea University Department of Microbiology, Institute for Viral Diseases, College of Medicine, Korea University Changmin Kang et al

USA/CA7/2020 EPI_ISL_411954 2020-02-06 California Department of Public Health Pathogen Discovery, Respiratory Viruses Branch, Division of Viral Diseases, Centers for Disease Control and Prevention Krista Queen et al

Beijing/BJ709/2020 EPI_ISL_430729 2020-02-07 Chinese PLA Institute for Disease Control and Prevention Chinese PLA Institute for Disease Control and Prevention Peng Li et al

Beijing/BJ710/2020 EPI_ISL_430740 2020-02-07 Chinese PLA Institute for Disease Control and Prevention Chinese PLA Institute for Disease Control and Prevention Peng Li et al

Guangdong/GD2020233-P0027/2020 EPI_ISL_413856 2020-02-07 Guangdong Provincial Institution of Public Health, Guangdong Provinical Center for Disease Control and Prevention Guangdong Provincial Institution of Public Health Jing Lu et al

Guangdong/GD2020234-P0023/2020 EPI_ISL_413855 2020-02-07 Guangdong Provincial Institution of Public Health, Guangdong Provinical Center for Disease Control and Prevention Guangdong Provincial Institution of Public Health Jing Lu et al

Guangzhou/GZMU0003/2020 EPI_ISL_429074 2020-02-07 The First Affiliated Hospital of Guangzhou Medical University BGI-shenzhen & The First Affiliated Hospital of Guangzhou Medical University et al

Guangzhou/GZMU0008/2020 EPI_ISL_429075 2020-02-07 The First Affiliated Hospital of Guangzhou Medical University BGI-shenzhen & The First Affiliated Hospital of Guangzhou Medical University et al

Guangzhou/GZMU0009/2020 EPI_ISL_429076 2020-02-07 The First Affiliated Hospital of Guangzhou Medical University BGI-shenzhen & The First Affiliated Hospital of Guangzhou Medical University et al

Guangzhou/GZMU0010/2020 EPI_ISL_429077 2020-02-07 The First Affiliated Hospital of Guangzhou Medical University BGI-shenzhen & The First Affiliated Hospital of Guangzhou Medical University et al

Guangzhou/GZMU0013/2020 EPI_ISL_429078 2020-02-07 The First Affiliated Hospital of Guangzhou Medical University BGI-shenzhen & The First Affiliated Hospital of Guangzhou Medical University et al

HongKong/CUHK1/2020 EPI_ISL_416314 2020-02-07 Department of Microbiology, Faculty of Medicine, The Chinese University of Hong Kong, Hong Kong SAR, China Department of Microbiology, Faculty of Medicine, Chinese University of Hong Kong, Hong Kong SAR, China Zigui Chen et al

Korea/KCDC2009/2020 EPI_ISL_426173 2020-02-07 Division of Viral Diseases, Center for Laboratory Control of Infectious Diseases, Korea Centers for Diseases Control and Prevention Division of Viral Diseases, Center for Laboratory Control of Infectious Diseases, Korea Centers for Diseases Control and Prevention Jeong-Min Kim et al

Shanghai/SH0070/2020 EPI_ISL_416373 2020-02-07 Shanghai Public Health Clinical Center, Shanghai Medical College, Fudan University National Research Center for Translational Medicine (Shanghai), Ruijin Hospital affiliated to Shanghai Jiao Tong University School of Medicine & Shanghai Public Health Clinical Center Shengyue Wang et al

Shanghai/SH0074/2020 EPI_ISL_416377 2020-02-07 Shanghai Public Health Clinical Center, Shanghai Medical College, Fudan University National Research Center for Translational Medicine (Shanghai), Ruijin Hospital affiliated to Shanghai Jiao Tong University School of Medicine & Shanghai Public Health Clinical Center Shengyue Wang et al

Sweden/01/2020 EPI_ISL_411951 2020-02-07 unknown Unit for Laboratory Development and Technology Transfer, Public Health Agency of Sweden Bengner et al

Taiwan/TSGH-01/2020 EPI_ISL_426629 2020-02-07 TSGH-CP molecular lab TSGH-CP molecular lab Cherng-Lih Perng et al

Thailand/SI202205-NT/2020 EPI_ISL_437616 2020-02-07 unknown Faculty of Medicine Rodpan et al

Wuhan/HBCDC-HB-06/2020 EPI_ISL_412982 2020-02-07 Wuhan Lung Hospital Hubei Provincial Center for Disease Control and Prevention Bin Fang et al

Australia/VIC07/2020 EPI_ISL_416415 2020-02-08 Victorian Infectious Diseases Reference Laboratory (VIDRL) Victorian Infectious Diseases Reference Laboratory and Microbiological Diagnostic Unit Public Health Laboratory, Doherty Institute Caly L. et al

Beijing/BJ752/2020 EPI_ISL_430724 2020-02-08 Chinese PLA Institute for Disease Control and Prevention Chinese PLA Institute for Disease Control and Prevention Peng Li et al

England/200690245/2020 EPI_ISL_414041 2020-02-08 Respiratory Virus Unit, Microbiology Services Colindale, Public Health England Respiratory Virus Unit, Microbiology Services Colindale, Public Health England Monica Galiano et al

England/200690300/2020 EPI_ISL_414042 2020-02-08 Respiratory Virus Unit, Microbiology Services Colindale, Public Health England Respiratory Virus Unit, Microbiology Services Colindale, Public Health England Monica Galiano et al

France/RA739/2020 EPI_ISL_410486 2020-02-08 CNR Virus des Infections Respiratoires - France SUD CNR Virus des Infections Respiratoires - France SUD Bal et al

Guangzhou/GZMU0037/2020 EPI_ISL_429089 2020-02-08 The First Affiliated Hospital of Guangzhou Medical University BGI-shenzhen & The First Affiliated Hospital of Guangzhou Medical University et al

Guangzhou/GZMU0039/2020 EPI_ISL_429090 2020-02-08 The First Affiliated Hospital of Guangzhou Medical University BGI-shenzhen & The First Affiliated Hospital of Guangzhou Medical University et al

Guangzhou/GZMU0041/2020 EPI_ISL_429091 2020-02-08 The First Affiliated Hospital of Guangzhou Medical University BGI-shenzhen & The First Affiliated Hospital of Guangzhou Medical University et al

Guangzhou/GZMU0045/2020 EPI_ISL_429092 2020-02-08 The First Affiliated Hospital of Guangzhou Medical University BGI-shenzhen & The First Affiliated Hospital of Guangzhou Medical University et al

Guangzhou/GZMU0046/2020 EPI_ISL_429093 2020-02-08 The First Affiliated Hospital of Guangzhou Medical University BGI-shenzhen & The First Affiliated Hospital of Guangzhou Medical University et al

Guangzhou/GZMU0049/2020 EPI_ISL_429094 2020-02-08 The First Affiliated Hospital of Guangzhou Medical University BGI-shenzhen & The First Affiliated Hospital of Guangzhou Medical University et al

Guangzhou/GZMU0050/2020 EPI_ISL_429095 2020-02-08 The First Affiliated Hospital of Guangzhou Medical University BGI-shenzhen & The First Affiliated Hospital of Guangzhou Medical University et al

HongKong/HKPU29_0102/2020 EPI_ISL_417187 2020-02-08 Department of Clinical Pathology, Pamela Youde Nethersole Eastern Hospital Department of Health Technology and Informatics, Faculty of Health and Social Science, The Hong Kong Polytechnic University Kenneth Siu-Sing LEUNG et al

HongKong/HKPU30_2901/2020 EPI_ISL_417188 2020-02-08 Department of Clinical Pathology, Pamela Youde Nethersole Eastern Hospital Department of Health Technology and Informatics, Faculty of Health and Social Science, The Hong Kong Polytechnic University Kenneth Siu-Sing LEUNG et al

Shanghai/SH0055/2020 EPI_ISL_416363 2020-02-08 Shanghai Public Health Clinical Center, Shanghai Medical College, Fudan University National Research Center for Translational Medicine (Shanghai), Ruijin Hospital affiliated to Shanghai Jiao Tong University School of Medicine & Shanghai Public Health Clinical Center Shengyue Wang et al

Shanghai/SH0056/2020 EPI_ISL_416364 2020-02-08 Shanghai Public Health Clinical Center, Shanghai Medical College, Fudan University National Research Center for Translational Medicine (Shanghai), Ruijin Hospital affiliated to Shanghai Jiao Tong University School of Medicine & Shanghai Public Health Clinical Center Shengyue Wang et al

Shanghai/SH0080/2020 EPI_ISL_416382 2020-02-08 Shanghai Public Health Clinical Center, Shanghai Medical College, Fudan University National Research Center for Translational Medicine (Shanghai), Ruijin Hospital affiliated to Shanghai Jiao Tong University School of Medicine & Shanghai Public Health Clinical Center Shengyue Wang et al

Shangrao/JX1974/2020 EPI_ISL_421258 2020-02-08 Jiangxi Province Center for Disease Control and Prevention Jiangxi Province Center for Disease Control and Prevention JianXiong Li et al

Tianmen/HBCDC-HB-07/2020 EPI_ISL_412983 2020-02-08 Tianmen Center for Disease Control and Prevention Hubei Provincial Center for Disease Control and Prevention Bin Fang et al

UnitedArabEmirates/L4280/2020 EPI_ISL_435134 2020-02-08 Mohammed Bin Rashid University of Medicine and Health Sciences Al Jalila Genomics Center Ahmad Abou Tayoun et al

England/09c/2020 EPI_ISL_412116 2020-02-09 Respiratory Virus Unit, Microbiology Services Colindale, Public Health England Respiratory Virus Unit, Microbiology Services Colindale, Public Health England Monica Galiano et al

Guangdong/GD2020246-P0028/2020 EPI_ISL_413864 2020-02-09 Guangdong Provincial Institution of Public Health, Guangdong Provinical Center for Disease Control and Prevention Guangdong Provincial Institution of Public Health Jing Lu et al

Guangzhou/GZMU0060/2020 EPI_ISL_429103 2020-02-09 The First Affiliated Hospital of Guangzhou Medical University BGI-shenzhen & The First Affiliated Hospital of Guangzhou Medical University et al

HongKong/HKPU28_3001/2020 EPI_ISL_417185 2020-02-09 Department of Pathology, United Christian Hospital Department of Health Technology and Informatics, Faculty of Health and Social Science, The Hong Kong Polytechnic University Kenneth Siu-Sing LEUNG et al

HongKong/HKPU32_0402/2020 EPI_ISL_417193 2020-02-09 Department of Clinical Pathology, Pamela Youde Nethersole Eastern Hospital Department of Health Technology and Informatics, Faculty of Health and Social Science, The Hong Kong Polytechnic University Kenneth Siu-Sing LEUNG et al

HongKong/HKPU34_3001/2020 EPI_ISL_417197 2020-02-09 Department of Clinical Pathology, Pamela Youde Nethersole Eastern Hospital Department of Health Technology and Informatics, Faculty of Health and Social Science, The Hong Kong Polytechnic University Kenneth Siu-Sing LEUNG et al

HongKong/HKPU36-0702/2020 EPI_ISL_418815 2020-02-09 Department of Clinical Pathology, Pamela Youde Nethersole Eastern Hospital Department of Health Technology and Informatics, Faculty of Health and Social Science, The Hong Kong Polytechnic University Kenneth Siu-Sing LEUNG et al

HongKong/VM20002493/2020 EPI_ISL_414527 2020-02-09 Hong Kong Department of Health School of Public Health, The University of Hong Kong Dominic N.C. Tsang et al

Korea/KCDC2012/2020 EPI_ISL_426182 2020-02-09 Division of Viral Diseases, Center for Laboratory Control of Infectious Diseases, Korea Centers for Diseases Control and Prevention Division of Viral Diseases, Center for Laboratory Control of Infectious Diseases, Korea Centers for Diseases Control and Prevention Jeong-Min Kim et al

Shanghai/SH0077/2020 EPI_ISL_416380 2020-02-09 Shanghai Public Health Clinical Center, Shanghai Medical College, Fudan University National Research Center for Translational Medicine (Shanghai), Ruijin Hospital affiliated to Shanghai Jiao Tong University School of Medicine & Shanghai Public Health Clinical Center Shengyue Wang et al

Shanghai/SH0101/2020 EPI_ISL_416393 2020-02-09 Shanghai Public Health Clinical Center, Shanghai Medical College, Fudan University National Research Center for Translational Medicine (Shanghai), Ruijin Hospital affiliated to Shanghai Jiao Tong University School of Medicine & Shanghai Public Health Clinical Center Shengyue Wang et al

Shanghai/SH0119/2020 EPI_ISL_416404 2020-02-09 Shanghai Public Health Clinical Center, Shanghai Medical College, Fudan University National Research Center for Translational Medicine (Shanghai), Ruijin Hospital affiliated to Shanghai Jiao Tong University School of Medicine & Shanghai Public Health Clinical Center Shengyue Wang et al

Singapore/6/2020 EPI_ISL_410537 2020-02-09 Singapore General Hospital, Molecular Laboratory, Division of Pathology Programme in Emerging Infectious Diseases, Duke-NUS Medical School Danielle E Anderson et al

Beijing/BJ782/2020 EPI_ISL_430741 2020-02-10 Chinese PLA Institute for Disease Control and Prevention Chinese PLA Institute for Disease Control and Prevention Peng Li et al

HongKong/HKPU39-3001/2020 EPI_ISL_419214 2020-02-10 Department of Clinical Pathology, Pamela Youde Nethersole Eastern Hospital Department of Health Technology and Informatics, Faculty of Health and Social Science, The Hong Kong Polytechnic University Kenneth Siu-Sing LEUNG et al

HongKong/HKPU41-0802/2020 EPI_ISL_419216 2020-02-10 Department of Clinical Pathology, Pamela Youde Nethersole Eastern Hospital Department of Health Technology and Informatics, Faculty of Health and Social Science, The Hong Kong Polytechnic University Kenneth Siu-Sing LEUNG et al

HongKong/VM20002507/2020 EPI_ISL_414528 2020-02-10 Hong Kong Department of Health School of Public Health, The University of Hong Kong Dominic N.C. Tsang et al

Japan/Hu_DP_Kng_19-020/2020 EPI_ISL_412968 2020-02-10 unknown Takayuki Hishiki Kanagawa Prefectural Institute of Public Health, Department of Microbiology Hishiki et al

Japan/Hu_DP_Kng_19-027/2020 EPI_ISL_412969 2020-02-10 unknown Takayuki Hishiki Kanagawa Prefectural Institute of Public Health, Department of Microbiology Hishiki et al

Shanghai/SH0106/2020 EPI_ISL_416396 2020-02-10 Shanghai Public Health Clinical Center, Shanghai Medical College, Fudan University National Research Center for Translational Medicine (Shanghai), Ruijin Hospital affiliated to Shanghai Jiao Tong University School of Medicine & Shanghai Public Health Clinical Center Shengyue Wang et al

Singapore/17/2020 EPI_ISL_418992 2020-02-10 National Public Health Laboratory, National Centre for Infectious Diseases National Public Health Laboratory, National Centre for Infectious Diseases Mak TM et al

USA/CA8/2020 EPI_ISL_411955 2020-02-10 California Department of Public Health Pathogen Discovery, Respiratory Viruses Branch, Division of Viral Diseases, Centers for Disease Control and Prevention Krista Queen et al

HongKong/HKPU48-0202/2020 EPI_ISL_419222 2020-02-11 Department of Pathology, Princess Margaret Hospital Department of Health Technology and Informatics, Faculty of Health and Social Science, The Hong Kong Polytechnic University Kenneth Siu-Sing LEUNG et al

Shanghai/SH0115/2020 EPI_ISL_416402 2020-02-11 Shanghai Public Health Clinical Center, Shanghai Medical College, Fudan University National Research Center for Translational Medicine (Shanghai), Ruijin Hospital affiliated to Shanghai Jiao Tong University School of Medicine & Shanghai Public Health Clinical Center Shengyue Wang et al

USA/TX1/2020 EPI_ISL_411956 2020-02-11 Texas Department of State Health Services Pathogen Discovery, Respiratory Viruses Branch, Division of Viral Diseases, Centers for Disease Control and Prevention Krista Queen et al

Vietnam/CM99/2020 EPI_ISL_416429 2020-02-11 National Influenza Center, National Institute of Hygiene and Epidemiology (NIHE) National Influenza Center, National Institute of Hygiene and Epidemiology (NIHE) Le Quynh Mai et al

HongKong/VM20002582/2020 EPI_ISL_414569 2020-02-12 Hong Kong Department of Health School of Public Health, The University of Hong Kong Dominic N.C. Tsang et al

Korea/KCDC2014/2020 EPI_ISL_426187 2020-02-12 Division of Viral Diseases, Center for Laboratory Control of Infectious Diseases, Korea Centers for Diseases Control and Prevention Division of Viral Diseases, Center for Laboratory Control of Infectious Diseases, Korea Centers for Diseases Control and Prevention Jeong-Min Kim et al

Malaysia/IMR_WC627/2020 EPI_ISL_430444 2020-02-12 Institute for Medical Research, Infectious Disease Research Centre, National Institutes of Health, Ministry of Health Malaysia Institute for Medical Research, Infectious Disease Research Centre, National Institutes of Health, Ministry of Health Malaysia Suppiah.J et al

HongKong/HKPU52-3101/2020 EPI_ISL_419224 2020-02-13 Department of Clinical Pathology, Pamela Youde Nethersole Eastern Hospital Department of Health Technology and Informatics, Faculty of Health and Social Science, The Hong Kong Polytechnic University Kenneth Siu-Sing LEUNG et al

Shanghai/SH0110/2020 EPI_ISL_416399 2020-02-13 Shanghai Public Health Clinical Center, Shanghai Medical College, Fudan University National Research Center for Translational Medicine (Shanghai), Ruijin Hospital affiliated to Shanghai Jiao Tong University School of Medicine & Shanghai Public Health Clinical Center Shengyue Wang et al

Singapore/14/2020 EPI_ISL_414380 2020-02-13 National Centre for Infectious Diseases Programme in Emerging Infectious Diseases, Duke-NUS Medical School Danielle E Anderson et al

Singapore/21/2020 EPI_ISL_419000 2020-02-13 National Public Health Laboratory, National Centre for Infectious Diseases National Public Health Laboratory, National Centre for Infectious Diseases Mak TM et al

Japan/Hu_DP_Kng_19-031/2020 EPI_ISL_420889 2020-02-14 unknown Takayuki Hishiki Kanagawa Prefectural Institute of Public Health Hishiki et al

Shanghai/SH0102/2020 EPI_ISL_416394 2020-02-14 Shanghai Public Health Clinical Center, Shanghai Medical College, Fudan University National Research Center for Translational Medicine (Shanghai), Ruijin Hospital affiliated to Shanghai Jiao Tong University School of Medicine & Shanghai Public Health Clinical Center Shengyue Wang et al

Singapore/14Clin/2020 EPI_ISL_418998 2020-02-14 National Public Health Laboratory, National Centre for Infectious Diseases National Public Health Laboratory, National Centre for Infectious Diseases Mak TM et al

Japan/DP0005/2020 EPI_ISL_416565 2020-02-15 Japanese Quarantine Stations Pathogen Genomics Center, National Institute of Infectious Diseases Tsuyoshi Sekizuka et al

Japan/DP0027/2020 EPI_ISL_416566 2020-02-15 Japanese Quarantine Stations Pathogen Genomics Center, National Institute of Infectious Diseases Tsuyoshi Sekizuka et al

Japan/DP0037/2020 EPI_ISL_416567 2020-02-15 Japanese Quarantine Stations Pathogen Genomics Center, National Institute of Infectious Diseases Tsuyoshi Sekizuka et al

Japan/DP0059/2020 EPI_ISL_416569 2020-02-15 Japanese Quarantine Stations Pathogen Genomics Center, National Institute of Infectious Diseases Tsuyoshi Sekizuka et al

Japan/DP0065/2020 EPI_ISL_416570 2020-02-15 Japanese Quarantine Stations Pathogen Genomics Center, National Institute of Infectious Diseases Tsuyoshi Sekizuka et al

Japan/DP0077/2020 EPI_ISL_416571 2020-02-15 Japanese Quarantine Stations Pathogen Genomics Center, National Institute of Infectious Diseases Tsuyoshi Sekizuka et al

Japan/DP0078/2020 EPI_ISL_416572 2020-02-15 Japanese Quarantine Stations Pathogen Genomics Center, National Institute of Infectious Diseases Tsuyoshi Sekizuka et al

Japan/DP0104/2020 EPI_ISL_416573 2020-02-15 Japanese Quarantine Stations Pathogen Genomics Center, National Institute of Infectious Diseases Tsuyoshi Sekizuka et al

Japan/DP0107/2020 EPI_ISL_416574 2020-02-15 Japanese Quarantine Stations Pathogen Genomics Center, National Institute of Infectious Diseases Tsuyoshi Sekizuka et al

Japan/DP0121/2020 EPI_ISL_416575 2020-02-15 Japanese Quarantine Stations Pathogen Genomics Center, National Institute of Infectious Diseases Tsuyoshi Sekizuka et al

Japan/DP0133/2020 EPI_ISL_416576 2020-02-15 Japanese Quarantine Stations Pathogen Genomics Center, National Institute of Infectious Diseases Tsuyoshi Sekizuka et al

Japan/DP0134/2020 EPI_ISL_416577 2020-02-15 Japanese Quarantine Stations Pathogen Genomics Center, National Institute of Infectious Diseases Tsuyoshi Sekizuka et al

Japan/DP0152/2020 EPI_ISL_416578 2020-02-15 Japanese Quarantine Stations Pathogen Genomics Center, National Institute of Infectious Diseases Tsuyoshi Sekizuka et al

Japan/DP0158/2020 EPI_ISL_416579 2020-02-15 Japanese Quarantine Stations Pathogen Genomics Center, National Institute of Infectious Diseases Tsuyoshi Sekizuka et al

Japan/DP0184/2020 EPI_ISL_416580 2020-02-15 Japanese Quarantine Stations Pathogen Genomics Center, National Institute of Infectious Diseases Tsuyoshi Sekizuka et al

Japan/DP0190/2020 EPI_ISL_416581 2020-02-15 Japanese Quarantine Stations Pathogen Genomics Center, National Institute of Infectious Diseases Tsuyoshi Sekizuka et al

Japan/DP0191/2020 EPI_ISL_416582 2020-02-15 Japanese Quarantine Stations Pathogen Genomics Center, National Institute of Infectious Diseases Tsuyoshi Sekizuka et al

Japan/DP0196/2020 EPI_ISL_416583 2020-02-15 Japanese Quarantine Stations Pathogen Genomics Center, National Institute of Infectious Diseases Tsuyoshi Sekizuka et al

Japan/DP0200/2020 EPI_ISL_416584 2020-02-15 Japanese Quarantine Stations Pathogen Genomics Center, National Institute of Infectious Diseases Tsuyoshi Sekizuka et al

Shanghai/SH0125/2020 EPI_ISL_416406 2020-02-15 Shanghai Public Health Clinical Center, Shanghai Medical College, Fudan University National Research Center for Translational Medicine (Shanghai), Ruijin Hospital affiliated to Shanghai Jiao Tong University School of Medicine & Shanghai Public Health Clinical Center Shengyue Wang et al

Shanghai/SH0126/2020 EPI_ISL_416407 2020-02-15 Shanghai Public Health Clinical Center, Shanghai Medical College, Fudan University National Research Center for Translational Medicine (Shanghai), Ruijin Hospital affiliated to Shanghai Jiao Tong University School of Medicine & Shanghai Public Health Clinical Center Shengyue Wang et al

Japan/DP0236/2020 EPI_ISL_416585 2020-02-16 Japanese Quarantine Stations Pathogen Genomics Center, National Institute of Infectious Diseases Tsuyoshi Sekizuka et al

Japan/DP0274/2020 EPI_ISL_416586 2020-02-16 Japanese Quarantine Stations Pathogen Genomics Center, National Institute of Infectious Diseases Tsuyoshi Sekizuka et al

Japan/DP0278/2020 EPI_ISL_416587 2020-02-16 Japanese Quarantine Stations Pathogen Genomics Center, National Institute of Infectious Diseases Tsuyoshi Sekizuka et al

Japan/DP0287/2020 EPI_ISL_416589 2020-02-16 Japanese Quarantine Stations Pathogen Genomics Center, National Institute of Infectious Diseases Tsuyoshi Sekizuka et al

Japan/DP0289/2020 EPI_ISL_416590 2020-02-16 Japanese Quarantine Stations Pathogen Genomics Center, National Institute of Infectious Diseases Tsuyoshi Sekizuka et al

Japan/DP0290/2020 EPI_ISL_416591 2020-02-16 Japanese Quarantine Stations Pathogen Genomics Center, National Institute of Infectious Diseases Tsuyoshi Sekizuka et al

Japan/DP0294/2020 EPI_ISL_416592 2020-02-16 Japanese Quarantine Stations Pathogen Genomics Center, National Institute of Infectious Diseases Tsuyoshi Sekizuka et al

Japan/DP0311/2020 EPI_ISL_416593 2020-02-16 Japanese Quarantine Stations Pathogen Genomics Center, National Institute of Infectious Diseases Tsuyoshi Sekizuka et al

Japan/DP0319/2020 EPI_ISL_416594 2020-02-16 Japanese Quarantine Stations Pathogen Genomics Center, National Institute of Infectious Diseases Tsuyoshi Sekizuka et al

Japan/DP0328/2020 EPI_ISL_416595 2020-02-16 Japanese Quarantine Stations Pathogen Genomics Center, National Institute of Infectious Diseases Tsuyoshi Sekizuka et al

Japan/DP0344/2020 EPI_ISL_416596 2020-02-16 Japanese Quarantine Stations Pathogen Genomics Center, National Institute of Infectious Diseases Tsuyoshi Sekizuka et al

Japan/DP0346/2020 EPI_ISL_416597 2020-02-16 Japanese Quarantine Stations Pathogen Genomics Center, National Institute of Infectious Diseases Tsuyoshi Sekizuka et al

Japan/DP0357/2020 EPI_ISL_416598 2020-02-16 Japanese Quarantine Stations Pathogen Genomics Center, National Institute of Infectious Diseases Tsuyoshi Sekizuka et al

Japan/DP0361/2020 EPI_ISL_416599 2020-02-16 Japanese Quarantine Stations Pathogen Genomics Center, National Institute of Infectious Diseases Tsuyoshi Sekizuka et al

Japan/DP0438/2020 EPI_ISL_416600 2020-02-16 Japanese Quarantine Stations Pathogen Genomics Center, National Institute of Infectious Diseases Tsuyoshi Sekizuka et al

Japan/DP0457/2020 EPI_ISL_416601 2020-02-16 Japanese Quarantine Stations Pathogen Genomics Center, National Institute of Infectious Diseases Tsuyoshi Sekizuka et al

Japan/DP0464/2020 EPI_ISL_416603 2020-02-16 Japanese Quarantine Stations Pathogen Genomics Center, National Institute of Infectious Diseases Tsuyoshi Sekizuka et al

Japan/DP0476/2020 EPI_ISL_416604 2020-02-16 Japanese Quarantine Stations Pathogen Genomics Center, National Institute of Infectious Diseases Tsuyoshi Sekizuka et al

Japan/DP0481/2020 EPI_ISL_416605 2020-02-16 Japanese Quarantine Stations Pathogen Genomics Center, National Institute of Infectious Diseases Tsuyoshi Sekizuka et al

Japan/DP0482/2020 EPI_ISL_416606 2020-02-16 Japanese Quarantine Stations Pathogen Genomics Center, National Institute of Infectious Diseases Tsuyoshi Sekizuka et al

Singapore/36/2020 EPI_ISL_422429 2020-02-16 National Public Health Laboratory, National Centre for Infectious Diseases National Public Health Laboratory, National Centre for Infectious Diseases Mak TM et al

Guangdong/20SF4047/2020 EPI_ISL_428462 2020-02-17 Guangdong Provincial Center for Diseases Control and Prevention;Guangdong Provincial Institute of Public Health School of Public Health, The University of Hong Kong Bosheng Li et al

Guangdong/20SF4051/2020 EPI_ISL_428463 2020-02-17 Guangdong Provincial Center for Diseases Control and Prevention;Guangdong Provincial Institute of Public Health School of Public Health, The University of Hong Kong Bosheng Li et al

Japan/DP0543/2020 EPI_ISL_416607 2020-02-17 Japanese Quarantine Stations Pathogen Genomics Center, National Institute of Infectious Diseases Tsuyoshi Sekizuka et al

Japan/DP0544/2020 EPI_ISL_416608 2020-02-17 Japanese Quarantine Stations Pathogen Genomics Center, National Institute of Infectious Diseases Tsuyoshi Sekizuka et al

Japan/DP0568/2020 EPI_ISL_416609 2020-02-17 Japanese Quarantine Stations Pathogen Genomics Center, National Institute of Infectious Diseases Tsuyoshi Sekizuka et al

Japan/DP0588/2020 EPI_ISL_416610 2020-02-17 Japanese Quarantine Stations Pathogen Genomics Center, National Institute of Infectious Diseases Tsuyoshi Sekizuka et al

Japan/DP0644/2020 EPI_ISL_416611 2020-02-17 Japanese Quarantine Stations Pathogen Genomics Center, National Institute of Infectious Diseases Tsuyoshi Sekizuka et al

Japan/DP0645/2020 EPI_ISL_416612 2020-02-17 Japanese Quarantine Stations Pathogen Genomics Center, National Institute of Infectious Diseases Tsuyoshi Sekizuka et al

Japan/DP0654/2020 EPI_ISL_416613 2020-02-17 Japanese Quarantine Stations Pathogen Genomics Center, National Institute of Infectious Diseases Tsuyoshi Sekizuka et al

Japan/DP0687/2020 EPI_ISL_416614 2020-02-17 Japanese Quarantine Stations Pathogen Genomics Center, National Institute of Infectious Diseases Tsuyoshi Sekizuka et al

Japan/DP0699/2020 EPI_ISL_416617 2020-02-17 Japanese Quarantine Stations Pathogen Genomics Center, National Institute of Infectious Diseases Tsuyoshi Sekizuka et al

Japan/DP0703/2020 EPI_ISL_416619 2020-02-17 Japanese Quarantine Stations Pathogen Genomics Center, National Institute of Infectious Diseases Tsuyoshi Sekizuka et al

Japan/DP0724/2020 EPI_ISL_416620 2020-02-17 Japanese Quarantine Stations Pathogen Genomics Center, National Institute of Infectious Diseases Tsuyoshi Sekizuka et al

Japan/DP0743/2020 EPI_ISL_416621 2020-02-17 Japanese Quarantine Stations Pathogen Genomics Center, National Institute of Infectious Diseases Tsuyoshi Sekizuka et al

Japan/DP0752/2020 EPI_ISL_416622 2020-02-17 Japanese Quarantine Stations Pathogen Genomics Center, National Institute of Infectious Diseases Tsuyoshi Sekizuka et al

Japan/DP0764/2020 EPI_ISL_416624 2020-02-17 Japanese Quarantine Stations Pathogen Genomics Center, National Institute of Infectious Diseases Tsuyoshi Sekizuka et al

Japan/DP0765/2020 EPI_ISL_416625 2020-02-17 Japanese Quarantine Stations Pathogen Genomics Center, National Institute of Infectious Diseases Tsuyoshi Sekizuka et al

Japan/DP0785/2020 EPI_ISL_416627 2020-02-17 Japanese Quarantine Stations Pathogen Genomics Center, National Institute of Infectious Diseases Tsuyoshi Sekizuka et al

Japan/DP0786/2020 EPI_ISL_416628 2020-02-17 Japanese Quarantine Stations Pathogen Genomics Center, National Institute of Infectious Diseases Tsuyoshi Sekizuka et al

Japan/DP0802/2020 EPI_ISL_416629 2020-02-17 Japanese Quarantine Stations Pathogen Genomics Center, National Institute of Infectious Diseases Tsuyoshi Sekizuka et al

Japan/DP0803/2020 EPI_ISL_416630 2020-02-17 Japanese Quarantine Stations Pathogen Genomics Center, National Institute of Infectious Diseases Tsuyoshi Sekizuka et al

Japan/DP0804/2020 EPI_ISL_416631 2020-02-17 Japanese Quarantine Stations Pathogen Genomics Center, National Institute of Infectious Diseases Tsuyoshi Sekizuka et al

Japan/DP0827/2020 EPI_ISL_416632 2020-02-17 Japanese Quarantine Stations Pathogen Genomics Center, National Institute of Infectious Diseases Tsuyoshi Sekizuka et al

Japan/DP0880/2020 EPI_ISL_416633 2020-02-17 Japanese Quarantine Stations Pathogen Genomics Center, National Institute of Infectious Diseases Tsuyoshi Sekizuka et al

Japan/DP0890/2020 EPI_ISL_416634 2020-02-17 Japanese Quarantine Stations Pathogen Genomics Center, National Institute of Infectious Diseases Tsuyoshi Sekizuka et al

Singapore/12/2020 EPI_ISL_414378 2020-02-17 National Centre for Infectious Diseases Programme in Emerging Infectious Diseases, Duke-NUS Medical School Danielle E Anderson et al

USA/CruiseA-1/2020 EPI_ISL_413606 2020-02-17 unknown Pathogen Discovery, Respiratory Viruses Branch, Division of Viral Diseases, Centers for Disease Control and Prevention Anna Uehara et al

USA/CruiseA-10/2020 EPI_ISL_413615 2020-02-17 unknown Pathogen Discovery, Respiratory Viruses Branch, Division of Viral Diseases, Centers for Disease Control and Prevention Ying Tao et al

USA/CruiseA-11/2020 EPI_ISL_413616 2020-02-17 unknown Pathogen Discovery, Respiratory Viruses Branch, Division of Viral Diseases, Centers for Disease Control and Prevention Ying Tao et al

USA/CruiseA-25/2020 EPI_ISL_414484 2020-02-17 unknown Pathogen Discovery, Respiratory Viruses Branch, Division of Viral Diseases, Centers for Disease Control and Prevention Krista Queen et al

USA/CruiseA-7/2020 EPI_ISL_413612 2020-02-17 unknown Pathogen Discovery, Respiratory Viruses Branch, Division of Viral Diseases, Centers for Disease Control and Prevention Ying Tao et al

USA/CruiseA-8/2020 EPI_ISL_413613 2020-02-17 unknown Pathogen Discovery, Respiratory Viruses Branch, Division of Viral Diseases, Centers for Disease Control and Prevention Ying Tao et al

USA/CruiseA-9/2020 EPI_ISL_413614 2020-02-17 unknown Pathogen Discovery, Respiratory Viruses Branch, Division of Viral Diseases, Centers for Disease Control and Prevention Ying Tao et al

Korea/KCDC2016/2020 EPI_ISL_427810 2020-02-18 Division of Viral Diseases, Center for Laboratory Control of Infectious Diseases, Korea Centers for Diseases Control and Prevention Division of Viral Diseases, Center for Laboratory Control of Infectious Diseases, Korea Centers for Diseases Control and Prevention Jeong-Min Kim et al

Korea/KCDC2017/2020 EPI_ISL_427811 2020-02-18 Division of Viral Diseases, Center for Laboratory Control of Infectious Diseases, Korea Centers for Diseases Control and Prevention Division of Viral Diseases, Center for Laboratory Control of Infectious Diseases, Korea Centers for Diseases Control and Prevention Jeong-Min Kim et al

Korea/KCDC2019/2020 EPI_ISL_427813 2020-02-18 Division of Viral Diseases, Center for Laboratory Control of Infectious Diseases, Korea Centers for Diseases Control and Prevention Division of Viral Diseases, Center for Laboratory Control of Infectious Diseases, Korea Centers for Diseases Control and Prevention Jeong-Min Kim et al

Singapore/13/2020 EPI_ISL_414379 2020-02-18 National Centre for Infectious Diseases Programme in Emerging Infectious Diseases, Duke-NUS Medical School Danielle E Anderson et al

USA/CruiseA-15/2020 EPI_ISL_413620 2020-02-18 unknown Pathogen Discovery, Respiratory Viruses Branch, Division of Viral Diseases, Centers for Disease Control and Prevention Clinton R. Paden et al

USA/CruiseA-16/2020 EPI_ISL_413621 2020-02-18 unknown Pathogen Discovery, Respiratory Viruses Branch, Division of Viral Diseases, Centers for Disease Control and Prevention Clinton R. Paden et al

USA/CruiseA-19/2020 EPI_ISL_414479 2020-02-18 unknown Pathogen Discovery, Respiratory Viruses Branch, Division of Viral Diseases, Centers for Disease Control and Prevention Ying Tao et al

USA/CruiseA-2/2020 EPI_ISL_413607 2020-02-18 unknown Pathogen Discovery, Respiratory Viruses Branch, Division of Viral Diseases, Centers for Disease Control and Prevention Anna Uehara et al

USA/CruiseA-23/2020 EPI_ISL_414482 2020-02-18 unknown Pathogen Discovery, Respiratory Viruses Branch, Division of Viral Diseases, Centers for Disease Control and Prevention Krista Queen et al

USA/CruiseA-3/2020 EPI_ISL_413608 2020-02-18 unknown Pathogen Discovery, Respiratory Viruses Branch, Division of Viral Diseases, Centers for Disease Control and Prevention Anna Uehara et al

HongKong/HKPU63-1402/2020 EPI_ISL_419231 2020-02-19 Department of Clinical Pathology, Tuen Mun Hospital, 23 Tsing Chung Koon Road, Tuen Mun, N.T. Department of Health Technology and Informatics, Faculty of Health and Social Science, The Hong Kong Polytechnic University Kenneth Siu-Sing LEUNG et al

HongKong/HKPU64-1202/2020 EPI_ISL_419232 2020-02-19 Department of Clinical Pathology, Pamela Youde Nethersole Eastern Hospital Department of Health Technology and Informatics, Faculty of Health and Social Science, The Hong Kong Polytechnic University Kenneth Siu-Sing LEUNG et al

Korea/KCDC2018/2020 EPI_ISL_427812 2020-02-19 Division of Viral Diseases, Center for Laboratory Control of Infectious Diseases, Korea Centers for Diseases Control and Prevention Division of Viral Diseases, Center for Laboratory Control of Infectious Diseases, Korea Centers for Diseases Control and Prevention Jeong-Min Kim et al

Canada/ON_PHL5472/2020 EPI_ISL_418325 2020-02-20 Public Health Ontario Laboratories Public Health Ontario Laboratories Alireza Eshaghi et al

Italy/CDG1/2020 EPI_ISL_412973 2020-02-20 Department of Infectious Diseases, Istituto Superiore di Sanit√†, Roma , Italy Virology Laboratory, Scientific Department, Army Medical Center Paola Stefanelli et al

Japan/TK-20-31-3/2020 EPI_ISL_413459 2020-02-20 Department of Pathology, Toshima Hospital Pathogen Genomics Center, National Institute of Infectious Diseases Tsuyoshi Sekizuka et al

USA/CruiseA-12/2020 EPI_ISL_413617 2020-02-20 unknown Pathogen Discovery, Respiratory Viruses Branch, Division of Viral Diseases, Centers for Disease Control and Prevention Ying Tao et al

USA/CruiseA-13/2020 EPI_ISL_413618 2020-02-20 unknown Pathogen Discovery, Respiratory Viruses Branch, Division of Viral Diseases, Centers for Disease Control and Prevention Clinton R. Paden et al

USA/WA-S2/2020 EPI_ISL_413456 2020-02-20 Seattle Flu Study Seattle Flu Study Chu et al et al

Australia/NT01/2020 EPI_ISL_419831 2020-02-21 Royal Darwin Hospital Victorian Infectious Diseases Reference Laboratory and Microbiological Diagnostic Unit Public Health Laboratory, Doherty Institute Meumann et al

Australia/NT02/2020 EPI_ISL_419832 2020-02-21 Royal Darwin Hospital Victorian Infectious Diseases Reference Laboratory and Microbiological Diagnostic Unit Public Health Laboratory, Doherty Institute Meumann et al

France/HF1463/2020 EPI_ISL_429968 2020-02-21 Centre Hospitalier Compi√®gne Laboratoire de Biologie National Reference Center for Viruses of Respiratory Infections, Institut Pasteur, Paris M√©lanie Albert et al

France/HF1465/2020 EPI_ISL_418218 2020-02-21 Centre Hospitalier Compi√®gne Laboratoire de Biologie National Reference Center for Viruses of Respiratory Infections, Institut Pasteur, Paris M√©lanie Albert et al

USA/CruiseA-21/2020 EPI_ISL_414480 2020-02-21 unknown Pathogen Discovery, Respiratory Viruses Branch, Division of Viral Diseases, Centers for Disease Control and Prevention Ying Tao et al

USA/CruiseA-22/2020 EPI_ISL_414481 2020-02-21 unknown Pathogen Discovery, Respiratory Viruses Branch, Division of Viral Diseases, Centers for Disease Control and Prevention Ying Tao et al

USA/CruiseA-4/2020 EPI_ISL_413609 2020-02-21 unknown Pathogen Discovery, Respiratory Viruses Branch, Division of Viral Diseases, Centers for Disease Control and Prevention Anna Uehara et al

USA/CruiseA-5/2020 EPI_ISL_413610 2020-02-21 unknown Pathogen Discovery, Respiratory Viruses Branch, Division of Viral Diseases, Centers for Disease Control and Prevention Anna Uehara et al

USA/CruiseA-6/2020 EPI_ISL_413611 2020-02-21 unknown Pathogen Discovery, Respiratory Viruses Branch, Division of Viral Diseases, Centers for Disease Control and Prevention Anna Uehara et al

USA/WA-S84/2020 EPI_ISL_417137 2020-02-21 Washington State Department of Health Seattle Flu Study Chu etl al et al

Australia/WA11/202 EPI_ISL_420456 2020-02-22 PathWest Laboratory Medicine WA PathWest Laboratory Medicine WA Chisha Sikazwe et al

HongKong/HKPU74-1302/2020 EPI_ISL_419245 2020-02-22 Department of Clinical Pathology, Pamela Youde Nethersole Eastern Hospital Department of Health Technology and Informatics, Faculty of Health and Social Science, The Hong Kong Polytechnic University Kenneth Siu-Sing LEUNG et al

HongKong/VM20002849/2020 EPI_ISL_414571 2020-02-22 Hong Kong Department of Health School of Public Health, The University of Hong Kong Dominic N.C. Tsang et al

Thailand/SI202620-NT/2020 EPI_ISL_437615 2020-02-22 unknown Faculty of Medicine Rodpan et al

USA/WA-S277/2020 EPI_ISL_430131 2020-02-22 Seattle Flu Study Seattle Flu Study Chu et al et al

USA/WA-S82/2020 EPI_ISL_417135 2020-02-22 Washington State Department of Health Seattle Flu Study Chu etl al et al

Australia/VIC138/2020 EPI_ISL_419834 2020-02-23 Victorian Infectious Diseases Reference Laboratory (VIDRL) Victorian Infectious Diseases Reference Laboratory and Microbiological Diagnostic Unit Public Health Laboratory, Doherty Institute Caly L. et al

Hefei/2/2020 EPI_ISL_412026 2020-02-23 Second Hospital of Anhui Medical University Second Hospital of Anhui Medical University Changtai Wang et al

Israel/ISR_JP0320/2020 EPI_ISL_419211 2020-02-23 Central Virology Laboratory Israel Institute for Biological Research Inbar Cohen-Gihon et al

Korea/KCDC2001/2020 EPI_ISL_425117 2020-02-23 Division of Viral Diseases, Center for Laboratory Control of Infectious Diseases, Korea Centers for Diseases Control and Prevention Division of Viral Diseases, Center for Laboratory Control of Infectious Diseases, Korea Centers for Diseases Control and Prevention Jeong-Min Kim et al

Korea/KCDC2003/2020 EPI_ISL_426163 2020-02-23 Division of Viral Diseases, Center for Laboratory Control of Infectious Diseases, Korea Centers for Diseases Control and Prevention Division of Viral Diseases, Center for Laboratory Control of Infectious Diseases, Korea Centers for Diseases Control and Prevention Jeong-Min Kim et al

Korea/KCDC2011/2020 EPI_ISL_426181 2020-02-23 Division of Viral Diseases, Center for Laboratory Control of Infectious Diseases, Korea Centers for Diseases Control and Prevention Division of Viral Diseases, Center for Laboratory Control of Infectious Diseases, Korea Centers for Diseases Control and Prevention Jeong-Min Kim et al

Korea/KCDC2013/2020 EPI_ISL_426183 2020-02-23 Division of Viral Diseases, Center for Laboratory Control of Infectious Diseases, Korea Centers for Diseases Control and Prevention Division of Viral Diseases, Center for Laboratory Control of Infectious Diseases, Korea Centers for Diseases Control and Prevention Jeong-Min Kim et al

USA/CA9/2020 EPI_ISL_412862 2020-02-23 California Department of Public Health Pathogen Discovery, Respiratory Viruses Branch, Division of Viral Diseases, Centers for Disease Control and Prevention Krista Queen et al

Australia/NT05/2020 EPI_ISL_419835 2020-02-24 Royal Darwin Hospital Victorian Infectious Diseases Reference Laboratory and Microbiological Diagnostic Unit Public Health Laboratory, Doherty Institute Meumann et al

HongKong/HKPU83-1702/2020 EPI_ISL_419247 2020-02-24 Department of Clinical Pathology, Pamela Youde Nethersole Eastern Hospital Department of Health Technology and Informatics, Faculty of Health and Social Science, The Hong Kong Polytechnic University Kenneth Siu-Sing LEUNG et al

HongKong/VM20002868/2020 EPI_ISL_414519 2020-02-24 Hong Kong Department of Health School of Public Health, The University of Hong Kong Dominic N.C. Tsang et al

Italy/UniMI03/2020 EPI_ISL_417447 2020-02-24 Laboratory of Infectious Diseases, Department of Biomedical and Clinical Sciences L. Sacco, University of Milan Laboratory of Infectious Diseases, Department of Biomedical and Clinical Sciences L. Sacco, University of Milan Gianguglielmo Zehender et al

Switzerland/TI9486/2020 EPI_ISL_413996 2020-02-24 Laboratoire de Virologie, HUG Swiss National Reference Centre for Influenza LAUBSCHER Florian et al. et al

USA/CruiseA-17/2020 EPI_ISL_413622 2020-02-24 unknown Pathogen Discovery, Respiratory Viruses Branch, Division of Viral Diseases, Centers for Disease Control and Prevention Clinton R. Paden et al

USA/CruiseA-26/2020 EPI_ISL_414485 2020-02-24 unknown Pathogen Discovery, Respiratory Viruses Branch, Division of Viral Diseases, Centers for Disease Control and Prevention Krista Queen et al

USA/WA-S254/2020 EPI_ISL_430292 2020-02-24 Washington State Department of Health Seattle Flu Study Chu et al et al

USA/WA-S7/2020 EPI_ISL_416462 2020-02-24 Seattle Flu Study Seattle Flu Study Chu et al et al

USA/WA2/2020 EPI_ISL_412970 2020-02-24 Washington State Department of Health Seattle Flu Study Helen Chu et al

Beijing/BJ1112/2020 EPI_ISL_430737 2020-02-25 Chinese PLA Institute for Disease Control and Prevention Chinese PLA Institute for Disease Control and Prevention Peng Li et al

Brazil/SPBR-01/2020 EPI_ISL_412964 2020-02-25 Hospital Israelita Albert Einstein Instituto Adolfo Lutz Interdisciplinary Procedures Center Strategic Laboratory Jaqueline Goes de Jesus et al

England/200940527/2020 EPI_ISL_414005 2020-02-25 Respiratory Virus Unit, Microbiology Services Colindale, Public Health England Respiratory Virus Unit, Microbiology Services Colindale, Public Health England Monica Galiano et al

England/200960515/2020 EPI_ISL_414009 2020-02-25 Respiratory Virus Unit, Microbiology Services Colindale, Public Health England Respiratory Virus Unit, Microbiology Services Colindale, Public Health England Monica Galiano et al

France/GE1583/2020 EPI_ISL_414623 2020-02-25 Laboratoire de Virologie Institut de Virologie - INSERM U 1109 H√¥pitaux Universitaires de Strasbourg National Reference Center for Viruses of Respiratory Infections, Institut Pasteur, Paris M√©lnie Albert et al

Germany/Baden-Wuerttemberg-1/2020 EPI_ISL_412912 2020-02-25 State Health Office Baden-Wuerttemberg Charit√© Universit√§tsmedizin Berlin, Institute of Virology Victor M Corman et al

Germany/NRW-02-1/2020 EPI_ISL_414497 2020-02-25 Center of Medical Microbiology, Virology, and Hospital Hygiene, University of Duesseldorf Center of Medical Microbiology, Virology, and Hospital Hygiene, University of Duesseldorf Ortwin Adams et al

Guangzhou/GZMU0014/2020 EPI_ISL_414692 2020-02-25 State Key Laboratory of Respiratory Disease, National Clinical Research Center for Respiratory Disease, Guangzhou Institute of Respiratory Health, the First Affiliated Hospital of Guangzhou Medical University The First Affiliated Hospital of Guangzhou Medical University & BGI-Shenzhen Zhao et al et al

Guangzhou/GZMU0016/2020 EPI_ISL_414663 2020-02-25 State Key Laboratory of Respiratory Disease, National Clinical Research Center for Respiratory Disease, Guangzhou Institute of Respiratory Health, the First Affiliated Hospital of Guangzhou Medical University the First Affiliated Hospital of Guangzhou Medical University & BGI-Shenzhen Zhao et al et al

Guangzhou/GZMU0042/2020 EPI_ISL_414688 2020-02-25 State Key Laboratory of Respiratory Disease, National Clinical Research Center for Respiratory Disease, Guangzhou Institute of Respiratory Health, the First Affiliated Hospital of Guangzhou Medical University The First Affiliated Hospital of Guangzhou Medical University & BGI-Shenzhen Zhao et al et al

Guangzhou/GZMU0044/2020 EPI_ISL_414689 2020-02-25 State Key Laboratory of Respiratory Disease, National Clinical Research Center for Respiratory Disease, Guangzhou Institute of Respiratory Health, the First Affiliated Hospital of Guangzhou Medical University The First Affiliated Hospital of Guangzhou Medical University & BGI-Shenzhen Zhao et al et al

Guangzhou/GZMU0047/2020 EPI_ISL_414690 2020-02-25 State Key Laboratory of Respiratory Disease, National Clinical Research Center for Respiratory Disease, Guangzhou Institute of Respiratory Health, the First Affiliated Hospital of Guangzhou Medical University The First Affiliated Hospital of Guangzhou Medical University & BGI-Shenzhen Zhao et al et al

Guangzhou/GZMU0048/2020 EPI_ISL_414691 2020-02-25 State Key Laboratory of Respiratory Disease, National Clinical Research Center for Respiratory Disease, Guangzhou Institute of Respiratory Health, the First Affiliated Hospital of Guangzhou Medical University The First Affiliated Hospital of Guangzhou Medical University & BGI-Shenzhen Zhao et al et al

HongKong/VM20002907/2020 EPI_ISL_414517 2020-02-25 Hong Kong Department of Health School of Public Health, The University of Hong Kong Dominic N.C. Tsang et al

Spain/Madrid201105/2020 EPI_ISL_418251 2020-02-25 HOSPITAL UNIVERSITARIO LA PAZ Instituto de Salud Carlos III Iglesias-Caballero et al

Taiwan/TSGH-26/2020 EPI_ISL_447252 2020-02-25 TSGH-CP molecular lab TSGH-CP molecular lab Cherng-Lih Perng et al

Thailand/Bangkok_1716/2020 EPI_ISL_447920 2020-02-25 n/a National Institute of Health. Department of medical Sciences, Ministry of Public Health, Thailand Pilailuk et al

Thailand/SI202769-NT/2020 EPI_ISL_437614 2020-02-25 unknown Faculty of Medicine Rodpan et al

Thailand/SI202872-NT/2020 EPI_ISL_437613 2020-02-25 unknown Faculty of Medicine Rodpan et al

USA/CruiseA-14/2020 EPI_ISL_413619 2020-02-25 unknown Pathogen Discovery, Respiratory Viruses Branch, Division of Viral Diseases, Centers for Disease Control and Prevention Clinton R. Paden et al

UnitedArabEmirates/L0184/2020 EPI_ISL_435121 2020-02-25 Mohammed Bin Rashid University of Medicine and Health Sciences Al Jalila Genomics Center Ahmad Abou Tayoun et al

UnitedArabEmirates/L0904/2020 EPI_ISL_435126 2020-02-25 Mohammed Bin Rashid University of Medicine and Health Sciences Al Jalila Genomics Center Ahmad Abou Tayoun et al

UnitedArabEmirates/L2409/2020 EPI_ISL_435131 2020-02-25 Mohammed Bin Rashid University of Medicine and Health Sciences Al Jalila Genomics Center Ahmad Abou Tayoun et al

UnitedArabEmirates/L4682/2020 EPI_ISL_435135 2020-02-25 Mohammed Bin Rashid University of Medicine and Health Sciences Al Jalila Genomics Center Ahmad Abou Tayoun et al

UnitedArabEmirates/L6599/2020 EPI_ISL_435138 2020-02-25 Mohammed Bin Rashid University of Medicine and Health Sciences Al Jalila Genomics Center Ahmad Abou Tayoun et al

UnitedArabEmirates/L6627/2020 EPI_ISL_435139 2020-02-25 Mohammed Bin Rashid University of Medicine and Health Sciences Al Jalila Genomics Center Ahmad Abou Tayoun et al

Austria/CeMM0002/2020 EPI_ISL_419655 2020-02-26 Center for Virology, Medical University of Vienna Bergthaler laboratory, CeMM Research Center for Molecular Medicine of the Austrian Academy of Sciences Alexandra Popa et al

Austria/CeMM0003/2020 EPI_ISL_419656 2020-02-26 Center for Virology, Medical University of Vienna Bergthaler laboratory, CeMM Research Center for Molecular Medicine of the Austrian Academy of Sciences Alexandra Popa et al

Denmark/SSI-01/2020 EPI_ISL_416142 2020-02-26 Department of Virus and Microbiological Special diagnostics, Statens Serum Institut, Copenhagen, Denmark. Statens Serum Institute Morten Rasmussen et al

England/200990006/2020 EPI_ISL_414011 2020-02-26 Respiratory Virus Unit, Microbiology Services Colindale, Public Health England Respiratory Virus Unit, Microbiology Services Colindale, Public Health England Monica Galiano et al

France/B1623/2020 EPI_ISL_418219 2020-02-26 CHU - H√¥pital Cavale Blanche - Labo. de Virologie National Reference Center for Viruses of Respiratory Infections, Institut Pasteur, Paris M√©lanie Albert et al

France/B2330/2020 EPI_ISL_416502 2020-02-26 CHRU Pontchaillou - Laboratoire de Virologie National Reference Center for Viruses of Respiratory Infections, Institut Pasteur, Paris M√©lnie Albert et al

France/N1620/2020 EPI_ISL_414624 2020-02-26 Centre Hositalier Universitaire de Rouen Laboratoire de Virologie National Reference Center for Viruses of Respiratory Infections, Institut Pasteur, Paris M√©lnie Albert et al

France/PL1643/2020 EPI_ISL_414625 2020-02-26 Centre Hospitalier R√©gional Universitaire de Nantes Laboratoire de Virologie National Reference Center for Viruses of Respiratory Infections, Institut Pasteur, Paris M√©lnie Albert et al

HongKong/HKPU89-2502/2020 EPI_ISL_419250 2020-02-26 Department of Clinical Pathology, Pamela Youde Nethersole Eastern Hospital Department of Health Technology and Informatics, Faculty of Health and Social Science, The Hong Kong Polytechnic University Kenneth Siu-Sing LEUNG et al

Korea/KCDC2015/2020 EPI_ISL_427809 2020-02-26 Division of Viral Diseases, Center for Laboratory Control of Infectious Diseases, Korea Centers for Diseases Control and Prevention Division of Viral Diseases, Center for Laboratory Control of Infectious Diseases, Korea Centers for Diseases Control and Prevention Jeong-Min Kim et al

Nanchang/JXN3T4/2020 EPI_ISL_421246 2020-02-26 Jiangxi Province Center for Disease Control and Prevention Jiangxi Province Center for Disease Control and Prevention JianXiong Li et al

Norway/1380/2020 EPI_ISL_417484 2020-02-26 Oslo University Hospital, Department of Medical Microbiology Norwegian Institute of Public Health, Department of Virology Kathrine Stene-Johansen et al

Spain/CastillayLeon201061/2020 EPI_ISL_418247 2020-02-26 HOSPITAL GENERAL DE SEGOVIA Instituto de Salud Carlos III Iglesias-Caballero et al

Switzerland/1000477102/2020 EPI_ISL_413019 2020-02-26 Department of Internal Medicine, Triemli Hospital Institute of Medical Virology, University of Zurich Stefan Schmutz et al

Switzerland/GE3895/2020 EPI_ISL_413997 2020-02-26 Laboratoire de Virologie, HUG Swiss National Reference Centre for Influenza LAUBSCHER Florian et al. et al

Taiwan/CGMH-CGU-03/2020 EPI_ISL_415741 2020-02-26 Laboratory Medicine Department of Laboratory Medicine, Lin-Kou Chang Gung Memorial Hospital, Taoyuan, Taiwan Kuo-Chien Tsao et al

USA/CA-CDPH-UC1/2020 EPI_ISL_413557 2020-02-26 California Department of Public Health Chiu Laboratory, University of California, San Francisco Xianding Deng et al

USA/CA-SCCPHD-UC101/2020 EPI_ISL_435580 2020-02-26 Santa Clara County Public Health Department Chiu Laboratory, University of California, San Francisco Xianding Deng et al

USA/CA_2602/2020 EPI_ISL_419554 2020-02-26 California Department of Public Health Pathogen Discovery, Respiratory Viruses Branch, Division of Viral Diseases, Centers for Disease Control and Prevention Ying Tao et al

USA/WA-S272/2020 EPI_ISL_430126 2020-02-26 Seattle Flu Study Seattle Flu Study Chu et al et al

USA/WA-S81/2020 EPI_ISL_417134 2020-02-26 Washington State Department of Health Seattle Flu Study Chu etl al et al

England/200990660/2020 EPI_ISL_414523 2020-02-27 Respiratory Virus Unit, Microbiology Services Colindale, Public Health England Respiratory Virus Unit, Microbiology Services Colindale, Public Health England Monica Galiano et al

England/200990723/2020 EPI_ISL_414012 2020-02-27 Respiratory Virus Unit, Microbiology Services Colindale, Public Health England Respiratory Virus Unit, Microbiology Services Colindale, Public Health England Monica Galiano et al

Georgia/Tb-54/2020 EPI_ISL_415641 2020-02-27 R. G. Lugar Center for Public Health Research, National Center for Disease Control and Public Health (NCDC) of Georgia. R. G. Lugar Center for Public Health Research, National Center for Disease Control and Public Health (NCDC) of Georgia. Nato Kotaria et al

Germany/NRW-06/2020 EPI_ISL_414505 2020-02-27 Center of Medical Microbiology, Virology, and Hospital Hygiene, University of Duesseldorf Center of Medical Microbiology, Virology, and Hospital Hygiene, University of Duesseldorf Ortwin Adams et al

Germany/NRW-08/2020 EPI_ISL_414508 2020-02-27 Center of Medical Microbiology, Virology, and Hospital Hygiene, University of Duesseldorf Center of Medical Microbiology, Virology, and Hospital Hygiene, University of Duesseldorf Ortwin Adams et al

Korea/BA-ACH_2604/2020 EPI_ISL_420799 2020-02-27 Brian D. Allgood Army Community Hospital Pathogen Discovery, Respiratory Viruses Branch, Division of Viral Diseases, Centers for Disease Control and Prevention Krista Queen et al

Mexico/CDMX-InDRE_01/2020 EPI_ISL_412972 2020-02-27 Instituto Nacional de Enfermedades Respiratorias Instituto de Diagnostico y Referencia Epidemiologicos (INDRE) Ramirez-Gonzalez Ernesto et al

NewZealand/01/2020 EPI_ISL_413490 2020-02-27 Auckland Hospital Institute of Environmental Science and Research (ESR) Matt Storey et al

Norway/1379/2020 EPI_ISL_417483 2020-02-27 Oslo University Hospital, Department of Medical Microbiology Norwegian Institute of Public Health Kathrine Stene-Johansen et al

Singapore/51/2020 EPI_ISL_428830 2020-02-27 National Public Health Laboratory, National Centre for Infectious Diseases National Public Health Laboratory, National Centre for Infectious Diseases Mak TM et al

SouthKorea/KUMC03/2020 EPI_ISL_413513 2020-02-27 Division of Infectious Diseases, Department of Internal Medicine, Korea University College of Medicine Department of Microbiology, Institute for Viral Diseases, College of Medicine, Korea University Changmin Kang et al

SouthKorea/KUMC04/2020 EPI_ISL_413514 2020-02-27 Department of Microbiology, Institute for Viral Diseases, College of Medicine, Korea University Department of Microbiology, Institute for Viral Diseases, College of Medicine, Korea University Changmin Kang et al

SouthKorea/KUMC05/2020 EPI_ISL_413515 2020-02-27 Division of Infectious Diseases, Department of Internal Medicine, Korea University College of Medicine Department of Microbiology, Institute for Viral Diseases, College of Medicine, Korea University Changmin Kang et al

SouthKorea/KUMC06/2020 EPI_ISL_413516 2020-02-27 Department of Microbiology, Institute for Viral Diseases, College of Medicine, Korea University Department of Microbiology, Institute for Viral Diseases, College of Medicine, Korea University Changmin Kang et al

Spain/Valencia5/2020 EPI_ISL_416484 2020-02-27 Servicio de Microbiolog√≠a. Consorcio Hospital General Universitario de Valencia Sequencing and Bioinformatics Service and Molecular Epidemiology Research Group. FISABIO-Public Health Maria Dolores Ocete et al

Sweden/20-04631/2020 EPI_ISL_430847 2020-02-27 HS mikrobiologi virus The Public Health Agency of Sweden Zhibing Yun et al

Switzerland/1000477377/2020 EPI_ISL_413020 2020-02-27 Department of Internal Medicine, Triemli Hospital Institute of Medical Virology, University of Zurich Stefan Schmutz et al

Switzerland/AG0361/2020 EPI_ISL_413999 2020-02-27 Laboratoire de Virologie, HUG Swiss National Reference Centre for Influenza LAUBSCHER Florian et al. et al

Switzerland/BL0902/2020 EPI_ISL_414021 2020-02-27 Laboratoire de Virologie, HUG Swiss National Reference Centre for Influenza LAUBSCHER Florian et al. et al

Switzerland/GE3121/2020 EPI_ISL_414019 2020-02-27 Laboratoire de Virologie, HUG Swiss National Reference Centre for Influenza LAUBSCHER Florian et al. et al

Switzerland/GE5373/2020 EPI_ISL_414020 2020-02-27 Laboratoire de Virologie, HUG Swiss National Reference Centre for Influenza LAUBSCHER Florian et al. et al

Switzerland/GE9586/2020 EPI_ISL_414022 2020-02-27 Laboratoire de Virologie, HUG Swiss National Reference Centre for Influenza LAUBSCHER Florian et al. et al

Switzerland/GR2988/2020 EPI_ISL_415698 2020-02-27 H√¥pitaux universitaires de Gen√®ve Laboratoire de Virologie H√¥pitaux universitaires de Gen√®ve Laboratoire de Virologie Laubscher F. et al

Switzerland/GR3043/2020 EPI_ISL_415699 2020-02-27 H√¥pitaux universitaires de Gen√®ve Laboratoire de Virologie H√¥pitaux universitaires de Gen√®ve Laboratoire de Virologie Laubscher F. et al

Taiwan/CGMH-CGU-04/2020 EPI_ISL_415742 2020-02-27 Laboratory Medicine Department of Laboratory Medicine, Lin-Kou Chang Gung Memorial Hospital, Taoyuan, Taiwan Kuo-Chien Tsao et al

Taiwan/CGMH-CGU-05/2020 EPI_ISL_415743 2020-02-27 Laboratory Medicine Department of Laboratory Medicine, Lin-Kou Chang Gung Memorial Hospital, Taoyuan, Taiwan Kuo-Chien Tsao et al

USA/CA-CDPH-UC2/2020 EPI_ISL_413558 2020-02-27 California Department of Public Health Chiu Laboratory, University of California, San Francisco Xianding Deng et al

USA/CA-CDPH-UC3/2020 EPI_ISL_413559 2020-02-27 California Department of Public Health Chiu Laboratory, University of California, San Francisco Xianding Deng et al

USA/CA-CDPH-UC4/2020 EPI_ISL_413561 2020-02-27 California Department of Public Health Chiu Laboratory, University of California, San Francisco Xianding Deng et al

USA/OR_2656/2020 EPI_ISL_419558 2020-02-27 OR State PHL-Virology/Immunology Section Pathogen Discovery, Respiratory Viruses Branch, Division of Viral Diseases, Centers for Disease Control and Prevention Ying Tao et al

USA/WA-S43/2020 EPI_ISL_417096 2020-02-27 Washington State Department of Health Seattle Flu Study Chu etl al et al

USA/WA3-UW1/2020 EPI_ISL_413025 2020-02-27 Harborview Medical Center UW Virology Lab Pavitra Roychoudhury et al

USA/WA_5030/2020 EPI_ISL_419555 2020-02-27 WA State Department of Health Pathogen Discovery, Respiratory Viruses Branch, Division of Viral Diseases, Centers for Disease Control and Prevention Ying Tao et al

Wales/PHW1/2020 EPI_ISL_413555 2020-02-27 Wales Specialist Virology Centre Public Health Wales Microbiology Cardiff Catherine Moore et al

Australia/NSW05/2020 EPI_ISL_412975 2020-02-28 Centre for Infectious Diseases and Microbiology Laboratory Services NSW Health Pathology - Institute of Clinical Pathology and Medical Research; Westmead Hospital; University of Sydney Eden J-S et al

Australia/NSW08/2020 EPI_ISL_413594 2020-02-28 Centre for Infectious Diseases and Microbiology Laboratory Services NSW Health Pathology - Institute of Clinical Pathology and Medical Research; Westmead Hospital; University of Sydney Rockett R et al

Australia/NSW09/2020 EPI_ISL_413595 2020-02-28 Centre for Infectious Diseases and Microbiology Laboratory Services NSW Health Pathology - Institute of Clinical Pathology and Medical Research; Westmead Hospital; University of Sydney Rockett R et al

Australia/NSW10/2020 EPI_ISL_413596 2020-02-28 Centre for Infectious Diseases and Microbiology - Public Health NSW Health Pathology - Institute of Clinical Pathology and Medical Research; Westmead Hospital; University of Sydney Rockett R et al

Australia/NSW219/202 EPI_ISL_427643 2020-02-28 Centre for Infectious Diseases and Microbiology Public Health NSW Health Pathology - Institute of Clinical Pathology and Medical Research; Westmead Hospital; University of Sydney Timms V et al

Australia/QLDID922/2020 EPI_ISL_418799 2020-02-28 Mater Pathology Public Health Virology Laboratory Bixing Huang et al

Austria/CeMM0146/2020 EPI_ISL_437994 2020-02-28 Center for Virology, Medical University of Vienna Bergthaler laboratory, CeMM Research Center for Molecular Medicine of the Austrian Academy of Sciences Alexandra Popa et al

Brazil/SPBR-02/2020 EPI_ISL_413016 2020-02-28 Hospital Israelita Albert Einstein Instituto Adolfo Lutz, Interdisciplinary Procedures Center, Strategic Laboratory Jaqueline Goes de Jesus et al

Denmark/SSI-02/2020 EPI_ISL_416143 2020-02-28 Department of Virus and Microbiological Special diagnostics, Statens Serum Institut, Copenhagen, Denmark. ViFU Morten Rasmussen et al

England/200990002/2020 EPI_ISL_414522 2020-02-28 Respiratory Virus Unit, Microbiology Services Colindale, Public Health England Respiratory Virus Unit, Microbiology Services Colindale, Public Health England Monica Galiano et al

England/200990724/2020 EPI_ISL_414006 2020-02-28 Respiratory Virus Unit, Microbiology Services Colindale, Public Health England Respiratory Virus Unit, Microbiology Services Colindale, Public Health England Monica Galiano et al

England/200990725/2020 EPI_ISL_414007 2020-02-28 Respiratory Virus Unit, Microbiology Services Colindale, Public Health England Respiratory Virus Unit, Microbiology Services Colindale, Public Health England Monica Galiano et al

France/HF1645/2020 EPI_ISL_418220 2020-02-28 Centre Hospitalier Compi√®gne Laboratoire de Biologie National Reference Center for Viruses of Respiratory Infections, Institut Pasteur, Paris M√©lanie Albert et al

Georgia/Tb-82/2020 EPI_ISL_415644 2020-02-28 R. G. Lugar Center for Public Health Research, National Center for Disease Control and Public Health (NCDC) of Georgia. R. G. Lugar Center for Public Health Research, National Center for Disease Control and Public Health (NCDC) of Georgia. Nato Kotaria et al

Germany/NRW-09/2020 EPI_ISL_414509 2020-02-28 Center of Medical Microbiology, Virology, and Hospital Hygiene, University of Duesseldorf Center of Medical Microbiology, Virology, and Hospital Hygiene, University of Duesseldorf Ortwin Adams et al

Italy/INMI4/2020 EPI_ISL_417922 2020-02-28 INMI Lazzaro Spallanzani IRCCS Laboratory of Virology, INMI Lazzaro Spallanzani IRCCS Cesare E. M. Gruber et al

Senegal/003/2020 EPI_ISL_418206 2020-02-28 Institut Pasteur Dakar Institut Pasteur de Dakar Ndongo Dia et al

Singapore/12Clin/2020 EPI_ISL_418995 2020-02-28 National Public Health Laboratory, National Centre for Infectious Diseases National Public Health Laboratory, National Centre for Infectious Diseases Mak TM et al

Spain/Andalucia201272/2020 EPI_ISL_418243 2020-02-28 HOSPITAL UNIVERSITARIO VIRGEN DE LAS NIEVES Instituto de Salud Carlos III Iglesias-Caballero et al

Spain/Valencia54/2020 EPI_ISL_425178 2020-02-28 Servicio de Microbiolog√≠a. Consorcio Hospital General Universitario de Valencia Sequencing and Bioinformatics Service and Molecular Epidemiology Research Group. FISABIO-Public Health David Navarro et al

Switzerland/GE1402/2020 EPI_ISL_415700 2020-02-28 H√¥pitaux universitaires de Gen√®ve Laboratoire de Virologie H√¥pitaux universitaires de Gen√®ve Laboratoire de Virologie Laubscher F. et al

Switzerland/GE1422/2020 EPI_ISL_415454 2020-02-28 H√¥pitaux universitaires de Gen√®ve Laboratoire de Virologie H√¥pitaux universitaires de Gen√®ve Laboratoire de Virologie Laubscher F. et al

USA/FL_5091/2020 EPI_ISL_419560 2020-02-28 FL Bureau of Public Health Laboratories-Tampa Pathogen Discovery, Respiratory Viruses Branch, Division of Viral Diseases, Centers for Disease Control and Prevention Anna Uehara et al

USA/FL_5125/2020 EPI_ISL_419559 2020-02-28 FL Bureau of Public Health Laboratories-Tampa Pathogen Discovery, Respiratory Viruses Branch, Division of Viral Diseases, Centers for Disease Control and Prevention Anna Uehara et al

USA/RI_0520/2020 EPI_ISL_419553 2020-02-28 RI State Health Laboratories Pathogen Discovery, Respiratory Viruses Branch, Division of Viral Diseases, Centers for Disease Control and Prevention Ying Tao et al

USA/WA-S101/2020 EPI_ISL_417154 2020-02-28 Washington State Department of Health Seattle Flu Study Chu etl al et al

USA/WA-S102/2020 EPI_ISL_417155 2020-02-28 Washington State Department of Health Seattle Flu Study Chu etl al et al

USA/WA-S103/2020 EPI_ISL_417156 2020-02-28 Washington State Department of Health Seattle Flu Study Chu etl al et al

USA/WA-S105/2020 EPI_ISL_417158 2020-02-28 Washington State Department of Health Seattle Flu Study Chu etl al et al

USA/WA-S234/2020 EPI_ISL_430272 2020-02-28 Washington State Department of Health Seattle Flu Study Chu et al et al

USA/WA-S3/2020 EPI_ISL_413560 2020-02-28 Seattle Flu Study Seattle Flu Study Chu et al et al

USA/WA-S40/2020 EPI_ISL_417093 2020-02-28 Washington State Department of Health Seattle Flu Study Chu etl al et al

USA/WA-S42/2020 EPI_ISL_417095 2020-02-28 Washington State Department of Health Seattle Flu Study Chu etl al et al

USA/WA-S44/2020 EPI_ISL_417097 2020-02-28 Washington State Department of Health Seattle Flu Study Chu etl al et al

USA/WA-S94/2020 EPI_ISL_417147 2020-02-28 Washington State Department of Health Seattle Flu Study Chu etl al et al

USA/WA-S95/2020 EPI_ISL_417148 2020-02-28 Washington State Department of Health Seattle Flu Study Chu etl al et al

USA/WA-S96/2020 EPI_ISL_417149 2020-02-28 Washington State Department of Health Seattle Flu Study Chu etl al et al

USA/WA-S99/2020 EPI_ISL_417152 2020-02-28 Washington State Department of Health Seattle Flu Study Chu etl al et al

USA/WA4-UW2/2020 EPI_ISL_413455 2020-02-28 Washington State Public Health Lab University of Washington Virology Lab Pavitra Roychoudhury et al

Australia/NSW06/2020 EPI_ISL_413213 2020-02-29 Centre for Infectious Diseases and Microbiology Laboratory Services NSW Health Pathology - Institute of Clinical Pathology and Medical Research; Westmead Hospital; University of Sydney Eden J-S et al

Australia/NSW07/2020 EPI_ISL_413214 2020-02-29 Centre for Infectious Diseases and Microbiology Laboratory Services NSW Health Pathology - Institute of Clinical Pathology and Medical Research; Westmead Hospital; University of Sydney Eden J-S et al

Australia/QLD09/2020 EPI_ISL_414414 2020-02-29 Pathology Queensland Public Health Virology Laboratory Bixing Huang et al

Belgium/BA-02291/2020 EPI_ISL_415159 2020-02-29 KU Leuven, Clinical and Epidemiological Virology KU Leuven, Clinical and Epidemiological Virology Bert Vanmechelen et al

Brazil/ES-225/2020 EPI_ISL_415128 2020-02-29 LACEN/ES - Laborat√≥rio Central de Sa√∫de P√∫blica do Esp√≠rito Santo Instituto Oswaldo Cruz FIOCRUZ - Laboratory of Respiratory Viruses and Measles (LVRS) Paola Resende et al

Brazil/SPBR-05/2020 EPI_ISL_414016 2020-02-29 Hospital S√£o Joaquim Beneficencia Portuguesa Instituto Adolfo Lutz, Interdiciplinary Procedures Center, Strategic Laboratory Claudio Tavares Sacchi et al

Brazil/SPBR-06/2020 EPI_ISL_414015 2020-02-29 Hospital S√£o Joaquim Beneficencia Portuguesa Instituto Adolfo Lutz, Interdiciplinary Procedures Center, Strategic Laboratory Claudio Tavares Sacchi et al

Canada/ON_PHL8751/2020 EPI_ISL_418345 2020-02-29 Public Health Ontario Laboratories Public Health Ontario Laboratories Alireza Eshaghi et al

England/20099038206/2020 EPI_ISL_415129 2020-02-29 Respiratory Virus Unit, Microbiology Services Colindale, Public Health England Respiratory Virus Unit, Microbiology Services Colindale, Public Health England Monica Galiano et al

England/20100004806/2020 EPI_ISL_415134 2020-02-29 Respiratory Virus Unit, Microbiology Services Colindale, Public Health England Respiratory Virus Unit, Microbiology Services Colindale, Public Health England Monica Galiano et al

England/20100121007/2020 EPI_ISL_415141 2020-02-29 Respiratory Virus Unit, Microbiology Services Colindale, Public Health England Respiratory Virus Unit, Microbiology Services Colindale, Public Health England Monica Galiano et al

France/HF1684/2020 EPI_ISL_414626 2020-02-29 unknown National Reference Center for Viruses of Respiratory Infections, Institut Pasteur, Paris M√©lnie Albert et al

Greece/127_HPI/2020 EPI_ISL_430469 2020-02-29 Hellenic Pasteur Institute, Public Health Laboratories Hellenic Pasteur Institute, Public Health Laboratories, Unit of Bioinformatics and Applied Genomics Vasiliki Pogka et al

Korea/BA-ACH_2718/2020 EPI_ISL_420800 2020-02-29 Brian D. Allgood Army Community Hospital Pathogen Discovery, Respiratory Viruses Branch, Division of Viral Diseases, Centers for Disease Control and Prevention Krista Queen et al

Korea/BA-ACH_2719/2020 EPI_ISL_420801 2020-02-29 Brian D. Allgood Army Community Hospital Pathogen Discovery, Respiratory Viruses Branch, Division of Viral Diseases, Centers for Disease Control and Prevention Krista Queen et al

Luxembourg/LNS0000001/2020 EPI_ISL_419562 2020-02-29 Laboratoire National de Sant√©, Microbiology, Virology Laboratoire National de Sant√©, Microbiology, Epidemiology and Microbial Genomics Anke Wienecke-Baldacchino et al

Luxembourg/Lux1/2020 EPI_ISL_413593 2020-02-29 Laboratoire National de Sant√© Erasmus Medical Center David Nieuwenhuijse et al

Malaysia/IMR_WC1097/2020 EPI_ISL_430441 2020-02-29 Institute for Medical Research, Infectious Disease Research Centre, National Institutes of Health, Ministry of Health Malaysia Institute for Medical Research, Infectious Disease Research Centre, National Institutes of Health, Ministry of Health Malaysia Suppiah.J et al

Netherlands/Helmond_1363548/2020 EPI_ISL_413574 2020-02-29 MHC West-Brabant Erasmus Medical Center David Nieuwenhuijse et al

Norway/1493/2020 EPI_ISL_417488 2020-02-29 Oslo University Hospital, Department of Medical Microbiology Norwegian Institute of Public Health, Department of Virology Kathrine Stene-Johansen et al

Switzerland/1000477757/2020 EPI_ISL_413021 2020-02-29 Klinik Hirslanden Zurich Institute of Medical Virology, University of Zurich Stefan Schmutz et al

Switzerland/1000477796/2020 EPI_ISL_413022 2020-02-29 Division of Infectious Diseases, University Hospital Zurich Institute of Medical Virology, University of Zurich Stefan Schmutz et al

Switzerland/1000477797/2020 EPI_ISL_413023 2020-02-29 Division of Infectious Diseases, University Hospital Zurich Institute of Medical Virology, University of Zurich Stefan Schmutz et al

Switzerland/AG7120/2020 EPI_ISL_415457 2020-02-29 H√¥pitaux universitaires de Gen√®ve Laboratoire de Virologie H√¥pitaux universitaires de Gen√®ve Laboratoire de Virologie Laubscher F. et al

Switzerland/BE6651/2020 EPI_ISL_415456 2020-02-29 H√¥pitaux universitaires de Gen√®ve Laboratoire de Virologie H√¥pitaux universitaires de Gen√®ve Laboratoire de Virologie Laubscher F. et al

Switzerland/VD0503/2020 EPI_ISL_415459 2020-02-29 H√¥pitaux universitaires de Gen√®ve Laboratoire de Virologie H√¥pitaux universitaires de Gen√®ve Laboratoire de Virologie Laubscher F. et al

Taiwan/CGMH-CGU-21/2020 EPI_ISL_444274 2020-02-29 Laboratory Medicine Department of Laboratory Medicine, Lin-Kou Chang Gung Memorial Hospital, Taoyuan, Taiwan Kuo-Chien Tsao et al

USA/CA-SCCPHD-UC104/2020 EPI_ISL_435583 2020-02-29 Santa Clara County Public Health Department Chiu Laboratory, University of California, San Francisco Xianding Deng et al

USA/CA-SCCPHD-UC108/2020 EPI_ISL_435587 2020-02-29 Santa Clara County Public Health Department Chiu Laboratory, University of California, San Francisco Xianding Deng et al

USA/CA-SCCPHD-UC14/2020 EPI_ISL_417318 2020-02-29 Santa Clara County Public Health Department Chiu Laboratory, University of California, San Francisco Xianding Deng et al

USA/GA_2741/2020 EPI_ISL_419556 2020-02-29 GA Department of Public Health Laboratory Pathogen Discovery, Respiratory Viruses Branch, Division of Viral Diseases, Centers for Disease Control and Prevention Ying Tao et al

USA/GA_2742/2020 EPI_ISL_419557 2020-02-29 GA Department of Public Health Laboratory Pathogen Discovery, Respiratory Viruses Branch, Division of Viral Diseases, Centers for Disease Control and Prevention Ying Tao et al

USA/NH_0004/2020 EPI_ISL_420791 2020-02-29 NH Department of Health and Human Services Public Health Labs Pathogen Discovery, Respiratory Viruses Branch, Division of Viral Diseases, Centers for Disease Control and Prevention Krista Queen et al

USA/NY1-PV08001/2020 EPI_ISL_414476 2020-02-29 MSHS Clinical Microbiology Laboratories MSHS Pathogen Surveillance Program Gopi Patel et al

USA/WA-S10/2020 EPI_ISL_416465 2020-02-29 Seattle Flu Study Seattle Flu Study Chu et al et al

USA/WA-S100/2020 EPI_ISL_417153 2020-02-29 Washington State Department of Health Seattle Flu Study Chu etl al et al

USA/WA-S106/2020 EPI_ISL_417159 2020-02-29 Washington State Department of Health Seattle Flu Study Chu etl al et al

USA/WA-S107/2020 EPI_ISL_417160 2020-02-29 Washington State Department of Health Seattle Flu Study Chu etl al et al

USA/WA-S108/2020 EPI_ISL_417161 2020-02-29 Washington State Department of Health Seattle Flu Study Chu etl al et al

USA/WA-S119/2020 EPI_ISL_417172 2020-02-29 Washington State Department of Health Seattle Flu Study Chu etl al et al

USA/WA-S123/2020 EPI_ISL_430160 2020-02-29 Washington State Department of Health Seattle Flu Study Chu et al et al

USA/WA-S235/2020 EPI_ISL_430273 2020-02-29 Washington State Department of Health Seattle Flu Study Chu et al et al

USA/WA-S243/2020 EPI_ISL_430281 2020-02-29 Washington State Department of Health Seattle Flu Study Chu et al et al

USA/WA-S281/2020 EPI_ISL_430135 2020-02-29 Seattle Flu Study Seattle Flu Study Chu et al et al

USA/WA-S45/2020 EPI_ISL_417098 2020-02-29 Washington State Department of Health Seattle Flu Study Chu etl al et al

USA/WA-S46/2020 EPI_ISL_417099 2020-02-29 Washington State Department of Health Seattle Flu Study Chu etl al et al

USA/WA-S47/2020 EPI_ISL_417100 2020-02-29 Washington State Department of Health Seattle Flu Study Chu etl al et al

USA/WA-S48/2020 EPI_ISL_417101 2020-02-29 Washington State Department of Health Seattle Flu Study Chu etl al et al

USA/WA-S5/2020 EPI_ISL_416460 2020-02-29 Seattle Flu Study Seattle Flu Study Chu et al et al

USA/WA-S55/2020 EPI_ISL_417108 2020-02-29 Washington State Department of Health Seattle Flu Study Chu etl al et al

USA/WA-S6/2020 EPI_ISL_416461 2020-02-29 Seattle Flu Study Seattle Flu Study Chu et al et al

USA/WA-S89/2020 EPI_ISL_417142 2020-02-29 Washington State Department of Health Seattle Flu Study Chu etl al et al

USA/WA-S90/2020 EPI_ISL_417143 2020-02-29 Washington State Department of Health Seattle Flu Study Chu etl al et al

USA/WA-S92/2020 EPI_ISL_417145 2020-02-29 Washington State Department of Health Seattle Flu Study Chu etl al et al

USA/WA-S93/2020 EPI_ISL_417146 2020-02-29 Washington State Department of Health Seattle Flu Study Chu etl al et al

USA/WA-S98/2020 EPI_ISL_417151 2020-02-29 Washington State Department of Health Seattle Flu Study Chu etl al et al

USA/WA6-UW3/2020 EPI_ISL_413457 2020-02-29 Washington State Public Health Lab UW Virology Lab Pavitra Roychoudhury et al

Belgium/BC-03016/2020 EPI_ISL_415157 2020-03-01 KU Leuven, Clinical and Epidemiological Virology KU Leuven, Clinical and Epidemiological Virology Bert Vanmechelen et al

Belgium/BM-03012/2020 EPI_ISL_415154 2020-03-01 KU Leuven, Clincal and Epidemiological Virology KU Leuven, Clincal and Epidemiological Virology Bert Vanmechelen et al

Belgium/CG-030158/2020 EPI_ISL_418987 2020-03-01 KU Leuven, Clinical and Epidemiological Virology KU Leuven, Clinical and Epidemiological Virology Bert Vanmechelen et al

Belgium/QKJ-03015/2020 EPI_ISL_415158 2020-03-01 KU Leuven, Clinical and Epidemiological Virology KU Leuven, Clinical and Epidemiological Virology Bert Vanmechelen et al

Belgium/SH-03014/2020 EPI_ISL_415156 2020-03-01 KU Leuven, Clinical and Epidemiological Virology KU Leuven, Clinical and Epidemiological Virology Bert Vanmechelen et al

Belgium/VAG-03013/2020 EPI_ISL_415155 2020-03-01 KU Leuven, Clinical and Epidemiological Virology KU Leuven, Clinical and Epidemiological Virology Bert Vanmechelen et al

Canada/BC_02421/2020 EPI_ISL_415581 2020-03-01 BCCDC Public Health Laboratory BCCDC Public Health Laboratory Harrigan et al

Denmark/SSI-03/2020 EPI_ISL_416144 2020-03-01 Department of Virus and Microbiological Special diagnostics, Statens Serum Institut, Copenhagen, Denmark. ViFU Morten Rasmussen et al

England/200991076/2020 EPI_ISL_414524 2020-03-01 Respiratory Virus Unit, Microbiology Services Colindale, Public Health England Respiratory Virus Unit, Microbiology Services Colindale, Public Health England Monica Galiano et al

England/20102000506/2020 EPI_ISL_415147 2020-03-01 Respiratory Virus Unit, Microbiology Services Colindale, Public Health England Respiratory Virus Unit, Microbiology Services Colindale, Public Health England Monica Galiano et al

England/20102068502/2020 EPI_ISL_417213 2020-03-01 Respiratory Virus Unit, Microbiology Services Colindale, Public Health England Respiratory Virus Unit, Microbiology Services Colindale, Public Health England Monica Galiano et al

England/CAMB-84787/2020 EPI_ISL_440506 2020-03-01 Department of Pathology, University of Cambridge Wellcome Sanger Institute for the COVID-19 Genomics UK Consortium Luke W Meredith et al

France/B2334/2020 EPI_ISL_416503 2020-03-01 CHRU Pontchaillou - Laboratoire de Virologie National Reference Center for Viruses of Respiratory Infections, Institut Pasteur, Paris M√©lnie Albert et al

Iceland/12/2020 EPI_ISL_417688 2020-03-01 The National University Hospital of Iceland deCODE genetics Daniel F Gudbjartsson et al

Iceland/14/2020 EPI_ISL_417736 2020-03-01 The National University Hospital of Iceland deCODE genetics Daniel F Gudbjartsson et al

India/GMC-RK100/2020 EPI_ISL_431101 2020-03-01 Department of Microbiology,Gandhi Medical College and Hospital Virus Research Laboratory, Department of Zoology, Osmania University,Hyderabad,India Muttineni Radhakrishna et al

Israel/51137031/2020 EPI_ISL_447330 2020-03-01 Clinical Virology Laboratory, Soroka Medical Center and the Faculty of Health Sciences, Ben-Gurion University of the Negev Stern Lab Stern Lab et al

Israel/ISR_IT0320/2020 EPI_ISL_419210 2020-03-01 The Chaim Sheba Medical Center Israel Institute for Biological Research Inbar Cohen-Gihon et al

Italy/FVG-ICGEB_S1/2020 EPI_ISL_417418 2020-03-01 Laboratory of Molecular Virology International Center fro Genetic Engineering and Biotechnology (ICGEB) ARGO Open Lab Platform for Genome sequencing Licastro D et al

Italy/FVG-ICGEB_S5/2020 EPI_ISL_417419 2020-03-01 Laboratory of Molecular Virology International Center for Genetic Engineering and Biotechnology (ICGEB) ARGO Open Lab Platform for Genome sequencing Licastro D et al

Italy/FVG-ICGEB_S8/2020 EPI_ISL_417421 2020-03-01 Laboratory of Molecular Virology International Center for Genetic Engineering and Biotechnology (ICGEB) ARGO Open Lab Platform for Genome sequencing Licastro D et al

Italy/FVG-ICGEB_S9/2020 EPI_ISL_417423 2020-03-01 Laboratory of Molecular Virology International Center for Genetic Engineering and Biotechnology (ICGEB) ARGO Open Lab Platform for Genome sequencing Licastro D et al

Italy/INMI3/2020 EPI_ISL_417921 2020-03-01 INMI Lazzaro Spallanzani IRCCS Laboratory of Virology, INMI Lazzaro Spallanzani IRCCS Martina Rueca et al

Netherlands/Andel_1365066/2020 EPI_ISL_413564 2020-03-01 MHC West-Brabant Erasmus Medical Center David Nieuwenhuijse et al

Netherlands/Dalen_1363624/2020 EPI_ISL_413568 2020-03-01 MHC Drente Erasmus Medical Center David Nieuwenhuijse et al

Netherlands/Haarlem_1363688/2020 EPI_ISL_413572 2020-03-01 MHC Kennemerland Erasmus Medical Center David Nieuwenhuijse et al

Netherlands/Rotterdam_1363790/2020 EPI_ISL_413582 2020-03-01 ErasmusMC Erasmus Medical Center David Nieuwenhuijse et al

Netherlands/Utrecht_1363564/2020 EPI_ISL_413588 2020-03-01 MHC Utrecht Erasmus Medical Center David Nieuwenhuijse et al

Netherlands/Utrecht_1363628/2020 EPI_ISL_413589 2020-03-01 MHC Utrecht Erasmus Medical Center David Nieuwenhuijse et al

Norway/1539/2020 EPI_ISL_417487 2020-03-01 Hospital of Southern Norway - Kristiansand, Department of Medical Microbiology Norwegian Institute of Public Health, Department of Virology Kathrine Stene-Johansen et al

Portugal/CV62/2020 EPI_ISL_413647 2020-03-01 Centro Hospital do Porto, E.P.E. - H. Geral de Santo Antonio Instituto Nacional de Saude (INSA) Raquel Guiomar et al

Portugal/CV63/2020 EPI_ISL_413648 2020-03-01 Centro Hospitalar e Universit√°rio de Sao Joao, Porto Instituto Nacional de Saude (INSA) Raquel Guiomar et al

Singapore/18/2020 EPI_ISL_418999 2020-03-01 National Public Health Laboratory, National Centre for Infectious Diseases National Public Health Laboratory, National Centre for Infectious Diseases Mak TM et al

Spain/CastillaLaMancha201328/2020 EPI_ISL_418245 2020-03-01 Hospital General y Universitario de Guadalajara Instituto de Salud Carlos III Iglesias-Caballero et al

Spain/CastillayLeon201323/2020 EPI_ISL_418248 2020-03-01 COMPLEJO ASISTENCIAL UNIVERSITARIO DE BURGOS Instituto de Salud Carlos III Iglesias-Caballero et al

Switzerland/GE8102/2020 EPI_ISL_415458 2020-03-01 H√¥pitaux universitaires de Gen√®ve Laboratoire de Virologie H√¥pitaux universitaires de Gen√®ve Laboratoire de Virologie Laubscher F. et al

Switzerland/TI2045/2020 EPI_ISL_415703 2020-03-01 H√¥pitaux universitaires de Gen√®ve Laboratoire de Virologie H√¥pitaux universitaires de Gen√®ve Laboratoire de Virologie Laubscher F. et al

Switzerland/VD5615/2020 EPI_ISL_414023 2020-03-01 Laboratoire de Virologie, HUG Swiss National Reference Centre for Influenza LAUBSCHER Florian et al. et al

Thailand/SI206377-NST/2020 EPI_ISL_437604 2020-03-01 unknown Faculty of Medicine Rodpan et al

USA/IL_1293/2020 EPI_ISL_420790 2020-03-01 Illinois Department of Public Health Chicago Laboratory Pathogen Discovery, Respiratory Viruses Branch, Division of Viral Diseases, Centers for Disease Control and Prevention Krista Queen et al

USA/IL_1375/2020 EPI_ISL_420789 2020-03-01 Illinois Department of Public Health Chicago Laboratory Pathogen Discovery, Respiratory Viruses Branch, Division of Viral Diseases, Centers for Disease Control and Prevention Krista Queen et al

USA/OR_5430/2020 EPI_ISL_420794 2020-03-01 Oregon State Public Health- Virology section Pathogen Discovery, Respiratory Viruses Branch, Division of Viral Diseases, Centers for Disease Control and Prevention Krista Queen et al

USA/RI_0556/2020 EPI_ISL_420795 2020-03-01 RI State Health Laboratory Pathogen Discovery, Respiratory Viruses Branch, Division of Viral Diseases, Centers for Disease Control and Prevention Krista Queen et al

USA/TX_2817/2020 EPI_ISL_420797 2020-03-01 Texas DSHS Lab Services Pathogen Discovery, Respiratory Viruses Branch, Division of Viral Diseases, Centers for Disease Control and Prevention Krista Queen et al

USA/TX_2967/2020 EPI_ISL_420798 2020-03-01 Texas DSHS Lab Services Pathogen Discovery, Respiratory Viruses Branch, Division of Viral Diseases, Centers for Disease Control and Prevention Krista Queen et al

USA/WA-S109/2020 EPI_ISL_417162 2020-03-01 Washington State Department of Health Seattle Flu Study Chu etl al et al

USA/WA-S118/2020 EPI_ISL_417171 2020-03-01 Washington State Department of Health Seattle Flu Study Chu etl al et al

USA/WA-S236/2020 EPI_ISL_430274 2020-03-01 Washington State Department of Health Seattle Flu Study Chu et al et al

USA/WA-S247/2020 EPI_ISL_430285 2020-03-01 Washington State Department of Health Seattle Flu Study Chu et al et al

USA/WA-S249/2020 EPI_ISL_430287 2020-03-01 Washington State Department of Health Seattle Flu Study Chu et al et al

USA/WA-S33/2020 EPI_ISL_417086 2020-03-01 Washington State Department of Health Seattle Flu Study Chu etl al et al

USA/WA-S86/2020 EPI_ISL_417139 2020-03-01 Washington State Department of Health Seattle Flu Study Chu etl al et al

USA/WA-S87/2020 EPI_ISL_417140 2020-03-01 Washington State Department of Health Seattle Flu Study Chu etl al et al

USA/WA-S88/2020 EPI_ISL_417141 2020-03-01 Washington State Department of Health Seattle Flu Study Chu etl al et al

USA/WA7-UW4/2020 EPI_ISL_413458 2020-03-01 Washington State Public Health Lab UW Virology Lab Pavitra Roychoudhury et al

USA/WA8-UW5/2020 EPI_ISL_413486 2020-03-01 Valley Medical Center University of Washington Virology Lab Pavitra Roychoudhury et al

USA/WA9-UW6/2020 EPI_ISL_413487 2020-03-01 Harborview Medical Center University of Washington Virology Lab Pavitra Roychoudhury et al

Algeria/G0638_2264/2020 EPI_ISL_418241 2020-03-02 NIC Viral Respiratory Unit - Institut Pasteur of Algeria National Reference Center for Viruses of Respiratory Infections, Institut Pasteur, Paris M√©lanie Albert et al

Algeria/G0860_2262/2020 EPI_ISL_420037 2020-03-02 NIC Viral Respiratory Unit - Institut Pasteur of Algeria National Reference Center for Viruses of Respiratory Infections, Institut Pasteur, Paris M√©lanie Albert et al

Australia/NSW11/2020 EPI_ISL_413597 2020-03-02 Centre for Infectious Diseases and Microbiology- Public Health NSW Health Pathology - Institute of Clinical Pathology and Medical Research; Westmead Hospital; University of Sydney Lam C et al

Australia/NSW217/2020 EPI_ISL_427644 2020-03-02 Centre for Infectious Diseases and Microbiology Public Health NSW Health Pathology - Institute of Clinical Pathology and Medical Research; Westmead Hospital; University of Sydney Rockett R et al

Australia/VIC04/2020 EPI_ISL_416412 2020-03-02 Victorian Infectious Diseases Reference Laboratory (VIDRL) Victorian Infectious Diseases Reference Laboratory and Microbiological Diagnostic Unit Public Health Laboratory, Doherty Institute Caly L. et al

Austria/CeMM0148/2020 EPI_ISL_437996 2020-03-02 Center for Virology, Medical University of Vienna Bergthaler laboratory, CeMM Research Center for Molecular Medicine of the Austrian Academy of Sciences Alexandra Popa et al

Belgium/DB-03023/2020 EPI_ISL_416470 2020-03-02 KU Leuven, Clinical and Epidemiological Virology KU Leuven, Clinical and Epidemiological Virology Bert Vanmechelen et al

Belgium/DBD-03024/2020 EPI_ISL_416471 2020-03-02 KU Leuven, Clinical and Epidemiological Virology KU Leuven, Clinical and Epidemiological Virology Bert Vanmechelen et al

Belgium/FR-03029/2020 EPI_ISL_417428 2020-03-02 KU Leuven, Clinical and Epidemiological Virology KU Leuven, Clinical and Epidemiological Virology Joan Marti-Carerras et al

Belgium/GMH-03022/2020 EPI_ISL_416468 2020-03-02 KU Leuven, Clinical and Epidemiological Virology KU Leuven, Clinical and Epidemiological Virology Bert Vanmechelen et al

Belgium/MTR-03021/2020 EPI_ISL_416467 2020-03-02 KU Leuven, Clinical and Epidemiological Virology KU Leuven, Clinical and Epidemiological Virology Bert Vanmechelen et al

Belgium/MTR-03026/2020 EPI_ISL_416476 2020-03-02 KU Leuven, Clinical and Epidemiological Virology KU Leuven, Clinical and Epidemiological Virology Bert Vanmechelen et al

Belgium/RS-030257/2020 EPI_ISL_418986 2020-03-02 KU Leuven, Clinical and Epidemiological Virology KU Leuven, Clinical and Epidemiological Virology Bert Vanmechelen et al

Belgium/UMF-03025/2020 EPI_ISL_416472 2020-03-02 KU Leuven, Clinical and Epidemiological Virology KU Leuven, Clinical and Epidemiological Virology Bert Vanmechelen et al

Belgium/VI-03027/2020 EPI_ISL_417427 2020-03-02 KU Leuven, Clinical and Epidemiological Virology KU Leuven, Clinical and Epidemiological Virology Joan Marti-Carerras et al

Brazil/SPBR-03/2020 EPI_ISL_414014 2020-03-02 Hospital Israelita Albert Einstein Instituto Adolfo Lutz, Interdiciplinary Procedures Center, Strategic Laboratory Claudio Tavares Sacchi et al

Canada/BC_41851/2020 EPI_ISL_415584 2020-03-02 BCCDC Public Health Laboratory BCCDC Public Health Laboratory Harrigan et al

Chile/Talca-1/2020 EPI_ISL_414577 2020-03-02 Hospital de Talca, Chile Instituto de Salud Publica de Chile Andr√©s E. Castillo et al

Denmark/ALAB-SSI-248/2020 EPI_ISL_437663 2020-03-02 Department of Virus and Microbiological Special Diagnostics, Statens Serum Institut, Copenhagen, Denmark, Artillerivej 5, 2300 Copenahgen S Albertsen lab, Department of Chemistry and Bioscience, Aalborg University, Denmark Rasmus Kirkegaard et al

Denmark/ALAB-SSI103/2020 EPI_ISL_429335 2020-03-02 Department of Virus and Microbiological Special Diagnostics, Statens Serum Institut, Copenhagen, Denmark, Artillerivej 5, 2300 Copenahgen S Albertsen lab, Department of Chemistry and Bioscience, Aalborg University, Denmark Rasmus Kirkegaard et al

Denmark/SSI-04/2020 EPI_ISL_416153 2020-03-02 Department of Virus and Microbiological Special diagnostics, Statens Serum Institut, Copenhagen, Denmark. ViFU Morten Rasmussen et al

Denmark/SSI-05/2020 EPI_ISL_416140 2020-03-02 Department of Virus and Microbiological Special diagnostics, Statens Serum Institut, Copenhagen, Denmark. Statens Serum Institute Morten Rasmussen et al

England/20100122106/2020 EPI_ISL_415142 2020-03-02 Respiratory Virus Unit, Microbiology Services Colindale, Public Health England Respiratory Virus Unit, Microbiology Services Colindale, Public Health England Monica Galiano et al

England/20104004902/2020 EPI_ISL_417226 2020-03-02 Respiratory Virus Unit, Microbiology Services Colindale, Public Health England Respiratory Virus Unit, Microbiology Services Colindale, Public Health England Monica Galiano et al

England/201040081/2020 EPI_ISL_414525 2020-03-02 Respiratory Virus Unit, Microbiology Services Colindale, Public Health England Respiratory Virus Unit, Microbiology Services Colindale, Public Health England Monica Galiano et al

England/20104008702/2020 EPI_ISL_417230 2020-03-02 Respiratory Virus Unit, Microbiology Services Colindale, Public Health England Respiratory Virus Unit, Microbiology Services Colindale, Public Health England Monica Galiano et al

England/20104009002/2020 EPI_ISL_417233 2020-03-02 Respiratory Virus Unit, Microbiology Services Colindale, Public Health England Respiratory Virus Unit, Microbiology Services Colindale, Public Health England Monica Galiano et al

England/20104009102/2020 EPI_ISL_417234 2020-03-02 Respiratory Virus Unit, Microbiology Services Colindale, Public Health England Respiratory Virus Unit, Microbiology Services Colindale, Public Health England Monica Galiano et al

England/20104013703/2020 EPI_ISL_417235 2020-03-02 Respiratory Virus Unit, Microbiology Services Colindale, Public Health England Respiratory Virus Unit, Microbiology Services Colindale, Public Health England Monica Galiano et al

England/BRIS-12175E/2020 EPI_ISL_440225 2020-03-02 PHE South West Regional Laboratory, National Infection Service Wellcome Sanger Institute for the COVID-19 Genomics UK Consortium Stephanie Hutchings et al

England/CAMB-847C3/2020 EPI_ISL_440494 2020-03-02 Department of Pathology, University of Cambridge Wellcome Sanger Institute for the COVID-19 Genomics UK Consortium Luke W Meredith et al

England/NOTT-10DF8D/2020 EPI_ISL_432900 2020-03-02 Queens Medical Centre, Clinical Microbiology Department / DeepSeq Nottingham COVID-19 Genomics UK (COG-UK) Consortium Gemma Clark et al

France/B2335/2020 EPI_ISL_416504 2020-03-02 CHRU Pontchaillou - Laboratoire de Virologie National Reference Center for Viruses of Respiratory Infections, Institut Pasteur, Paris M√©lnie Albert et al

France/B2336/2020 EPI_ISL_416505 2020-03-02 CHRU Pontchaillou - Laboratoire de Virologie National Reference Center for Viruses of Respiratory Infections, Institut Pasteur, Paris M√©lnie Albert et al

France/HF1795/2020 EPI_ISL_414627 2020-03-02 Centre Hospitalier Compi√®gne Laboratoire de Biologie National Reference Center for Viruses of Respiratory Infections, Institut Pasteur, Paris M√©lnie Albert et al

France/HF1805/2020 EPI_ISL_414628 2020-03-02 Centre Hospitalier Compi√®gne Laboratoire de Biologie National Reference Center for Viruses of Respiratory Infections, Institut Pasteur, Paris M√©lnie Albert et al

France/IDF2075/2020 EPI_ISL_415650 2020-03-02 H√¥pital Instruction des Arm√©es - BEGIN National Reference Center for Viruses of Respiratory Infections, Institut Pasteur, Paris M√©lnie Albert et al

Germany/BavPat2/2020 EPI_ISL_414520 2020-03-02 Bundeswehr Institute of Microbiology Bundeswehr Institute of Microbiology Mathias C Walter et al

Germany/BavPat3/2020 EPI_ISL_414521 2020-03-02 Bundeswehr Institute of Microbiology Bundeswehr Institute of Microbiology Mathias C Walter et al

Iceland/15/2020 EPI_ISL_417800 2020-03-02 The National University Hospital of Iceland deCODE genetics Daniel F Gudbjartsson et al

Iceland/19/2020 EPI_ISL_417817 2020-03-02 The National University Hospital of Iceland deCODE genetics Daniel F Gudbjartsson et al

Iceland/22/2020 EPI_ISL_417734 2020-03-02 The National University Hospital of Iceland deCODE genetics Daniel F Gudbjartsson et al

Iceland/23/2020 EPI_ISL_417840 2020-03-02 The National University Hospital of Iceland deCODE genetics Daniel F Gudbjartsson et al

Iceland/25/2020 EPI_ISL_417693 2020-03-02 The National University Hospital of Iceland deCODE genetics Daniel F Gudbjartsson et al

India/c31/2020 EPI_ISL_426179 2020-03-02 National Influenza Center, Indian Council of Medical Research - National Institute of Virology Indian Council of Medical Research-National Institute of Virology, Microbial Containment Complex Pragya D. Yadav et al

Kuwait/KU09/2020 EPI_ISL_416541 2020-03-02 Dasman Diabetes Institute and Virology Laboratory Ministry of Health Dasman Diabetes Institute Fahd Al-Mulla et al

Kuwait/KU12/2020 EPI_ISL_416458 2020-03-02 Virology laboratory Ministry of Health Kuwait sequenced at Dasman Diabetes Institute Dasman Diabetes Institute Fahd Al-Mulla et al

Kuwait/KU17/2020 EPI_ISL_416542 2020-03-02 Dasman Diabetes Institute Dasman Diabetes Institute Fahd Al-Mulla et al

Kuwait/KU18/2020 EPI_ISL_416543 2020-03-02 Dasman Diabetes Institute Dasman Diabetes Institute Fahd Al-Mulla et al

Netherlands/Blaricum_1364780/2020 EPI_ISL_413566 2020-03-02 MHC Gooi & Vechtstreek Erasmus Medical Center David Nieuwenhuijse et al

Netherlands/Eindhoven_1363782/2020 EPI_ISL_413571 2020-03-02 MHC Brabant Zuidoost Erasmus Medical Center David Nieuwenhuijse et al

Netherlands/Gelderland_1/2020 EPI_ISL_414423 2020-03-02 Dutch COVID-19 response team Erasmus Medical Center David Nieuwenhuijse et al

Netherlands/Hardinxveld_Giessendam_1364806/2020 EPI_ISL_413573 2020-03-02 Dienst Gezondheid & Jeugd Zuid-Holland Zuid Erasmus Medical Center David Nieuwenhuijse et al

Netherlands/Naarden_1364774/2020 EPI_ISL_413577 2020-03-02 MHC Gooi & Vechtstreek Erasmus Medical Center David Nieuwenhuijse et al

Netherlands/NoordBrabant_1/2020 EPI_ISL_414428 2020-03-02 Dutch COVID-19 response team Erasmus Medical Center David Nieuwenhuijse et al

Netherlands/NoordBrabant_11/2020 EPI_ISL_414432 2020-03-02 Dutch COVID-19 response team Erasmus Medical Center David Nieuwenhuijse et al

Netherlands/NoordBrabant_3/2020 EPI_ISL_414429 2020-03-02 Dutch COVID-19 response team Erasmus Medical Center David Nieuwenhuijse et al

Netherlands/NoordBrabant_69/2020 EPI_ISL_422865 2020-03-02 Dutch COVID-19 response team Erasmus Medical Center Bas Oude Munnink et al

Netherlands/Oisterwijk_1364072/2020 EPI_ISL_413580 2020-03-02 MHC Hart voor Brabant Erasmus Medical Center David Nieuwenhuijse et al

Netherlands/Rotterdam_1364040/2020 EPI_ISL_413583 2020-03-02 MHC Rotterdam-Rijnmond Erasmus Medical Center David Nieuwenhuijse et al

Netherlands/Utrecht_1364066/2020 EPI_ISL_413590 2020-03-02 MHC Utrecht Erasmus Medical Center David Nieuwenhuijse et al

Netherlands/Utrecht_5/2020 EPI_ISL_414439 2020-03-02 Dutch COVID-19 response team Erasmus Medical Center David Nieuwenhuijse et al

Netherlands/Zeewolde_1365080/2020 EPI_ISL_413591 2020-03-02 MHC Flevoland Erasmus Medical Center David Nieuwenhuijse et al

Netherlands/ZuidHolland_1/2020 EPI_ISL_414444 2020-03-02 Dutch COVID-19 response team Erasmus Medical Center David Nieuwenhuijse et al

NewZealand/20VR0189/2020 EPI_ISL_416519 2020-03-02 Auckland Hospital Institute of Environmental Science and Research (ESR) Matt Storey et al

Norway/1526/2020 EPI_ISL_417485 2020-03-02 University Hospital of Northern Norway, Department for Microbiology and Infectious Disease Control Norwegian Institute of Public Health, Department of Virology Kathrine Stene-Johansen et al

Senegal/016/2020 EPI_ISL_418207 2020-03-02 Institut Pasteur Dakar Institut Pasteur de Dakar Ndongo Dia et al

Singapore/19/2020 EPI_ISL_419001 2020-03-02 National Public Health Laboratory, National Centre for Infectious Diseases National Public Health Laboratory, National Centre for Infectious Diseases Mak TM et al

Singapore/22/2020 EPI_ISL_420099 2020-03-02 National Centre for Infectious Diseases Programme in Emerging Infectious Diseases, Duke-NUS Medical School Danielle E Anderson et al

Singapore/23/2020 EPI_ISL_420100 2020-03-02 National Centre for Infectious Diseases Programme in Emerging Infectious Diseases, Duke-NUS Medical School Danielle E Anderson et al

Spain/Andalucia201373/2020 EPI_ISL_418244 2020-03-02 HOSPITAL UNIVERSITARIO VIRGEN DE LAS NIEVES Instituto de Salud Carlos III Iglesias-Caballero et al

Spain/Madrid_R10_33/2020 EPI_ISL_417981 2020-03-02 Hospital Universitario Ram√≥n y Cajal Hospital Universitario La Paz Elias Dahdouh et al

Spain/PaisVasco201382/2020 EPI_ISL_418253 2020-03-02 HOSPITAL TXAGORRITXU Instituto de Salud Carlos III Iglesias-Caballero et al

Spain/Valencia15/2020 EPI_ISL_419679 2020-03-02 Servicio de Microbiolog√≠a. Consorcio Hospital General Universitario de Valencia Sequencing and Bioinformatics Service and Molecular Epidemiology Research Group. FISABIO-Public Health Neris Garcia-Gonzalez et al

Switzerland/BS0914/2020 EPI_ISL_415701 2020-03-02 H√¥pitaux universitaires de Gen√®ve Laboratoire de Virologie H√¥pitaux universitaires de Gen√®ve Laboratoire de Virologie Laubscher F. et al

Switzerland/SZ1417/2020 EPI_ISL_415702 2020-03-02 H√¥pitaux universitaires de Gen√®ve Laboratoire de Virologie H√¥pitaux universitaires de Gen√®ve Laboratoire de Virologie Laubscher F. et al

USA/AZ_4811/2020 EPI_ISL_420784 2020-03-02 AZ Department of Health Services Pathogen Discovery, Respiratory Viruses Branch, Division of Viral Diseases, Centers for Disease Control and Prevention Krista Queen et al

USA/CT-Yale-079/2020 EPI_ISL_431080 2020-03-02 Yale COVID-19 Biorepository Grubaugh Lab - Yale School of Public Health Joseph Fauver et al

USA/FL_6318/2020 EPI_ISL_420785 2020-03-02 FL Bureau of Health Laboratories Tampa Pathogen Discovery, Respiratory Viruses Branch, Division of Viral Diseases, Centers for Disease Control and Prevention Krista Queen et al

USA/FL_7169/2020 EPI_ISL_424855 2020-03-02 FL Bureau of Public Health Laboratories-Tampa Pathogen Discovery, Respiratory Viruses Branch, Division of Viral Diseases, Centers for Disease Control and Prevention Yan Li et al

USA/NH_0008/2020 EPI_ISL_420792 2020-03-02 NH Department of Health and Human Services Public Health Labs Pathogen Discovery, Respiratory Viruses Branch, Division of Viral Diseases, Centers for Disease Control and Prevention Krista Queen et al

USA/NY-SURV0144/2020 EPI_ISL_436050 2020-03-02 NYC Department of Health and Mental Hygiene Pathogen Discovery, Respiratory Viruses Branch, Division of Viral Diseases, Centers for Disease Control and Prevention Ying Tao et al

USA/NY_2929/2020 EPI_ISL_420793 2020-03-02 NYC Department of Health and Mental Hygiene Pathogen Discovery, Respiratory Viruses Branch, Division of Viral Diseases, Centers for Disease Control and Prevention Krista Queen et al

USA/WA-S116/2020 EPI_ISL_417169 2020-03-02 Washington State Department of Health Seattle Flu Study Chu etl al et al

USA/WA-S117/2020 EPI_ISL_417170 2020-03-02 Washington State Department of Health Seattle Flu Study Chu etl al et al

USA/WA-S122/2020 EPI_ISL_417175 2020-03-02 Washington State Department of Health Seattle Flu Study Chu etl al et al

USA/WA-S15/2020 EPI_ISL_417068 2020-03-02 Washington State Department of Health Seattle Flu Study Chu etl al et al

USA/WA-S164/2020 EPI_ISL_430202 2020-03-02 Washington State Department of Health Seattle Flu Study Chu et al et al

USA/WA-S19/2020 EPI_ISL_417072 2020-03-02 Washington State Department of Health Seattle Flu Study Chu etl al et al

USA/WA-S20/2020 EPI_ISL_417073 2020-03-02 Washington State Department of Health Seattle Flu Study Chu etl al et al

USA/WA-S21/2020 EPI_ISL_417074 2020-03-02 Washington State Department of Health Seattle Flu Study Chu etl al et al

USA/WA-S22/2020 EPI_ISL_417075 2020-03-02 Washington State Department of Health Seattle Flu Study Chu etl al et al

USA/WA-S23/2020 EPI_ISL_417076 2020-03-02 Washington State Department of Health Seattle Flu Study Chu etl al et al

USA/WA-S238/2020 EPI_ISL_430276 2020-03-02 Washington State Department of Health Seattle Flu Study Chu et al et al

USA/WA-S24/2020 EPI_ISL_417077 2020-03-02 Washington State Department of Health Seattle Flu Study Chu etl al et al

USA/WA-S252/2020 EPI_ISL_430290 2020-03-02 Washington State Department of Health Seattle Flu Study Chu et al et al

USA/WA-S256/2020 EPI_ISL_430294 2020-03-02 Washington State Department of Health Seattle Flu Study Chu et al et al

USA/WA-S26/2020 EPI_ISL_417079 2020-03-02 Washington State Department of Health Seattle Flu Study Chu etl al et al

USA/WA-S28/2020 EPI_ISL_417081 2020-03-02 Washington State Department of Health Seattle Flu Study Chu etl al et al

USA/WA-S29/2020 EPI_ISL_417082 2020-03-02 Washington State Department of Health Seattle Flu Study Chu etl al et al

USA/WA-S32/2020 EPI_ISL_417085 2020-03-02 Washington State Department of Health Seattle Flu Study Chu etl al et al

USA/WA-S34/2020 EPI_ISL_417087 2020-03-02 Washington State Department of Health Seattle Flu Study Chu etl al et al

USA/WA-S35/2020 EPI_ISL_417088 2020-03-02 Washington State Department of Health Seattle Flu Study Chu etl al et al

USA/WA-S36/2020 EPI_ISL_417089 2020-03-02 Washington State Department of Health Seattle Flu Study Chu etl al et al

USA/WA-S59/2020 EPI_ISL_417112 2020-03-02 Washington State Department of Health Seattle Flu Study Chu etl al et al

USA/WA-S91/2020 EPI_ISL_417144 2020-03-02 Washington State Department of Health Seattle Flu Study Chu etl al et al

USA/WA11-UW7/2020 EPI_ISL_413562 2020-03-02 UW Virology Lab UW Virology Lab Pavitra Roychoudhury et al

Australia/NSW14/2020 EPI_ISL_413600 2020-03-03 Centre for Infectious Diseases and Microbiology - Public Health NSW Health Pathology - Institute of Clinical Pathology and Medical Research; Westmead Hospital; University of Sydney Gall et al

Australia/NSW15/2020 EPI_ISL_417383 2020-03-03 Centre for Infectious Diseases and Microbiology Public Health NSW Health Pathology - Institute of Clinical Pathology and Medical Research; Westmead Hospital; University of Sydney Rockett R et al

Australia/NSW22/2020 EPI_ISL_427649 2020-03-03 Centre for Infectious Diseases and Microbiology Public Health NSW Health Pathology - Institute of Clinical Pathology and Medical Research; Westmead Hospital; University of Sydney Timms V et al

Austria/CeMM0149/2020 EPI_ISL_437997 2020-03-03 Center for Virology, Medical University of Vienna Bergthaler laboratory, CeMM Research Center for Molecular Medicine of the Austrian Academy of Sciences Alexandra Popa et al

Austria/CeMM0150/2020 EPI_ISL_437998 2020-03-03 Center for Virology, Medical University of Vienna Bergthaler laboratory, CeMM Research Center for Molecular Medicine of the Austrian Academy of Sciences Alexandra Popa et al

Belgium/DBA-03032/2020 EPI_ISL_416475 2020-03-03 KU Leuven, Clinical and Epidemiological Virology KU Leuven, Clinical and Epidemiological Virology Bert Vanmechelen et al

Belgium/MMJ-03034/2020 EPI_ISL_417429 2020-03-03 KU Leuven, Clinical and Epidemiological Virology KU Leuven, Clinical and Epidemiological Virology Joan Marti-Carerras et al

Belgium/RT-03035/2020 EPI_ISL_417430 2020-03-03 KU Leuven, Clinical and Epidemiological Virology KU Leuven, Clinical and Epidemiological Virology Joan Marti-Carerras et al

Belgium/SN-03031/2020 EPI_ISL_416469 2020-03-03 KU Leuven, Clinical and Epidemiological Virology KU Leuven, Clinical and Epidemiological Virology Bert Vanmechelen et al

Belgium/VLM-03011/2020 EPI_ISL_415153 2020-03-03 KU Leuven, Clinical and Epidemiological Virology KU Leuven, Clinical and Epidemiological Virology Bert Vanmechelen et al

Brazil/SPBR-07/2020 EPI_ISL_416028 2020-03-03 National Influenza Center - Instituto Adolfo Lutz Instituto Adolfo Lutz, Interdiciplinary Procedures Center, Strategic Laboratory Claudio Tavares Sacchi et al

Brazil/SPBR-11/2020 EPI_ISL_416033 2020-03-03 Hospital Israelita Albert Einstein Instituto Adolfo Lutz, Interdiciplinary Procedures Center, Strategic Laboratory Claudio Tavares Sacchi et al

Canada/BC_4078583/2020 EPI_ISL_418824 2020-03-03 BCCDC Public Health Laboratory BCCDC Public Health Laboratory Harrigan et al

Canada/BC_40860/2020 EPI_ISL_415583 2020-03-03 BCCDC Public Health Laboratory BCCDC Public Health Laboratory Harrigan et al

Chile/Santiago-1/2020 EPI_ISL_414579 2020-03-03 Clinica Alemana de Santiago, Chile Instituto de Salud Publica de Chile Andr√©s E. Castillo et al

Denmark/ALAB-SSI29/2020 EPI_ISL_429447 2020-03-03 Department of Virus and Microbiological Special Diagnostics, Statens Serum Institut, Copenhagen, Denmark, Artillerivej 5, 2300 Copenahgen S Albertsen lab, Department of Chemistry and Bioscience, Aalborg University, Denmark Rasmus Kirkegaard et al

Denmark/SSI-09/2020 EPI_ISL_416141 2020-03-03 Department of Virus and Microbiological Special diagnostics, Statens Serum Institut, Copenhagen, Denmark. Statens Serum Institute Morten Rasmussen et al

Denmark/SSI-101/2020 EPI_ISL_415646 2020-03-03 Department of Virus and Microbiological Special diagnostics, Statens Serum Institut, Copenhagen, Denmark. ViFU Morten Rasmussen et al

Denmark/SSI-102/2020 EPI_ISL_415647 2020-03-03 Department of Virus and Microbiological Special diagnostics, Statens Serum Institut, Copenhagen, Denmark. Statens Serum Institute Morten Rasmussen et al

Denmark/SSI-104/2020 EPI_ISL_415648 2020-03-03 Department of Virus and Microbiological Special diagnostics, Statens Serum Institut, Copenhagen, Denmark. ViFU Morten Rasmussen et al

England/20104007503/2020 EPI_ISL_417227 2020-03-03 Respiratory Virus Unit, Microbiology Services Colindale, Public Health England Respiratory Virus Unit, Microbiology Services Colindale, Public Health England Monica Galiano et al

England/20104008402/2020 EPI_ISL_417228 2020-03-03 Respiratory Virus Unit, Microbiology Services Colindale, Public Health England Respiratory Virus Unit, Microbiology Services Colindale, Public Health England Monica Galiano et al

England/20104008802/2020 EPI_ISL_417231 2020-03-03 Respiratory Virus Unit, Microbiology Services Colindale, Public Health England Respiratory Virus Unit, Microbiology Services Colindale, Public Health England Monica Galiano et al

England/201040141/2020 EPI_ISL_414526 2020-03-03 Respiratory Virus Unit, Microbiology Services Colindale, Public Health England Respiratory Virus Unit, Microbiology Services Colindale, Public Health England Monica Galiano et al

England/20106003303/2020 EPI_ISL_417239 2020-03-03 Respiratory Virus Unit, Microbiology Services Colindale, Public Health England Respiratory Virus Unit, Microbiology Services Colindale, Public Health England Monica Galiano et al

England/20106004803/2020 EPI_ISL_417240 2020-03-03 Respiratory Virus Unit, Microbiology Services Colindale, Public Health England Respiratory Virus Unit, Microbiology Services Colindale, Public Health England Monica Galiano et al

England/20106005403/2020 EPI_ISL_417244 2020-03-03 Respiratory Virus Unit, Microbiology Services Colindale, Public Health England Respiratory Virus Unit, Microbiology Services Colindale, Public Health England Monica Galiano et al

England/20106145903/2020 EPI_ISL_417246 2020-03-03 Respiratory Virus Unit, Microbiology Services Colindale, Public Health England Respiratory Virus Unit, Microbiology Services Colindale, Public Health England Monica Galiano et al

England/20108003302/2020 EPI_ISL_417248 2020-03-03 Respiratory Virus Unit, Microbiology Services Colindale, Public Health England Respiratory Virus Unit, Microbiology Services Colindale, Public Health England Monica Galiano et al

England/20108007302/2020 EPI_ISL_417256 2020-03-03 Respiratory Virus Unit, Microbiology Services Colindale, Public Health England Respiratory Virus Unit, Microbiology Services Colindale, Public Health England Monica Galiano et al

England/SHEF-BFCB1/2020 EPI_ISL_416730 2020-03-03 Virology Department, Sheffield Teaching Hospitals NHS Foundation Trust Department of Infection, Immunity and Cardiovascular Disease, The Florey Institute, The Medical School, University of Sheffield Thushan de Silva et al

England/SHEF-BFCC0/2020 EPI_ISL_416731 2020-03-03 Virology Department, Sheffield Teaching Hospitals NHS Foundation Trust Department of Infection, Immunity and Cardiovascular Disease, The Florey Institute, The Medical School, University of Sheffield Thushan de Silva et al

England/SHEF-BFCDF/2020 EPI_ISL_416732 2020-03-03 Virology Department, Sheffield Teaching Hospitals NHS Foundation Trust Department of Infection, Immunity and Cardiovascular Disease, The Florey Institute, The Medical School, University of Sheffield Thushan de Silva et al

England/SHEF-BFD54/2020 EPI_ISL_416740 2020-03-03 Virology Department, Sheffield Teaching Hospitals NHS Foundation Trust Department of Infection, Immunity and Cardiovascular Disease, The Florey Institute, The Medical School, University of Sheffield Thushan de Silva et al

Finland/FIN03032020A/2020 EPI_ISL_413602 2020-03-03 Department of Virology and Immunology, University of Helsinki and Helsinki University Hospital, Huslab Finland Department of Virology, Faculty of Medicine, University of Helsinki, Helsinki, Finland Teemu Smura et al

Finland/FIN03032020B/2020 EPI_ISL_413603 2020-03-03 Department of Virology and Immunology, University of Helsinki and Helsinki University Hospital, Huslab Finland Department of Virology, Faculty of Medicine, University of Helsinki, Helsinki, Finland Teemu Smura et al

Finland/FIN03032020C/2020 EPI_ISL_413604 2020-03-03 Department of Virology and Immunology, University of Helsinki and Helsinki University Hospital, Huslab Finland Department of Virology, Faculty of Medicine, University of Helsinki, Helsinki, Finland Teemu Smura et al

France/B2337/2020 EPI_ISL_416506 2020-03-03 CHRU Pontchaillou - Laboratoire de Virologie National Reference Center for Viruses of Respiratory Infections, Institut Pasteur, Paris M√©lnie Albert et al

France/HF1870/2020 EPI_ISL_414629 2020-03-03 Centre Hospitalier Compi√®gne Laboratoire de Biologie National Reference Center for Viruses of Respiratory Infections, Institut Pasteur, Paris M√©lnie Albert et al

France/HF1871/2020 EPI_ISL_414630 2020-03-03 Centre Hospitalier Compi√®gne Laboratoire de Biologie National Reference Center for Viruses of Respiratory Infections, Institut Pasteur, Paris M√©lnie Albert et al

France/Valence_425/2020 EPI_ISL_416746 2020-03-03 CNR Virus des Infections Respiratoires - France SUD CNR Virus des Infections Respiratoires - France SUD Bal et al

Iceland/30/2020 EPI_ISL_417773 2020-03-03 The National University Hospital of Iceland deCODE genetics Daniel F Gudbjartsson et al

Iceland/31/2020 EPI_ISL_417842 2020-03-03 The National University Hospital of Iceland deCODE genetics Daniel F Gudbjartsson et al

India/763/2020 EPI_ISL_420543 2020-03-03 National Influenza Center, Indian Council of Medical Research - National Institute of Virology Indian Council of Medical Research-National Institute of Virology, Microbial Containment Complex Pragya D. Yadav. Savita Patil et al

India/770/2020 EPI_ISL_420545 2020-03-03 National Influenza Center, Indian Council of Medical Research - National Institute of Virology Indian Council of Medical Research-National Institute of Virology, Microbial Containment Complex Pragya D. Yadav. Savita Patil et al

India/772/2020 EPI_ISL_420547 2020-03-03 National Influenza Center, Indian Council of Medical Research - National Institute of Virology Indian Council of Medical Research-National Institute of Virology, Microbial Containment Complex Pragya D. Yadav. Savita Patil et al

India/773/2020 EPI_ISL_420549 2020-03-03 National Influenza Center, Indian Council of Medical Research - National Institute of Virology Indian Council of Medical Research-National Institute of Virology, Microbial Containment Complex Pragya D. Yadav. Savita Patil et al

India/777/2020 EPI_ISL_420551 2020-03-03 National Influenza Center, Indian Council of Medical Research - National Institute of Virology Indian Council of Medical Research-National Institute of Virology, Microbial Containment Complex Pragya D. Yadav. Savita Patil et al

India/781/2020 EPI_ISL_420553 2020-03-03 National Influenza Center, Indian Council of Medical Research - National Institute of Virology Indian Council of Medical Research-National Institute of Virology, Microbial Containment Complex Pragya D. Yadav. Savita Patil et al

India/c32/2020 EPI_ISL_420555 2020-03-03 National Influenza Center, Indian Council of Medical Research - National Institute of Virology Indian Council of Medical Research-National Institute of Virology, Microbial Containment Complex Pragya D. Yadav. Savita Patil et al

Ireland/Limerick-19934/2020 EPI_ISL_414586 2020-03-03 UCD National Virus Reference Laboratory UCD National Virus Reference Laboratory Michael Carr et al

Italy/UniSR1/2020 EPI_ISL_413489 2020-03-03 Laboratorio di Microbiologia e Virologia, Universit√† Vita-Salute San Raffaele, Milano Laboratorio di Microbiologia e Virologia, Universit√† Vita-Salute San Raffaele, Milano R.A Diotti et al

Italy/UnivPM1/2020 EPI_ISL_417491 2020-03-03 Virology Laboratory, Department of Biomedical Sciences and Public Health, University Politecnica delle Marche Virology and Legal Medicine Laboratories, Department of Biomedical Sciences and Public Health, University Politecnica delle Marche Bagnarelli et al

Netherlands/Limburg_2/2020 EPI_ISL_414424 2020-03-03 Dutch COVID-19 response team Erasmus Medical Center David Nieuwenhuijse et al

Netherlands/Limburg_3/2020 EPI_ISL_414425 2020-03-03 Dutch COVID-19 response team Erasmus Medical Center David Nieuwenhuijse et al

Netherlands/Limburg_4/2020 EPI_ISL_414426 2020-03-03 Dutch COVID-19 response team Erasmus Medical Center David Nieuwenhuijse et al

Netherlands/NA_64/2020 EPI_ISL_422825 2020-03-03 Dutch COVID-19 response team Erasmus Medical Center Bas Oude Munnink et al

Netherlands/NoordBrabant_2/2020 EPI_ISL_414449 2020-03-03 Dutch COVID-19 response team Erasmus Medical Center David Nieuwenhuijse et al

Netherlands/NoordBrabant_36/2020 EPI_ISL_414545 2020-03-03 Dutch COVID-19 response team Erasmus Medical Center David Nieuwenhuijse et al

Netherlands/NoordHolland_1/2020 EPI_ISL_414433 2020-03-03 Dutch COVID-19 response team Erasmus Medical Center David Nieuwenhuijse et al

Netherlands/NoordHolland_2/2020 EPI_ISL_414549 2020-03-03 Dutch COVID-19 response team Erasmus Medical Center David Nieuwenhuijse et al

Netherlands/Nootdorp_1364222/2020 EPI_ISL_413579 2020-03-03 MHC Haaglanden Erasmus Medical Center David Nieuwenhuijse et al

Netherlands/Overijssel_1/2020 EPI_ISL_414434 2020-03-03 Dutch COVID-19 response team Erasmus Medical Center David Nieuwenhuijse et al

Netherlands/Overijssel_2/2020 EPI_ISL_414460 2020-03-03 Dutch COVID-19 response team Erasmus Medical Center David Nieuwenhuijse et al

Netherlands/Rotterdam_1364740/2020 EPI_ISL_413584 2020-03-03 unknown Erasmus Medical Center David Nieuwenhuijse et al

Netherlands/Tilburg_1364286/2020 EPI_ISL_413587 2020-03-03 Foundation Elisabeth-Tweesteden Ziekenhuis Erasmus Medical Center David Nieuwenhuijse et al

Netherlands/Utrecht_1/2020 EPI_ISL_414435 2020-03-03 Dutch COVID-19 response team Erasmus Medical Center David Nieuwenhuijse et al

Netherlands/Utrecht_10/2020 EPI_ISL_414442 2020-03-03 Dutch COVID-19 response team Erasmus Medical Center David Nieuwenhuijse et al

Netherlands/Utrecht_11/2020 EPI_ISL_414443 2020-03-03 Dutch COVID-19 response team Erasmus Medical Center David Nieuwenhuijse et al

Netherlands/Utrecht_2/2020 EPI_ISL_414436 2020-03-03 Dutch COVID-19 response team Erasmus Medical Center David Nieuwenhuijse et al

Netherlands/Utrecht_21/2020 EPI_ISL_422898 2020-03-03 Dutch COVID-19 response team Erasmus Medical Center Bas Oude Munnink et al

Netherlands/Utrecht_3/2020 EPI_ISL_414437 2020-03-03 Dutch COVID-19 response team Erasmus Medical Center David Nieuwenhuijse et al

Netherlands/Utrecht_4/2020 EPI_ISL_414438 2020-03-03 Dutch COVID-19 response team Erasmus Medical Center David Nieuwenhuijse et al

Netherlands/Utrecht_7/2020 EPI_ISL_414440 2020-03-03 Dutch COVID-19 response team Erasmus Medical Center David Nieuwenhuijse et al

Netherlands/Utrecht_8/2020 EPI_ISL_414441 2020-03-03 Dutch COVID-19 response team Erasmus Medical Center David Nieuwenhuijse et al

Netherlands/ZuidHolland_10/2020 EPI_ISL_414446 2020-03-03 Dutch COVID-19 response team Erasmus Medical Center David Nieuwenhuijse et al

Netherlands/ZuidHolland_18/2020 EPI_ISL_414560 2020-03-03 Dutch COVID-19 response team Erasmus Medical Center David Nieuwenhuijse et al

Netherlands/ZuidHolland_20/2020 EPI_ISL_414562 2020-03-03 Dutch COVID-19 response team Erasmus Medical Center David Nieuwenhuijse et al

Netherlands/ZuidHolland_21/2020 EPI_ISL_414563 2020-03-03 Dutch COVID-19 response team Erasmus Medical Center David Nieuwenhuijse et al

Netherlands/ZuidHolland_9/2020 EPI_ISL_414445 2020-03-03 Dutch COVID-19 response team Erasmus Medical Center David Nieuwenhuijse et al

Poland/PL_P1/2020 EPI_ISL_416488 2020-03-03 ViroGenetics - BSL3 Laboratory of Virology; Human Genome Variation Research Group & Genomics Centre MCB; Bioinformatics Research Group Department of Virology ViroGenetics - BSL3 Laboratory of Virology; Human Genome Variation Research Group & Genomics Centre MCB; Bioinformatics Research Group Department of Virology Aleksandra Milewska et al

Portugal/PT0001b/2020 EPI_ISL_417986 2020-03-03 Centro Hospitalar e Universitario de Sao Joao, Porto Instituto Nacional de Saude (INSA) Guiomar et al et al

Portugal/PT0003/2020 EPI_ISL_417987 2020-03-03 Centro Hospitalar e Universitario de Sao Joao, Porto Instituto Nacional de Saude (INSA) Guiomar et al et al

Portugal/PT0025/2020 EPI_ISL_418010 2020-03-03 CHULC - H Curry Cabral Instituto Nacional de Saude (INSA) Guiomar et al et al

Scotland/CVR64/2020 EPI_ISL_425795 2020-03-03 West of Scotland Specialist Virology Centre, NHSGGC / MRC-University of Glasgow Centre for Virus Research COVID-19 Genomics UK (COG-UK) Consortium Ana da Silva Filipe et al

Scotland/CVR77/2020 EPI_ISL_425807 2020-03-03 West of Scotland Specialist Virology Centre, NHSGGC / MRC-University of Glasgow Centre for Virus Research COVID-19 Genomics UK (COG-UK) Consortium Ana da Silva Filipe et al

Senegal/026/2020 EPI_ISL_418209 2020-03-03 Institut Pasteur Dakar Institut Pasteur de Dakar Ndongo Dia et al

Spain/CastillayLeon201372/2020 EPI_ISL_418249 2020-03-03 COMPLEJO ASISTENCIAL UNIVERSITARIO DE BURGOS Instituto de Salud Carlos III Iglesias-Caballero et al

Spain/LaRioja201575/2020 EPI_ISL_419234 2020-03-03 Hospital San Pedro Instituto de Salud Carlos III Iglesias-Caballero et al

Spain/Madrid_H12_13/2020 EPI_ISL_428683 2020-03-03 Hospital Universitario 12 de Octubre Hospital Universitario 12 de Octubre Sara Gonz√°lez et al

Spain/Madrid_R2_15/2020 EPI_ISL_417979 2020-03-03 Hospital Universitario Ram√≥n y Cajal Hospital Universitario La Paz Elias Dahdouh et al

Spain/Madrid_R5_8/2020 EPI_ISL_417980 2020-03-03 Hospital Universitario Ram√≥n y Cajal Hospital Universitario La Paz Elias Dahdouh et al

Spain/PaisVasco201607/2020 EPI_ISL_419240 2020-03-03 HOSPITAL TXAGORRITXU Instituto de Salud Carlos III Iglesias-Caballero et al

Thailand/NIH-1889/2020 EPI_ISL_434697 2020-03-03 unknown National Institute of Health. Department of medical Sciences, Ministry of Public Health, Thailand Pilailuk et al

Thailand/TNIC-1889/2020 EPI_ISL_430837 2020-03-03 n/a Thai National Influenza Center, Department of medical Science, Ministry of Public Health, Thailand Pilailuk et al

USA/GA_1299/2020 EPI_ISL_420787 2020-03-03 GA Department of Public Health Pathogen Discovery, Respiratory Viruses Branch, Division of Viral Diseases, Centers for Disease Control and Prevention Krista Queen et al

USA/GA_1320/2020 EPI_ISL_420786 2020-03-03 GA Department of Public Health Pathogen Discovery, Respiratory Viruses Branch, Division of Viral Diseases, Centers for Disease Control and Prevention Krista Queen et al

USA/NY-Wadsworth-10495-01/2020 EPI_ISL_425224 2020-03-03 Wadsworth Center, New York State Department of Health Wadsworth Center, New York State Department of Health Kirsten St. George et al

USA/NY-Wadsworth-10496-01/2020 EPI_ISL_426025 2020-03-03 Wadsworth Center, New York State Department.of Health Wadsworth Center, New York State Department.of Health Kirsten St. George et al

USA/NY-Wadsworth-10528-01/2020 EPI_ISL_426026 2020-03-03 Wadsworth Center, New York State Department.of Health Wadsworth Center, New York State Department.of Health Kirsten St. George et al

USA/WA-S11/2020 EPI_ISL_416466 2020-03-03 Seattle Flu Study Seattle Flu Study Chu et al et al

USA/WA-S12/2020 EPI_ISL_417065 2020-03-03 Washington State Department of Health Seattle Flu Study Chu etl al et al

USA/WA-S13/2020 EPI_ISL_417066 2020-03-03 Washington State Department of Health Seattle Flu Study Chu etl al et al

USA/WA-S16/2020 EPI_ISL_417069 2020-03-03 Washington State Department of Health Seattle Flu Study Chu etl al et al

USA/WA-S17/2020 EPI_ISL_417070 2020-03-03 Washington State Department of Health Seattle Flu Study Chu etl al et al

USA/WA-S233/2020 EPI_ISL_430271 2020-03-03 Washington State Department of Health Seattle Flu Study Chu et al et al

USA/WA-S250/2020 EPI_ISL_430288 2020-03-03 Washington State Department of Health Seattle Flu Study Chu et al et al

USA/WA-S264/2020 EPI_ISL_430118 2020-03-03 Seattle Flu Study Seattle Flu Study Chu et al et al

USA/WA-S51/2020 EPI_ISL_417104 2020-03-03 Washington State Department of Health Seattle Flu Study Chu etl al et al

USA/WA-S52/2020 EPI_ISL_417105 2020-03-03 Washington State Department of Health Seattle Flu Study Chu etl al et al

USA/WA-S53/2020 EPI_ISL_417106 2020-03-03 Washington State Department of Health Seattle Flu Study Chu etl al et al

USA/WA-S57/2020 EPI_ISL_417110 2020-03-03 Washington State Department of Health Seattle Flu Study Chu etl al et al

USA/WA-S62/2020 EPI_ISL_417115 2020-03-03 Washington State Department of Health Seattle Flu Study Chu etl al et al

USA/WA-S64/2020 EPI_ISL_417117 2020-03-03 Washington State Department of Health Seattle Flu Study Chu etl al et al

USA/WA-S65/2020 EPI_ISL_417118 2020-03-03 Washington State Department of Health Seattle Flu Study Chu etl al et al

USA/WA12-UW8/2020 EPI_ISL_413563 2020-03-03 UW Virology Lab UW Virology Lab Pavitra Roychoudhury et al

Australia/NSW12/2020 EPI_ISL_413598 2020-03-04 Centre for Infectious Diseases and Microbiology - Public Health NSW Health Pathology - Institute of Clinical Pathology and Medical Research; Westmead Hospital; University of Sydney Gray K et al

Australia/NSW13/2020 EPI_ISL_413599 2020-03-04 Centre for Infectious Diseases and Microbiology - Public Health NSW Health Pathology - Institute of Clinical Pathology and Medical Research; Westmead Hospital; University of Sydney Timms et al

Australia/NSW21/2020 EPI_ISL_417386 2020-03-04 Centre for Infectious Diseases and Microbiology Public Health NSW Health Pathology - Institute of Clinical Pathology and Medical Research; Westmead Hospital; University of Sydney Gray K et al

Australia/NSW23/2020 EPI_ISL_427650 2020-03-04 Centre for Infectious Diseases and Microbiology Public Health NSW Health Pathology - Institute of Clinical Pathology and Medical Research; Westmead Hospital; University of Sydney Gall M et al

Australia/NSW66/2020 EPI_ISL_427646 2020-03-04 Centre for Infectious Diseases and Microbiology Public Health NSW Health Pathology - Institute of Clinical Pathology and Medical Research; Westmead Hospital; University of Sydney Timms V et al

Australia/WA06/2020 EPI_ISL_420534 2020-03-04 Department of Microbiology, PathWest QEII Medical Centre Department of Microbiology, PathWest QEII Medical Centre Chisha Sikazwe et al

Austria/CeMM0157/2020 EPI_ISL_438003 2020-03-04 Center for Virology, Medical University of Vienna Bergthaler laboratory, CeMM Research Center for Molecular Medicine of the Austrian Academy of Sciences Alexandra Popa et al

Austria/CeMM0159/2020 EPI_ISL_438005 2020-03-04 Center for Virology, Medical University of Vienna Bergthaler laboratory, CeMM Research Center for Molecular Medicine of the Austrian Academy of Sciences Alexandra Popa et al

Austria/CeMM0160/2020 EPI_ISL_438006 2020-03-04 Center for Virology, Medical University of Vienna Bergthaler laboratory, CeMM Research Center for Molecular Medicine of the Austrian Academy of Sciences Alexandra Popa et al

Belgium/BGM-030444/2020 EPI_ISL_420442 2020-03-04 KU Leuven, Clinical and Epidemiological Virology KU Leuven, Clinical and Epidemiological Virology Joan Marti-Carreras et al

Belgium/DHWM-03041/2020 EPI_ISL_417422 2020-03-04 KU Leuven, Clinical and Epidemiological Virology KU Leuven, Clinical and Epidemiological Virology Joan Marti-Carerras et al

Belgium/DJ-030452/2020 EPI_ISL_420447 2020-03-04 KU Leuven, Clinical and Epidemiological Virology KU Leuven, Clinical and Epidemiological Virology Joan Marti-Carreras et al

Belgium/DVBJ-030468/2020 EPI_ISL_420324 2020-03-04 KU Leuven, Clinical and Epidemiological Virology KU Leuven, Clinical and Epidemiological Virology Joan Marti-Carreras et al

Belgium/GJ-030458/2020 EPI_ISL_420319 2020-03-04 KU Leuven, Clinical and Epidemiological Virology KU Leuven, Clinical and Epidemiological Virology Joan Marti-Carreras et al

Belgium/GSA-030454/2020 EPI_ISL_420448 2020-03-04 KU Leuven, Clinical and Epidemiological Virology KU Leuven, Clinical and Epidemiological Virology Joan Marti-Carreras et al

Belgium/JL-03044/2020 EPI_ISL_418270 2020-03-04 KU Leuven, Clinical and Epidemiological Virology KU Leuven, Clinical and Epidemiological Virology Tony Wawina et al

Belgium/JRH-030459/2020 EPI_ISL_418988 2020-03-04 Institute information KU Leuven, Clinical and Epidemiological Virology Institute information KU Leuven, Clinical and Epidemiological Virology Bert Vanmechelen et al

Belgium/KA-03042/2020 EPI_ISL_417424 2020-03-04 KU Leuven, Clinical and Epidemiological Virology KU Leuven, Clinical and Epidemiological Virology Joan Marti-Carerras et al

Belgium/NL-030447/2020 EPI_ISL_420314 2020-03-04 KU Leuven, Clinical and Epidemiological Virology KU Leuven, Clinical and Epidemiological Virology Joan Marti-Carreras et al

Belgium/RA-030445/2020 EPI_ISL_420443 2020-03-04 KU Leuven, Clinical and Epidemiological Virology KU Leuven, Clinical and Epidemiological Virology Joan Marti-Carreras et al

Belgium/RT-030460/2020 EPI_ISL_418989 2020-03-04 KU Leuven, Clinical and Epidemiological Virology KU Leuven, Clinical and Epidemiological Virology Bert Vanmechelen et al

Belgium/SQ-03043/2020 EPI_ISL_417426 2020-03-04 KU Leuven, Clinical and Epidemiological Virology KU Leuven, Clinical and Epidemiological Virology Joan Marti-Carerras et al

Belgium/VJP-030463/2020 EPI_ISL_420454 2020-03-04 KU Leuven, Clinical and Epidemiological Virology KU Leuven, Clinical and Epidemiological Virology Joan Marti-Carreras et al

Brazil/RJ-314/2020 EPI_ISL_414045 2020-03-04 LACEN RJ - Laborat√≥rio Central de Sa√∫de P√∫blica Noel Nutels Instituto Oswaldo Cruz FIOCRUZ - Laboratory of Respiratory Viruses and Measles (LVRS) Paola Resende et al

Brazil/SPBR-04/2020 EPI_ISL_414017 2020-03-04 Hospital S√£o Joaquim Beneficencia Portuguesa Instituto Adolfo Lutz, Interdiciplinary Procedures Center, Strategic Laboratory Claudio Tavares Sacchi et al

Brazil/SPBR-08/2020 EPI_ISL_416029 2020-03-04 Laboirat√≥rio Fleury Instituto Adolfo Lutz, Interdiciplinary Procedures Center, Strategic Laboratory Claudio Tavares Sacchi et al

Brazil/SPBR-09/2020 EPI_ISL_416031 2020-03-04 National Influenza Center - Instituto Adolfo Lutz Instituto Adolfo Lutz, Interdiciplinary Procedures Center, Strategic Laboratory Claudio Tavares Sacchi et al

Brazil/SPBR-10/2020 EPI_ISL_416032 2020-03-04 National Influenza Center - Instituto Adolfo Lutz Instituto Adolfo Lutz, Interdiciplinary Procedures Center, Strategic Laboratory Claudio Tavares Sacchi et al

Brazil/SPBR-12/2020 EPI_ISL_416034 2020-03-04 Hospital Israelita Albert Einstein Instituto Adolfo Lutz, Interdiciplinary Procedures Center, Strategic Laboratory Claudio Tavares Sacchi et al

Chile/Talca-2/2020 EPI_ISL_414578 2020-03-04 Hospital de Talca, Chile Instituto de Salud Publica de Chile Andr√©s E. Castillo et al

Denmark/ALAB-SSI109/2020 EPI_ISL_429341 2020-03-04 Department of Virus and Microbiological Special Diagnostics, Statens Serum Institut, Copenhagen, Denmark, Artillerivej 5, 2300 Copenahgen S Albertsen lab, Department of Chemistry and Bioscience, Aalborg University, Denmark Rasmus Kirkegaard et al

England/20104003002/2020 EPI_ISL_417222 2020-03-04 Respiratory Virus Unit, Microbiology Services Colindale, Public Health England Respiratory Virus Unit, Microbiology Services Colindale, Public Health England Monica Galiano et al

England/20104008902/2020 EPI_ISL_417232 2020-03-04 Respiratory Virus Unit, Microbiology Services Colindale, Public Health England Respiratory Virus Unit, Microbiology Services Colindale, Public Health England Monica Galiano et al

England/20108007002/2020 EPI_ISL_417255 2020-03-04 Respiratory Virus Unit, Microbiology Services Colindale, Public Health England Respiratory Virus Unit, Microbiology Services Colindale, Public Health England Monica Galiano et al

England/20109050306/2020 EPI_ISL_417264 2020-03-04 Respiratory Virus Unit, Microbiology Services Colindale, Public Health England Respiratory Virus Unit, Microbiology Services Colindale, Public Health England Monica Galiano et al

England/CAMB-8482A/2020 EPI_ISL_440521 2020-03-04 Department of Pathology, University of Cambridge Wellcome Sanger Institute for the COVID-19 Genomics UK Consortium Luke W Meredith et al

England/CAMB-84839/2020 EPI_ISL_440507 2020-03-04 Department of Pathology, University of Cambridge Wellcome Sanger Institute for the COVID-19 Genomics UK Consortium Luke W Meredith et al

England/CAMB-84848/2020 EPI_ISL_440518 2020-03-04 Department of Pathology, University of Cambridge Wellcome Sanger Institute for the COVID-19 Genomics UK Consortium Luke W Meredith et al

England/Sheff01/2020 EPI_ISL_414500 2020-03-04 Virology Department, Sheffield Teaching Hospitals NHS Foundation Trust Department of Infection, Immunity and Cardiovascular Disease, The Florey Institute, The Medical School, University of Sheffield Thushan de Silva et al

Finland/FIN-266/2020 EPI_ISL_414646 2020-03-04 Department of Virology and Immunology, University of Helsinki and Helsinki University Hospital, Huslab Finland Department of Virology, Faculty of Medicine, University of Helsinki, Helsinki, Finland Teemu Smura et al

France/CVL2000/2020 EPI_ISL_418222 2020-03-04 CHRU Bretonneau - Serv. Bacterio-Virol. National Reference Center for Viruses of Respiratory Infections, Institut Pasteur, Paris M√©lanie Albert et al

France/Clermont-Ferrand_650/2020 EPI_ISL_416752 2020-03-04 CHU Gabriel Montpied CNR Virus des Infections Respiratoires - France SUD Bal et al

France/GE1973/2020 EPI_ISL_414631 2020-03-04 H√¥pital Robert Debr√© Laboratoire de Virologie National Reference Center for Viruses of Respiratory Infections, Institut Pasteur, Paris M√©lnie Albert et al

France/GE1977/2020 EPI_ISL_414632 2020-03-04 H√¥pital Robert Debr√© Laboratoire de Virologie National Reference Center for Viruses of Respiratory Infections, Institut Pasteur, Paris M√©lnie Albert et al

France/HF1986/2020 EPI_ISL_414634 2020-03-04 Centre Hospitalier Compi√®gne Laboratoire de Biologie National Reference Center for Viruses of Respiratory Infections, Institut Pasteur, Paris M√©lnie Albert et al

France/HF1988/2020 EPI_ISL_414635 2020-03-04 Centre Hospitalier Compi√®gne Laboratoire de Biologie National Reference Center for Viruses of Respiratory Infections, Institut Pasteur, Paris M√©lnie Albert et al

France/HF1989/2020 EPI_ISL_414636 2020-03-04 Centre Hospitalier Compi√®gne Laboratoire de Biologie National Reference Center for Viruses of Respiratory Infections, Institut Pasteur, Paris M√©lnie Albert et al

France/HF1993/2020 EPI_ISL_414637 2020-03-04 Centre Hospitalier Compi√®gne Laboratoire de Biologie National Reference Center for Viruses of Respiratory Infections, Institut Pasteur, Paris M√©lnie Albert et al

France/HF1995/2020 EPI_ISL_414638 2020-03-04 Centre Hospitalier Compi√®gne Laboratoire de Biologie National Reference Center for Viruses of Respiratory Infections, Institut Pasteur, Paris M√©lnie Albert et al

France/IDF1980/2020 EPI_ISL_414633 2020-03-04 Centre Hospitalier Ren√© Dubois Laboratoire de Microbiologie - B√¢t A National Reference Center for Viruses of Respiratory Infections, Institut Pasteur, Paris M√©lnie Albert et al

France/Lyon_06042/2020 EPI_ISL_417333 2020-03-04 Institut des Agents Infectieux (IAI), Hospices Civils de Lyon CNR Virus des Infections Respiratoires - France SUD Antonin Bal et al

France/Lyon_06056/2020 EPI_ISL_417334 2020-03-04 Institut des Agents Infectieux (IAI), Hospices Civils de Lyon CNR Virus des Infections Respiratoires - France SUD Antonin Bal et al

France/Lyon_487/2020 EPI_ISL_416747 2020-03-04 Institut des Agents Infectieux (IAI) Hospices Civils de Lyon CNR Virus des Infections Respiratoires - France SUD Bal et al

France/Lyon_508/2020 EPI_ISL_416748 2020-03-04 Institut des Agents Infectieux (IAI) Hospices Civils de Lyon CNR Virus des Infections Respiratoires - France SUD Bal et al

France/N2223/2020 EPI_ISL_416494 2020-03-04 Centre Hositalier Universitaire de Rouen Laboratoire de Virologie National Reference Center for Viruses of Respiratory Infections, Institut Pasteur, Paris M√©lnie Albert et al

France/Valence_532/2020 EPI_ISL_416749 2020-03-04 Centre Hospitalier de Valence CNR Virus des Infections Respiratoires - France SUD Bal et al

Iceland/36/2020 EPI_ISL_417844 2020-03-04 The National University Hospital of Iceland deCODE genetics Daniel F Gudbjartsson et al

Iceland/37/2020 EPI_ISL_417681 2020-03-04 The National University Hospital of Iceland deCODE genetics Daniel F Gudbjartsson et al

Iceland/39/2020 EPI_ISL_417677 2020-03-04 The National University Hospital of Iceland deCODE genetics Daniel F Gudbjartsson et al

Iceland/40/2020 EPI_ISL_417846 2020-03-04 The National University Hospital of Iceland deCODE genetics Daniel F Gudbjartsson et al

Iceland/45/2020 EPI_ISL_417715 2020-03-04 The National University Hospital of Iceland deCODE genetics Daniel F Gudbjartsson et al

Ireland/COR-20134/2020 EPI_ISL_414487 2020-03-04 UCD National Virus Reference Laboratory UCD National Virus Reference Laboratory Michael Carr et al

Italy/INMI10/2020 EPI_ISL_424344 2020-03-04 INMI Lazzaro Spallanzani IRCCS Laboratory of Virology, INMI Lazzaro Spallanzani IRCCS Eleonora Lalle et al

Italy/INMI5/2020 EPI_ISL_417923 2020-03-04 INMI Lazzaro Spallanzani IRCCS Laboratory of Virology, INMI Lazzaro Spallanzani IRCCS Francesco Messina et al

Mexico/EdoMex-InDRE_03/2020 EPI_ISL_424667 2020-03-04 Laboratorio Estatal de Salud Publica del Estado de M√©xico Instituto de Diagn√≥stico y Referencia Epidemiol√≥gicos Irma L√≥pez Mart√≠nez et al

Netherlands/Limburg_5/2020 EPI_ISL_414448 2020-03-04 Dutch COVID-19 response team Erasmus Medical Center David Nieuwenhuijse et al

Netherlands/NoordBrabant_16/2020 EPI_ISL_414456 2020-03-04 Dutch COVID-19 response team Erasmus Medical Center David Nieuwenhuijse et al

Netherlands/NoordBrabant_20/2020 EPI_ISL_414529 2020-03-04 Dutch COVID-19 response team Erasmus Medical Center David Nieuwenhuijse et al

Netherlands/NoordBrabant_21/2020 EPI_ISL_414530 2020-03-04 Dutch COVID-19 response team Erasmus Medical Center David Nieuwenhuijse et al

Netherlands/NoordBrabant_22/2020 EPI_ISL_414531 2020-03-04 Dutch COVID-19 response team Erasmus Medical Center David Nieuwenhuijse et al

Netherlands/NoordBrabant_39/2020 EPI_ISL_414548 2020-03-04 Dutch COVID-19 response team Erasmus Medical Center David Nieuwenhuijse et al

Netherlands/Utrecht_12/2020 EPI_ISL_414462 2020-03-04 Dutch COVID-19 response team Erasmus Medical Center David Nieuwenhuijse et al

Netherlands/Utrecht_13/2020 EPI_ISL_414463 2020-03-04 Dutch COVID-19 response team Erasmus Medical Center David Nieuwenhuijse et al

Netherlands/Utrecht_14/2020 EPI_ISL_414464 2020-03-04 Dutch COVID-19 response team Erasmus Medical Center David Nieuwenhuijse et al

Netherlands/ZuidHolland_11/2020 EPI_ISL_414469 2020-03-04 Dutch COVID-19 response team Erasmus Medical Center David Nieuwenhuijse et al

Norway/1694/2020 EPI_ISL_420136 2020-03-04 Akershus University Hospital, Department for Microbiology and Infectious Disease Control Norwegian Institute of Public Health, Department of Virology Kathrine Stene-Johansen et al

Pakistan/Gilgit1/2020 EPI_ISL_417444 2020-03-04 unknown Department of Healthcare Biotechnology Javed et al

Portugal/PT0005/2020 EPI_ISL_417989 2020-03-04 Centro Hospitalar e Universitario de Sao Joao, Porto Instituto Nacional de Saude (INSA) Guiomar et al et al

Portugal/PT0026/2020 EPI_ISL_418011 2020-03-04 CHULC - H Curry Cabral Instituto Nacional de Saude (INSA) Guiomar et al et al

Scotland/CVR05/2020 EPI_ISL_414027 2020-03-04 West of Scotland Specialist Virology Centre, NHSGGC MRC-University of Glasgow Centre for Virus Research Emma Thomson et al

Scotland/CVR72/2020 EPI_ISL_425803 2020-03-04 West of Scotland Specialist Virology Centre, NHSGGC / MRC-University of Glasgow Centre for Virus Research COVID-19 Genomics UK (COG-UK) Consortium Ana da Silva Filipe et al

Scotland/EDB004/2020 EPI_ISL_415629 2020-03-04 Virology Department, Royal Infirmary of Edinburgh, NHS Lothian Virology Department, Royal Infirmary of Edinburgh, NHS Lothian McHugh M et al

Senegal/020/2020 EPI_ISL_418208 2020-03-04 Institut Pasteur Dakar Institut Pasteur de Dakar Ndongo Dia et al

Singapore/24/2020 EPI_ISL_420101 2020-03-04 National Centre for Infectious Diseases Programme in Emerging Infectious Diseases, Duke-NUS Medical School Danielle E Anderson et al

Spain/Canarias201495/2020 EPI_ISL_419233 2020-03-04 Hospital Universitario de Canarias Instituto de Salud Carlos III Iglesias-Caballero et al

Spain/CastillayLeon201437/2020 EPI_ISL_416994 2020-03-04 COMPLEJO ASISTENCIAL UNIVERSITARIO DE BURGOS Instituto de Salud Carlos III Iglesias-Caballero et al

Spain/Madrid201442/2020 EPI_ISL_417010 2020-03-04 FUNDACION JIMENEZ DIAZ Instituto de Salud Carlos III Iglesias-Caballero et al

Spain/Madrid201449/2020 EPI_ISL_418252 2020-03-04 FUNDACION JIMENEZ DIAZ Instituto de Salud Carlos III Iglesias-Caballero et al

Spain/PaisVasco201602/2020 EPI_ISL_419709 2020-03-04 HOSPITAL TXAGORRITXU Instituto de Salud Carlos III Iglesias-Caballero et al

Spain/Valencia8/2020 EPI_ISL_416487 2020-03-04 Servicio de Microbiolog√≠a. Consorcio Hospital General Universitario de Valencia Sequencing and Bioinformatics Service and Molecular Epidemiology Research Group. FISABIO-Public Health Giuseppe D'Auria et al

Sweden/20-50043/2020 EPI_ISL_430860 2020-03-04 Klinisk mikrobiologi Orebro The Public Health Agency of Sweden Martin Sundqvist et al

Sweden/20-50048/2020 EPI_ISL_429136 2020-03-04 Klinisk mikrobiologi Orebro The Public Health Agency of Sweden Martin Sundqvist et al

Sweden/20-50076/2020 EPI_ISL_429137 2020-03-04 Klinisk mikrobiologi Orebro The Public Health Agency of Sweden Martin Sundqvist et al

Switzerland/BE2536/2020 EPI_ISL_415704 2020-03-04 H√¥pitaux universitaires de Gen√®ve Laboratoire de Virologie H√¥pitaux universitaires de Gen√®ve Laboratoire de Virologie Laubscher F. et al

Taiwan/NTU04/2020 EPI_ISL_422407 2020-03-04 Department of Laboratory Medicine, National Taiwan University Hospital Microbial Genomics Core Lab, National Taiwan University Centers of Genomic and Precision Medicine Shiou-Hwei Yeh et al

USA/CA-SCCPHD-UC115/2020 EPI_ISL_435594 2020-03-04 Santa Clara County Public Health Department Chiu Laboratory, University of California, San Francisco Xianding Deng et al

USA/CA-SCCPHD-UC117/2020 EPI_ISL_435596 2020-03-04 Santa Clara County Public Health Department Chiu Laboratory, University of California, San Francisco Xianding Deng et al

USA/CA-SCCPHD-UC16/2020 EPI_ISL_417320 2020-03-04 Santa Clara County Public Health Department Chiu Laboratory, University of California, San Francisco Xianding Deng et al

USA/GA_1445/2020 EPI_ISL_420788 2020-03-04 GA Department of Public Health Pathogen Discovery, Respiratory Viruses Branch, Division of Viral Diseases, Centers for Disease Control and Prevention Krista Queen et al

USA/MA_9704/2020 EPI_ISL_424911 2020-03-04 MA State Public Health Laboratory Pathogen Discovery, Respiratory Viruses Branch, Division of Viral Diseases, Centers for Disease Control and Prevention Ying Tao et al

USA/MA_9889/2020 EPI_ISL_424912 2020-03-04 MA State Public Health Laboratory Pathogen Discovery, Respiratory Viruses Branch, Division of Viral Diseases, Centers for Disease Control and Prevention Ying Tao et al

USA/MD_0026/2020 EPI_ISL_424869 2020-03-04 MD DOH Laboratories Administration Pathogen Discovery, Respiratory Viruses Branch, Division of Viral Diseases, Centers for Disease Control and Prevention Yan Li et al

USA/NJ_3201/2020 EPI_ISL_424901 2020-03-04 NJ Public Health and Environmental Laboratories Pathogen Discovery, Respiratory Viruses Branch, Division of Viral Diseases, Centers for Disease Control and Prevention Ying Tao et al

USA/NY-PV09444/2020 EPI_ISL_450120 2020-03-04 MSHS Clinical Microbiology Laboratories MSHS Pathogen Surveillance Program Ana S. Gonzalez-Reiche et al

USA/NY-SURV052/2020 EPI_ISL_436047 2020-03-04 NYC Department of Health and Mental Hygiene Pathogen Discovery, Respiratory Viruses Branch, Division of Viral Diseases, Centers for Disease Control and Prevention Ying Tao et al

USA/NY-Wadsworth-10683-01/2020 EPI_ISL_426027 2020-03-04 Wadsworth Center, New York State Department.of Health Wadsworth Center, New York State Department.of Health Kirsten St. George et al

USA/NY-Wadsworth-10690-01/2020 EPI_ISL_426028 2020-03-04 Wadsworth Center, New York State Department.of Health Wadsworth Center, New York State Department.of Health Kirsten St. George et al

USA/NY-Wadsworth-10695-01/2020 EPI_ISL_426029 2020-03-04 Wadsworth Center, New York State Department.of Health Wadsworth Center, New York State Department.of Health Kirsten St. George et al

USA/NY-Wadsworth-10703-01/2020 EPI_ISL_426030 2020-03-04 Wadsworth Center, New York State Department.of Health Wadsworth Center, New York State Department.of Health Kirsten St. George et al

USA/NY-Wadsworth-10704-01/2020 EPI_ISL_426031 2020-03-04 Wadsworth Center, New York State Department.of Health Wadsworth Center, New York State Department.of Health Kirsten St. George et al

USA/NY-Wadsworth-10707-01/2020 EPI_ISL_426032 2020-03-04 Wadsworth Center, New York State Department.of Health Wadsworth Center, New York State Department.of Health Kirsten St. George et al

USA/NY-Wadsworth-10935-01/2020 EPI_ISL_426033 2020-03-04 Wadsworth Center, New York State Department.of Health Wadsworth Center, New York State Department.of Health Kirsten St. George et al

USA/NY-Wadsworth-10955-01/2020 EPI_ISL_426034 2020-03-04 Wadsworth Center, New York State Department.of Health Wadsworth Center, New York State Department.of Health Kirsten St. George et al

USA/NY-Wadsworth-10957-02/2020 EPI_ISL_426035 2020-03-04 Wadsworth Center, New York State Department.of Health Wadsworth Center, New York State Department.of Health Kirsten St. George et al

USA/NY-Wadsworth-10958-01/2020 EPI_ISL_426036 2020-03-04 Wadsworth Center, New York State Department.of Health Wadsworth Center, New York State Department.of Health Kirsten St. George et al

USA/NY2-PV08100/2020 EPI_ISL_415151 2020-03-04 MSHS Clinical Microbiology Laboratories MSHS Pathogen Surveillance Program Gopi Patel et al

USA/NY_1922/2020 EPI_ISL_424906 2020-03-04 NYC Department of Health and Mental Hygiene Pathogen Discovery, Respiratory Viruses Branch, Division of Viral Diseases, Centers for Disease Control and Prevention Ying Tao et al

USA/WA-S162/2020 EPI_ISL_430200 2020-03-04 Washington State Department of Health Seattle Flu Study Chu et al et al

USA/WA-S163/2020 EPI_ISL_430201 2020-03-04 Washington State Department of Health Seattle Flu Study Chu et al et al

USA/WA-S241/2020 EPI_ISL_430279 2020-03-04 Washington State Department of Health Seattle Flu Study Chu et al et al

USA/WA-S259/2020 EPI_ISL_430113 2020-03-04 Seattle Flu Study Seattle Flu Study Chu et al et al

USA/WA-S279/2020 EPI_ISL_430133 2020-03-04 Seattle Flu Study Seattle Flu Study Chu et al et al

USA/WA-S38/2020 EPI_ISL_417091 2020-03-04 Washington State Department of Health Seattle Flu Study Chu etl al et al

USA/WA-S39/2020 EPI_ISL_417092 2020-03-04 Washington State Department of Health Seattle Flu Study Chu etl al et al

USA/WA-S49/2020 EPI_ISL_417102 2020-03-04 Washington State Department of Health Seattle Flu Study Chu etl al et al

USA/WA-S63/2020 EPI_ISL_417116 2020-03-04 Washington State Department of Health Seattle Flu Study Chu etl al et al

USA/WA-S67/2020 EPI_ISL_417120 2020-03-04 Washington State Department of Health Seattle Flu Study Chu etl al et al

USA/WA-S68/2020 EPI_ISL_417121 2020-03-04 Washington State Department of Health Seattle Flu Study Chu etl al et al

USA/WA-UW15/2020 EPI_ISL_414363 2020-03-04 UW Virology Lab UW Virology Lab Pavitra Roychoudhury et al

USA/WA-UW27/2020 EPI_ISL_414596 2020-03-04 UW Virology Lab UW Virology Lab Pavitra Roychoudhury et al

USA/WA-UW28/2020 EPI_ISL_414597 2020-03-04 UW Virology Lab UW Virology Lab Pavitra Roychoudhury et al

Australia/NSW18/2020 EPI_ISL_417384 2020-03-05 Centre for Infectious Diseases and Microbiology Public Health NSW Health Pathology - Institute of Clinical Pathology and Medical Research; Westmead Hospital; University of Sydney Eden J-S et al

Australia/NSW25/2020 EPI_ISL_417388 2020-03-05 Centre for Infectious Diseases and Microbiology Public Health NSW Health Pathology - Institute of Clinical Pathology and Medical Research; Westmead Hospital; University of Sydney Gall M et al

Australia/NSW65/2020 EPI_ISL_427645 2020-03-05 Centre for Infectious Diseases and Microbiology Public Health NSW Health Pathology - Institute of Clinical Pathology and Medical Research; Westmead Hospital; University of Sydney Gray K et al

Austria/CeMM0161/2020 EPI_ISL_438007 2020-03-05 Center for Virology, Medical University of Vienna Bergthaler laboratory, CeMM Research Center for Molecular Medicine of the Austrian Academy of Sciences Alexandra Popa et al

Austria/CeMM0162/2020 EPI_ISL_438008 2020-03-05 Center for Virology, Medical University of Vienna Bergthaler laboratory, CeMM Research Center for Molecular Medicine of the Austrian Academy of Sciences Alexandra Popa et al

Belgium/BG-030551/2020 EPI_ISL_418806 2020-03-05 KU Leuven, Clinical and Epidemiological Virology KU Leuven, Clinical and Epidemiological Virology Bert Vanmechelen et al

Belgium/BS-030598/2020 EPI_ISL_420354 2020-03-05 KU Leuven, Clinical and Epidemiological Virology KU Leuven, Clinical and Epidemiological Virology Joan Marti-Carreras et al

Belgium/DBOM-030566/2020 EPI_ISL_420322 2020-03-05 KU Leuven, Clinical and Epidemiological Virology KU Leuven, Clinical and Epidemiological Virology Joan Marti-Carreras et al

Belgium/DD-030593/2020 EPI_ISL_420349 2020-03-05 KU Leuven, Clinical and Epidemiological Virology KU Leuven, Clinical and Epidemiological Virology Joan Marti-Carreras et al

Belgium/DLDJ-030569/2020 EPI_ISL_420325 2020-03-05 KU Leuven, Clinical and Epidemiological Virology KU Leuven, Clinical and Epidemiological Virology Joan Marti-Carreras et al

Belgium/FM-030592/2020 EPI_ISL_420348 2020-03-05 KU Leuven, Clinical and Epidemiological Virology KU Leuven, Clinical and Epidemiological Virology Joan Marti-Carreras et al

Belgium/GE-030573/2020 EPI_ISL_420329 2020-03-05 KU Leuven, Clinical and Epidemiological Virology KU Leuven, Clinical and Epidemiological Virology Joan Marti-Carreras et al

Belgium/GL-030546/2020 EPI_ISL_418792 2020-03-05 KU Leuven, Clinical and Epidemiological Virology KU Leuven, Clinical and Epidemiological Virology Bert Vanmechelen et al

Belgium/GOF-030578/2020 EPI_ISL_420334 2020-03-05 KU Leuven, Clinical and Epidemiological Virology KU Leuven, Clinical and Epidemiological Virology Joan Marti-Carreras et al

Belgium/GS-030549/2020 EPI_ISL_418798 2020-03-05 KU Leuven, Clinical and Epidemiological Virology KU Leuven, Clinical and Epidemiological Virology Bert Vanmechelen et al

Belgium/JK-0305110/2020 EPI_ISL_420367 2020-03-05 KU Leuven, Clinical and Epidemiological Virology KU Leuven, Clinical and Epidemiological Virology Joan Marti-Carreras et al

Belgium/LD-030597/2020 EPI_ISL_420353 2020-03-05 KU Leuven, Clinical and Epidemiological Virology KU Leuven, Clinical and Epidemiological Virology Joan Marti-Carreras et al

Belgium/LY-030575/2020 EPI_ISL_420331 2020-03-05 KU Leuven, Clinical and Epidemiological Virology KU Leuven, Clinical and Epidemiological Virology Joan Marti-Carreras et al

Belgium/MCW-030574/2020 EPI_ISL_420330 2020-03-05 KU Leuven, Clinical and Epidemiological Virology KU Leuven, Clinical and Epidemiological Virology Joan Marti-Carreras et al

Belgium/MF-030546/2020 EPI_ISL_420444 2020-03-05 KU Leuven, Clinical and Epidemiological Virology KU Leuven, Clinical and Epidemiological Virology Joan Marti-Carreras et al

Belgium/PAN-030681/2020 EPI_ISL_420337 2020-03-05 KU Leuven, Clinical and Epidemiological Virology KU Leuven, Clinical and Epidemiological Virology Joan Marti-Carreras et al

Belgium/RJ-030552/2020 EPI_ISL_420317 2020-03-05 KU Leuven, Clinical and Epidemiological Virology KU Leuven, Clinical and Epidemiological Virology Joan Marti-Carreras et al

Belgium/RJL-030588/2020 EPI_ISL_420344 2020-03-05 KU Leuven, Clinical and Epidemiological Virology KU Leuven, Clinical and Epidemiological Virology Joan Marti-Carreras et al

Belgium/SJ-030583/2020 EPI_ISL_420339 2020-03-05 KU Leuven, Clinical and Epidemiological Virology KU Leuven, Clinical and Epidemiological Virology Joan Marti-Carreras et al

Belgium/ULG-3000/2020 EPI_ISL_417004 2020-03-05 Department of Clinical Microbiology GIGA Medical Genomics Durkin Keith et al

Belgium/VDLH-030548/2020 EPI_ISL_418797 2020-03-05 KU Leuven, Clinical and Epidemiological Virology KU Leuven, Clinical and Epidemiological Virology Bert Vanmechelen et al

Belgium/VF-030562/2020 EPI_ISL_420453 2020-03-05 KU Leuven, Clinical and Epidemiological Virology KU Leuven, Clinical and Epidemiological Virology Joan Marti-Carreras et al

Belgium/VS-030542/2020 EPI_ISL_418863 2020-03-05 KU Leuven, Clinical and Epidemiological Virology KU Leuven, Clinical and Epidemiological Virology Bert Vanmechelen et al

Brazil/RJ-352/2020 EPI_ISL_427299 2020-03-05 Instituto Oswaldo Cruz FIOCRUZ - Laboratory of Respiratory Viruses and Measles (LVRS) Instituto Oswaldo Cruz FIOCRUZ - Laboratory of Respiratory Viruses and Measles (LVRS) Paola Resende et al

Brazil/SPBR-13/2020 EPI_ISL_416035 2020-03-05 National Influenza Center - Instituto Adolfo Lutz Instituto Adolfo Lutz, Interdiciplinary Procedures Center, Strategic Laboratory Claudio Tavares Sacchi et al

Canada/BC_8159203/2020 EPI_ISL_418848 2020-03-05 BCCDC Public Health Laboratory BCCDC Public Health Laboratory Harrigan et al

Canada/ON_PHL3350/2020 EPI_ISL_418366 2020-03-05 Public Health Ontario Laboratories Public Health Ontario Laboratories Alireza Eshaghi et al

Canada/ON_PHL8580/2020 EPI_ISL_418330 2020-03-05 Public Health Ontario Laboratories Public Health Ontario Laboratories Alireza Eshaghi et al

Chile/Puerto_Montt_1/2020 EPI_ISL_445246 2020-03-05 HOSPITAL PUERTO MONTT Instituto de Salud Publica de Chile Andr√©s E Castillo et al

Chile/Santiago-2/2020 EPI_ISL_414580 2020-03-05 Clinica Santa Maria, Santiago, Chile Instituto de Salud Publica de Chile Andr√©s E. Castillo et al

England/20108006003/2020 EPI_ISL_417252 2020-03-05 Respiratory Virus Unit, Microbiology Services Colindale, Public Health England Respiratory Virus Unit, Microbiology Services Colindale, Public Health England Monica Galiano et al

England/20109035906/2020 EPI_ISL_417260 2020-03-05 Respiratory Virus Unit, Microbiology Services Colindale, Public Health England Respiratory Virus Unit, Microbiology Services Colindale, Public Health England Monica Galiano et al

England/20109050706/2020 EPI_ISL_417268 2020-03-05 Respiratory Virus Unit, Microbiology Services Colindale, Public Health England Respiratory Virus Unit, Microbiology Services Colindale, Public Health England Monica Galiano et al

England/20109056906/2020 EPI_ISL_417282 2020-03-05 Respiratory Virus Unit, Microbiology Services Colindale, Public Health England Respiratory Virus Unit, Microbiology Services Colindale, Public Health England Monica Galiano et al

England/20109093706/2020 EPI_ISL_417286 2020-03-05 Respiratory Virus Unit, Microbiology Services Colindale, Public Health England Respiratory Virus Unit, Microbiology Services Colindale, Public Health England Monica Galiano et al

England/20110000706/2020 EPI_ISL_417302 2020-03-05 Respiratory Virus Unit, Microbiology Services Colindale, Public Health England Respiratory Virus Unit, Microbiology Services Colindale, Public Health England Monica Galiano et al

Finland/FIN-313/2020 EPI_ISL_414641 2020-03-05 Department of Virology and Immunology, University of Helsinki and Helsinki University Hospital, Huslab Finland Department of Virology, Faculty of Medicine, University of Helsinki, Helsinki, Finland Teemu Smura et al

France/B2340/2020 EPI_ISL_416507 2020-03-05 CHRU Pontchaillou - Laboratoire de Virologie National Reference Center for Viruses of Respiratory Infections, Institut Pasteur, Paris M√©lnie Albert et al

France/B6041/2020 EPI_ISL_443313 2020-03-05 Cabinet M√©dical National Reference Center for Viruses of Respiratory Infections, Institut Pasteur, Paris M√©lanie Albert et al

France/BFC2094/2020 EPI_ISL_415651 2020-03-05 Unknown National Reference Center for Viruses of Respiratory Infections, Institut Pasteur, Paris M√©lnie Albert et al

France/BFC2147/2020 EPI_ISL_415652 2020-03-05 unknown National Reference Center for Viruses of Respiratory Infections, Institut Pasteur, Paris M√©lnie Albert et al

France/Clermont-Ferrand_651/2020 EPI_ISL_416751 2020-03-05 CHU Gabriel Montpied CNR Virus des Infections Respiratoires - France SUD Bal et al

France/HF2039/2020 EPI_ISL_415649 2020-03-05 unknown National Reference Center for Viruses of Respiratory Infections, Institut Pasteur, Paris M√©lnie Albert et al

France/HF2060/2020 EPI_ISL_418223 2020-03-05 Centre Hospitalier Compi√®gne Laboratoire de Biologie National Reference Center for Viruses of Respiratory Infections, Institut Pasteur, Paris M√©lanie Albert et al

Georgia/Tb-273/2020 EPI_ISL_416479 2020-03-05 R. G. Lugar Center for Public Health Research, National Center for Disease Control and Public Health (NCDC) of Georgia. R. G. Lugar Center for Public Health Research, National Center for Disease Control and Public Health (NCDC) of Georgia. Marine Murtskhvaladze et al

Greece/234_31670/2020 EPI_ISL_437894 2020-03-05 Laboratory of Microbiology, Medical School, National and Kapodistrian University of Athens Laboratory of Biology, Department of Medicine, Democritus University of Thrace Kassela K. et al

Iceland/47/2020 EPI_ISL_417683 2020-03-05 The National University Hospital of Iceland deCODE genetics Daniel F Gudbjartsson et al

Iceland/53/2020 EPI_ISL_417851 2020-03-05 The National University Hospital of Iceland deCODE genetics Daniel F Gudbjartsson et al

Luxembourg/LNS0641910/2020 EPI_ISL_419566 2020-03-05 Laboratoire National de Sant√©, Microbiology, Virology Laboratoire National de Sant√©, Microbiology, Epidemiology and Microbial Genomics Anke Wienecke-Baldacchino et al

Malaysia/IMR_WC1170/2020 EPI_ISL_430440 2020-03-05 Institute for Medical Research, Infectious Disease Research Centre, National Institutes of Health, Ministry of Health Malaysia Institute for Medical Research, Infectious Disease Research Centre, National Institutes of Health, Ministry of Health Malaysia Suppiah.J et al

Malaysia/IMR_WC1177/2020 EPI_ISL_430439 2020-03-05 Institute for Medical Research, Infectious Disease Research Centre, National Institutes of Health, Ministry of Health Malaysia Institute for Medical Research Infectious Disease Research Centre, National Institutes of Health, Ministry of Health Malaysia Suppiah.J et al

Netherlands/NoordBrabant_14/2020 EPI_ISL_414454 2020-03-05 Dutch COVID-19 response team Erasmus Medical Center David Nieuwenhuijse et al

Netherlands/NoordBrabant_18/2020 EPI_ISL_414458 2020-03-05 Dutch COVID-19 response team Erasmus Medical Center David Nieuwenhuijse et al

Netherlands/NoordBrabant_23/2020 EPI_ISL_414532 2020-03-05 Dutch COVID-19 response team Erasmus Medical Center David Nieuwenhuijse et al

Netherlands/NoordBrabant_5/2020 EPI_ISL_414430 2020-03-05 Dutch COVID-19 response team Erasmus Medical Center David Nieuwenhuijse et al

Netherlands/ZuidHolland_14/2020 EPI_ISL_414471 2020-03-05 Dutch COVID-19 response team Erasmus Medical Center David Nieuwenhuijse et al

Netherlands/ZuidHolland_19/2020 EPI_ISL_414561 2020-03-05 Dutch COVID-19 response team Erasmus Medical Center David Nieuwenhuijse et al

Norway/1811/2020 EPI_ISL_420139 2020-03-05 Akershus University Hospital, Department for Microbiology and Infectious Disease Control Norwegian Institute of Public Health, Department of Virology Kathrine Stene-Johansen et al

Portugal/PT0004/2020 EPI_ISL_417988 2020-03-05 CHULC - H Curry Cabral Instituto Nacional de Saude (INSA) Guiomar et al et al

Scotland/CVR76/2020 EPI_ISL_425806 2020-03-05 West of Scotland Specialist Virology Centre, NHSGGC / MRC-University of Glasgow Centre for Virus Research COVID-19 Genomics UK (COG-UK) Consortium Ana da Silva Filipe et al

Scotland/EDB002/2020 EPI_ISL_425819 2020-03-05 Virology Department, Royal Infirmary of Edinburgh, NHS Lothian / School of Biological Sciences, University of Edinburgh / Institute of Genetics and Molecular Medicine, University of Edinburgh COVID-19 Genomics UK (COG-UK) Consortium McHugh M et al

Singapore/25/2020 EPI_ISL_420102 2020-03-05 National Centre for Infectious Diseases Programme in Emerging Infectious Diseases, Duke-NUS Medical School Danielle E Anderson et al

Singapore/26/2020 EPI_ISL_420103 2020-03-05 National Centre for Infectious Diseases Programme in Emerging Infectious Diseases, Duke-NUS Medical School Danielle E Anderson et al

Singapore/27/2020 EPI_ISL_420104 2020-03-05 National Centre for Infectious Diseases Programme in Emerging Infectious Diseases, Duke-NUS Medical School Danielle E Anderson et al

Slovenia/808/2020 EPI_ISL_420541 2020-03-05 Institute of Microbiology and Immunology, Faculty of Medicine, University of Ljubljana Institute of Microbiology and Immunology, Faculty of Medicine, University of Ljubljana Toma≈æ Mark Zorec et al

Spain/Andalucia201617/2020 EPI_ISL_419230 2020-03-05 Hospital Universitario Virgen de las Nieves Instituto de Salud Carlos III Iglesias-Caballero et al

Spain/Madrid_H12_1301/2020 EPI_ISL_421171 2020-03-05 Hospital Universitario 12 de Octubre Hospital Universitario 12 de Octubre Esther Viedma et al

Spain/PaisVasco201493/2020 EPI_ISL_419238 2020-03-05 HOSPITAL DE CRUCES. Instituto de Salud Carlos III Iglesias-Caballero et al

Spain/Valencia3/2020 EPI_ISL_414598 2020-03-05 Servicio Microbiologia, Hospital Clinico Universitario, Valencia Sequencing and Bioinformatics Service and Molecular Epidemiology Research Group. FISABIO-Public Health. David Navarro et al

Sweden/20-50087/2020 EPI_ISL_429138 2020-03-05 Klinisk mikrobiologi Orebro The Public Health Agency of Sweden Martin Sundqvist et al

Taiwan/CGMH-CGU-06/2020 EPI_ISL_417519 2020-03-05 Laboratory Medicine Department of Laboratory Medicine, Lin-Kou Chang Gung Memorial Hospital, Taoyuan, Taiwan Kuo-Chien Tsao et al

USA/AZ-TG268893/2020 EPI_ISL_426512 2020-03-05 AZ SPHL, Arizona Department of Health Services TGen North Jolene Bowers et al

USA/CA-CDPH-UC11/2020 EPI_ISL_413931 2020-03-05 California Department of Public Health Chiu Laboratory, University of California, San Francisco Xianding Deng et al

USA/CA-CDPH-UC7/2020 EPI_ISL_413925 2020-03-05 California Department of Public Health Chiu Laboratory, University of California, San Francisco Xianding Deng et al

USA/CA-CDPH-UC9/2020 EPI_ISL_413928 2020-03-05 California Department of Public Health Chiu Laboratory, University of California, San Francisco Xianding Deng et al

USA/CA-SCCPHD-UC122/2020 EPI_ISL_435601 2020-03-05 Santa Clara County Public Health Department Chiu Laboratory, University of California, San Francisco Xianding Deng et al

USA/FL_9590/2020 EPI_ISL_424853 2020-03-05 FL Bureau of Public Health Laboratories-Miami Pathogen Discovery, Respiratory Viruses Branch, Division of Viral Diseases, Centers for Disease Control and Prevention Yan Li et al

USA/FL_9655/2020 EPI_ISL_424854 2020-03-05 FL Bureau of Public Health Laboratories-Miami Pathogen Discovery, Respiratory Viruses Branch, Division of Viral Diseases, Centers for Disease Control and Prevention Yan Li et al

USA/GA_1847/2020 EPI_ISL_424859 2020-03-05 GA Department of Public Health Laboratory Pathogen Discovery, Respiratory Viruses Branch, Division of Viral Diseases, Centers for Disease Control and Prevention Yan Li et al

USA/HI_4970/2020 EPI_ISL_426420 2020-03-05 HI Dept. of Health, State Laboratories Division Pathogen Discovery, Respiratory Viruses Branch, Division of Viral Diseases, Centers for Disease Control and Prevention Anna Uehara et al

USA/MA_0020/2020 EPI_ISL_424843 2020-03-05 MA State Public Health Laboratory Pathogen Discovery, Respiratory Viruses Branch, Division of Viral Diseases, Centers for Disease Control and Prevention Yan Li et al

USA/MA_1355/2020 EPI_ISL_424913 2020-03-05 MA State Public Health Laboratory Pathogen Discovery, Respiratory Viruses Branch, Division of Viral Diseases, Centers for Disease Control and Prevention Ying Tao et al

USA/MA_8932/2020 EPI_ISL_424908 2020-03-05 MA State Public Health Laboratory Pathogen Discovery, Respiratory Viruses Branch, Division of Viral Diseases, Centers for Disease Control and Prevention Ying Tao et al

USA/MA_8933/2020 EPI_ISL_424909 2020-03-05 MA State Public Health Laboratory Pathogen Discovery, Respiratory Viruses Branch, Division of Viral Diseases, Centers for Disease Control and Prevention Ying Tao et al

USA/MA_9703/2020 EPI_ISL_424910 2020-03-05 MA State Public Health Laboratory Pathogen Discovery, Respiratory Viruses Branch, Division of Viral Diseases, Centers for Disease Control and Prevention Ying Tao et al

USA/MN_0100/2020 EPI_ISL_426426 2020-03-05 MN PHL Division, Minnesota Department of Health Pathogen Discovery, Respiratory Viruses Branch, Division of Viral Diseases, Centers for Disease Control and Prevention Krista Queen et al

USA/NE_6605/2020 EPI_ISL_424874 2020-03-05 NE Public Health Laboratory Pathogen Discovery, Respiratory Viruses Branch, Division of Viral Diseases, Centers for Disease Control and Prevention Yan Li et al

USA/NY-SURV076/2020 EPI_ISL_436048 2020-03-05 NYC Department of Health and Mental Hygiene Pathogen Discovery, Respiratory Viruses Branch, Division of Viral Diseases, Centers for Disease Control and Prevention Ying Tao et al

USA/NY-Wadsworth-10999-01/2020 EPI_ISL_426037 2020-03-05 Wadsworth Center, New York State Department.of Health Wadsworth Center, New York State Department.of Health Kirsten St. George et al

USA/NY-Wadsworth-11000-01/2020 EPI_ISL_426038 2020-03-05 Wadsworth Center, New York State Department.of Health Wadsworth Center, New York State Department.of Health Kirsten St. George et al

USA/NY-Wadsworth-11003-01/2020 EPI_ISL_426039 2020-03-05 Wadsworth Center, New York State Department.of Health Wadsworth Center, New York State Department.of Health Kirsten St. George et al

USA/NY-Wadsworth-11017-01/2020 EPI_ISL_426040 2020-03-05 Wadsworth Center, New York State Department.of Health Wadsworth Center, New York State Department.of Health Kirsten St. George et al

USA/PA_1802/2020 EPI_ISL_424881 2020-03-05 PA Department of Health, Bureau of Laboratories Pathogen Discovery, Respiratory Viruses Branch, Division of Viral Diseases, Centers for Disease Control and Prevention Yan Li et al

USA/PA_1881/2020 EPI_ISL_424882 2020-03-05 PA Department of Health, Bureau of Laboratories Pathogen Discovery, Respiratory Viruses Branch, Division of Viral Diseases, Centers for Disease Control and Prevention Yan Li et al

USA/RI_0702/2020 EPI_ISL_424887 2020-03-05 RI State Health Laboratories Pathogen Discovery, Respiratory Viruses Branch, Division of Viral Diseases, Centers for Disease Control and Prevention Yan Li et al

USA/SC_3520/2020 EPI_ISL_424902 2020-03-05 SC Dept of Health and Env. Control-Bureau of Laboratories Pathogen Discovery, Respiratory Viruses Branch, Division of Viral Diseases, Centers for Disease Control and Prevention Ying Tao et al

USA/SC_3572/2020 EPI_ISL_424905 2020-03-05 SC Dept of Health and Env. Control-Bureau of Laboratories Pathogen Discovery, Respiratory Viruses Branch, Division of Viral Diseases, Centers for Disease Control and Prevention Ying Tao et al

USA/UT_8199/2020 EPI_ISL_424889 2020-03-05 UT-Unified State Labs: Public Health Utah DOH Pathogen Discovery, Respiratory Viruses Branch, Division of Viral Diseases, Centers for Disease Control and Prevention Yan Li et al

USA/VT-CDC-0303/2020 EPI_ISL_447846 2020-03-05 VT Dept. of Health Laboratory Pathogen Discovery, Respiratory Viruses Branch, Division of Viral Diseases, Centers for Disease Control and Prevention Krista Queen et al

USA/WA-S104/2020 EPI_ISL_417157 2020-03-05 Washington State Department of Health Seattle Flu Study Chu etl al et al

USA/WA-S110/2020 EPI_ISL_417163 2020-03-05 Seattle Flu Study Seattle Flu Study Chu etl al et al

USA/WA-S114/2020 EPI_ISL_417167 2020-03-05 Washington State Department of Health Seattle Flu Study Chu etl al et al

USA/WA-S263/2020 EPI_ISL_430117 2020-03-05 Seattle Flu Study Seattle Flu Study Chu et al et al

USA/WA-S265/2020 EPI_ISL_430119 2020-03-05 Seattle Flu Study Seattle Flu Study Chu et al et al

USA/WA-S268/2020 EPI_ISL_430122 2020-03-05 Seattle Flu Study Seattle Flu Study Chu et al et al

USA/WA-S292/2020 EPI_ISL_430146 2020-03-05 Seattle Flu Study Seattle Flu Study Chu et al et al

USA/WA-S298/2020 EPI_ISL_430152 2020-03-05 Seattle Flu Study Seattle Flu Study Chu et al et al

USA/WA-S50/2020 EPI_ISL_417103 2020-03-05 Washington State Department of Health Seattle Flu Study Chu etl al et al

USA/WA-S54/2020 EPI_ISL_417107 2020-03-05 Washington State Department of Health Seattle Flu Study Chu etl al et al

USA/WA-S58/2020 EPI_ISL_417111 2020-03-05 Washington State Department of Health Seattle Flu Study Chu etl al et al

USA/WA-S61/2020 EPI_ISL_417114 2020-03-05 Washington State Department of Health Seattle Flu Study Chu etl al et al

USA/WA-S69/2020 EPI_ISL_417122 2020-03-05 Washington State Department of Health Seattle Flu Study Chu etl al et al

USA/WA-S70/2020 EPI_ISL_417123 2020-03-05 Washington State Department of Health Seattle Flu Study Chu etl al et al

USA/WA-S71/2020 EPI_ISL_417124 2020-03-05 Washington State Department of Health Seattle Flu Study Chu etl al et al

USA/WA-S73/2020 EPI_ISL_417126 2020-03-05 Washington State Department of Health Seattle Flu Study Chu etl al et al

USA/WA-S74/2020 EPI_ISL_417127 2020-03-05 Washington State Department of Health Seattle Flu Study Chu etl al et al

USA/WA-S75/2020 EPI_ISL_417128 2020-03-05 Washington State Department of Health Seattle Flu Study Chu etl al et al

USA/WA-S76/2020 EPI_ISL_417129 2020-03-05 Washington State Department of Health Seattle Flu Study Chu etl al et al

USA/WA-S77/2020 EPI_ISL_417130 2020-03-05 Washington State Department of Health Seattle Flu Study Chu etl al et al

USA/WA-S79/2020 EPI_ISL_417132 2020-03-05 Washington State Department of Health Seattle Flu Study Chu etl al et al

USA/WA-S80/2020 EPI_ISL_417133 2020-03-05 Washington State Department of Health Seattle Flu Study Chu etl al et al

USA/WA-S83/2020 EPI_ISL_417136 2020-03-05 Washington State Department of Health Seattle Flu Study Chu etl al et al

USA/WA-UW16/2020 EPI_ISL_414364 2020-03-05 UW Virology Lab UW Virology Lab Pavitra Roychoudhury et al

USA/WA-UW18/2020 EPI_ISL_414366 2020-03-05 UW Virology Lab UW Virology Lab Pavitra Roychoudhury et al

USA/WA-UW19/2020 EPI_ISL_414367 2020-03-05 UW Virology Lab UW Virology Lab Pavitra Roychoudhury et al

USA/WA-UW20/2020 EPI_ISL_414368 2020-03-05 UW Virology Lab UW Virology Lab Pavitra Roychoudhury et al

USA/WA-UW21/2020 EPI_ISL_414369 2020-03-05 UW Virology Lab UW Virology Lab Pavitra Roychoudhury et al

USA/WA-UW24/2020 EPI_ISL_414593 2020-03-05 UW Virology Lab UW Virology Lab Pavitra Roychoudhury et al

USA/WA-UW25/2020 EPI_ISL_414594 2020-03-05 UW Virology Lab UW Virology Lab Pavitra Roychoudhury et al

USA/WA-UW26/2020 EPI_ISL_414595 2020-03-05 UW Virology Lab UW Virology Lab Pavitra Roychoudhury et al

USA/WA-UW40/2020 EPI_ISL_415605 2020-03-05 UW Virology Lab UW Virology Lab Pavitra Roychoudhury et al

USA/WA15-UW11/2020 EPI_ISL_413650 2020-03-05 UW Virology Lab UW Virology Lab Pavitra Roychoudhury et al

USA/WA16-UW12/2020 EPI_ISL_413651 2020-03-05 UW Virology Lab UW Virology Lab Pavitra Roychoudhury et al

USA/WA17-UW13/2020 EPI_ISL_413652 2020-03-05 UW Virology Lab UW Virology Lab Pavitra Roychoudhury et al

USA/WA18-UW14/2020 EPI_ISL_413653 2020-03-05 UW Virology Lab UW Virology Lab Pavitra Roychoudhury et al

Australia/NSW152/2020 EPI_ISL_427648 2020-03-06 Centre for Infectious Diseases and Microbiology Public Health NSW Health Pathology - Institute of Clinical Pathology and Medical Research; Westmead Hospital; University of Sydney Bachmann N et al

Australia/NSW155/2020 EPI_ISL_427647 2020-03-06 Centre for Infectious Diseases and Microbiology Public Health NSW Health Pathology - Institute of Clinical Pathology and Medical Research; Westmead Hospital; University of Sydney Gray K et al

Australia/NSW19/2020 EPI_ISL_417385 2020-03-06 Centre for Infectious Diseases and Microbiology Public Health NSW Health Pathology - Institute of Clinical Pathology and Medical Research; Westmead Hospital; University of Sydney Lam C et al

Australia/NSW24/2020 EPI_ISL_417387 2020-03-06 Centre for Infectious Diseases and Microbiology Public Health NSW Health Pathology - Institute of Clinical Pathology and Medical Research; Westmead Hospital; University of Sydney Timms V et al

Australia/NSW33/2020 EPI_ISL_427654 2020-03-06 Centre for Infectious Diseases and Microbiology Public Health NSW Health Pathology - Institute of Clinical Pathology and Medical Research; Westmead Hospital; University of Sydney Sadsad R et al

Austria/CeMM0005/2020 EPI_ISL_419658 2020-03-06 Center for Virology, Medical University of Vienna Bergthaler laboratory, CeMM Research Center for Molecular Medicine of the Austrian Academy of Sciences Alexandra Popa et al

Austria/CeMM0165/2020 EPI_ISL_438010 2020-03-06 Center for Virology, Medical University of Vienna Bergthaler laboratory, CeMM Research Center for Molecular Medicine of the Austrian Academy of Sciences Alexandra Popa et al

Austria/CeMM0166/2020 EPI_ISL_438011 2020-03-06 Center for Virology, Medical University of Vienna Bergthaler laboratory, CeMM Research Center for Molecular Medicine of the Austrian Academy of Sciences Alexandra Popa et al

Austria/CeMM0167/2020 EPI_ISL_438012 2020-03-06 Center for Virology, Medical University of Vienna Bergthaler laboratory, CeMM Research Center for Molecular Medicine of the Austrian Academy of Sciences Alexandra Popa et al

Belgium/AKM-030670/2020 EPI_ISL_420326 2020-03-06 KU Leuven, Clinical and Epidemiological Virology KU Leuven, Clinical and Epidemiological Virology Joan Marti-Carreras et al

Belgium/BDW-030694/2020 EPI_ISL_420350 2020-03-06 KU Leuven, Clinical and Epidemiological Virology KU Leuven, Clinical and Epidemiological Virology Joan Marti-Carreras et al

Belgium/BM-030687/2020 EPI_ISL_420343 2020-03-06 KU Leuven, Clinical and Epidemiological Virology KU Leuven, Clinical and Epidemiological Virology Joan Marti-Carreras et al

Belgium/CD-030679/2020 EPI_ISL_420335 2020-03-06 KU Leuven, Clinical and Epidemiological Virology KU Leuven, Clinical and Epidemiological Virology Joan Marti-Carreras et al

Belgium/DA-030691/2020 EPI_ISL_420347 2020-03-06 KU Leuven, Clinical and Epidemiological Virology KU Leuven, Clinical and Epidemiological Virology Joan Marti-Carreras et al

Belgium/DAL-030650/2020 EPI_ISL_420446 2020-03-06 KU Leuven, Clinical and Epidemiological Virology KU Leuven, Clinical and Epidemiological Virology Joan Marti-Carreras et al

Belgium/ECC-030686/2020 EPI_ISL_420342 2020-03-06 KU Leuven, Clinical and Epidemiological Virology KU Leuven, Clinical and Epidemiological Virology Joan Marti-Carreras et al

Belgium/LM-030685/2020 EPI_ISL_420341 2020-03-06 KU Leuven, Clinical and Epidemiological Virology KU Leuven, Clinical and Epidemiological Virology Joan Marti-Carreras et al

Belgium/MC-030689/2020 EPI_ISL_420345 2020-03-06 KU Leuven, Clinical and Epidemiological Virology KU Leuven, Clinical and Epidemiological Virology Joan Marti-Carreras et al

Belgium/MJP-0306100/2020 EPI_ISL_420356 2020-03-06 KU Leuven, Clinical and Epidemiological Virology KU Leuven, Clinical and Epidemiological Virology Joan Marti-Carreras et al

Belgium/MJP-030684/2020 EPI_ISL_420340 2020-03-06 KU Leuven, Clinical and Epidemiological Virology KU Leuven, Clinical and Epidemiological Virology Joan Marti-Carreras et al

Belgium/NKR-030645/2020 EPI_ISL_418795 2020-03-06 KU Leuven, Clinical and Epidemiological Virology KU Leuven, Clinical and Epidemiological Virology Bert Vanmechelen et al

Belgium/PA-030680/2020 EPI_ISL_420336 2020-03-06 KU Leuven, Clinical and Epidemiological Virology KU Leuven, Clinical and Epidemiological Virology Joan Marti-Carreras et al

Belgium/RA-030664/2020 EPI_ISL_420441 2020-03-06 KU Leuven, Clinical and Epidemiological Virology KU Leuven, Clinical and Epidemiological Virology Joan Marti-Carreras et al

Belgium/RR-030699/2020 EPI_ISL_420355 2020-03-06 KU Leuven, Clinical and Epidemiological Virology KU Leuven, Clinical and Epidemiological Virology Joan Marti-Carreras et al

Belgium/RS-030677/2020 EPI_ISL_420333 2020-03-06 KU Leuven, Clinical and Epidemiological Virology KU Leuven, Clinical and Epidemiological Virology Joan Marti-Carreras et al

Belgium/RYR-030649/2020 EPI_ISL_420315 2020-03-06 KU Leuven, Clinical and Epidemiological Virology KU Leuven, Clinical and Epidemiological Virology Joan Marti-Carreras et al

Belgium/VBK-03061/2020 EPI_ISL_417425 2020-03-06 KU Leuven, Clinical and Epidemiological Virology KU Leuven, Clinical and Epidemiological Virology Joan Marti-Carerras et al

Belgium/VHC-030655/2020 EPI_ISL_420449 2020-03-06 KU Leuven, Clinical and Epidemiological Virology KU Leuven, Clinical and Epidemiological Virology Joan Marti-Carreras et al

Belgium/VHRA-030644/2020 EPI_ISL_418794 2020-03-06 KU Leuven, Clinical and Epidemiological Virology KU Leuven, Clinical and Epidemiological Virology Bert Vanmechelen et al

Belgium/VMC-030695/2020 EPI_ISL_420351 2020-03-06 KU Leuven, Clinical and Epidemiological Virology KU Leuven, Clinical and Epidemiological Virology Joan Marti-Carreras et al

Belgium/VPE-030650/2020 EPI_ISL_418805 2020-03-06 KU Leuven, Clinical and Epidemiological Virology KU Leuven, Clinical and Epidemiological Virology Bert Vanmechelen et al

Belgium/VRAR-030643/2020 EPI_ISL_418981 2020-03-06 KU Leuven, Clinical and Epidemiological Virology KU Leuven, Clinical and Epidemiological Virology Bert Vanmechelen et al

Belgium/WAM-030676/2020 EPI_ISL_420332 2020-03-06 KU Leuven, Clinical and Epidemiological Virology KU Leuven, Clinical and Epidemiological Virology Joan Marti-Carreras et al

Belgium/WWM-030665/2020 EPI_ISL_420321 2020-03-06 KU Leuven, Clinical and Epidemiological Virology KU Leuven, Clinical and Epidemiological Virology Joan Marti-Carreras et al

Brazil/BA-510/2020 EPI_ISL_427293 2020-03-06 LACEN-BA - Laborat√≥rio Central de Sa√∫de P√∫blica Professor Gon√ßalo Moniz Instituto Oswaldo Cruz FIOCRUZ - Laboratory of Respiratory Viruses and Measles (LVRS) Paola Resende et al

Canada/BC_1318414/2020 EPI_ISL_418818 2020-03-06 BCCDC Public Health Laboratory BCCDC Public Health Laboratory Harrigan et al

Chile/Santiago_op2d1/2020 EPI_ISL_415658 2020-03-06 Laboratory of Molecular Virology, Pontificia Universidad Cat√≥lica de Chile MSHS Pathogen Surveillance Program Rafael A. Medina et al

Denmark/ALAB-SSI-245/2020 EPI_ISL_437660 2020-03-06 Department of Virus and Microbiological Special Diagnostics, Statens Serum Institut, Copenhagen, Denmark, Artillerivej 5, 2300 Copenahgen S Albertsen lab, Department of Chemistry and Bioscience, Aalborg University, Denmark Rasmus Kirkegaard et al

Denmark/ALAB-SSI117/2020 EPI_ISL_429346 2020-03-06 Department of Virus and Microbiological Special Diagnostics, Statens Serum Institut, Copenhagen, Denmark, Artillerivej 5, 2300 Copenahgen S Albertsen lab, Department of Chemistry and Bioscience, Aalborg University, Denmark Rasmus Kirkegaard et al

England/20108034006/2020 EPI_ISL_417258 2020-03-06 Respiratory Virus Unit, Microbiology Services Colindale, Public Health England Respiratory Virus Unit, Microbiology Services Colindale, Public Health England Monica Galiano et al

England/20109039306/2020 EPI_ISL_417262 2020-03-06 Respiratory Virus Unit, Microbiology Services Colindale, Public Health England Respiratory Virus Unit, Microbiology Services Colindale, Public Health England Monica Galiano et al

England/20109050106/2020 EPI_ISL_417263 2020-03-06 Respiratory Virus Unit, Microbiology Services Colindale, Public Health England Respiratory Virus Unit, Microbiology Services Colindale, Public Health England Monica Galiano et al

England/20109051806/2020 EPI_ISL_417270 2020-03-06 Respiratory Virus Unit, Microbiology Services Colindale, Public Health England Respiratory Virus Unit, Microbiology Services Colindale, Public Health England Monica Galiano et al

England/20109052006/2020 EPI_ISL_417272 2020-03-06 Respiratory Virus Unit, Microbiology Services Colindale, Public Health England Respiratory Virus Unit, Microbiology Services Colindale, Public Health England Monica Galiano et al

England/20109058906/2020 EPI_ISL_417283 2020-03-06 Respiratory Virus Unit, Microbiology Services Colindale, Public Health England Respiratory Virus Unit, Microbiology Services Colindale, Public Health England Monica Galiano et al

England/20109093606/2020 EPI_ISL_417285 2020-03-06 Respiratory Virus Unit, Microbiology Services Colindale, Public Health England Respiratory Virus Unit, Microbiology Services Colindale, Public Health England Monica Galiano et al

England/20109094006/2020 EPI_ISL_417289 2020-03-06 Respiratory Virus Unit, Microbiology Services Colindale, Public Health England Respiratory Virus Unit, Microbiology Services Colindale, Public Health England Monica Galiano et al

England/20109094106/2020 EPI_ISL_417290 2020-03-06 Respiratory Virus Unit, Microbiology Services Colindale, Public Health England Respiratory Virus Unit, Microbiology Services Colindale, Public Health England Monica Galiano et al

England/20109099006/2020 EPI_ISL_417297 2020-03-06 Respiratory Virus Unit, Microbiology Services Colindale, Public Health England Respiratory Virus Unit, Microbiology Services Colindale, Public Health England Monica Galiano et al

England/20110000606/2020 EPI_ISL_417301 2020-03-06 Respiratory Virus Unit, Microbiology Services Colindale, Public Health England Respiratory Virus Unit, Microbiology Services Colindale, Public Health England Monica Galiano et al

England/CAMB-84918/2020 EPI_ISL_440491 2020-03-06 Department of Pathology, University of Cambridge Wellcome Sanger Institute for the COVID-19 Genomics UK Consortium Luke W Meredith et al

France/B2343/2020 EPI_ISL_416508 2020-03-06 CHRU Pontchaillou - Laboratoire de Virologie National Reference Center for Viruses of Respiratory Infections, Institut Pasteur, Paris M√©lnie Albert et al

France/B2344/2020 EPI_ISL_416509 2020-03-06 CHRU Pontchaillou - Laboratoire de Virologie National Reference Center for Viruses of Respiratory Infections, Institut Pasteur, Paris M√©lnie Albert et al

France/B2346/2020 EPI_ISL_416510 2020-03-06 CHRU Pontchaillou - Laboratoire de Virologie National Reference Center for Viruses of Respiratory Infections, Institut Pasteur, Paris M√©lnie Albert et al

France/Lyon_06464/2020 EPI_ISL_416753 2020-03-06 Institut des Agents Infectieux (IAI) Hospices Civils de Lyon CNR Virus des Infections Respiratoires - France SUD Bal et al

France/Lyon_06487/2020 EPI_ISL_416754 2020-03-06 Institut des Agents Infectieux (IAI) Hospices Civils de Lyon CNR Virus des Infections Respiratoires - France SUD Bal et al

France/Lyon_06531/2020 EPI_ISL_416756 2020-03-06 Institut des Agents Infectieux (IAI) Hospices Civils de Lyon CNR Virus des Infections Respiratoires - France SUD Bal et al

France/Lyon_06573/2020 EPI_ISL_417335 2020-03-06 Institut des Agents Infectieux (IAI), Hospices Civils de Lyon CNR Virus des Infections Respiratoires - France SUD Antonin Bal et al

France/Lyon_0668/2020 EPI_ISL_417336 2020-03-06 Institut des Agents Infectieux (IAI), Hospices Civils de Lyon CNR Virus des Infections Respiratoires - France SUD Antonin Bal et al

France/Lyon_683/2020 EPI_ISL_416750 2020-03-06 Institut des Agents Infectieux (IAI) Hospices Civils de Lyon CNR Virus des Infections Respiratoires - France SUD Bal et al

Iceland/54/2020 EPI_ISL_417684 2020-03-06 The National University Hospital of Iceland deCODE genetics Daniel F Gudbjartsson et al

Iceland/56/2020 EPI_ISL_417852 2020-03-06 The National University Hospital of Iceland deCODE genetics Daniel F Gudbjartsson et al

Iceland/57/2020 EPI_ISL_417692 2020-03-06 The National University Hospital of Iceland deCODE genetics Daniel F Gudbjartsson et al

Iceland/59/2020 EPI_ISL_417853 2020-03-06 The National University Hospital of Iceland deCODE genetics Daniel F Gudbjartsson et al

Iceland/61/2020 EPI_ISL_417731 2020-03-06 The National University Hospital of Iceland deCODE genetics Daniel F Gudbjartsson et al

Iceland/62/2020 EPI_ISL_417757 2020-03-06 The National University Hospital of Iceland deCODE genetics Daniel F Gudbjartsson et al

Ireland/21023/2020 EPI_ISL_418516 2020-03-06 UCD National Virus Reference Laboratory UCD National Virus Reference Laboratory Michael Carr et al

Ireland/21145/2020 EPI_ISL_418548 2020-03-06 UCD National Virus Reference Laboratory UCD National Virus Reference Laboratory Michael Carr et al

Netherlands/NoordBrabant_12/2020 EPI_ISL_414452 2020-03-06 Dutch COVID-19 response team Erasmus Medical Center David Nieuwenhuijse et al

Netherlands/NoordBrabant_17/2020 EPI_ISL_414457 2020-03-06 Dutch COVID-19 response team Erasmus Medical Center David Nieuwenhuijse et al

Netherlands/NoordBrabant_25/2020 EPI_ISL_414534 2020-03-06 Dutch COVID-19 response team Erasmus Medical Center David Nieuwenhuijse et al

Netherlands/NoordBrabant_26/2020 EPI_ISL_414535 2020-03-06 Dutch COVID-19 response team Erasmus Medical Center David Nieuwenhuijse et al

Netherlands/NoordBrabant_6/2020 EPI_ISL_414451 2020-03-06 Dutch COVID-19 response team Erasmus Medical Center David Nieuwenhuijse et al

Netherlands/ZuidHolland_13/2020 EPI_ISL_414470 2020-03-06 Dutch COVID-19 response team Erasmus Medical Center David Nieuwenhuijse et al

Netherlands/ZuidHolland_16/2020 EPI_ISL_414558 2020-03-06 Dutch COVID-19 response team Erasmus Medical Center David Nieuwenhuijse et al

Netherlands/ZuidHolland_2/2020 EPI_ISL_414556 2020-03-06 Dutch COVID-19 response team Erasmus Medical Center David Nieuwenhuijse et al

Netherlands/ZuidHolland_24/2020 EPI_ISL_414566 2020-03-06 Dutch COVID-19 response team Erasmus Medical Center David Nieuwenhuijse et al

Netherlands/ZuidHolland_8/2020 EPI_ISL_414468 2020-03-06 Dutch COVID-19 response team Erasmus Medical Center David Nieuwenhuijse et al

Portugal/PT0006a/2020 EPI_ISL_417990 2020-03-06 CHULC - H Curry Cabral Instituto Nacional de Saude (INSA) Guiomar et al et al

Portugal/PT0027/2020 EPI_ISL_418012 2020-03-06 CHULC - H Curry Cabral Instituto Nacional de Saude (INSA) Guiomar et al et al

Scotland/CVR65/2020 EPI_ISL_425796 2020-03-06 West of Scotland Specialist Virology Centre, NHSGGC / MRC-University of Glasgow Centre for Virus Research COVID-19 Genomics UK (COG-UK) Consortium Ana da Silva Filipe et al

Scotland/CVR66/2020 EPI_ISL_425797 2020-03-06 West of Scotland Specialist Virology Centre, NHSGGC / MRC-University of Glasgow Centre for Virus Research COVID-19 Genomics UK (COG-UK) Consortium Ana da Silva Filipe et al

Scotland/CVR71/2020 EPI_ISL_425802 2020-03-06 West of Scotland Specialist Virology Centre, NHSGGC / MRC-University of Glasgow Centre for Virus Research COVID-19 Genomics UK (COG-UK) Consortium Ana da Silva Filipe et al

Scotland/CVR75/2020 EPI_ISL_425805 2020-03-06 West of Scotland Specialist Virology Centre, NHSGGC / MRC-University of Glasgow Centre for Virus Research COVID-19 Genomics UK (COG-UK) Consortium Ana da Silva Filipe et al

Scotland/CVR78/2020 EPI_ISL_425808 2020-03-06 West of Scotland Specialist Virology Centre, NHSGGC / MRC-University of Glasgow Centre for Virus Research COVID-19 Genomics UK (COG-UK) Consortium Ana da Silva Filipe et al

Singapore/28/2020 EPI_ISL_420105 2020-03-06 National Centre for Infectious Diseases Programme in Emerging Infectious Diseases, Duke-NUS Medical School Danielle E Anderson et al

Slovakia/SK-BMC1/2020 EPI_ISL_417877 2020-03-06 Institute of Virology, Biomedical Research Center of the Slovak Academy of Sciences, Bratislava; Public Health Authority of the Slovak Republic, Bratislava Institute of Virology, Biomedical Research Center of the Slovak Academy of Sciences, Bratislava; Comenius University Science Park, Bratislava Monika Sl√°vikova et al

Slovakia/SK-BMC5/2020 EPI_ISL_417879 2020-03-06 Institute of Virology, Biomedical Research Center of the Slovak Academy of Sciences, Bratislava; Public Health Authority of the Slovak Republic, Bratislava Institute of Virology, Biomedical Research Center of the Slovak Academy of Sciences, Bratislava; Comenius University Science Park, Bratislava Monika Sl√°vikova et al

SouthAfrica/R02827/2020 EPI_ISL_430297 2020-03-06 National Institute for Communicable Diseases of the National Health Laboratory Service National Institute for Communicable Diseases of the National Health Laboratory Service Allam M et al

Sweden/20-50123/2020 EPI_ISL_429139 2020-03-06 Klinisk mikrobiologi Orebro The Public Health Agency of Sweden Martin Sundqvist et al

Sweden/20-50125/2020 EPI_ISL_429140 2020-03-06 Klinisk mikrobiologi Orebro The Public Health Agency of Sweden Martin Sundqvist et al

Sweden/20-50129/2020 EPI_ISL_430861 2020-03-06 Klinisk mikrobiologi Orebro The Public Health Agency of Sweden Martin Sundqvist et al

Sweden/20-50130/2020 EPI_ISL_429141 2020-03-06 Klinisk mikrobiologi Orebro The Public Health Agency of Sweden Martin Sundqvist et al

Sweden/20-50131/2020 EPI_ISL_429142 2020-03-06 Klinisk mikrobiologi Orebro The Public Health Agency of Sweden Martin Sundqvist et al

Sweden/20-50132/2020 EPI_ISL_429143 2020-03-06 Klinisk mikrobiologi Orebro The Public Health Agency of Sweden Martin Sundqvist et al

Sweden/20-50133/2020 EPI_ISL_429144 2020-03-06 Klinisk mikrobiologi Orebro The Public Health Agency of Sweden Martin Sundqvist et al

Switzerland/GE06207/2020 EPI_ISL_415706 2020-03-06 H√¥pitaux universitaires de Gen√®ve Laboratoire de Virologie H√¥pitaux universitaires de Gen√®ve Laboratoire de Virologie Laubscher F. et al

Switzerland/GE4135/2020 EPI_ISL_415705 2020-03-06 H√¥pitaux universitaires de Gen√®ve Laboratoire de Virologie H√¥pitaux universitaires de Gen√®ve Laboratoire de Virologie Laubscher F. et al

USA/CA-SCCPHD-UC129/2020 EPI_ISL_435608 2020-03-06 Santa Clara County Public Health Department Chiu Laboratory, University of California, San Francisco Xianding Deng et al

USA/CA-SCCPHD-UC130/2020 EPI_ISL_435609 2020-03-06 Santa Clara County Public Health Department Chiu Laboratory, University of California, San Francisco Xianding Deng et al

USA/DC_0004/2020 EPI_ISL_424852 2020-03-06 DC Public Health Lab/ Dept. of Forensic Sciences Pathogen Discovery, Respiratory Viruses Branch, Division of Viral Diseases, Centers for Disease Control and Prevention Yan Li et al

USA/FL_9656/2020 EPI_ISL_424856 2020-03-06 FL Bur. of Public Health Laboratories-Jacksonville Pathogen Discovery, Respiratory Viruses Branch, Division of Viral Diseases, Centers for Disease Control and Prevention Yan Li et al

USA/KS_4126/2020 EPI_ISL_424867 2020-03-06 KS Health and Environmental Laboratories Pathogen Discovery, Respiratory Viruses Branch, Division of Viral Diseases, Centers for Disease Control and Prevention Yan Li et al

USA/MA_2652/2020 EPI_ISL_424914 2020-03-06 MA State Public Health Laboratory Pathogen Discovery, Respiratory Viruses Branch, Division of Viral Diseases, Centers for Disease Control and Prevention Ying Tao et al

USA/MA_3614/2020 EPI_ISL_424915 2020-03-06 MA State Public Health Laboratory Pathogen Discovery, Respiratory Viruses Branch, Division of Viral Diseases, Centers for Disease Control and Prevention Ying Tao et al

USA/MA_3616/2020 EPI_ISL_424916 2020-03-06 MA State Public Health Laboratory Pathogen Discovery, Respiratory Viruses Branch, Division of Viral Diseases, Centers for Disease Control and Prevention Ying Tao et al

USA/MA_3623/2020 EPI_ISL_424917 2020-03-06 MA State Public Health Laboratory Pathogen Discovery, Respiratory Viruses Branch, Division of Viral Diseases, Centers for Disease Control and Prevention Ying Tao et al

USA/MA_3626/2020 EPI_ISL_424918 2020-03-06 MA State Public Health Laboratory Pathogen Discovery, Respiratory Viruses Branch, Division of Viral Diseases, Centers for Disease Control and Prevention Ying Tao et al

USA/MA_3653/2020 EPI_ISL_424920 2020-03-06 MA State Public Health Laboratory Pathogen Discovery, Respiratory Viruses Branch, Division of Viral Diseases, Centers for Disease Control and Prevention Ying Tao et al

USA/MA_3672/2020 EPI_ISL_424844 2020-03-06 MA State Public Health Laboratory Pathogen Discovery, Respiratory Viruses Branch, Division of Viral Diseases, Centers for Disease Control and Prevention Yan Li et al

USA/MA_3699/2020 EPI_ISL_424845 2020-03-06 MA State Public Health Laboratory Pathogen Discovery, Respiratory Viruses Branch, Division of Viral Diseases, Centers for Disease Control and Prevention Yan Li et al

USA/MA_3878/2020 EPI_ISL_424846 2020-03-06 MA State Public Health Laboratory Pathogen Discovery, Respiratory Viruses Branch, Division of Viral Diseases, Centers for Disease Control and Prevention Yan Li et al

USA/MO_4470/2020 EPI_ISL_424870 2020-03-06 MO State Public Health Laboratory Pathogen Discovery, Respiratory Viruses Branch, Division of Viral Diseases, Centers for Disease Control and Prevention Yan Li et al

USA/NC_6999/2020 EPI_ISL_424871 2020-03-06 NC State Laboratory of Public Health Pathogen Discovery, Respiratory Viruses Branch, Division of Viral Diseases, Centers for Disease Control and Prevention Yan Li et al

USA/NH_0029/2020 EPI_ISL_424876 2020-03-06 NH Dept. of Health and Human Services Public Health Labs Pathogen Discovery, Respiratory Viruses Branch, Division of Viral Diseases, Centers for Disease Control and Prevention Yan Li et al

USA/NH_0033/2020 EPI_ISL_424877 2020-03-06 NH Dept. of Health and Human Services Public Health Labs Pathogen Discovery, Respiratory Viruses Branch, Division of Viral Diseases, Centers for Disease Control and Prevention Yan Li et al

USA/NY-Wadsworth-11180-01/2020 EPI_ISL_426041 2020-03-06 Wadsworth Center, New York State Department.of Health Wadsworth Center, New York State Department.of Health Kirsten St. George et al

USA/NY-Wadsworth-11190-01/2020 EPI_ISL_426324 2020-03-06 Wadsworth Center, New York State Department.of Health Wadsworth Center, New York State Department.of Health Kirsten St. George et al

USA/NY-Wadsworth-11191-01/2020 EPI_ISL_426325 2020-03-06 Wadsworth Center, New York State Department.of Health Wadsworth Center, New York State Department.of Health Kirsten St. George et al

USA/NY-Wadsworth-11202-01/2020 EPI_ISL_426042 2020-03-06 Wadsworth Center, New York State Department.of Health Wadsworth Center, New York State Department.of Health Kirsten St. George et al

USA/NY-Wadsworth-11207-01/2020 EPI_ISL_426292 2020-03-06 Wadsworth Center, New York State Department.of Health Wadsworth Center, New York State Department.of Health Kirsten St. George et al

USA/NY-Wadsworth-11249-01/2020 EPI_ISL_426327 2020-03-06 Wadsworth Center, New York State Department.of Health Wadsworth Center, New York State Department.of Health Kirsten St. George et al

USA/NY-Wadsworth-11284-01/2020 EPI_ISL_426043 2020-03-06 Wadsworth Center, New York State Department.of Health Wadsworth Center, New York State Department.of Health Kirsten St. George et al

USA/NY-Wadsworth-11291-01/2020 EPI_ISL_426044 2020-03-06 Wadsworth Center, New York State Department.of Health Wadsworth Center, New York State Department.of Health Kirsten St. George et al

USA/NY-Wadsworth-11344-01/2020 EPI_ISL_426045 2020-03-06 Wadsworth Center, New York State Department.of Health Wadsworth Center, New York State Department.of Health Kirsten St. George et al

USA/NY-Wadsworth-11353-01/2020 EPI_ISL_426046 2020-03-06 Wadsworth Center, New York State Department.of Health Wadsworth Center, New York State Department.of Health Kirsten St. George et al

USA/NY-Wadsworth-11354-01/2020 EPI_ISL_426047 2020-03-06 Wadsworth Center, New York State Department.of Health Wadsworth Center, New York State Department.of Health Kirsten St. George et al

USA/NY-Wadsworth-11379-01/2020 EPI_ISL_426048 2020-03-06 Wadsworth Center, New York State Department.of Health Wadsworth Center, New York State Department.of Health Kirsten St. George et al

USA/NY-Wadsworth-11380-01/2020 EPI_ISL_426049 2020-03-06 Wadsworth Center, New York State Department.of Health Wadsworth Center, New York State Department.of Health Kirsten St. George et al

USA/PA_4405/2020 EPI_ISL_424886 2020-03-06 PA Department of Health, Bureau of Laboratories Pathogen Discovery, Respiratory Viruses Branch, Division of Viral Diseases, Centers for Disease Control and Prevention Yan Li et al

USA/SC_3569/2020 EPI_ISL_424841 2020-03-06 SC Dept of Health and Env. Control-Bureau of Laboratories Pathogen Discovery, Respiratory Viruses Branch, Division of Viral Diseases, Centers for Disease Control and Prevention Yan Li et al

USA/VA_6171/2020 EPI_ISL_424907 2020-03-06 VA-Division of Consolidated Laboratory Services Pathogen Discovery, Respiratory Viruses Branch, Division of Viral Diseases, Centers for Disease Control and Prevention Ying Tao et al

USA/WA-S240/2020 EPI_ISL_430278 2020-03-06 Washington State Department of Health Seattle Flu Study Chu et al et al

USA/WA-S270/2020 EPI_ISL_430124 2020-03-06 Seattle Flu Study Seattle Flu Study Chu et al et al

USA/WA-S275/2020 EPI_ISL_430129 2020-03-06 Seattle Flu Study Seattle Flu Study Chu et al et al

USA/WA-S287/2020 EPI_ISL_430141 2020-03-06 Seattle Flu Study Seattle Flu Study Chu et al et al

USA/WA-S288/2020 EPI_ISL_430142 2020-03-06 Seattle Flu Study Seattle Flu Study Chu et al et al

USA/WA-S66/2020 EPI_ISL_417119 2020-03-06 Washington State Department of Health Seattle Flu Study Chu etl al et al

USA/WA-S72/2020 EPI_ISL_417125 2020-03-06 Washington State Department of Health Seattle Flu Study Chu etl al et al

USA/WA-UW17/2020 EPI_ISL_414365 2020-03-06 UW Virology Lab UW Virology Lab Pavitra Roychoudhury et al

USA/WA-UW22/2020 EPI_ISL_414591 2020-03-06 UW Virology Lab UW Virology Lab Pavitra Roychoudhury et al

USA/WA-UW23/2020 EPI_ISL_414592 2020-03-06 UW Virology Lab UW Virology Lab Pavitra Roychoudhury et al

USA/WA-UW37/2020 EPI_ISL_416454 2020-03-06 UW Virology Lab UW Virology Lab Pavitra Roychoudhury et al

USA/WA-UW39/2020 EPI_ISL_416456 2020-03-06 UW Virology Lab UW Virology Lab Pavitra Roychoudhury et al

Vietnam/CM295/2020 EPI_ISL_416430 2020-03-06 National Influenza Center, National Institute of Hygiene and Epidemiology (NIHE) National Influenza Center, National Institute of Hygiene and Epidemiology (NIHE) Le Quynh Mai et al

Vietnam/CM296/2020 EPI_ISL_416431 2020-03-06 National Influenza Center, National Institute of Hygiene and Epidemiology (NIHE) National Influenza Center, National Institute of Hygiene and Epidemiology (NIHE) Le Quynh Mai et al

Wales/PHW06/2020 EPI_ISL_415435 2020-03-06 Wales Specialist Virology Centre Public Health Wales Microbiology Cardiff Catherine Moore et al

Yunnan/0306-466/2020 EPI_ISL_429239 2020-03-06 Department of Clinical Laboratory, the First People's Hospital of Yunnan Province Department of Clinical Laboratory, the First People's Hospital of Yunnan Province Yi Sun et al

Argentina/C121/2020 EPI_ISL_420600 2020-03-07 Servicio Virosis Respiratorias-Departamento Virolog√≠a-INEI Instituto Nacional Enfermedades Infecciosas C.G.Malbran Baumeister E. et al

Australia/VIC-CBA4/2020 EPI_ISL_430064 2020-03-07 Geelong Centre for Emerging Infectious Diseases Geelong Centre for Emerging Infectious Diseases Chamings A. et al

Australia/VIC17/2020 EPI_ISL_419736 2020-03-07 Victorian Infectious Diseases Reference Laboratory (VIDRL) Victorian Infectious Diseases Reference Laboratory and Microbiological Diagnostic Unit Public Health Laboratory, Doherty Institute Caly L. et al

Australia/VIC18/2020 EPI_ISL_419737 2020-03-07 Victorian Infectious Diseases Reference Laboratory (VIDRL) Victorian Infectious Diseases Reference Laboratory and Microbiological Diagnostic Unit Public Health Laboratory, Doherty Institute Caly L. et al

Austria/CeMM0168/2020 EPI_ISL_438013 2020-03-07 Center for Virology, Medical University of Vienna Bergthaler laboratory, CeMM Research Center for Molecular Medicine of the Austrian Academy of Sciences Alexandra Popa et al

Austria/CeMM0169/2020 EPI_ISL_438014 2020-03-07 Center for Virology, Medical University of Vienna Bergthaler laboratory, CeMM Research Center for Molecular Medicine of the Austrian Academy of Sciences Alexandra Popa et al

Austria/CeMM0171/2020 EPI_ISL_438016 2020-03-07 Center for Virology, Medical University of Vienna Bergthaler laboratory, CeMM Research Center for Molecular Medicine of the Austrian Academy of Sciences Alexandra Popa et al

Belgium/BJ-030767/2020 EPI_ISL_420323 2020-03-07 KU Leuven, Clinical and Epidemiological Virology KU Leuven, Clinical and Epidemiological Virology Joan Marti-Carreras et al

Belgium/DCS-030796/2020 EPI_ISL_420352 2020-03-07 KU Leuven, Clinical and Epidemiological Virology KU Leuven, Clinical and Epidemiological Virology Joan Marti-Carreras et al

Belgium/HC-030760/2020 EPI_ISL_420451 2020-03-07 KU Leuven, Clinical and Epidemiological Virology KU Leuven, Clinical and Epidemiological Virology Joan Marti-Carreras et al

Belgium/HL-030771/2020 EPI_ISL_420327 2020-03-07 KU Leuven, Clinical and Epidemiological Virology KU Leuven, Clinical and Epidemiological Virology Joan Marti-Carreras et al

Belgium/KN-030756/2020 EPI_ISL_420450 2020-03-07 KU Leuven, Clinical and Epidemiological Virology KU Leuven, Clinical and Epidemiological Virology Joan Marti-Carreras et al

Belgium/QOJ-030751/2020 EPI_ISL_420316 2020-03-07 KU Leuven, Clinical and Epidemiological Virology KU Leuven, Clinical and Epidemiological Virology Joan Marti-Carreras et al

Belgium/TG-030757/2020 EPI_ISL_420318 2020-03-07 KU Leuven, Clinical and Epidemiological Virology KU Leuven, Clinical and Epidemiological Virology Joan Marti-Carreras et al

Belgium/ULG-3665/2020 EPI_ISL_417009 2020-03-07 Department of Clinical Microbiology GIGA Medical Genomics Durkin Keith et al

Canada/BC_8718874/2020 EPI_ISL_418852 2020-03-07 BCCDC Public Health Laboratory BCCDC Public Health Laboratory Harrigan et al

Canada/ON_PHL0743/2020 EPI_ISL_418346 2020-03-07 Public Health Ontario Laboratories Public Health Ontario Laboratories Alireza Eshaghi et al

Canada/ON_PHL2653/2020 EPI_ISL_418371 2020-03-07 Public Health Ontario Laboratories Public Health Ontario Laboratories Alireza Eshaghi et al

Canada/ON_PHL3650/2020 EPI_ISL_418349 2020-03-07 Public Health Ontario Laboratories Public Health Ontario Laboratories Alireza Eshaghi et al

Canada/ON_PHL4069/2020 EPI_ISL_418334 2020-03-07 Public Health Ontario Laboratories Public Health Ontario Laboratories Alireza Eshaghi et al

Chile/Santiago_op3d1/2020 EPI_ISL_415660 2020-03-07 Laboratory of Molecular Virology, Pontificia Universidad Cat√≥lica de Chile MSHS Pathogen Surveillance Program Rafael A. Medina et al

Denmark/ALAB-SSI107/2020 EPI_ISL_429339 2020-03-07 Department of Virus and Microbiological Special Diagnostics, Statens Serum Institut, Copenhagen, Denmark, Artillerivej 5, 2300 Copenahgen S Albertsen lab, Department of Chemistry and Bioscience, Aalborg University, Denmark Rasmus Kirkegaard et al

Denmark/ALAB-SSI108/2020 EPI_ISL_429340 2020-03-07 Department of Virus and Microbiological Special Diagnostics, Statens Serum Institut, Copenhagen, Denmark, Artillerivej 5, 2300 Copenahgen S Albertsen lab, Department of Chemistry and Bioscience, Aalborg University, Denmark Rasmus Kirkegaard et al

England/20109047803/2020 EPI_ISL_418667 2020-03-07 Respiratory Virus Unit, Microbiology Services Colindale, Public Health England Respiratory Virus Unit, Microbiology Services Colindale, Public Health England Monica Galiano et al

England/20109052506/2020 EPI_ISL_417276 2020-03-07 Respiratory Virus Unit, Microbiology Services Colindale, Public Health England Respiratory Virus Unit, Microbiology Services Colindale, Public Health England Monica Galiano et al

England/20109053606/2020 EPI_ISL_417279 2020-03-07 Respiratory Virus Unit, Microbiology Services Colindale, Public Health England Respiratory Virus Unit, Microbiology Services Colindale, Public Health England Monica Galiano et al

England/20109054806/2020 EPI_ISL_417280 2020-03-07 Respiratory Virus Unit, Microbiology Services Colindale, Public Health England Respiratory Virus Unit, Microbiology Services Colindale, Public Health England Monica Galiano et al

England/SHEF-BFCEE/2020 EPI_ISL_416733 2020-03-07 Virology Department, Sheffield Teaching Hospitals NHS Foundation Trust Department of Infection, Immunity and Cardiovascular Disease, The Florey Institute, The Medical School, University of Sheffield Thushan de Silva et al

Finland/FIN-508/2020 EPI_ISL_414643 2020-03-07 Department of Virology and Immunology, University of Helsinki and Helsinki University Hospital, Huslab Finland Department of Virology, Faculty of Medicine, University of Helsinki, Helsinki, Finland Teemu Smura et al

France/B2348/2020 EPI_ISL_416511 2020-03-07 CHRU Pontchaillou - Laboratoire de Virologie National Reference Center for Viruses of Respiratory Infections, Institut Pasteur, Paris M√©lnie Albert et al

France/B2349/2020 EPI_ISL_416512 2020-03-07 CHRU Pontchaillou - Laboratoire de Virologie National Reference Center for Viruses of Respiratory Infections, Institut Pasteur, Paris M√©lnie Albert et al

France/B2351/2020 EPI_ISL_416513 2020-03-07 CHRU Pontchaillou - Laboratoire de Virologie National Reference Center for Viruses of Respiratory Infections, Institut Pasteur, Paris M√©lnie Albert et al

France/Bourg-en-Bresse_06678/2020 EPI_ISL_416757 2020-03-07 Centre Hospitalier de Bourg en Bresse CNR Virus des Infections Respiratoires - France SUD Bal et al

France/Bourg-en-Bresse_06813/2020 EPI_ISL_417340 2020-03-07 Centre Hospitalier de Bourg en Bresse CNR Virus des Infections Respiratoires - France SUD Antonin Bal et al

France/Lyon_06625/2020 EPI_ISL_417337 2020-03-07 Institut des Agents Infectieux (IAI), Hospices Civils de Lyon CNR Virus des Infections Respiratoires - France SUD Antonin Bal et al

France/Macon_06756/2020 EPI_ISL_417338 2020-03-07 Centre Hospitalier de Macon CNR Virus des Infections Respiratoires - France SUD Antonin Bal et al

Germany/BAV-MVP0009/2020 EPI_ISL_437210 2020-03-07 Max von Pettenkofer Institute, Virology, National Reference Center for Retroviruses, LMU M√ºnchen Laboratory for Functional Genome Analysis, Dept. Genomics, Gene Center of the LMU Munich Max Muenchhoff et al

Greece/236_31815/2020 EPI_ISL_437895 2020-03-07 Laboratory of Microbiology, Medical School, National and Kapodistrian University of Athens Laboratory of Biology, Department of Medicine, Democritus University of Thrace Kassela K. et al

Iceland/63/2020 EPI_ISL_417855 2020-03-07 The National University Hospital of Iceland deCODE genetics Daniel F Gudbjartsson et al

Iceland/65/2020 EPI_ISL_417705 2020-03-07 The National University Hospital of Iceland deCODE genetics Daniel F Gudbjartsson et al

Iceland/66/2020 EPI_ISL_417857 2020-03-07 The National University Hospital of Iceland deCODE genetics Daniel F Gudbjartsson et al

Italy/INMI8/2020 EPI_ISL_424342 2020-03-07 INMI Lazzaro Spallanzani IRCCS Laboratory of Virology, INMI Lazzaro Spallanzani IRCCS Concetta Castilletti et al

Luxembourg/LNS0945359/2020 EPI_ISL_419569 2020-03-07 Laboratoire National de Sant√©, Microbiology, Virology Laboratoire National de Sant√©, Microbiology, Epidemiology and Microbial Genomics Anke Wienecke-Baldacchino et al

Netherlands/NA_34/2020 EPI_ISL_415491 2020-03-07 Dutch COVID-19 response team Erasmus Medical Center David Nieuwenhuijse et al

Netherlands/NA_43/2020 EPI_ISL_422804 2020-03-07 Dutch COVID-19 response team Erasmus Medical Center Bas Oude Munnink et al

Netherlands/ZuidHolland_17/2020 EPI_ISL_414559 2020-03-07 Dutch COVID-19 response team Erasmus Medical Center David Nieuwenhuijse et al

Portugal/PT0007/2020 EPI_ISL_417992 2020-03-07 CHULC - H D Estefania Instituto Nacional de Saude (INSA) Guiomar et al et al

Portugal/PT0012/2020 EPI_ISL_417997 2020-03-07 Centro Hospital do Porto, E.P.E. - H. Geral de Santo Antonio Instituto Nacional de Saude (INSA) Guiomar et al et al

Portugal/PT0014/2020 EPI_ISL_417999 2020-03-07 Centro Hospital do Porto, E.P.E. - H. Geral de Santo Antonio Instituto Nacional de Saude (INSA) Guiomar et al et al

Portugal/PT0028/2020 EPI_ISL_418013 2020-03-07 CHULC - H Curry Cabral Instituto Nacional de Saude (INSA) Guiomar et al et al

SaudiArabia/KAIMRC-Alghoribi/2020 EPI_ISL_416432 2020-03-07 Clinical Microbiology Lab Infectious Disease Research Department, King Abdullah International Medical Research Center (KAIMRC) Majed Alghoribi et al

Scotland/CVR74/2020 EPI_ISL_425804 2020-03-07 West of Scotland Specialist Virology Centre, NHSGGC / MRC-University of Glasgow Centre for Virus Research COVID-19 Genomics UK (COG-UK) Consortium Ana da Silva Filipe et al

Slovakia/SK-BMC2/2020 EPI_ISL_417878 2020-03-07 Institute of Virology, Biomedical Research Center of the Slovak Academy of Sciences, Bratislava; Public Health Authority of the Slovak Republic, Bratislava Institute of Virology, Biomedical Research Center of the Slovak Academy of Sciences, Bratislava; Comenius University Science Park, Bratislava Monika Sl√°vikova et al

SouthAfrica/R03006/2020 EPI_ISL_417186 2020-03-07 National Institute for Communicable Diseases of the National Health Laboratory Service National Institute for Communicable Diseases of the National Health Laboratory Service Allam M et al

Spain/Galicia201663/2020 EPI_ISL_417007 2020-03-07 HOSPITAL SANTA MARIA NAI Instituto de Salud Carlos III Iglesias-Caballero et al

Spain/Madrid201706/2020 EPI_ISL_419235 2020-03-07 Fundacion Jimenez Diaz Instituto de Salud Carlos III Iglesias-Caballero et al

Spain/Madrid201709/2020 EPI_ISL_419236 2020-03-07 Fundacion Jimenez Diaz Instituto de Salud Carlos III Iglesias-Caballero et al

Spain/Madrid201738/2020 EPI_ISL_419237 2020-03-07 Fundacion Jimenez Diaz Instituto de Salud Carlos III Iglesias-Caballero et al

Spain/Madrid_LP19_4952/2020 EPI_ISL_428676 2020-03-07 Hospital Universitario La Paz Hospital Universitario 12 de Octubre Elias Dahdouh et al

Spain/Valencia40/2020 EPI_ISL_421515 2020-03-07 Servicio de Microbiologia. Hospital Clinico Universitario de Valencia Sequencing and Bioinformatics Service and Molecular Epidemiology Research Group. FISABIO-Public Health Giuseppe D'Auria et al

Switzerland/GE4984/2020 EPI_ISL_415708 2020-03-07 H√¥pitaux universitaires de Gen√®ve Laboratoire de Virologie H√¥pitaux universitaires de Gen√®ve Laboratoire de Virologie Laubscher F. et al

USA/CA-SCCPHD-UC133/2020 EPI_ISL_435612 2020-03-07 Santa Clara County Public Health Department Chiu Laboratory, University of California, San Francisco Xianding Deng et al

USA/CA-SCCPHD-UC136/2020 EPI_ISL_435615 2020-03-07 Santa Clara County Public Health Department Chiu Laboratory, University of California, San Francisco Xianding Deng et al

USA/GA_2070/2020 EPI_ISL_426417 2020-03-07 GA Department of Public Health Laboratory Pathogen Discovery, Respiratory Viruses Branch, Division of Viral Diseases, Centers for Disease Control and Prevention Anna Uehara et al

USA/HI_7881/2020 EPI_ISL_426421 2020-03-07 HI Dept. of Health, State Laboratories Division Pathogen Discovery, Respiratory Viruses Branch, Division of Viral Diseases, Centers for Disease Control and Prevention Anna Uehara et al

USA/IA-CDC-8200/2020 EPI_ISL_447842 2020-03-07 IA State Hygienic Laboratory Pathogen Discovery, Respiratory Viruses Branch, Division of Viral Diseases, Centers for Disease Control and Prevention Krista Queen et al

USA/IA_6390/2020 EPI_ISL_424900 2020-03-07 IA State Hygienic Laboratory Pathogen Discovery, Respiratory Viruses Branch, Division of Viral Diseases, Centers for Disease Control and Prevention Ying Tao et al

USA/IA_6391/2020 EPI_ISL_424899 2020-03-07 IA State Hygienic Laboratory Pathogen Discovery, Respiratory Viruses Branch, Division of Viral Diseases, Centers for Disease Control and Prevention Ying Tao et al

USA/IL_0087/2020 EPI_ISL_424850 2020-03-07 IL Department of Public Health Chicago Laboratory Pathogen Discovery, Respiratory Viruses Branch, Division of Viral Diseases, Centers for Disease Control and Prevention Yan Li et al

USA/IL_0089/2020 EPI_ISL_424851 2020-03-07 IL Department of Public Health Chicago Laboratory Pathogen Discovery, Respiratory Viruses Branch, Division of Viral Diseases, Centers for Disease Control and Prevention Yan Li et al

USA/MA_3642/2020 EPI_ISL_424919 2020-03-07 MA State Public Health Laboratory Pathogen Discovery, Respiratory Viruses Branch, Division of Viral Diseases, Centers for Disease Control and Prevention Ying Tao et al

USA/MA_4811/2020 EPI_ISL_424847 2020-03-07 MA State Public Health Laboratory Pathogen Discovery, Respiratory Viruses Branch, Division of Viral Diseases, Centers for Disease Control and Prevention Yan Li et al

USA/MN2-MDH2/2020 EPI_ISL_414589 2020-03-07 Minnesota Department of Health, Public Health Laboratory Minnesota Department of Health, Public Health Laboratory Matt Plumb et al

USA/MN_0101/2020 EPI_ISL_426427 2020-03-07 MN PHL Division, Minnesota Department of Health Pathogen Discovery, Respiratory Viruses Branch, Division of Viral Diseases, Centers for Disease Control and Prevention Krista Queen et al

USA/NC_0031/2020 EPI_ISL_424873 2020-03-07 NC State Laboratory of Public Health Pathogen Discovery, Respiratory Viruses Branch, Division of Viral Diseases, Centers for Disease Control and Prevention Yan Li et al

USA/NE_7025/2020 EPI_ISL_424875 2020-03-07 NE Public Health Laboratory Pathogen Discovery, Respiratory Viruses Branch, Division of Viral Diseases, Centers for Disease Control and Prevention Yan Li et al

USA/NV_0016/2020 EPI_ISL_426429 2020-03-07 NV State Public Health Laboratory Pathogen Discovery, Respiratory Viruses Branch, Division of Viral Diseases, Centers for Disease Control and Prevention Krista Queen et al

USA/NY-PV09439/2020 EPI_ISL_450116 2020-03-07 MSHS Clinical Microbiology Laboratories MSHS Pathogen Surveillance Program Ana S. Gonzalez-Reiche et al

USA/NY-Wadsworth-11330-01/2020 EPI_ISL_426328 2020-03-07 Wadsworth Center, New York State Department.of Health Wadsworth Center, New York State Department.of Health Kirsten St. George et al

USA/OH_0020/2020 EPI_ISL_426431 2020-03-07 OH Department of Health Laboratory Pathogen Discovery, Respiratory Viruses Branch, Division of Viral Diseases, Centers for Disease Control and Prevention Krista Queen et al

USA/PA_2317/2020 EPI_ISL_424883 2020-03-07 PA Department of Health, Bureau of Laboratories Pathogen Discovery, Respiratory Viruses Branch, Division of Viral Diseases, Centers for Disease Control and Prevention Yan Li et al

USA/PA_2734/2020 EPI_ISL_426432 2020-03-07 PA Department of Health, Bureau of Laboratories Pathogen Discovery, Respiratory Viruses Branch, Division of Viral Diseases, Centers for Disease Control and Prevention Krista Queen et al

USA/PA_2735/2020 EPI_ISL_426433 2020-03-07 PA Department of Health, Bureau of Laboratories Pathogen Discovery, Respiratory Viruses Branch, Division of Viral Diseases, Centers for Disease Control and Prevention Krista Queen et al

USA/SC_3570/2020 EPI_ISL_424903 2020-03-07 SC Dept of Health and Env. Control-Bureau of Laboratories Pathogen Discovery, Respiratory Viruses Branch, Division of Viral Diseases, Centers for Disease Control and Prevention Ying Tao et al

USA/SC_3571/2020 EPI_ISL_424904 2020-03-07 SC Dept of Health and Env. Control-Bureau of Laboratories Pathogen Discovery, Respiratory Viruses Branch, Division of Viral Diseases, Centers for Disease Control and Prevention Ying Tao et al

USA/SC_3575/2020 EPI_ISL_424842 2020-03-07 SC Dept of Health and Env. Control-Bureau of Laboratories Pathogen Discovery, Respiratory Viruses Branch, Division of Viral Diseases, Centers for Disease Control and Prevention Yan Li et al

USA/UT_8906/2020 EPI_ISL_424890 2020-03-07 UT-Unified State Labs: Public Health Utah DOH Pathogen Discovery, Respiratory Viruses Branch, Division of Viral Diseases, Centers for Disease Control and Prevention Yan Li et al

USA/WA-S111/2020 EPI_ISL_417164 2020-03-07 Seattle Flu Study Seattle Flu Study Chu etl al et al

USA/WA-S239/2020 EPI_ISL_430277 2020-03-07 Washington State Department of Health Seattle Flu Study Chu et al et al

USA/WA-S253/2020 EPI_ISL_430291 2020-03-07 Washington State Department of Health Seattle Flu Study Chu et al et al

USA/WA-UW32/2020 EPI_ISL_414619 2020-03-07 UW Virology Lab UW Virology Lab Pavitra Roychoudhury et al

USA/WA-UW36/2020 EPI_ISL_416453 2020-03-07 UW Virology Lab UW Virology Lab Pavitra Roychoudhury et al

USA/WA-UW38/2020 EPI_ISL_416455 2020-03-07 UW Virology Lab UW Virology Lab Pavitra Roychoudhury et al

Vietnam/39607/2020 EPI_ISL_416428 2020-03-07 National Influenza Center, National Institute of Hygiene and Epidemiology (NIHE) National Influenza Center, National Institute of Hygiene and Epidemiology (NIHE) Le Quynh Mai et al

Vietnam/OUCRU022/2020 EPI_ISL_435303 2020-03-07 National Hospital of Tropical Diseases Oxford University Clinical Research Unit, Hanoi, Vietnam Nguyen Thi Tam et al

Vietnam/OUCRU026/2020 EPI_ISL_435305 2020-03-07 National Hospital of Tropical Diseases Oxford University Clinical Research Unit, Hanoi, Vietnam Nguyen Thi Tam et al

Algeria/G0640_2265/2020 EPI_ISL_418242 2020-03-08 NIC Viral Respiratory Unit - Institut Pasteur of Algeria National Reference Center for Viruses of Respiratory Infections, Institut Pasteur, Paris M√©lanie Albert et al

Australia/NSW153/2020 EPI_ISL_427653 2020-03-08 Centre for Infectious Diseases and Microbiology Public Health NSW Health Pathology - Institute of Clinical Pathology and Medical Research; Westmead Hospital; University of Sydney Rockett R et al

Australia/NSW27/2020 EPI_ISL_417390 2020-03-08 Centre for Infectious Diseases and Microbiology Public Health NSW Health Pathology - Institute of Clinical Pathology and Medical Research; Westmead Hospital; University of Sydney Sadsad R et al

Australia/NSW28/2020 EPI_ISL_417391 2020-03-08 Centre for Infectious Diseases and Microbiology Public Health NSW Health Pathology - Institute of Clinical Pathology and Medical Research; Westmead Hospital; University of Sydney Carter I et al

Australia/NSW29/2020 EPI_ISL_427651 2020-03-08 Centre for Infectious Diseases and Microbiology Public Health NSW Health Pathology - Institute of Clinical Pathology and Medical Research; Westmead Hospital; University of Sydney Arnott A et al

Australia/NSW35/2020 EPI_ISL_417393 2020-03-08 Centre for Infectious Diseases and Microbiology Public Health NSW Health Pathology - Institute of Clinical Pathology and Medical Research; Westmead Hospital; University of Sydney Holmes EC et al

Australia/VIC20/2020 EPI_ISL_419741 2020-03-08 Victorian Infectious Diseases Reference Laboratory (VIDRL) Victorian Infectious Diseases Reference Laboratory and Microbiological Diagnostic Unit Public Health Laboratory, Doherty Institute Caly L. et al

Austria/CeMM0176/2020 EPI_ISL_438019 2020-03-08 Center for Virology, Medical University of Vienna Bergthaler laboratory, CeMM Research Center for Molecular Medicine of the Austrian Academy of Sciences Alexandra Popa et al

Canada/ON_PHL3680/2020 EPI_ISL_418348 2020-03-08 Public Health Ontario Laboratories Public Health Ontario Laboratories Alireza Eshaghi et al

Canada/ON_PHL3917/2020 EPI_ISL_418322 2020-03-08 Public Health Ontario Laboratories Public Health Ontario Laboratories Alireza Eshaghi et al

Canada/ON_PHL6883/2020 EPI_ISL_418331 2020-03-08 Public Health Ontario Laboratories Public Health Ontario Laboratories Alireza Eshaghi et al

Canada/ON_PHLU8150/2020 EPI_ISL_418355 2020-03-08 Public Health Ontario Laboratories Public Health Ontario Laboratories Alireza Eshaghi et al

Chile/Santiago_4/2020 EPI_ISL_445247 2020-03-08 UNIVERSIDAD DE LOS ANDES Instituto de Salud Publica de Chile Andr√©s E Castillo et al

Chile/Santiago_op4d1/2020 EPI_ISL_415661 2020-03-08 Laboratory of Molecular Virology, Pontificia Universidad Cat√≥lica de Chile MSHS Pathogen Surveillance Program Rafael A. Medina et al

Denmark/ALAB-SSI-133/2020 EPI_ISL_437642 2020-03-08 Department of Virus and Microbiological Special Diagnostics, Statens Serum Institut, Copenhagen, Denmark, Artillerivej 5, 2300 Copenahgen S Albertsen lab, Department of Chemistry and Bioscience, Aalborg University, Denmark Rasmus Kirkegaard et al

Denmark/ALAB-SSI-135/2020 EPI_ISL_437647 2020-03-08 Department of Virus and Microbiological Special Diagnostics, Statens Serum Institut, Copenhagen, Denmark, Artillerivej 5, 2300 Copenahgen S Albertsen lab, Department of Chemistry and Bioscience, Aalborg University, Denmark Rasmus Kirkegaard et al

Denmark/ALAB-SSI-136/2020 EPI_ISL_437648 2020-03-08 Department of Virus and Microbiological Special Diagnostics, Statens Serum Institut, Copenhagen, Denmark, Artillerivej 5, 2300 Copenahgen S Albertsen lab, Department of Chemistry and Bioscience, Aalborg University, Denmark Rasmus Kirkegaard et al

Denmark/ALAB-SSI-138/2020 EPI_ISL_437650 2020-03-08 Department of Virus and Microbiological Special Diagnostics, Statens Serum Institut, Copenhagen, Denmark, Artillerivej 5, 2300 Copenahgen S Albertsen lab, Department of Chemistry and Bioscience, Aalborg University, Denmark Rasmus Kirkegaard et al

Denmark/ALAB-SSI105/2020 EPI_ISL_429337 2020-03-08 Department of Virus and Microbiological Special Diagnostics, Statens Serum Institut, Copenhagen, Denmark, Artillerivej 5, 2300 Copenahgen S Albertsen lab, Department of Chemistry and Bioscience, Aalborg University, Denmark Rasmus Kirkegaard et al

Denmark/ALAB-SSI110/2020 EPI_ISL_429342 2020-03-08 Department of Virus and Microbiological Special Diagnostics, Statens Serum Institut, Copenhagen, Denmark, Artillerivej 5, 2300 Copenahgen S Albertsen lab, Department of Chemistry and Bioscience, Aalborg University, Denmark Rasmus Kirkegaard et al

Denmark/ALAB-SSI113/2020 EPI_ISL_429344 2020-03-08 Department of Virus and Microbiological Special Diagnostics, Statens Serum Institut, Copenhagen, Denmark, Artillerivej 5, 2300 Copenahgen S Albertsen lab, Department of Chemistry and Bioscience, Aalborg University, Denmark Rasmus Kirkegaard et al

Denmark/ALAB-SSI119/2020 EPI_ISL_429348 2020-03-08 Department of Virus and Microbiological Special Diagnostics, Statens Serum Institut, Copenhagen, Denmark, Artillerivej 5, 2300 Copenahgen S Albertsen lab, Department of Chemistry and Bioscience, Aalborg University, Denmark Rasmus Kirkegaard et al

Denmark/ALAB-SSI120/2020 EPI_ISL_429349 2020-03-08 Department of Virus and Microbiological Special Diagnostics, Statens Serum Institut, Copenhagen, Denmark, Artillerivej 5, 2300 Copenahgen S Albertsen lab, Department of Chemistry and Bioscience, Aalborg University, Denmark Rasmus Kirkegaard et al

Denmark/ALAB-SSI121/2020 EPI_ISL_429350 2020-03-08 Department of Virus and Microbiological Special Diagnostics, Statens Serum Institut, Copenhagen, Denmark, Artillerivej 5, 2300 Copenahgen S Albertsen lab, Department of Chemistry and Bioscience, Aalborg University, Denmark Rasmus Kirkegaard et al

Denmark/ALAB-SSI122/2020 EPI_ISL_429351 2020-03-08 Department of Virus and Microbiological Special Diagnostics, Statens Serum Institut, Copenhagen, Denmark, Artillerivej 5, 2300 Copenahgen S Albertsen lab, Department of Chemistry and Bioscience, Aalborg University, Denmark Rasmus Kirkegaard et al

Denmark/ALAB-SSI123/2020 EPI_ISL_429352 2020-03-08 Department of Virus and Microbiological Special Diagnostics, Statens Serum Institut, Copenhagen, Denmark, Artillerivej 5, 2300 Copenahgen S Albertsen lab, Department of Chemistry and Bioscience, Aalborg University, Denmark Rasmus Kirkegaard et al

Denmark/ALAB-SSI125/2020 EPI_ISL_429353 2020-03-08 Department of Virus and Microbiological Special Diagnostics, Statens Serum Institut, Copenhagen, Denmark, Artillerivej 5, 2300 Copenahgen S Albertsen lab, Department of Chemistry and Bioscience, Aalborg University, Denmark Rasmus Kirkegaard et al

Denmark/ALAB-SSI126/2020 EPI_ISL_429354 2020-03-08 Department of Virus and Microbiological Special Diagnostics, Statens Serum Institut, Copenhagen, Denmark, Artillerivej 5, 2300 Copenahgen S Albertsen lab, Department of Chemistry and Bioscience, Aalborg University, Denmark Rasmus Kirkegaard et al

Denmark/ALAB-SSI128/2020 EPI_ISL_429355 2020-03-08 Department of Virus and Microbiological Special Diagnostics, Statens Serum Institut, Copenhagen, Denmark, Artillerivej 5, 2300 Copenahgen S Albertsen lab, Department of Chemistry and Bioscience, Aalborg University, Denmark Rasmus Kirkegaard et al

Denmark/ALAB-SSI129/2020 EPI_ISL_429356 2020-03-08 Department of Virus and Microbiological Special Diagnostics, Statens Serum Institut, Copenhagen, Denmark, Artillerivej 5, 2300 Copenahgen S Albertsen lab, Department of Chemistry and Bioscience, Aalborg University, Denmark Rasmus Kirkegaard et al

Denmark/ALAB-SSI132/2020 EPI_ISL_429357 2020-03-08 Department of Virus and Microbiological Special Diagnostics, Statens Serum Institut, Copenhagen, Denmark, Artillerivej 5, 2300 Copenahgen S Albertsen lab, Department of Chemistry and Bioscience, Aalborg University, Denmark Rasmus Kirkegaard et al

Denmark/ALAB-SSI404/2020 EPI_ISL_429497 2020-03-08 Department of Virus and Microbiological Special Diagnostics, Statens Serum Institut, Copenhagen, Denmark, Artillerivej 5, 2300 Copenahgen S Albertsen lab, Department of Chemistry and Bioscience, Aalborg University, Denmark Rasmus Kirkegaard et al

Denmark/ALAB-SSI405/2020 EPI_ISL_429499 2020-03-08 Department of Virus and Microbiological Special Diagnostics, Statens Serum Institut, Copenhagen, Denmark, Artillerivej 5, 2300 Copenahgen S Albertsen lab, Department of Chemistry and Bioscience, Aalborg University, Denmark Rasmus Kirkegaard et al

England/20109050406/2020 EPI_ISL_417265 2020-03-08 Respiratory Virus Unit, Microbiology Services Colindale, Public Health England Respiratory Virus Unit, Microbiology Services Colindale, Public Health England Monica Galiano et al

England/20109094506/2020 EPI_ISL_417292 2020-03-08 Respiratory Virus Unit, Microbiology Services Colindale, Public Health England Respiratory Virus Unit, Microbiology Services Colindale, Public Health England Monica Galiano et al

England/20109097506/2020 EPI_ISL_417293 2020-03-08 Respiratory Virus Unit, Microbiology Services Colindale, Public Health England Respiratory Virus Unit, Microbiology Services Colindale, Public Health England Monica Galiano et al

England/20109098806/2020 EPI_ISL_417295 2020-03-08 Respiratory Virus Unit, Microbiology Services Colindale, Public Health England Respiratory Virus Unit, Microbiology Services Colindale, Public Health England Monica Galiano et al

England/20109099206/2020 EPI_ISL_417299 2020-03-08 Respiratory Virus Unit, Microbiology Services Colindale, Public Health England Respiratory Virus Unit, Microbiology Services Colindale, Public Health England Monica Galiano et al

England/20110023706/2020 EPI_ISL_417307 2020-03-08 Respiratory Virus Unit, Microbiology Services Colindale, Public Health England Respiratory Virus Unit, Microbiology Services Colindale, Public Health England Monica Galiano et al

England/20110059306/2020 EPI_ISL_417312 2020-03-08 Respiratory Virus Unit, Microbiology Services Colindale, Public Health England Respiratory Virus Unit, Microbiology Services Colindale, Public Health England Monica Galiano et al

England/20110097506/2020 EPI_ISL_417313 2020-03-08 Respiratory Virus Unit, Microbiology Services Colindale, Public Health England Respiratory Virus Unit, Microbiology Services Colindale, Public Health England Monica Galiano et al

Finland/FIN-455/2020 EPI_ISL_414642 2020-03-08 Department of Virology and Immunology, University of Helsinki and Helsinki University Hospital, Huslab Finland Department of Virology, Faculty of Medicine, University of Helsinki, Helsinki, Finland Teemu Smura et al

France/HF2150/2020 EPI_ISL_418224 2020-03-08 Centre Hospitalier Compi√®gne Laboratoire de Biologie National Reference Center for Viruses of Respiratory Infections, Institut Pasteur, Paris M√©lanie Albert et al

France/HF2155/2020 EPI_ISL_418225 2020-03-08 Centre Hospitalier Compi√®gne Laboratoire de Biologie National Reference Center for Viruses of Respiratory Infections, Institut Pasteur, Paris M√©lanie Albert et al

France/HF2196/2020 EPI_ISL_416493 2020-03-08 CH Jean de Navarre Laboratoire de Biologie National Reference Center for Viruses of Respiratory Infections, Institut Pasteur, Paris M√©lnie Albert et al

France/Lyon_06820/2020 EPI_ISL_417339 2020-03-08 Institut des Agents Infectieux (IAI), Hospices Civils de Lyon CNR Virus des Infections Respiratoires - France SUD Antonin Bal et al

France/Lyon_0693/2020 EPI_ISL_416758 2020-03-08 Institut des Agents Infectieux (IAI) Hospices Civils de Lyon CNR Virus des Infections Respiratoires - France SUD Bal et al

Georgia/Tb-390/2020 EPI_ISL_416477 2020-03-08 R. G. Lugar Center for Public Health Research, National Center for Disease Control and Public Health (NCDC) of Georgia. R. G. Lugar Center for Public Health Research, National Center for Disease Control and Public Health (NCDC) of Georgia. Marine Murtskhvaladze et al

Greece/237_31890/2020 EPI_ISL_437896 2020-03-08 Laboratory of Microbiology, Medical School, National and Kapodistrian University of Athens Laboratory of Biology, Department of Medicine, Democritus University of Thrace Kassela K. et al

Iceland/69/2020 EPI_ISL_417749 2020-03-08 The National University Hospital of Iceland deCODE genetics Daniel F Gudbjartsson et al

Iceland/70/2020 EPI_ISL_417728 2020-03-08 The National University Hospital of Iceland deCODE genetics Daniel F Gudbjartsson et al

Ireland/Dublin-22361/2020 EPI_ISL_418580 2020-03-08 UCD National Virus Reference Laboratory UCD National Virus Reference Laboratory Michael Carr et al

Ireland/Dublin-22428/2020 EPI_ISL_418581 2020-03-08 UCD National Virus Reference Laboratory UCD National Virus Reference Laboratory Michael Carr et al

Japan/P1/2020 EPI_ISL_419296 2020-03-08 Kochi Prefectural Institute of Public Health Pathogen Genomics Center, National Institute of Infectious Diseases Tsuyoshi Sekizuka et al

Luxembourg/LNS2614631/2020 EPI_ISL_419578 2020-03-08 Laboratoire National de Sant√©, Microbiology, Virology Laboratoire National de Sant√©, Microbiology, Epidemiology and Microbial Genomics Anke Wienecke-Baldacchino et al

Netherlands/NA_21/2020 EPI_ISL_415478 2020-03-08 Dutch COVID-19 response team Erasmus Medical Center David Nieuwenhuijse et al

Netherlands/NA_22/2020 EPI_ISL_415479 2020-03-08 Dutch COVID-19 response team Erasmus Medical Center David Nieuwenhuijse et al

Netherlands/NA_24/2020 EPI_ISL_415481 2020-03-08 Dutch COVID-19 response team Erasmus Medical Center David Nieuwenhuijse et al

Netherlands/NA_44/2020 EPI_ISL_422805 2020-03-08 Dutch COVID-19 response team Erasmus Medical Center Bas Oude Munnink et al

Netherlands/NA_46/2020 EPI_ISL_422807 2020-03-08 Dutch COVID-19 response team Erasmus Medical Center Bas Oude Munnink et al

Netherlands/NA_47/2020 EPI_ISL_422808 2020-03-08 Dutch COVID-19 response team Erasmus Medical Center Bas Oude Munnink et al

Netherlands/NA_50/2020 EPI_ISL_422811 2020-03-08 Dutch COVID-19 response team Erasmus Medical Center Bas Oude Munnink et al

Netherlands/NA_57/2020 EPI_ISL_422818 2020-03-08 Dutch COVID-19 response team Erasmus Medical Center Bas Oude Munnink et al

Netherlands/NA_58/2020 EPI_ISL_422819 2020-03-08 Dutch COVID-19 response team Erasmus Medical Center Bas Oude Munnink et al

Netherlands/NoordBrabant_105/2020 EPI_ISL_422864 2020-03-08 Dutch COVID-19 response team Erasmus Medical Center Bas Oude Munnink et al

Netherlands/NoordBrabant_27/2020 EPI_ISL_414536 2020-03-08 Dutch COVID-19 response team Erasmus Medical Center David Nieuwenhuijse et al

Netherlands/NoordBrabant_28/2020 EPI_ISL_414537 2020-03-08 Dutch COVID-19 response team Erasmus Medical Center David Nieuwenhuijse et al

Netherlands/NoordBrabant_30/2020 EPI_ISL_414539 2020-03-08 Dutch COVID-19 response team Erasmus Medical Center David Nieuwenhuijse et al

Netherlands/NoordBrabant_32/2020 EPI_ISL_414541 2020-03-08 Dutch COVID-19 response team Erasmus Medical Center David Nieuwenhuijse et al

Netherlands/Utrecht_15/2020 EPI_ISL_414554 2020-03-08 Dutch COVID-19 response team Erasmus Medical Center David Nieuwenhuijse et al

Netherlands/Utrecht_16/2020 EPI_ISL_414555 2020-03-08 Dutch COVID-19 response team Erasmus Medical Center David Nieuwenhuijse et al

Netherlands/ZuidHolland_15/2020 EPI_ISL_414557 2020-03-08 Dutch COVID-19 response team Erasmus Medical Center David Nieuwenhuijse et al

Netherlands/ZuidHolland_22/2020 EPI_ISL_414564 2020-03-08 Dutch COVID-19 response team Erasmus Medical Center David Nieuwenhuijse et al

Norway/1772/2020 EPI_ISL_420137 2020-03-08 Vestfold Hospital, Tonsberg Department of Microbiology Norwegian Institute of Public Health, Department of Virology Kathrine Stene-Johansen et al

Portugal/PT0006b/2020 EPI_ISL_417991 2020-03-08 CHULC - H Curry Cabral Instituto Nacional de Saude (INSA) Guiomar et al et al

Portugal/PT0008/2020 EPI_ISL_417993 2020-03-08 CHULC - H D Estefania Instituto Nacional de Saude (INSA) Guiomar et al et al

Portugal/PT0009/2020 EPI_ISL_417994 2020-03-08 CHULC - H Curry Cabral Instituto Nacional de Saude (INSA) Guiomar et al et al

Portugal/PT0010/2020 EPI_ISL_417995 2020-03-08 CHULC - H Curry Cabral Instituto Nacional de Saude (INSA) Guiomar et al et al

Portugal/PT0011/2020 EPI_ISL_417996 2020-03-08 CHULC - H Curry Cabral Instituto Nacional de Saude (INSA) Guiomar et al et al

Portugal/PT0013/2020 EPI_ISL_417998 2020-03-08 Centro Hospital do Porto, E.P.E. - H. Geral de Santo Antonio Instituto Nacional de Saude (INSA) Guiomar et al et al

Scotland/EDB003/2020 EPI_ISL_415640 2020-03-08 Virology Department, Royal Infirmary of Edinburgh, NHS Lothian Virology Department, Royal Infirmary of Edinburgh, NHS Lothian McHugh M et al

Singapore/49/2020 EPI_ISL_428828 2020-03-08 National Public Health Laboratory, National Centre for Infectious Diseases National Public Health Laboratory, National Centre for Infectious Diseases Mak TM et al

Singapore/50/2020 EPI_ISL_428829 2020-03-08 National Public Health Laboratory, National Centre for Infectious Diseases National Public Health Laboratory, National Centre for Infectious Diseases Mak TM et al

Slovakia/SK-BMC6/2020 EPI_ISL_417880 2020-03-08 Institute of Virology, Biomedical Research Center of the Slovak Academy of Sciences, Bratislava; Public Health Authority of the Slovak Republic, Bratislava Institute of Virology, Biomedical Research Center of the Slovak Academy of Sciences, Bratislava; Comenius University Science Park, Bratislava Monika Sl√°vikova et al

Spain/Madrid_H12_1703/2020 EPI_ISL_421173 2020-03-08 Hospital Universitario 12 de Octubre Hospital Universitario 12 de Octubre Esther Viedma et al

Spain/Madrid_LP22_5885/2020 EPI_ISL_428678 2020-03-08 Hospital Universitario La Paz Hospital Universitario 12 de Octubre Elias Dahdouh et al

Spain/Madrid_LP23_5852/2020 EPI_ISL_428679 2020-03-08 Hospital Universitario La Paz Hospital Universitario 12 de Octubre Elias Dahdouh et al

Spain/Valencia19/2020 EPI_ISL_419683 2020-03-08 Servicio de Microbiolog√≠a. Consorcio Hospital General Universitario de Valencia Sequencing and Bioinformatics Service and Molecular Epidemiology Research Group. FISABIO-Public Health Griselda De Marco et al

Sweden/20-50170/2020 EPI_ISL_429145 2020-03-08 Klinisk mikrobiologi Orebro The Public Health Agency of Sweden Martin Sundqvist et al

Sweden/20-50171/2020 EPI_ISL_429146 2020-03-08 Klinisk mikrobiologi Orebro The Public Health Agency of Sweden Martin Sundqvist et al

Sweden/20-50172/2020 EPI_ISL_429147 2020-03-08 Klinisk mikrobiologi Orebro The Public Health Agency of Sweden Martin Sundqvist et al

Sweden/20-50173/2020 EPI_ISL_429148 2020-03-08 Klinisk mikrobiologi Orebro The Public Health Agency of Sweden Martin Sundqvist et al

Sweden/20-50175/2020 EPI_ISL_429150 2020-03-08 Klinisk mikrobiologi Orebro The Public Health Agency of Sweden Martin Sundqvist et al

Sweden/20-50176/2020 EPI_ISL_429151 2020-03-08 Klinisk mikrobiologi Orebro The Public Health Agency of Sweden Martin Sundqvist et al

Sweden/20-50179/2020 EPI_ISL_429152 2020-03-08 Klinisk mikrobiologi Orebro The Public Health Agency of Sweden Martin Sundqvist et al

Sweden/20-50180/2020 EPI_ISL_429153 2020-03-08 Klinisk mikrobiologi Orebro The Public Health Agency of Sweden Martin Sundqvist et al

Sweden/20-50181/2020 EPI_ISL_429154 2020-03-08 Klinisk mikrobiologi Orebro The Public Health Agency of Sweden Martin Sundqvist et al

Sweden/20-50185/2020 EPI_ISL_430862 2020-03-08 The Public Health Agency of Sweden The Public Health Agency of Sweden Oskar Karlsson Lindsjo et al

Switzerland/GE6679/2020 EPI_ISL_415707 2020-03-08 H√¥pitaux universitaires de Gen√®ve Laboratoire de Virologie H√¥pitaux universitaires de Gen√®ve Laboratoire de Virologie Laubscher F. et al

USA/DC-CDC-0019/2020 EPI_ISL_447840 2020-03-08 DC Public Health Lab/ Dept. of Forensic Sciences Pathogen Discovery, Respiratory Viruses Branch, Division of Viral Diseases, Centers for Disease Control and Prevention Krista Queen et al

USA/GA_2118/2020 EPI_ISL_424861 2020-03-08 GA Department of Public Health Laboratory Pathogen Discovery, Respiratory Viruses Branch, Division of Viral Diseases, Centers for Disease Control and Prevention Yan Li et al

USA/IA_6394/2020 EPI_ISL_424865 2020-03-08 IA State Hygienic Laboratory Pathogen Discovery, Respiratory Viruses Branch, Division of Viral Diseases, Centers for Disease Control and Prevention Yan Li et al

USA/IA_6395/2020 EPI_ISL_424896 2020-03-08 IA State Hygienic Laboratory Pathogen Discovery, Respiratory Viruses Branch, Division of Viral Diseases, Centers for Disease Control and Prevention Ying Tao et al

USA/IA_6396/2020 EPI_ISL_424895 2020-03-08 IA State Hygienic Laboratory Pathogen Discovery, Respiratory Viruses Branch, Division of Viral Diseases, Centers for Disease Control and Prevention Ying Tao et al

USA/IA_6399/2020 EPI_ISL_424898 2020-03-08 IA State Hygienic Laboratory Pathogen Discovery, Respiratory Viruses Branch, Division of Viral Diseases, Centers for Disease Control and Prevention Ying Tao et al

USA/IA_6401/2020 EPI_ISL_424897 2020-03-08 IA State Hygienic Laboratory Pathogen Discovery, Respiratory Viruses Branch, Division of Viral Diseases, Centers for Disease Control and Prevention Ying Tao et al

USA/NY-SURV0319/2020 EPI_ISL_436061 2020-03-08 NYC Department of Health and Mental Hygiene Pathogen Discovery, Respiratory Viruses Branch, Division of Viral Diseases, Centers for Disease Control and Prevention Ying Tao et al

USA/NY-SURV0592/2020 EPI_ISL_436072 2020-03-08 NYC Department of Health and Mental Hygiene Pathogen Discovery, Respiratory Viruses Branch, Division of Viral Diseases, Centers for Disease Control and Prevention Ying Tao et al

USA/NY-Wadsworth-11666-01/2020 EPI_ISL_426050 2020-03-08 Wadsworth Center, New York State Department.of Health Wadsworth Center, New York State Department.of Health Kirsten St. George et al

USA/NY-Wadsworth-11717-01/2020 EPI_ISL_426291 2020-03-08 Wadsworth Center, New York State Department.of Health Wadsworth Center, New York State Department.of Health Kirsten St. George et al

USA/NY-Wadsworth-11735-01/2020 EPI_ISL_426290 2020-03-08 Wadsworth Center, New York State Department of Health Wadsworth Center, New York State Department.of Health Kirsten St. George et al

USA/NY-Wadsworth-11736-02/2020 EPI_ISL_426311 2020-03-08 Wadsworth Center, New York State Department.of Health Wadsworth Center, New York State Department.of Health Kirsten St. George et al

USA/OH_0019/2020 EPI_ISL_426430 2020-03-08 OH Department of Health Laboratory Pathogen Discovery, Respiratory Viruses Branch, Division of Viral Diseases, Centers for Disease Control and Prevention Krista Queen et al

USA/OH_0023/2020 EPI_ISL_424880 2020-03-08 OH Department of Health Laboratory Pathogen Discovery, Respiratory Viruses Branch, Division of Viral Diseases, Centers for Disease Control and Prevention Yan Li et al

USA/PA-CDC-2908/2020 EPI_ISL_447844 2020-03-08 PA Department of Health, Bureau of Laboratories Pathogen Discovery, Respiratory Viruses Branch, Division of Viral Diseases, Centers for Disease Control and Prevention Krista Queen et al

USA/PR-CDC-3578/2020 EPI_ISL_447845 2020-03-08 PR - Biological and Chemical Emergencies Lab Office of Public Health Preparedness and Response Pathogen Discovery, Respiratory Viruses Branch, Division of Viral Diseases, Centers for Disease Control and Prevention Krista Queen et al

USA/SC_6370/2020 EPI_ISL_424888 2020-03-08 SC Dept of Health and Env. Control-Bureau of Laboratories Pathogen Discovery, Respiratory Viruses Branch, Division of Viral Diseases, Centers for Disease Control and Prevention Yan Li et al

USA/VA-DCLS-0008/2020 EPI_ISL_419260 2020-03-08 Virginia DCLS Virginia DCLS Virginia DCLS et al

USA/VA_6352/2020 EPI_ISL_424891 2020-03-08 VA-Division of Consolidated Laboratory Services Pathogen Discovery, Respiratory Viruses Branch, Division of Viral Diseases, Centers for Disease Control and Prevention Yan Li et al

USA/VA_6377/2020 EPI_ISL_424892 2020-03-08 VA-Division of Consolidated Laboratory Services Pathogen Discovery, Respiratory Viruses Branch, Division of Viral Diseases, Centers for Disease Control and Prevention Yan Li et al

USA/VA_6382/2020 EPI_ISL_424893 2020-03-08 VA-Division of Consolidated Laboratory Services Pathogen Discovery, Respiratory Viruses Branch, Division of Viral Diseases, Centers for Disease Control and Prevention Yan Li et al

USA/VA_6389/2020 EPI_ISL_424894 2020-03-08 VA-Division of Consolidated Laboratory Services Pathogen Discovery, Respiratory Viruses Branch, Division of Viral Diseases, Centers for Disease Control and Prevention Yan Li et al

USA/WA-S112/2020 EPI_ISL_417165 2020-03-08 Seattle Flu Study Seattle Flu Study Chu etl al et al

USA/WA-S232/2020 EPI_ISL_430270 2020-03-08 Washington State Department of Health Seattle Flu Study Chu et al et al

USA/WA-S255/2020 EPI_ISL_430293 2020-03-08 Washington State Department of Health Seattle Flu Study Chu et al et al

USA/WA-S274/2020 EPI_ISL_430128 2020-03-08 Seattle Flu Study Seattle Flu Study Chu et al et al

USA/WA-S367/2020 EPI_ISL_434127 2020-03-08 Washington State Department of Health Seattle Flu Study Chu et al et al

USA/WA-UW30/2020 EPI_ISL_414617 2020-03-08 UW Virology Lab UW Virology Lab Pavitra Roychoudhury et al

USA/WA-UW31/2020 EPI_ISL_414618 2020-03-08 UW Virology Lab UW Virology Lab Pavitra Roychoudhury et al

USA/WA-UW33/2020 EPI_ISL_414620 2020-03-08 UW Virology Lab UW Virology Lab Pavitra Roychoudhury et al

USA/WA-UW41/2020 EPI_ISL_415606 2020-03-08 UW Virology Lab UW Virology Lab Pavitra Roychoudhury et al

USA/WA-UW43/2020 EPI_ISL_415608 2020-03-08 UW Virology Lab UW Virology Lab Pavitra Roychoudhury et al

USA/WA-UW44/2020 EPI_ISL_415609 2020-03-08 UW Virology Lab UW Virology Lab Pavitra Roychoudhury et al

USA/WA-UW50/2020 EPI_ISL_415615 2020-03-08 UW Virology Lab UW Virology Lab Pavitra Roychoudhury et al

USA/WA-UW51/2020 EPI_ISL_415616 2020-03-08 UW Virology Lab UW Virology Lab Pavitra Roychoudhury et al

Australia/NSW154/2020 EPI_ISL_427652 2020-03-09 Centre for Infectious Diseases and Microbiology Public Health NSW Health Pathology - Institute of Clinical Pathology and Medical Research; Westmead Hospital; University of Sydney Lam C et al

Australia/NSW26/2020 EPI_ISL_417389 2020-03-09 Centre for Infectious Diseases and Microbiology Public Health NSW Health Pathology - Institute of Clinical Pathology and Medical Research; Westmead Hospital; University of Sydney Arnott A et al

Australia/VIC21/2020 EPI_ISL_419740 2020-03-09 Victorian Infectious Diseases Reference Laboratory (VIDRL) Victorian Infectious Diseases Reference Laboratory and Microbiological Diagnostic Unit Public Health Laboratory, Doherty Institute Caly L. et al

Australia/VIC22/2020 EPI_ISL_419742 2020-03-09 Victorian Infectious Diseases Reference Laboratory (VIDRL) Victorian Infectious Diseases Reference Laboratory and Microbiological Diagnostic Unit Public Health Laboratory, Doherty Institute Caly L. et al

Australia/VIC23/2020 EPI_ISL_419743 2020-03-09 Victorian Infectious Diseases Reference Laboratory (VIDRL) Victorian Infectious Diseases Reference Laboratory and Microbiological Diagnostic Unit Public Health Laboratory, Doherty Institute Caly L. et al

Austria/CeMM0023/2020 EPI_ISL_437913 2020-03-09 Institut f√ºr Virologie am Department f√ºr Hygiene, Mikrobiologie und Public Health Bergthaler laboratory, CeMM Research Center for Molecular Medicine of the Austrian Academy of Sciences Alexandra Popa et al

Belgium/030959/2020 EPI_ISL_420320 2020-03-09 KU Leuven, Clinical and Epidemiological Virology KU Leuven, Clinical and Epidemiological Virology Joan Marti-Carreras et al

Belgium/AC-030982/2020 EPI_ISL_420338 2020-03-09 KU Leuven, Clinical and Epidemiological Virology KU Leuven, Clinical and Epidemiological Virology Joan Marti-Carreras et al

Belgium/BG-030955/2020 EPI_ISL_418984 2020-03-09 KU Leuven, Clinical and Epidemiological Virology KU Leuven, Clinical and Epidemiological Virology Bert Vanmechelen et al

Belgium/DWAEE-030954/2020 EPI_ISL_418983 2020-03-09 KU Leuven, Clinical and Epidemiological Virology KU Leuven, Clinical and Epidemiological Virology Bert Vanmechelen et al

Belgium/FAE-030948/2020 EPI_ISL_420445 2020-03-09 KU Leuven, Clinical and Epidemiological Virology KU Leuven, Clinical and Epidemiological Virology Joan Marti-Carreras et al

Belgium/HAA-030953/2020 EPI_ISL_418982 2020-03-09 KU Leuven, Clinical and Epidemiological Virology KU Leuven, Clinical and Epidemiological Virology Bert Vanmechelen et al

Belgium/LT-030956/2020 EPI_ISL_418985 2020-03-09 KU Leuven, Clinical and Epidemiological Virology KU Leuven, Clinical and Epidemiological Virology Bert Vanmechelen et al

Belgium/RDC-030961/2020 EPI_ISL_420452 2020-03-09 KU Leuven, Clinical and Epidemiological Virology KU Leuven, Clinical and Epidemiological Virology Joan Marti-Carreras et al

Belgium/SB-030990/2020 EPI_ISL_420346 2020-03-09 KU Leuven, Clinical and Epidemiological Virology KU Leuven, Clinical and Epidemiological Virology Joan Marti-Carreras et al

Brazil/CV4/2020 EPI_ISL_429667 2020-03-09 Central Public Health Laboratory/Oct√°vio Magalh√£es Institute (IOM) from the Ezequiel Dias Foundation (FUNED) Instituto Oct√°vio Magalh√£es / Funda√ß√£o Ezequiel Dias (IOM/Funed) Talita Adelino et al

Canada/BC_3808524/2020 EPI_ISL_418819 2020-03-09 BCCDC Public Health Laboratory BCCDC Public Health Laboratory Harrigan et al

Canada/BC_3842755/2020 EPI_ISL_418820 2020-03-09 BCCDC Public Health Laboratory BCCDC Public Health Laboratory Harrigan et al

Canada/BC_3989992/2020 EPI_ISL_418823 2020-03-09 BCCDC Public Health Laboratory BCCDC Public Health Laboratory Harrigan et al

Canada/BC_4799711/2020 EPI_ISL_418830 2020-03-09 BCCDC Public Health Laboratory BCCDC Public Health Laboratory Harrigan et al

Canada/NB_1/2020 EPI_ISL_429806 2020-03-09 Dr. Georges-L.-Dumont University Hospital Centre National Microbiology Laboratory Anna Majer et al

Canada/ON_PHL0539/2020 EPI_ISL_418353 2020-03-09 Public Health Ontario Laboratories Public Health Ontario Laboratories Alireza Eshaghi et al

Canada/ON_PHL1083/2020 EPI_ISL_418340 2020-03-09 Public Health Ontario Laboratories Public Health Ontario Laboratories Alireza Eshaghi et al

Canada/ON_PHL3877/2020 EPI_ISL_418339 2020-03-09 Public Health Ontario Laboratories Public Health Ontario Laboratories Alireza Eshaghi et al

Canada/SK_4/2020 EPI_ISL_418810 2020-03-09 Roy Romanow Provincial Laboratory National Microbiology Laboratory Anna Majer et al

Chile/Santiago_6/2020 EPI_ISL_445249 2020-03-09 CLINICA SANTA MARIA S.A. Instituto de Salud Publica de Chile Andr√©s E Castillo et al

Chile/Santiago_7/2020 EPI_ISL_445250 2020-03-09 CLINICA ALEMANA DE SANTIAGO S.A. Instituto de Salud Publica de Chile Andr√©s E Castillo et al

Chile/Santiago_9/2020 EPI_ISL_445252 2020-03-09 PONTIFICIA U. CATOLICA FAC. MEDICINA Instituto de Salud Publica de Chile Andr√©s E Castillo et al

Denmark/ALAB-SSI-1261/2020 EPI_ISL_444833 2020-03-09 Department of Virus and Microbiological Special Diagnostics, Statens Serum Institut, Copenhagen, Denmark, Artillerivej 5, 2300 Copenahgen S Albertsen lab, Department of Chemistry and Bioscience, Aalborg University, Denmark Rasmus Kirkegaard et al

Denmark/ALAB-SSI-1262/2020 EPI_ISL_444834 2020-03-09 Department of Virus and Microbiological Special Diagnostics, Statens Serum Institut, Copenhagen, Denmark, Artillerivej 5, 2300 Copenahgen S Albertsen lab, Department of Chemistry and Bioscience, Aalborg University, Denmark Rasmus Kirkegaard et al

Denmark/ALAB-SSI-1263/2020 EPI_ISL_444835 2020-03-09 Department of Virus and Microbiological Special Diagnostics, Statens Serum Institut, Copenhagen, Denmark, Artillerivej 5, 2300 Copenahgen S Albertsen lab, Department of Chemistry and Bioscience, Aalborg University, Denmark Rasmus Kirkegaard et al

Denmark/ALAB-SSI-1264/2020 EPI_ISL_444836 2020-03-09 Department of Virus and Microbiological Special Diagnostics, Statens Serum Institut, Copenhagen, Denmark, Artillerivej 5, 2300 Copenahgen S Albertsen lab, Department of Chemistry and Bioscience, Aalborg University, Denmark Rasmus Kirkegaard et al

Denmark/ALAB-SSI-1265/2020 EPI_ISL_444837 2020-03-09 Department of Virus and Microbiological Special Diagnostics, Statens Serum Institut, Copenhagen, Denmark, Artillerivej 5, 2300 Copenahgen S Albertsen lab, Department of Chemistry and Bioscience, Aalborg University, Denmark Rasmus Kirkegaard et al

Denmark/ALAB-SSI-1268/2020 EPI_ISL_444838 2020-03-09 Department of Virus and Microbiological Special Diagnostics, Statens Serum Institut, Copenhagen, Denmark, Artillerivej 5, 2300 Copenahgen S Albertsen lab, Department of Chemistry and Bioscience, Aalborg University, Denmark Rasmus Kirkegaard et al

Denmark/ALAB-SSI-1269/2020 EPI_ISL_444839 2020-03-09 Department of Virus and Microbiological Special Diagnostics, Statens Serum Institut, Copenhagen, Denmark, Artillerivej 5, 2300 Copenahgen S Albertsen lab, Department of Chemistry and Bioscience, Aalborg University, Denmark Rasmus Kirkegaard et al

Denmark/ALAB-SSI-1270/2020 EPI_ISL_444840 2020-03-09 Department of Virus and Microbiological Special Diagnostics, Statens Serum Institut, Copenhagen, Denmark, Artillerivej 5, 2300 Copenahgen S Albertsen lab, Department of Chemistry and Bioscience, Aalborg University, Denmark Rasmus Kirkegaard et al

Denmark/ALAB-SSI-197/2020 EPI_ISL_436963 2020-03-09 Department of Virus and Microbiological Special Diagnostics, Statens Serum Institut, Copenhagen, Denmark, Artillerivej 5, 2300 Copenahgen S Albertsen lab, Department of Chemistry and Bioscience, Aalborg University, Denmark Rasmus Kirkegaard et al

Denmark/ALAB-SSI-762/2020 EPI_ISL_444893 2020-03-09 Department of Virus and Microbiological Special Diagnostics, Statens Serum Institut, Copenhagen, Denmark, Artillerivej 5, 2300 Copenahgen S Albertsen lab, Department of Chemistry and Bioscience, Aalborg University, Denmark Rasmus Kirkegaard et al

Denmark/ALAB-SSI111/2020 EPI_ISL_429343 2020-03-09 Department of Virus and Microbiological Special Diagnostics, Statens Serum Institut, Copenhagen, Denmark, Artillerivej 5, 2300 Copenahgen S Albertsen lab, Department of Chemistry and Bioscience, Aalborg University, Denmark Rasmus Kirkegaard et al

Denmark/ALAB-SSI114/2020 EPI_ISL_429345 2020-03-09 Department of Virus and Microbiological Special Diagnostics, Statens Serum Institut, Copenhagen, Denmark, Artillerivej 5, 2300 Copenahgen S Albertsen lab, Department of Chemistry and Bioscience, Aalborg University, Denmark Rasmus Kirkegaard et al

Denmark/ALAB-SSI142/2020 EPI_ISL_429360 2020-03-09 Department of Virus and Microbiological Special Diagnostics, Statens Serum Institut, Copenhagen, Denmark, Artillerivej 5, 2300 Copenahgen S Albertsen lab, Department of Chemistry and Bioscience, Aalborg University, Denmark Rasmus Kirkegaard et al

Denmark/ALAB-SSI143/2020 EPI_ISL_429361 2020-03-09 Department of Virus and Microbiological Special Diagnostics, Statens Serum Institut, Copenhagen, Denmark, Artillerivej 5, 2300 Copenahgen S Albertsen lab, Department of Chemistry and Bioscience, Aalborg University, Denmark Rasmus Kirkegaard et al

Denmark/ALAB-SSI146/2020 EPI_ISL_429364 2020-03-09 Department of Virus and Microbiological Special Diagnostics, Statens Serum Institut, Copenhagen, Denmark, Artillerivej 5, 2300 Copenahgen S Albertsen lab, Department of Chemistry and Bioscience, Aalborg University, Denmark Rasmus Kirkegaard et al

Denmark/ALAB-SSI150/2020 EPI_ISL_429368 2020-03-09 Department of Virus and Microbiological Special Diagnostics, Statens Serum Institut, Copenhagen, Denmark, Artillerivej 5, 2300 Copenahgen S Albertsen lab, Department of Chemistry and Bioscience, Aalborg University, Denmark Rasmus Kirkegaard et al

Denmark/ALAB-SSI151/2020 EPI_ISL_429369 2020-03-09 Department of Virus and Microbiological Special Diagnostics, Statens Serum Institut, Copenhagen, Denmark, Artillerivej 5, 2300 Copenahgen S Albertsen lab, Department of Chemistry and Bioscience, Aalborg University, Denmark Rasmus Kirkegaard et al

Denmark/ALAB-SSI154/2020 EPI_ISL_429370 2020-03-09 Department of Virus and Microbiological Special Diagnostics, Statens Serum Institut, Copenhagen, Denmark, Artillerivej 5, 2300 Copenahgen S Albertsen lab, Department of Chemistry and Bioscience, Aalborg University, Denmark Rasmus Kirkegaard et al

Denmark/ALAB-SSI157/2020 EPI_ISL_429373 2020-03-09 Department of Virus and Microbiological Special Diagnostics, Statens Serum Institut, Copenhagen, Denmark, Artillerivej 5, 2300 Copenahgen S Albertsen lab, Department of Chemistry and Bioscience, Aalborg University, Denmark Rasmus Kirkegaard et al

Denmark/ALAB-SSI158/2020 EPI_ISL_429374 2020-03-09 Department of Virus and Microbiological Special Diagnostics, Statens Serum Institut, Copenhagen, Denmark, Artillerivej 5, 2300 Copenahgen S Albertsen lab, Department of Chemistry and Bioscience, Aalborg University, Denmark Rasmus Kirkegaard et al

Denmark/ALAB-SSI159/2020 EPI_ISL_429375 2020-03-09 Department of Virus and Microbiological Special Diagnostics, Statens Serum Institut, Copenhagen, Denmark, Artillerivej 5, 2300 Copenahgen S Albertsen lab, Department of Chemistry and Bioscience, Aalborg University, Denmark Rasmus Kirkegaard et al

Denmark/ALAB-SSI160/2020 EPI_ISL_429376 2020-03-09 Department of Virus and Microbiological Special Diagnostics, Statens Serum Institut, Copenhagen, Denmark, Artillerivej 5, 2300 Copenahgen S Albertsen lab, Department of Chemistry and Bioscience, Aalborg University, Denmark Rasmus Kirkegaard et al

Denmark/ALAB-SSI162/2020 EPI_ISL_429377 2020-03-09 Department of Virus and Microbiological Special Diagnostics, Statens Serum Institut, Copenhagen, Denmark, Artillerivej 5, 2300 Copenahgen S Albertsen lab, Department of Chemistry and Bioscience, Aalborg University, Denmark Rasmus Kirkegaard et al

Denmark/ALAB-SSI163/2020 EPI_ISL_429378 2020-03-09 Department of Virus and Microbiological Special Diagnostics, Statens Serum Institut, Copenhagen, Denmark, Artillerivej 5, 2300 Copenahgen S Albertsen lab, Department of Chemistry and Bioscience, Aalborg University, Denmark Rasmus Kirkegaard et al

Denmark/ALAB-SSI164/2020 EPI_ISL_429379 2020-03-09 Department of Virus and Microbiological Special Diagnostics, Statens Serum Institut, Copenhagen, Denmark, Artillerivej 5, 2300 Copenahgen S Albertsen lab, Department of Chemistry and Bioscience, Aalborg University, Denmark Rasmus Kirkegaard et al

Denmark/ALAB-SSI167/2020 EPI_ISL_429381 2020-03-09 Department of Virus and Microbiological Special Diagnostics, Statens Serum Institut, Copenhagen, Denmark, Artillerivej 5, 2300 Copenahgen S Albertsen lab, Department of Chemistry and Bioscience, Aalborg University, Denmark Rasmus Kirkegaard et al

Denmark/ALAB-SSI168/2020 EPI_ISL_429382 2020-03-09 Department of Virus and Microbiological Special Diagnostics, Statens Serum Institut, Copenhagen, Denmark, Artillerivej 5, 2300 Copenahgen S Albertsen lab, Department of Chemistry and Bioscience, Aalborg University, Denmark Rasmus Kirkegaard et al

Denmark/ALAB-SSI169/2020 EPI_ISL_429383 2020-03-09 Department of Virus and Microbiological Special Diagnostics, Statens Serum Institut, Copenhagen, Denmark, Artillerivej 5, 2300 Copenahgen S Albertsen lab, Department of Chemistry and Bioscience, Aalborg University, Denmark Rasmus Kirkegaard et al

Denmark/ALAB-SSI171/2020 EPI_ISL_429384 2020-03-09 Department of Virus and Microbiological Special Diagnostics, Statens Serum Institut, Copenhagen, Denmark, Artillerivej 5, 2300 Copenahgen S Albertsen lab, Department of Chemistry and Bioscience, Aalborg University, Denmark Rasmus Kirkegaard et al

Denmark/ALAB-SSI172/2020 EPI_ISL_429385 2020-03-09 Department of Virus and Microbiological Special Diagnostics, Statens Serum Institut, Copenhagen, Denmark, Artillerivej 5, 2300 Copenahgen S Albertsen lab, Department of Chemistry and Bioscience, Aalborg University, Denmark Rasmus Kirkegaard et al

Denmark/ALAB-SSI173/2020 EPI_ISL_429386 2020-03-09 Department of Virus and Microbiological Special Diagnostics, Statens Serum Institut, Copenhagen, Denmark, Artillerivej 5, 2300 Copenahgen S Albertsen lab, Department of Chemistry and Bioscience, Aalborg University, Denmark Rasmus Kirkegaard et al

Denmark/ALAB-SSI174/2020 EPI_ISL_429387 2020-03-09 Department of Virus and Microbiological Special Diagnostics, Statens Serum Institut, Copenhagen, Denmark, Artillerivej 5, 2300 Copenahgen S Albertsen lab, Department of Chemistry and Bioscience, Aalborg University, Denmark Rasmus Kirkegaard et al

Denmark/ALAB-SSI194/2020 EPI_ISL_429395 2020-03-09 Department of Virus and Microbiological Special Diagnostics, Statens Serum Institut, Copenhagen, Denmark, Artillerivej 5, 2300 Copenahgen S Albertsen lab, Department of Chemistry and Bioscience, Aalborg University, Denmark Rasmus Kirkegaard et al

Denmark/ALAB-SSI195/2020 EPI_ISL_429396 2020-03-09 Department of Virus and Microbiological Special Diagnostics, Statens Serum Institut, Copenhagen, Denmark, Artillerivej 5, 2300 Copenahgen S Albertsen lab, Department of Chemistry and Bioscience, Aalborg University, Denmark Rasmus Kirkegaard et al

Denmark/ALAB-SSI201/2020 EPI_ISL_429399 2020-03-09 Department of Virus and Microbiological Special Diagnostics, Statens Serum Institut, Copenhagen, Denmark, Artillerivej 5, 2300 Copenahgen S Albertsen lab, Department of Chemistry and Bioscience, Aalborg University, Denmark Rasmus Kirkegaard et al

Denmark/ALAB-SSI204/2020 EPI_ISL_429402 2020-03-09 Department of Virus and Microbiological Special Diagnostics, Statens Serum Institut, Copenhagen, Denmark, Artillerivej 5, 2300 Copenahgen S Albertsen lab, Department of Chemistry and Bioscience, Aalborg University, Denmark Rasmus Kirkegaard et al

Denmark/ALAB-SSI235/2020 EPI_ISL_429414 2020-03-09 Department of Virus and Microbiological Special Diagnostics, Statens Serum Institut, Copenhagen, Denmark, Artillerivej 5, 2300 Copenahgen S Albertsen lab, Department of Chemistry and Bioscience, Aalborg University, Denmark Rasmus Kirkegaard et al

England/20110058706/2020 EPI_ISL_417311 2020-03-09 Respiratory Virus Unit, Microbiology Services Colindale, Public Health England Respiratory Virus Unit, Microbiology Services Colindale, Public Health England Monica Galiano et al

England/20110136302/2020 EPI_ISL_418668 2020-03-09 Respiratory Virus Unit, Microbiology Services Colindale, Public Health England Respiratory Virus Unit, Microbiology Services Colindale, Public Health England Monica Galiano et al

England/20129059204/2020 EPI_ISL_424054 2020-03-09 Respiratory Virus Unit, Microbiology Services Colindale, Public Health England Respiratory Virus Unit, Microbiology Services Colindale, Public Health England Monica Galiano et al

England/BRIS-121879/2020 EPI_ISL_440178 2020-03-09 PHE South West Regional Laboratory, National Infection Service Wellcome Sanger Institute for the COVID-19 Genomics UK Consortium Stephanie Hutchings et al

England/CAMB-846C6/2020 EPI_ISL_440538 2020-03-09 Department of Pathology, University of Cambridge Wellcome Sanger Institute for the COVID-19 Genomics UK Consortium Luke W Meredith et al

England/CAMB-84A42/2020 EPI_ISL_440533 2020-03-09 Department of Pathology, University of Cambridge Wellcome Sanger Institute for the COVID-19 Genomics UK Consortium Luke W Meredith et al

England/CAMB-84ACA/2020 EPI_ISL_440487 2020-03-09 Department of Pathology, University of Cambridge Wellcome Sanger Institute for the COVID-19 Genomics UK Consortium Luke W Meredith et al

England/SHEF-BFCFD/2020 EPI_ISL_416734 2020-03-09 Virology Department, Sheffield Teaching Hospitals NHS Foundation Trust Department of Infection, Immunity and Cardiovascular Disease, The Florey Institute, The Medical School, University of Sheffield Thushan de Silva et al

England/SHEF-BFD36/2020 EPI_ISL_416738 2020-03-09 Virology Department, Sheffield Teaching Hospitals NHS Foundation Trust Department of Infection, Immunity and Cardiovascular Disease, The Florey Institute, The Medical School, University of Sheffield Thushan de Silva et al

France/HF2174/2020 EPI_ISL_415654 2020-03-09 Centre Hospitalier Compi√®gne Laboratoire de Biologie National Reference Center for Viruses of Respiratory Infections, Institut Pasteur, Paris M√©lnie Albert et al

France/HF2381/2020 EPI_ISL_418226 2020-03-09 EHPAD - R√©sidences les C√®dres National Reference Center for Viruses of Respiratory Infections, Institut Pasteur, Paris M√©lanie Albert et al

Greece/133/2020 EPI_ISL_434455 2020-03-09 Laboratory of Microbiology, Medical School, National and Kapodistrian University of Athens Laboratory of Biology, Department of Medicine, Democritus University of Thrace Kassela K. et al

Greece/148/2020 EPI_ISL_434484 2020-03-09 Laboratory of Microbiology, Medical School, National and Kapodistrian University of Athens Laboratory of Biology, Department of Medicine, Democritus University of Thrace Kassela K. et al

Greece/238_31927/2020 EPI_ISL_447645 2020-03-09 unknown Department of Medicine Kassela et al

Greece/259_31928/2020 EPI_ISL_447651 2020-03-09 unknown Department of Medicine Kassela et al

Iceland/73/2020 EPI_ISL_417861 2020-03-09 The National University Hospital of Iceland deCODE genetics Daniel F Gudbjartsson et al

Iceland/77/2020 EPI_ISL_417862 2020-03-09 The National University Hospital of Iceland deCODE genetics Daniel F Gudbjartsson et al

Iceland/78/2020 EPI_ISL_417863 2020-03-09 The National University Hospital of Iceland deCODE genetics Daniel F Gudbjartsson et al

Iceland/80/2020 EPI_ISL_417865 2020-03-09 The National University Hospital of Iceland deCODE genetics Daniel F Gudbjartsson et al

Iceland/81/2020 EPI_ISL_417866 2020-03-09 The National University Hospital of Iceland deCODE genetics Daniel F Gudbjartsson et al

Iceland/82/2020 EPI_ISL_417867 2020-03-09 The National University Hospital of Iceland deCODE genetics Daniel F Gudbjartsson et al

Iceland/83/2020 EPI_ISL_417868 2020-03-09 The National University Hospital of Iceland deCODE genetics Daniel F Gudbjartsson et al

Iran/KHGRC-1.1-IPI-8206/2020 EPI_ISL_442523 2020-03-09 Pasteur Institute of Iran Kawsar Human Genetic Research Company Sirous Zeinali et al

Israel/CVL-n3120/2020 EPI_ISL_447251 2020-03-09 Central Virology Laboratory Central Virology Laboratory Neta Zuckerman et al

Japan/Hu_Kng_19-437/2020 EPI_ISL_420890 2020-03-09 unknown Takayuki Hishiki Kanagawa Prefectural Institute of Public Health Hishiki et al

Japan/P3-1/2020 EPI_ISL_419299 2020-03-09 Ishikawa Prefectural Institute of Public Health and Environmental Science Pathogen Genomics Center, National Institute of Infectious Diseases Tsuyoshi Sekizuka et al

Netherlands/Flevoland_1/2020 EPI_ISL_415460 2020-03-09 Dutch COVID-19 response team Erasmus Medical Center David Nieuwenhuijse et al

Netherlands/Gelderland_2/2020 EPI_ISL_415462 2020-03-09 Dutch COVID-19 response team Erasmus Medical Center David Nieuwenhuijse et al

Netherlands/Gelderland_3/2020 EPI_ISL_415463 2020-03-09 Dutch COVID-19 response team Erasmus Medical Center David Nieuwenhuijse et al

Netherlands/Gelderland_6/2020 EPI_ISL_422642 2020-03-09 Dutch COVID-19 response team Erasmus Medical Center Bas Oude Munnink et al

Netherlands/NA_10/2020 EPI_ISL_415466 2020-03-09 Dutch COVID-19 response team Erasmus Medical Center David Nieuwenhuijse et al

Netherlands/NA_17/2020 EPI_ISL_415473 2020-03-09 Dutch COVID-19 response team Erasmus Medical Center David Nieuwenhuijse et al

Netherlands/NA_18/2020 EPI_ISL_415474 2020-03-09 Dutch COVID-19 response team Erasmus Medical Center David Nieuwenhuijse et al

Netherlands/NA_23/2020 EPI_ISL_415480 2020-03-09 Dutch COVID-19 response team Erasmus Medical Center David Nieuwenhuijse et al

Netherlands/NA_25/2020 EPI_ISL_415482 2020-03-09 Dutch COVID-19 response team Erasmus Medical Center David Nieuwenhuijse et al

Netherlands/NA_26/2020 EPI_ISL_415483 2020-03-09 Dutch COVID-19 response team Erasmus Medical Center David Nieuwenhuijse et al

Netherlands/NA_48/2020 EPI_ISL_422809 2020-03-09 Dutch COVID-19 response team Erasmus Medical Center Bas Oude Munnink et al

Netherlands/NA_49/2020 EPI_ISL_422810 2020-03-09 Dutch COVID-19 response team Erasmus Medical Center Bas Oude Munnink et al

Netherlands/NA_59/2020 EPI_ISL_422820 2020-03-09 Dutch COVID-19 response team Erasmus Medical Center Bas Oude Munnink et al

Netherlands/NA_60/2020 EPI_ISL_422821 2020-03-09 Dutch COVID-19 response team Erasmus Medical Center Bas Oude Munnink et al

Netherlands/NA_61/2020 EPI_ISL_422822 2020-03-09 Dutch COVID-19 response team Erasmus Medical Center Bas Oude Munnink et al

Netherlands/NA_62/2020 EPI_ISL_422823 2020-03-09 Dutch COVID-19 response team Erasmus Medical Center Bas Oude Munnink et al

Netherlands/NA_67/2020 EPI_ISL_422827 2020-03-09 Dutch COVID-19 response team Erasmus Medical Center Bas Oude Munnink et al

Netherlands/NA_7/2020 EPI_ISL_415496 2020-03-09 Dutch COVID-19 response team Erasmus Medical Center David Nieuwenhuijse et al

Netherlands/NA_77/2020 EPI_ISL_422837 2020-03-09 Dutch COVID-19 response team Erasmus Medical Center Bas Oude Munnink et al

Netherlands/NA_78/2020 EPI_ISL_422838 2020-03-09 Dutch COVID-19 response team Erasmus Medical Center Bas Oude Munnink et al

Netherlands/NA_8/2020 EPI_ISL_415497 2020-03-09 Dutch COVID-19 response team Erasmus Medical Center David Nieuwenhuijse et al

Netherlands/NA_9/2020 EPI_ISL_415498 2020-03-09 Dutch COVID-19 response team Erasmus Medical Center David Nieuwenhuijse et al

Netherlands/NoordBrabant_103/2020 EPI_ISL_422862 2020-03-09 Dutch COVID-19 response team Erasmus Medical Center Bas Oude Munnink et al

Netherlands/NoordBrabant_104/2020 EPI_ISL_422863 2020-03-09 Dutch COVID-19 response team Erasmus Medical Center Bas Oude Munnink et al

Netherlands/NoordBrabant_35/2020 EPI_ISL_414544 2020-03-09 Dutch COVID-19 response team Erasmus Medical Center David Nieuwenhuijse et al

Netherlands/NoordBrabant_45/2020 EPI_ISL_415502 2020-03-09 Dutch COVID-19 response team Erasmus Medical Center David Nieuwenhuijse et al

Netherlands/NoordBrabant_52/2020 EPI_ISL_415508 2020-03-09 Dutch COVID-19 response team Erasmus Medical Center David Nieuwenhuijse et al

Netherlands/NoordBrabant_54/2020 EPI_ISL_415510 2020-03-09 Dutch COVID-19 response team Erasmus Medical Center David Nieuwenhuijse et al

Netherlands/NoordBrabant_55/2020 EPI_ISL_415511 2020-03-09 Dutch COVID-19 response team Erasmus Medical Center David Nieuwenhuijse et al

Netherlands/NoordBrabant_56/2020 EPI_ISL_415512 2020-03-09 Dutch COVID-19 response team Erasmus Medical Center David Nieuwenhuijse et al

Netherlands/NoordBrabant_74/2020 EPI_ISL_422870 2020-03-09 Dutch COVID-19 response team Erasmus Medical Center Bas Oude Munnink et al

Netherlands/NoordBrabant_75/2020 EPI_ISL_422871 2020-03-09 Dutch COVID-19 response team Erasmus Medical Center Bas Oude Munnink et al

Netherlands/NoordBrabant_76/2020 EPI_ISL_422872 2020-03-09 Dutch COVID-19 response team Erasmus Medical Center Bas Oude Munnink et al

Netherlands/NoordBrabant_85/2020 EPI_ISL_422879 2020-03-09 Dutch COVID-19 response team Erasmus Medical Center Bas Oude Munnink et al

Netherlands/NoordBrabant_86/2020 EPI_ISL_422880 2020-03-09 Dutch COVID-19 response team Erasmus Medical Center Bas Oude Munnink et al

Netherlands/NoordBrabant_87/2020 EPI_ISL_422881 2020-03-09 Dutch COVID-19 response team Erasmus Medical Center Bas Oude Munnink et al

Netherlands/NoordBrabant_93/2020 EPI_ISL_422887 2020-03-09 Dutch COVID-19 response team Erasmus Medical Center Bas Oude Munnink et al

Netherlands/ZuidHolland_25/2020 EPI_ISL_415529 2020-03-09 Dutch COVID-19 response team Erasmus Medical Center David Nieuwenhuijse et al

Netherlands/ZuidHolland_26/2020 EPI_ISL_415530 2020-03-09 Dutch COVID-19 response team Erasmus Medical Center David Nieuwenhuijse et al

Netherlands/ZuidHolland_27/2020 EPI_ISL_415531 2020-03-09 Dutch COVID-19 response team Erasmus Medical Center David Nieuwenhuijse et al

Netherlands/ZuidHolland_28/2020 EPI_ISL_415532 2020-03-09 Dutch COVID-19 response team Erasmus Medical Center David Nieuwenhuijse et al

Netherlands/ZuidHolland_29/2020 EPI_ISL_415533 2020-03-09 Dutch COVID-19 response team Erasmus Medical Center David Nieuwenhuijse et al

Netherlands/ZuidHolland_32/2020 EPI_ISL_422904 2020-03-09 Dutch COVID-19 response team Erasmus Medical Center Bas Oude Munnink et al

Norway/1951/2020 EPI_ISL_420145 2020-03-09 Forde Hospital Department of Microbiology Norwegian Institute of Public Health, Department of Virology Kathrine Stene-Johansen et al

Norway/2084/2020 EPI_ISL_420150 2020-03-09 Oslo University Hospital, Department of Medical Microbiology Norwegian Institute of Public Health, Department of Virology Kathrine Stene-Johansen et al

Portugal/PT0029/2020 EPI_ISL_418014 2020-03-09 CHULC - H Curry Cabral Instituto Nacional de Saude (INSA) Guiomar et al et al

Portugal/PT0030/2020 EPI_ISL_418015 2020-03-09 CHULC - H Curry Cabral Instituto Nacional de Saude (INSA) Guiomar et al et al

Scotland/CVR07/2020 EPI_ISL_415630 2020-03-09 West of Scotland Specialist Virology Centre, NHSGGC MRC-University of Glasgow Centre for Virus Research Kathy Smollett et al

Scotland/CVR17/2020 EPI_ISL_425709 2020-03-09 West of Scotland Specialist Virology Centre, NHSGGC / MRC-University of Glasgow Centre for Virus Research COVID-19 Genomics UK (COG-UK) Consortium Ana da Silva Filipe et al

Scotland/CVR68/2020 EPI_ISL_425799 2020-03-09 West of Scotland Specialist Virology Centre, NHSGGC / MRC-University of Glasgow Centre for Virus Research COVID-19 Genomics UK (COG-UK) Consortium Ana da Silva Filipe et al

Scotland/CVR70/2020 EPI_ISL_425801 2020-03-09 West of Scotland Specialist Virology Centre, NHSGGC / MRC-University of Glasgow Centre for Virus Research COVID-19 Genomics UK (COG-UK) Consortium Ana da Silva Filipe et al

Scotland/CVR79/2020 EPI_ISL_425809 2020-03-09 West of Scotland Specialist Virology Centre, NHSGGC / MRC-University of Glasgow Centre for Virus Research COVID-19 Genomics UK (COG-UK) Consortium Ana da Silva Filipe et al

Scotland/EDB039/2020 EPI_ISL_425845 2020-03-09 Virology Department, Royal Infirmary of Edinburgh, NHS Lothian / School of Biological Sciences, University of Edinburgh / Institute of Genetics and Molecular Medicine, University of Edinburgh COVID-19 Genomics UK (COG-UK) Consortium McHugh M et al

Singapore/30/2020 EPI_ISL_420107 2020-03-09 National Centre for Infectious Diseases Programme in Emerging Infectious Diseases, Duke-NUS Medical School Danielle E Anderson et al

Spain/Madrid-LP32/2020 EPI_ISL_430720 2020-03-09 Hospital Universitario La Paz Hospital Universitario 12 de Octubre Elias Dahdouh et al

Spain/Madrid_H12_36/2020 EPI_ISL_428699 2020-03-09 Hospital Universitario 12 de Octubre Hospital Universitario 12 de Octubre Ra√∫l Recio et al

Spain/Madrid_LP11_2271/2020 EPI_ISL_428674 2020-03-09 Hospital Universitario La Paz Hospital Universitario 12 de Octubre Elias Dahdouh et al

Spain/Madrid_LP12_21/2020 EPI_ISL_417972 2020-03-09 Hospital Universitario La Paz Hospital Universitario La Paz Elias Dahdouh et al

Spain/Madrid_LP14_3/2020 EPI_ISL_417975 2020-03-09 Hospital Universitario La Paz Hospital Universitario La Paz Elias Dahdouh et al

Spain/Madrid_LP15_4/2020 EPI_ISL_417978 2020-03-09 Hospital Universitario La Paz Hospital Universitario La Paz Elias Dahdouh et al

Spain/Madrid_LP16_6193/2020 EPI_ISL_428675 2020-03-09 Hospital Universitario La Paz Hospital Universitario 12 de Octubre Elias Dahdouh et al

Spain/Madrid_LP24_5999/2020 EPI_ISL_428680 2020-03-09 Hospital Universitario La Paz Hospital Universitario 12 de Octubre Elias Dahdouh et al

Spain/Valencia12/2020 EPI_ISL_419676 2020-03-09 Servicio de Microbiolog√≠a. Consorcio Hospital General Universitario de Valencia Sequencing and Bioinformatics Service and Molecular Epidemiology Research Group. FISABIO-Public Health Maria Dolores Ocete et al

Spain/Valencia13/2020 EPI_ISL_419677 2020-03-09 Servicio de Microbiolog√≠a. Consorcio Hospital General Universitario de Valencia Sequencing and Bioinformatics Service and Molecular Epidemiology Research Group. FISABIO-Public Health Giuseppe D'Auria et al

Spain/Valencia14/2020 EPI_ISL_419678 2020-03-09 Servicio de Microbiolog√≠a. Consorcio Hospital General Universitario de Valencia Sequencing and Bioinformatics Service and Molecular Epidemiology Research Group. FISABIO-Public Health Griselda De Marco et al

Spain/Valencia27/2020 EPI_ISL_420112 2020-03-09 Servicio de Microbiolog√≠a. Consorcio Hospital General Universitario de Valencia Sequencing and Bioinformatics Service and Molecular Epidemiology Research Group. FISABIO-Public Health Lidia Ruiz Roldan et al

Spain/Valencia28/2020 EPI_ISL_420113 2020-03-09 Servicio de Microbiolog√≠a. Consorcio Hospital General Universitario de Valencia Sequencing and Bioinformatics Service and Molecular Epidemiology Research Group. FISABIO-Public Health Beatriz Beamud et al

Spain/Valencia29/2020 EPI_ISL_420114 2020-03-09 Servicio de Microbiolog√≠a. Consorcio Hospital General Universitario de Valencia Sequencing and Bioinformatics Service and Molecular Epidemiology Research Group. FISABIO-Public Health Griselda De Marco et al

Spain/Valencia30/2020 EPI_ISL_420115 2020-03-09 Servicio de Microbiolog√≠a. Consorcio Hospital General Universitario de Valencia Sequencing and Bioinformatics Service and Molecular Epidemiology Research Group. FISABIO-Public Health Marta Pla Diaz et al

Spain/Valencia31/2020 EPI_ISL_420116 2020-03-09 Servicio de Microbiolog√≠a. Consorcio Hospital General Universitario de Valencia Sequencing and Bioinformatics Service and Molecular Epidemiology Research Group. FISABIO-Public Health Neris Garcia-Gonzalez et al

Spain/Valencia32/2020 EPI_ISL_420117 2020-03-09 Servicio de Microbiolog√≠a. Consorcio Hospital General Universitario de Valencia Sequencing and Bioinformatics Service and Molecular Epidemiology Research Group. FISABIO-Public Health Loreto Ferr√∫s Abad et al

Spain/Valencia33/2020 EPI_ISL_420118 2020-03-09 Servicio de Microbiolog√≠a. Consorcio Hospital General Universitario de Valencia Sequencing and Bioinformatics Service and Molecular Epidemiology Research Group. FISABIO-Public Health Inma Gal√°n Vendrell et al

Spain/Valencia34/2020 EPI_ISL_420119 2020-03-09 Servicio de Microbiolog√≠a. Consorcio Hospital General Universitario de Valencia Sequencing and Bioinformatics Service and Molecular Epidemiology Research Group. FISABIO-Public Health Paula Ruiz-Hueso et al

Spain/Valencia35/2020 EPI_ISL_420120 2020-03-09 Servicio de Microbiolog√≠a. Consorcio Hospital General Universitario de Valencia Sequencing and Bioinformatics Service and Molecular Epidemiology Research Group. FISABIO-Public Health Mariana Reyes-Prieto et al

Spain/Valencia36/2020 EPI_ISL_420121 2020-03-09 Servicio de Microbiolog√≠a. Consorcio Hospital General Universitario de Valencia Sequencing and Bioinformatics Service and Molecular Epidemiology Research Group. FISABIO-Public Health Vicente Soriano Chirona et al

Spain/Valencia37/2020 EPI_ISL_420122 2020-03-09 Servicio de Microbiolog√≠a. Consorcio Hospital General Universitario de Valencia Sequencing and Bioinformatics Service and Molecular Epidemiology Research Group. FISABIO-Public Health Maria Alma Bracho et al

Spain/Valencia38/2020 EPI_ISL_420123 2020-03-09 Servicio de Microbiolog√≠a. Consorcio Hospital General Universitario de Valencia Sequencing and Bioinformatics Service and Molecular Epidemiology Research Group. FISABIO-Public Health Maria Dolores Ocete et al

Spain/Valencia39/2020 EPI_ISL_420124 2020-03-09 Servicio de Microbiolog√≠a. Consorcio Hospital General Universitario de Valencia Sequencing and Bioinformatics Service and Molecular Epidemiology Research Group. FISABIO-Public Health Concepcion Gimeno et al

Sweden/20-50230/2020 EPI_ISL_429155 2020-03-09 Klinisk mikrobiologi Orebro The Public Health Agency of Sweden Martin Sundqvist et al

Sweden/20-50231/2020 EPI_ISL_430863 2020-03-09 Klinisk mikrobiologi Orebro The Public Health Agency of Sweden Martin Sundqvist et al

Sweden/20-50232/2020 EPI_ISL_429156 2020-03-09 Klinisk mikrobiologi Orebro The Public Health Agency of Sweden Martin Sundqvist et al

Taiwan/CGMH-CGU-07/2020 EPI_ISL_417520 2020-03-09 Laboratory Medicine Department of Laboratory Medicine, Lin-Kou Chang Gung Memorial Hospital, Taoyuan, Taiwan Kuo-Chien Tsao et al

USA/AZ_8132/2020 EPI_ISL_424848 2020-03-09 AZ SPHL, Arizona Department of Health Services Pathogen Discovery, Respiratory Viruses Branch, Division of Viral Diseases, Centers for Disease Control and Prevention Yan Li et al

USA/AZ_8135/2020 EPI_ISL_424849 2020-03-09 AZ SPHL, Arizona Department of Health Services Pathogen Discovery, Respiratory Viruses Branch, Division of Viral Diseases, Centers for Disease Control and Prevention Yan Li et al

USA/CA-SCCPHD-UC142/2020 EPI_ISL_435621 2020-03-09 Santa Clara County Public Health Department Chiu Laboratory, University of California, San Francisco Xianding Deng et al

USA/IN_2001/2020 EPI_ISL_424866 2020-03-09 IN State Department of Health Laboratory Services Pathogen Discovery, Respiratory Viruses Branch, Division of Viral Diseases, Centers for Disease Control and Prevention Yan Li et al

USA/LA_0842/2020 EPI_ISL_424868 2020-03-09 LA Office of Public Health Laboratories Pathogen Discovery, Respiratory Viruses Branch, Division of Viral Diseases, Centers for Disease Control and Prevention Yan Li et al

USA/MN3-MDH3/2020 EPI_ISL_414590 2020-03-09 Minnesota Department of Health, Public Health Laboratory Minnesota Department of Health, Public Health Laboratory Matt Plumb et al

USA/NV_0052/2020 EPI_ISL_424879 2020-03-09 NV-Southern Nevada Public Health Laboratory Pathogen Discovery, Respiratory Viruses Branch, Division of Viral Diseases, Centers for Disease Control and Prevention Yan Li et al

USA/NY-PV09413/2020 EPI_ISL_450093 2020-03-09 MSHS Clinical Microbiology Laboratories MSHS Pathogen Surveillance Program Ana S. Gonzalez-Reiche et al

USA/NY-PV09449/2020 EPI_ISL_450123 2020-03-09 MSHS Clinical Microbiology Laboratories MSHS Pathogen Surveillance Program Ana S. Gonzalez-Reiche et al

USA/NY-SURV0162/2020 EPI_ISL_436051 2020-03-09 NYC Department of Health and Mental Hygiene Pathogen Discovery, Respiratory Viruses Branch, Division of Viral Diseases, Centers for Disease Control and Prevention Ying Tao et al

USA/NY-SURV0165/2020 EPI_ISL_436052 2020-03-09 NYC Department of Health and Mental Hygiene Pathogen Discovery, Respiratory Viruses Branch, Division of Viral Diseases, Centers for Disease Control and Prevention Ying Tao et al

USA/NY-SURV0166/2020 EPI_ISL_436053 2020-03-09 NYC Department of Health and Mental Hygiene Pathogen Discovery, Respiratory Viruses Branch, Division of Viral Diseases, Centers for Disease Control and Prevention Ying Tao et al

USA/NY-SURV0168/2020 EPI_ISL_436054 2020-03-09 NYC Department of Health and Mental Hygiene Pathogen Discovery, Respiratory Viruses Branch, Division of Viral Diseases, Centers for Disease Control and Prevention Ying Tao et al

USA/NY-SURV0171/2020 EPI_ISL_436055 2020-03-09 NYC Department of Health and Mental Hygiene Pathogen Discovery, Respiratory Viruses Branch, Division of Viral Diseases, Centers for Disease Control and Prevention Ying Tao et al

USA/NY-SURV0175/2020 EPI_ISL_436056 2020-03-09 NYC Department of Health and Mental Hygiene Pathogen Discovery, Respiratory Viruses Branch, Division of Viral Diseases, Centers for Disease Control and Prevention Ying Tao et al

USA/NY-SURV0302/2020 EPI_ISL_436060 2020-03-09 NYC Department of Health and Mental Hygiene Pathogen Discovery, Respiratory Viruses Branch, Division of Viral Diseases, Centers for Disease Control and Prevention Ying Tao et al

USA/NY-SURV0331/2020 EPI_ISL_436062 2020-03-09 NYC Department of Health and Mental Hygiene Pathogen Discovery, Respiratory Viruses Branch, Division of Viral Diseases, Centers for Disease Control and Prevention Ying Tao et al

USA/NY-SURV0334/2020 EPI_ISL_436063 2020-03-09 NYC Department of Health and Mental Hygiene Pathogen Discovery, Respiratory Viruses Branch, Division of Viral Diseases, Centers for Disease Control and Prevention Ying Tao et al

USA/NY-SURV0440/2020 EPI_ISL_436066 2020-03-09 NYC Department of Health and Mental Hygiene Pathogen Discovery, Respiratory Viruses Branch, Division of Viral Diseases, Centers for Disease Control and Prevention Ying Tao et al

USA/NY-SURV0444/2020 EPI_ISL_436067 2020-03-09 NYC Department of Health and Mental Hygiene Pathogen Discovery, Respiratory Viruses Branch, Division of Viral Diseases, Centers for Disease Control and Prevention Ying Tao et al

USA/NY-SURV0472/2020 EPI_ISL_436068 2020-03-09 NYC Department of Health and Mental Hygiene Pathogen Discovery, Respiratory Viruses Branch, Division of Viral Diseases, Centers for Disease Control and Prevention Ying Tao et al

USA/NY-SURV0475/2020 EPI_ISL_436069 2020-03-09 NYC Department of Health and Mental Hygiene Pathogen Discovery, Respiratory Viruses Branch, Division of Viral Diseases, Centers for Disease Control and Prevention Ying Tao et al

USA/NY-SURV0874/2020 EPI_ISL_436076 2020-03-09 NYC Department of Health and Mental Hygiene Pathogen Discovery, Respiratory Viruses Branch, Division of Viral Diseases, Centers for Disease Control and Prevention Ying Tao et al

USA/NY-SURV097/2020 EPI_ISL_436049 2020-03-09 NYC Department of Health and Mental Hygiene Pathogen Discovery, Respiratory Viruses Branch, Division of Viral Diseases, Centers for Disease Control and Prevention Ying Tao et al

USA/PA_4395/2020 EPI_ISL_424885 2020-03-09 PA Department of Health, Bureau of Laboratories Pathogen Discovery, Respiratory Viruses Branch, Division of Viral Diseases, Centers for Disease Control and Prevention Yan Li et al

USA/RI_0882/2020 EPI_ISL_426435 2020-03-09 RI State Health Laboratories Pathogen Discovery, Respiratory Viruses Branch, Division of Viral Diseases, Centers for Disease Control and Prevention Krista Queen et al

USA/TX-HMH035/2020 EPI_ISL_434744 2020-03-09 Houston Methodist Hospital Houston Methodist Hospital S. Wesley Long et al

USA/TX-HMH036/2020 EPI_ISL_434745 2020-03-09 Houston Methodist Hospital Houston Methodist Hospital S. Wesley Long et al

USA/VA-DCLS-00010/2020 EPI_ISL_419262 2020-03-09 Virginia DCLS Virginia DCLS Virginia DCLS et al

USA/VA-DCLS-0009/2020 EPI_ISL_419261 2020-03-09 Virginia DCLS Virginia DCLS Virginia DCLS et al

USA/WA-S280/2020 EPI_ISL_430134 2020-03-09 Seattle Flu Study Seattle Flu Study Chu et al et al

USA/WA-S366/2020 EPI_ISL_434126 2020-03-09 Washington State Department of Health Seattle Flu Study Chu et al et al

USA/WA-UW144/2020 EPI_ISL_416682 2020-03-09 UW Virology Lab UW Virology Lab Pavitra Roychoudhury et al

USA/WA-UW42/2020 EPI_ISL_415607 2020-03-09 UW Virology Lab UW Virology Lab Pavitra Roychoudhury et al

USA/WA-UW46/2020 EPI_ISL_415611 2020-03-09 UW Virology Lab UW Virology Lab Pavitra Roychoudhury et al

USA/WA-UW47/2020 EPI_ISL_415612 2020-03-09 UW Virology Lab UW Virology Lab Pavitra Roychoudhury et al

USA/WA-UW48/2020 EPI_ISL_415613 2020-03-09 UW Virology Lab UW Virology Lab Pavitra Roychoudhury et al

USA/WA-UW49/2020 EPI_ISL_415614 2020-03-09 UW Virology Lab UW Virology Lab Pavitra Roychoudhury et al

USA/WA-UW52/2020 EPI_ISL_415617 2020-03-09 UW Virology Lab UW Virology Lab Pavitra Roychoudhury et al

USA/WA-UW54/2020 EPI_ISL_415619 2020-03-09 UW Virology Lab UW Virology Lab Pavitra Roychoudhury et al

USA/WA-UW55/2020 EPI_ISL_415620 2020-03-09 UW Virology Lab UW Virology Lab Pavitra Roychoudhury et al

USA/WA-UW56/2020 EPI_ISL_415621 2020-03-09 UW Virology Lab UW Virology Lab Pavitra Roychoudhury et al

USA/WA-UW57/2020 EPI_ISL_415622 2020-03-09 UW Virology Lab UW Virology Lab Pavitra Roychoudhury et al

USA/WA-UW59/2020 EPI_ISL_415624 2020-03-09 UW Virology Lab UW Virology Lab Pavitra Roychoudhury et al

USA/WA-UW60/2020 EPI_ISL_415625 2020-03-09 UW Virology Lab UW Virology Lab Pavitra Roychoudhury et al

USA/WA-UW62/2020 EPI_ISL_415627 2020-03-09 UW Virology Lab UW Virology Lab Pavitra Roychoudhury et al

USA/WA-UW64/2020 EPI_ISL_415592 2020-03-09 UW Virology Lab UW Virology Lab Pavitra Roychoudhury et al

USA/WA-UW67/2020 EPI_ISL_415595 2020-03-09 UW Virology Lab UW Virology Lab Pavitra Roychoudhury et al

USA/WA-UW68/2020 EPI_ISL_415596 2020-03-09 UW Virology Lab UW Virology Lab Pavitra Roychoudhury et al

USA/WA-UW72/2020 EPI_ISL_415600 2020-03-09 UW Virology Lab UW Virology Lab Pavitra Roychoudhury et al

Vietnam/OUCRU0299/2020 EPI_ISL_435308 2020-03-09 National Hospital of Tropical Diseases Oxford University Clinical Research Unit, Hanoi, Vietnam Nguyen Thi Tam et al

Vietnam/OUCRU0300/2020 EPI_ISL_435310 2020-03-09 National Hospital of Tropical Diseases Oxford University Clinical Research Unit, Hanoi, Vietnam Nguyen Thi Tam et al

Australia/NSW103/2020 EPI_ISL_427657 2020-03-10 Centre for Infectious Diseases and Microbiology Public Health NSW Health Pathology - Institute of Clinical Pathology and Medical Research; Westmead Hospital; University of Sydney Rockett R et al

Australia/NSW31/2020 EPI_ISL_417392 2020-03-10 Centre for Infectious Diseases and Microbiology Public Health NSW Health Pathology - Institute of Clinical Pathology and Medical Research; Westmead Hospital; University of Sydney Rahman H et al

Australia/NSW36/2020 EPI_ISL_417394 2020-03-10 Centre for Infectious Diseases and Microbiology Public Health NSW Health Pathology - Institute of Clinical Pathology and Medical Research; Westmead Hospital; University of Sydney O‚ÄôSullivan MV et al

Australia/NSW37/2020 EPI_ISL_417395 2020-03-10 Centre for Infectious Diseases and Microbiology Public Health NSW Health Pathology - Institute of Clinical Pathology and Medical Research; Westmead Hospital; University of Sydney Sintchenko V et al

Australia/NSW38/2020 EPI_ISL_417396 2020-03-10 Centre for Infectious Diseases and Microbiology Public Health NSW Health Pathology - Institute of Clinical Pathology and Medical Research; Westmead Hospital; University of Sydney Chen SC et al

Australia/NSW41/2020 EPI_ISL_417399 2020-03-10 Centre for Infectious Diseases and Microbiology Public Health NSW Health Pathology - Institute of Clinical Pathology and Medical Research; Westmead Hospital; University of Sydney Dwyer DE et al

Australia/NSW44/2020 EPI_ISL_417400 2020-03-10 Centre for Infectious Diseases and Microbiology Public Health NSW Health Pathology - Institute of Clinical Pathology and Medical Research; Westmead Hospital; University of Sydney Rockett R et al

Australia/NSW46/2020 EPI_ISL_427658 2020-03-10 Centre for Infectious Diseases and Microbiology Public Health NSW Health Pathology - Institute of Clinical Pathology and Medical Research; Westmead Hospital; University of Sydney Sim E et al

Australia/NSW49/2020 EPI_ISL_427659 2020-03-10 Centre for Infectious Diseases and Microbiology Public Health NSW Health Pathology - Institute of Clinical Pathology and Medical Research; Westmead Hospital; University of Sydney Bachmann N et al

Australia/NSW51/2020 EPI_ISL_427660 2020-03-10 Centre for Infectious Diseases and Microbiology Public Health NSW Health Pathology - Institute of Clinical Pathology and Medical Research; Westmead Hospital; University of Sydney Rockett R et al

Australia/VIC26/2020 EPI_ISL_419745 2020-03-10 Victorian Infectious Diseases Reference Laboratory (VIDRL) Victorian Infectious Diseases Reference Laboratory and Microbiological Diagnostic Unit Public Health Laboratory, Doherty Institute Caly L. et al

Australia/VIC27/2020 EPI_ISL_419746 2020-03-10 Victorian Infectious Diseases Reference Laboratory (VIDRL) Victorian Infectious Diseases Reference Laboratory and Microbiological Diagnostic Unit Public Health Laboratory, Doherty Institute Caly L. et al

Australia/VIC28/2020 EPI_ISL_419747 2020-03-10 Victorian Infectious Diseases Reference Laboratory (VIDRL) Victorian Infectious Diseases Reference Laboratory and Microbiological Diagnostic Unit Public Health Laboratory, Doherty Institute Caly L. et al

Australia/VIC30/2020 EPI_ISL_419749 2020-03-10 Victorian Infectious Diseases Reference Laboratory (VIDRL) Victorian Infectious Diseases Reference Laboratory and Microbiological Diagnostic Unit Public Health Laboratory, Doherty Institute Caly L. et al

Australia/VIC31/2020 EPI_ISL_419750 2020-03-10 Victorian Infectious Diseases Reference Laboratory (VIDRL) Victorian Infectious Diseases Reference Laboratory and Microbiological Diagnostic Unit Public Health Laboratory, Doherty Institute Caly L. et al

Australia/VIC32/2020 EPI_ISL_419751 2020-03-10 Victorian Infectious Diseases Reference Laboratory (VIDRL) Victorian Infectious Diseases Reference Laboratory and Microbiological Diagnostic Unit Public Health Laboratory, Doherty Institute Caly L. et al

Australia/VIC33/2020 EPI_ISL_419752 2020-03-10 Victorian Infectious Diseases Reference Laboratory (VIDRL) Victorian Infectious Diseases Reference Laboratory and Microbiological Diagnostic Unit Public Health Laboratory, Doherty Institute Caly L. et al

Australia/VIC34/2020 EPI_ISL_419753 2020-03-10 Victorian Infectious Diseases Reference Laboratory (VIDRL) Victorian Infectious Diseases Reference Laboratory and Microbiological Diagnostic Unit Public Health Laboratory, Doherty Institute Caly L. et al

Australia/VIC35/2020 EPI_ISL_419755 2020-03-10 Victorian Infectious Diseases Reference Laboratory (VIDRL) Victorian Infectious Diseases Reference Laboratory and Microbiological Diagnostic Unit Public Health Laboratory, Doherty Institute Caly L. et al

Australia/VIC36/2020 EPI_ISL_419765 2020-03-10 Victorian Infectious Diseases Reference Laboratory (VIDRL) Victorian Infectious Diseases Reference Laboratory and Microbiological Diagnostic Unit Public Health Laboratory, Doherty Institute Caly L. et al

Austria/CeMM0006/2020 EPI_ISL_419659 2020-03-10 Center for Virology, Medical University of Vienna Bergthaler laboratory, CeMM Research Center for Molecular Medicine of the Austrian Academy of Sciences Alexandra Popa et al

Austria/CeMM0012/2020 EPI_ISL_419665 2020-03-10 Center for Virology, Medical University of Vienna Bergthaler laboratory, CeMM Research Center for Molecular Medicine of the Austrian Academy of Sciences Alexandra Popa et al

Austria/CeMM0034/2020 EPI_ISL_437923 2020-03-10 Institut f√ºr Virologie am Department f√ºr Hygiene, Mikrobiologie und Public Health Bergthaler laboratory, CeMM Research Center for Molecular Medicine of the Austrian Academy of Sciences Alexandra Popa et al

Austria/CeMM0039/2020 EPI_ISL_437928 2020-03-10 Institut f√ºr Virologie am Department f√ºr Hygiene, Mikrobiologie und Public Health Bergthaler laboratory, CeMM Research Center for Molecular Medicine of the Austrian Academy of Sciences Alexandra Popa et al

Austria/CeMM0116/2020 EPI_ISL_437974 2020-03-10 Institut f√ºr Virologie am Department f√ºr Hygiene, Mikrobiologie und Public Health Bergthaler laboratory, CeMM Research Center for Molecular Medicine of the Austrian Academy of Sciences Alexandra Popa et al

Austria/CeMM0183/2020 EPI_ISL_438025 2020-03-10 Center for Virology, Medical University of Vienna Bergthaler laboratory, CeMM Research Center for Molecular Medicine of the Austrian Academy of Sciences Alexandra Popa et al

Belgium/CS-031052/2020 EPI_ISL_418800 2020-03-10 KU Leuven, Clinical and Epidemiological Virology KU Leuven, Clinical and Epidemiological Virology Bert Vanmechelen et al

Belgium/SS-031047/2020 EPI_ISL_418793 2020-03-10 KU Leuven, Clinical and Epidemiological Virology KU Leuven, Clinical and Epidemiological Virology Bert Vanmechelen et al

Brazil/SC-766/2020 EPI_ISL_427305 2020-03-10 LACEN-SC - Laboratorio Central de Santa Catarina Instituto Oswaldo Cruz FIOCRUZ - Laboratory of Respiratory Viruses and Measles (LVRS) Paola Resende et al

Brazil/SC-769/2020 EPI_ISL_427306 2020-03-10 LACEN-SC - Laboratorio Central de Santa Catarina Instituto Oswaldo Cruz FIOCRUZ - Laboratory of Respiratory Viruses and Measles (LVRS) Paola Resende et al

Canada/BC_3968175/2020 EPI_ISL_418821 2020-03-10 BCCDC Public Health Laboratory BCCDC Public Health Laboratory Harrigan et al

Canada/BC_3972884/2020 EPI_ISL_418822 2020-03-10 BCCDC Public Health Laboratory BCCDC Public Health Laboratory Harrigan et al

Canada/BC_4118226/2020 EPI_ISL_418825 2020-03-10 BCCDC Public Health Laboratory BCCDC Public Health Laboratory Harrigan et al

Canada/BC_4122951/2020 EPI_ISL_418826 2020-03-10 BCCDC Public Health Laboratory BCCDC Public Health Laboratory Harrigan et al

Canada/BC_4540462/2020 EPI_ISL_418829 2020-03-10 BCCDC Public Health Laboratory BCCDC Public Health Laboratory Harrigan et al

Canada/BC_6004567/2020 EPI_ISL_418836 2020-03-10 BCCDC Public Health Laboratory BCCDC Public Health Laboratory Harrigan et al

Canada/BC_6129127/2020 EPI_ISL_418837 2020-03-10 BCCDC Public Health Laboratory BCCDC Public Health Laboratory Harrigan et al

Canada/BC_6489864/2020 EPI_ISL_418839 2020-03-10 BCCDC Public Health Laboratory BCCDC Public Health Laboratory Harrigan et al

Canada/MB_2/2020 EPI_ISL_429807 2020-03-10 Cadham Provincial Laboratory National Microbiology Laboratory Anna Majer et al

Canada/ON_PHL0977/2020 EPI_ISL_418365 2020-03-10 Public Health Ontario Laboratories Public Health Ontario Laboratories Alireza Eshaghi et al

Canada/ON_PHL3501/2020 EPI_ISL_418333 2020-03-10 Public Health Ontario Laboratories Public Health Ontario Laboratories Alireza Eshaghi et al

Canada/ON_PHL3536/2020 EPI_ISL_418362 2020-03-10 Public Health Ontario Laboratories Public Health Ontario Laboratories Alireza Eshaghi et al

Canada/ON_PHL8539/2020 EPI_ISL_418369 2020-03-10 Public Health Ontario Laboratories Public Health Ontario Laboratories Alireza Eshaghi et al

Chile/Santiago_10/2020 EPI_ISL_445253 2020-03-10 CLINICA ALEMANA DE SANTIAGO S.A. Instituto de Salud Publica de Chile Andr√©s E Castillo et al

Chile/Santiago_12/2020 EPI_ISL_445255 2020-03-10 CLINICA ALEMANA DE SANTIAGO S.A. Instituto de Salud Publica de Chile Andr√©s E Castillo et al

Chile/Santiago_13/2020 EPI_ISL_445256 2020-03-10 CLINICA LAS CONDES S.A. Instituto de Salud Publica de Chile Andr√©s E Castillo et al

Chile/Santiago_14/2020 EPI_ISL_445257 2020-03-10 CLINICA TABANCURA Instituto de Salud Publica de Chile Andr√©s E Castillo et al

Chile/Santiago_16/2020 EPI_ISL_445259 2020-03-10 CLINICA LAS CONDES S.A. Instituto de Salud Publica de Chile Andr√©s E Castillo et al

Chile/Santiago_8/2020 EPI_ISL_445251 2020-03-10 HOSPITAL DE CARABINEROS Instituto de Salud Publica de Chile Andr√©s E Castillo et al

Denmark/ALAB-HH01/2020 EPI_ISL_429262 2020-03-10 Department of Clinical Microbiology, Copenhagen University Hospital, Hvidovre, Kettegaard Alle 30, 2650 Hvidovre. Albertsen lab, Department of Chemistry and Bioscience, Aalborg University, Denmark Rasmus Kirkegaard et al

Denmark/ALAB-HH02/2020 EPI_ISL_429263 2020-03-10 Department of Clinical Microbiology, Copenhagen University Hospital, Hvidovre, Kettegaard Alle 30, 2650 Hvidovre. Albertsen lab, Department of Chemistry and Bioscience, Aalborg University, Denmark Rasmus Kirkegaard et al

Denmark/ALAB-HH03/2020 EPI_ISL_429264 2020-03-10 Department of Clinical Microbiology, Copenhagen University Hospital, Hvidovre, Kettegaard Alle 30, 2650 Hvidovre. Albertsen lab, Department of Chemistry and Bioscience, Aalborg University, Denmark Rasmus Kirkegaard et al

Denmark/ALAB-HH04/2020 EPI_ISL_429265 2020-03-10 Department of Clinical Microbiology, Copenhagen University Hospital, Hvidovre, Kettegaard Alle 30, 2650 Hvidovre. Albertsen lab, Department of Chemistry and Bioscience, Aalborg University, Denmark Rasmus Kirkegaard et al

Denmark/ALAB-HH05/2020 EPI_ISL_429266 2020-03-10 Department of Clinical Microbiology, Copenhagen University Hospital, Hvidovre, Kettegaard Alle 30, 2650 Hvidovre. Albertsen lab, Department of Chemistry and Bioscience, Aalborg University, Denmark Rasmus Kirkegaard et al

Denmark/ALAB-HH06/2020 EPI_ISL_429267 2020-03-10 Department of Clinical Microbiology, Copenhagen University Hospital, Hvidovre, Kettegaard Alle 30, 2650 Hvidovre. Albertsen lab, Department of Chemistry and Bioscience, Aalborg University, Denmark Rasmus Kirkegaard et al

Denmark/ALAB-HH07/2020 EPI_ISL_429268 2020-03-10 Department of Clinical Microbiology, Copenhagen University Hospital, Hvidovre, Kettegaard Alle 30, 2650 Hvidovre. Albertsen lab, Department of Chemistry and Bioscience, Aalborg University, Denmark Rasmus Kirkegaard et al

Denmark/ALAB-HH08/2020 EPI_ISL_429269 2020-03-10 Department of Clinical Microbiology, Copenhagen University Hospital, Hvidovre, Kettegaard Alle 30, 2650 Hvidovre. Albertsen lab, Department of Chemistry and Bioscience, Aalborg University, Denmark Rasmus Kirkegaard et al

Denmark/ALAB-HH11/2020 EPI_ISL_429270 2020-03-10 Department of Clinical Microbiology, Copenhagen University Hospital, Hvidovre, Kettegaard Alle 30, 2650 Hvidovre. Albertsen lab, Department of Chemistry and Bioscience, Aalborg University, Denmark Rasmus Kirkegaard et al

Denmark/ALAB-HH13/2020 EPI_ISL_429271 2020-03-10 Department of Clinical Microbiology, Copenhagen University Hospital, Hvidovre, Kettegaard Alle 30, 2650 Hvidovre. Albertsen lab, Department of Chemistry and Bioscience, Aalborg University, Denmark Rasmus Kirkegaard et al

Denmark/ALAB-HH14/2020 EPI_ISL_429272 2020-03-10 Department of Clinical Microbiology, Copenhagen University Hospital, Hvidovre, Kettegaard Alle 30, 2650 Hvidovre. Albertsen lab, Department of Chemistry and Bioscience, Aalborg University, Denmark Rasmus Kirkegaard et al

Denmark/ALAB-HH15/2020 EPI_ISL_429273 2020-03-10 Department of Clinical Microbiology, Copenhagen University Hospital, Hvidovre, Kettegaard Alle 30, 2650 Hvidovre. Albertsen lab, Department of Chemistry and Bioscience, Aalborg University, Denmark Rasmus Kirkegaard et al

Denmark/ALAB-HH16/2020 EPI_ISL_429274 2020-03-10 Department of Clinical Microbiology, Copenhagen University Hospital, Hvidovre, Kettegaard Alle 30, 2650 Hvidovre. Albertsen lab, Department of Chemistry and Bioscience, Aalborg University, Denmark Rasmus Kirkegaard et al

Denmark/ALAB-HH17/2020 EPI_ISL_429275 2020-03-10 Department of Clinical Microbiology, Copenhagen University Hospital, Hvidovre, Kettegaard Alle 30, 2650 Hvidovre. Albertsen lab, Department of Chemistry and Bioscience, Aalborg University, Denmark Rasmus Kirkegaard et al

Denmark/ALAB-HH18/2020 EPI_ISL_429276 2020-03-10 Department of Clinical Microbiology, Copenhagen University Hospital, Hvidovre, Kettegaard Alle 30, 2650 Hvidovre. Albertsen lab, Department of Chemistry and Bioscience, Aalborg University, Denmark Rasmus Kirkegaard et al

Denmark/ALAB-HH19/2020 EPI_ISL_429277 2020-03-10 Department of Clinical Microbiology, Copenhagen University Hospital, Hvidovre, Kettegaard Alle 30, 2650 Hvidovre. Albertsen lab, Department of Chemistry and Bioscience, Aalborg University, Denmark Rasmus Kirkegaard et al

Denmark/ALAB-HH20/2020 EPI_ISL_429278 2020-03-10 Department of Clinical Microbiology, Copenhagen University Hospital, Hvidovre, Kettegaard Alle 30, 2650 Hvidovre. Albertsen lab, Department of Chemistry and Bioscience, Aalborg University, Denmark Rasmus Kirkegaard et al

Denmark/ALAB-HH21/2020 EPI_ISL_429279 2020-03-10 Department of Clinical Microbiology, Copenhagen University Hospital, Hvidovre, Kettegaard Alle 30, 2650 Hvidovre. Albertsen lab, Department of Chemistry and Bioscience, Aalborg University, Denmark Rasmus Kirkegaard et al

Denmark/ALAB-HH22/2020 EPI_ISL_429280 2020-03-10 Department of Clinical Microbiology, Copenhagen University Hospital, Hvidovre, Kettegaard Alle 30, 2650 Hvidovre. Albertsen lab, Department of Chemistry and Bioscience, Aalborg University, Denmark Rasmus Kirkegaard et al

Denmark/ALAB-HH23/2020 EPI_ISL_429281 2020-03-10 Department of Clinical Microbiology, Copenhagen University Hospital, Hvidovre, Kettegaard Alle 30, 2650 Hvidovre. Albertsen lab, Department of Chemistry and Bioscience, Aalborg University, Denmark Rasmus Kirkegaard et al

Denmark/ALAB-SSI-1272/2020 EPI_ISL_444841 2020-03-10 Department of Virus and Microbiological Special Diagnostics, Statens Serum Institut, Copenhagen, Denmark, Artillerivej 5, 2300 Copenahgen S Albertsen lab, Department of Chemistry and Bioscience, Aalborg University, Denmark Rasmus Kirkegaard et al

Denmark/ALAB-SSI-242/2020 EPI_ISL_437657 2020-03-10 Department of Virus and Microbiological Special Diagnostics, Statens Serum Institut, Copenhagen, Denmark, Artillerivej 5, 2300 Copenahgen S Albertsen lab, Department of Chemistry and Bioscience, Aalborg University, Denmark Rasmus Kirkegaard et al

Denmark/ALAB-SSI-244/2020 EPI_ISL_437659 2020-03-10 Department of Virus and Microbiological Special Diagnostics, Statens Serum Institut, Copenhagen, Denmark, Artillerivej 5, 2300 Copenahgen S Albertsen lab, Department of Chemistry and Bioscience, Aalborg University, Denmark Rasmus Kirkegaard et al

Denmark/ALAB-SSI-246/2020 EPI_ISL_437661 2020-03-10 Department of Virus and Microbiological Special Diagnostics, Statens Serum Institut, Copenhagen, Denmark, Artillerivej 5, 2300 Copenahgen S Albertsen lab, Department of Chemistry and Bioscience, Aalborg University, Denmark Rasmus Kirkegaard et al

Denmark/ALAB-SSI-251/2020 EPI_ISL_437664 2020-03-10 Department of Virus and Microbiological Special Diagnostics, Statens Serum Institut, Copenhagen, Denmark, Artillerivej 5, 2300 Copenahgen S Albertsen lab, Department of Chemistry and Bioscience, Aalborg University, Denmark Rasmus Kirkegaard et al

Denmark/ALAB-SSI-284/2020 EPI_ISL_436965 2020-03-10 Department of Virus and Microbiological Special Diagnostics, Statens Serum Institut, Copenhagen, Denmark, Artillerivej 5, 2300 Copenahgen S Albertsen lab, Department of Chemistry and Bioscience, Aalborg University, Denmark Rasmus Kirkegaard et al

Denmark/ALAB-SSI155/2020 EPI_ISL_429371 2020-03-10 Department of Virus and Microbiological Special Diagnostics, Statens Serum Institut, Copenhagen, Denmark, Artillerivej 5, 2300 Copenahgen S Albertsen lab, Department of Chemistry and Bioscience, Aalborg University, Denmark Rasmus Kirkegaard et al

Denmark/ALAB-SSI156/2020 EPI_ISL_429372 2020-03-10 Department of Virus and Microbiological Special Diagnostics, Statens Serum Institut, Copenhagen, Denmark, Artillerivej 5, 2300 Copenahgen S Albertsen lab, Department of Chemistry and Bioscience, Aalborg University, Denmark Rasmus Kirkegaard et al

Denmark/ALAB-SSI207/2020 EPI_ISL_429404 2020-03-10 Department of Virus and Microbiological Special Diagnostics, Statens Serum Institut, Copenhagen, Denmark, Artillerivej 5, 2300 Copenahgen S Albertsen lab, Department of Chemistry and Bioscience, Aalborg University, Denmark Rasmus Kirkegaard et al

Denmark/ALAB-SSI209/2020 EPI_ISL_429405 2020-03-10 Department of Virus and Microbiological Special Diagnostics, Statens Serum Institut, Copenhagen, Denmark, Artillerivej 5, 2300 Copenahgen S Albertsen lab, Department of Chemistry and Bioscience, Aalborg University, Denmark Rasmus Kirkegaard et al

Denmark/ALAB-SSI216/2020 EPI_ISL_429408 2020-03-10 Department of Virus and Microbiological Special Diagnostics, Statens Serum Institut, Copenhagen, Denmark, Artillerivej 5, 2300 Copenahgen S Albertsen lab, Department of Chemistry and Bioscience, Aalborg University, Denmark Rasmus Kirkegaard et al

Denmark/ALAB-SSI221/2020 EPI_ISL_429410 2020-03-10 Department of Virus and Microbiological Special Diagnostics, Statens Serum Institut, Copenhagen, Denmark, Artillerivej 5, 2300 Copenahgen S Albertsen lab, Department of Chemistry and Bioscience, Aalborg University, Denmark Rasmus Kirkegaard et al

Denmark/ALAB-SSI258/2020 EPI_ISL_429420 2020-03-10 Department of Virus and Microbiological Special Diagnostics, Statens Serum Institut, Copenhagen, Denmark, Artillerivej 5, 2300 Copenahgen S Albertsen lab, Department of Chemistry and Bioscience, Aalborg University, Denmark Rasmus Kirkegaard et al

Denmark/ALAB-SSI259/2020 EPI_ISL_429421 2020-03-10 Department of Virus and Microbiological Special Diagnostics, Statens Serum Institut, Copenhagen, Denmark, Artillerivej 5, 2300 Copenahgen S Albertsen lab, Department of Chemistry and Bioscience, Aalborg University, Denmark Rasmus Kirkegaard et al

Denmark/ALAB-SSI262/2020 EPI_ISL_429424 2020-03-10 Department of Virus and Microbiological Special Diagnostics, Statens Serum Institut, Copenhagen, Denmark, Artillerivej 5, 2300 Copenahgen S Albertsen lab, Department of Chemistry and Bioscience, Aalborg University, Denmark Rasmus Kirkegaard et al

Denmark/ALAB-SSI263/2020 EPI_ISL_429425 2020-03-10 Department of Virus and Microbiological Special Diagnostics, Statens Serum Institut, Copenhagen, Denmark, Artillerivej 5, 2300 Copenahgen S Albertsen lab, Department of Chemistry and Bioscience, Aalborg University, Denmark Rasmus Kirkegaard et al

Denmark/ALAB-SSI265/2020 EPI_ISL_429427 2020-03-10 Department of Virus and Microbiological Special Diagnostics, Statens Serum Institut, Copenhagen, Denmark, Artillerivej 5, 2300 Copenahgen S Albertsen lab, Department of Chemistry and Bioscience, Aalborg University, Denmark Rasmus Kirkegaard et al

Denmark/ALAB-SSI268/2020 EPI_ISL_429428 2020-03-10 Department of Virus and Microbiological Special Diagnostics, Statens Serum Institut, Copenhagen, Denmark, Artillerivej 5, 2300 Copenahgen S Albertsen lab, Department of Chemistry and Bioscience, Aalborg University, Denmark Rasmus Kirkegaard et al

Denmark/ALAB-SSI271/2020 EPI_ISL_429431 2020-03-10 Department of Virus and Microbiological Special Diagnostics, Statens Serum Institut, Copenhagen, Denmark, Artillerivej 5, 2300 Copenahgen S Albertsen lab, Department of Chemistry and Bioscience, Aalborg University, Denmark Rasmus Kirkegaard et al

Denmark/ALAB-SSI272/2020 EPI_ISL_429433 2020-03-10 Department of Virus and Microbiological Special Diagnostics, Statens Serum Institut, Copenhagen, Denmark, Artillerivej 5, 2300 Copenahgen S Albertsen lab, Department of Chemistry and Bioscience, Aalborg University, Denmark Rasmus Kirkegaard et al

Denmark/ALAB-SSI273/2020 EPI_ISL_429434 2020-03-10 Department of Virus and Microbiological Special Diagnostics, Statens Serum Institut, Copenhagen, Denmark, Artillerivej 5, 2300 Copenahgen S Albertsen lab, Department of Chemistry and Bioscience, Aalborg University, Denmark Rasmus Kirkegaard et al

Denmark/ALAB-SSI279/2020 EPI_ISL_429439 2020-03-10 Department of Virus and Microbiological Special Diagnostics, Statens Serum Institut, Copenhagen, Denmark, Artillerivej 5, 2300 Copenahgen S Albertsen lab, Department of Chemistry and Bioscience, Aalborg University, Denmark Rasmus Kirkegaard et al

Denmark/ALAB-SSI280/2020 EPI_ISL_429440 2020-03-10 Department of Virus and Microbiological Special Diagnostics, Statens Serum Institut, Copenhagen, Denmark, Artillerivej 5, 2300 Copenahgen S Albertsen lab, Department of Chemistry and Bioscience, Aalborg University, Denmark Rasmus Kirkegaard et al

Denmark/ALAB-SSI293/2020 EPI_ISL_429448 2020-03-10 Department of Virus and Microbiological Special Diagnostics, Statens Serum Institut, Copenhagen, Denmark, Artillerivej 5, 2300 Copenahgen S Albertsen lab, Department of Chemistry and Bioscience, Aalborg University, Denmark Rasmus Kirkegaard et al

Denmark/ALAB-SSI298/2020 EPI_ISL_429449 2020-03-10 Department of Virus and Microbiological Special Diagnostics, Statens Serum Institut, Copenhagen, Denmark, Artillerivej 5, 2300 Copenahgen S Albertsen lab, Department of Chemistry and Bioscience, Aalborg University, Denmark Rasmus Kirkegaard et al

Denmark/ALAB-SSI299/2020 EPI_ISL_429450 2020-03-10 Department of Virus and Microbiological Special Diagnostics, Statens Serum Institut, Copenhagen, Denmark, Artillerivej 5, 2300 Copenahgen S Albertsen lab, Department of Chemistry and Bioscience, Aalborg University, Denmark Rasmus Kirkegaard et al

Denmark/ALAB-SSI300/2020 EPI_ISL_429451 2020-03-10 Department of Virus and Microbiological Special Diagnostics, Statens Serum Institut, Copenhagen, Denmark, Artillerivej 5, 2300 Copenahgen S Albertsen lab, Department of Chemistry and Bioscience, Aalborg University, Denmark Rasmus Kirkegaard et al

Denmark/ALAB-SSI301/2020 EPI_ISL_429452 2020-03-10 Department of Virus and Microbiological Special Diagnostics, Statens Serum Institut, Copenhagen, Denmark, Artillerivej 5, 2300 Copenahgen S Albertsen lab, Department of Chemistry and Bioscience, Aalborg University, Denmark Rasmus Kirkegaard et al

Denmark/ALAB-SSI302/2020 EPI_ISL_429453 2020-03-10 Department of Virus and Microbiological Special Diagnostics, Statens Serum Institut, Copenhagen, Denmark, Artillerivej 5, 2300 Copenahgen S Albertsen lab, Department of Chemistry and Bioscience, Aalborg University, Denmark Rasmus Kirkegaard et al

Denmark/ALAB-SSI407/2020 EPI_ISL_429501 2020-03-10 Department of Virus and Microbiological Special Diagnostics, Statens Serum Institut, Copenhagen, Denmark, Artillerivej 5, 2300 Copenahgen S Albertsen lab, Department of Chemistry and Bioscience, Aalborg University, Denmark Rasmus Kirkegaard et al

Denmark/ALAB-SSI408/2020 EPI_ISL_429502 2020-03-10 Department of Virus and Microbiological Special Diagnostics, Statens Serum Institut, Copenhagen, Denmark, Artillerivej 5, 2300 Copenahgen S Albertsen lab, Department of Chemistry and Bioscience, Aalborg University, Denmark Rasmus Kirkegaard et al

Denmark/ALAB-SSI409/2020 EPI_ISL_429503 2020-03-10 Department of Virus and Microbiological Special Diagnostics, Statens Serum Institut, Copenhagen, Denmark, Artillerivej 5, 2300 Copenahgen S Albertsen lab, Department of Chemistry and Bioscience, Aalborg University, Denmark Rasmus Kirkegaard et al

Denmark/ALAB-SSI411/2020 EPI_ISL_429505 2020-03-10 Department of Virus and Microbiological Special Diagnostics, Statens Serum Institut, Copenhagen, Denmark, Artillerivej 5, 2300 Copenahgen S Albertsen lab, Department of Chemistry and Bioscience, Aalborg University, Denmark Rasmus Kirkegaard et al

Denmark/ALAB-SSI412/2020 EPI_ISL_429507 2020-03-10 Department of Virus and Microbiological Special Diagnostics, Statens Serum Institut, Copenhagen, Denmark, Artillerivej 5, 2300 Copenahgen S Albertsen lab, Department of Chemistry and Bioscience, Aalborg University, Denmark Rasmus Kirkegaard et al

Denmark/ALAB-SSI413/2020 EPI_ISL_429509 2020-03-10 Department of Virus and Microbiological Special Diagnostics, Statens Serum Institut, Copenhagen, Denmark, Artillerivej 5, 2300 Copenahgen S Albertsen lab, Department of Chemistry and Bioscience, Aalborg University, Denmark Rasmus Kirkegaard et al

Denmark/ALAB-SSI414/2020 EPI_ISL_429511 2020-03-10 Department of Virus and Microbiological Special Diagnostics, Statens Serum Institut, Copenhagen, Denmark, Artillerivej 5, 2300 Copenahgen S Albertsen lab, Department of Chemistry and Bioscience, Aalborg University, Denmark Rasmus Kirkegaard et al

Denmark/ALAB-SSI416/2020 EPI_ISL_429513 2020-03-10 Department of Virus and Microbiological Special Diagnostics, Statens Serum Institut, Copenhagen, Denmark, Artillerivej 5, 2300 Copenahgen S Albertsen lab, Department of Chemistry and Bioscience, Aalborg University, Denmark Rasmus Kirkegaard et al

Denmark/ALAB-SSI417/2020 EPI_ISL_429514 2020-03-10 Department of Virus and Microbiological Special Diagnostics, Statens Serum Institut, Copenhagen, Denmark, Artillerivej 5, 2300 Copenahgen S Albertsen lab, Department of Chemistry and Bioscience, Aalborg University, Denmark Rasmus Kirkegaard et al

Denmark/ALAB-SSI418/2020 EPI_ISL_429516 2020-03-10 Department of Virus and Microbiological Special Diagnostics, Statens Serum Institut, Copenhagen, Denmark, Artillerivej 5, 2300 Copenahgen S Albertsen lab, Department of Chemistry and Bioscience, Aalborg University, Denmark Rasmus Kirkegaard et al

England/20122074602/2020 EPI_ISL_418676 2020-03-10 Respiratory Virus Unit, Microbiology Services Colindale, Public Health England Respiratory Virus Unit, Microbiology Services Colindale, Public Health England Monica Galiano et al

England/BRIS-12186A/2020 EPI_ISL_440250 2020-03-10 PHE South West Regional Laboratory, National Infection Service Wellcome Sanger Institute for the COVID-19 Genomics UK Consortium Stephanie Hutchings et al

England/CAMB-846B7/2020 EPI_ISL_440492 2020-03-10 Department of Pathology, University of Cambridge Wellcome Sanger Institute for the COVID-19 Genomics UK Consortium Luke W Meredith et al

England/CAMB-846E4/2020 EPI_ISL_440534 2020-03-10 Department of Pathology, University of Cambridge Wellcome Sanger Institute for the COVID-19 Genomics UK Consortium Luke W Meredith et al

England/CAMB-84A9D/2020 EPI_ISL_440519 2020-03-10 Department of Pathology, University of Cambridge Wellcome Sanger Institute for the COVID-19 Genomics UK Consortium Luke W Meredith et al

England/CAMB-84B7C/2020 EPI_ISL_440553 2020-03-10 Department of Pathology, University of Cambridge Wellcome Sanger Institute for the COVID-19 Genomics UK Consortium Luke W Meredith et al

France/HF2234/2020 EPI_ISL_416495 2020-03-10 Centre Hospitalier Compi√®gne Laboratoire de Biologie National Reference Center for Viruses of Respiratory Infections, Institut Pasteur, Paris M√©lnie Albert et al

France/HF2237/2020 EPI_ISL_416496 2020-03-10 Centre Hospitalier Compi√®gne Laboratoire de Biologie National Reference Center for Viruses of Respiratory Infections, Institut Pasteur, Paris M√©lnie Albert et al

France/HF2239/2020 EPI_ISL_416497 2020-03-10 Centre Hospitalier Compi√®gne Laboratoire de Biologie National Reference Center for Viruses of Respiratory Infections, Institut Pasteur, Paris M√©lnie Albert et al

France/Pollionay_1733/2020 EPI_ISL_416745 2020-03-10 CNR Virus des Infections Respiratoires - France SUD CNR Virus des Infections Respiratoires - France SUD Bal et al

Fuyang/FY002/2020 EPI_ISL_424352 2020-03-10 unknown Clinical Laboratory Ge et al
[truncated: 2,332,097 more chars]
